# GeoEPred: A multimodal structure-aware geometric deep learning framework for Gram-negative bacterial secreted effector prediction with sequence semantics

**DOI:** 10.1371/journal.pcbi.1014344

**Published:** 2026-09-30

**Authors:** Shouzhen Song, Hua Shi, Hongfeng Wu, Dachen Liu, Yihang Lin, Nor Ashidi Mat Isa, Quan Zou, Leyi Wei

**Affiliations:** 1 School of Opto-electronic and Communication Engineering, Xiamen University of Technology, Xiamen, Fujian, China; 2 School of Electrical and Electronic Engineering, Engineering Campus, Universiti Sains Malaysia, Nibong Tebal, Pulau Pinang, Malaysia; 3 Yangtze Delta Region Institute (Quzhou), University of Electronic Science and Technology of China, Quzhou, Zhejiang, China; 4 Institute of Fundamental and Frontier Sciences, University of Electronic Science and Technology of China, Chengdu, Sichuan, China; 5 Faculty of Applied Sciences, Macao Polytechnic University, Macao, China; CSIR-IHBT: Institute of Himalayan Bioresource Technology CSIR, INDIA

## Abstract

Accurate prediction of effector proteins secreted by Gram-negative bacteria is important for elucidating bacterial pathogenic mechanisms and developing precise anti-infective strategies. Although existing methods have benefited from the strong sequence feature extraction capacity of pretrained protein language models, reliance on linear sequence information alone often fails to fully capture the three-dimensional conformational signals required for virulence functions. Meanwhile, conventional structure-based methods are limited by the scarcity of experimentally resolved protein structures. To address these challenges, we propose GeoEPred, a multimodal deep learning framework designed for the synergistic modeling of protein sequence and structure to identify Gram-negative bacterial effector proteins. Specifically, the model integrates sequence-contextual embeddings from a pretrained protein language model with three-dimensional structural representations predicted by ESMFold. A feature projection network refines fine-grained sequence signals associated with effector functions, while geometric vector perceptrons characterize inter-residue orientations, distances, and local spatial topology to capture potential structural conformational motifs. To further enable effective cross-modal fusion, we design a cross-modal alignment and feature-tokenized self-attention module. This module enhances consistency between the sequence-semantic and structural-geometric spaces through contrastive learning and models associations between linear functional motifs and spatial conformational patterns at a fine-grained token level. Extensive evaluations on multiple benchmark datasets show that GeoEPred achieves better predictive performance than existing leading models in T3SE, T4SE, and T6SE prediction tasks, while maintaining stable performance in remote homolog recognition scenarios. Moreover, the modular and extensible architecture of GeoEPred demonstrates strong generalization ability and substantial application potential for genome-scale effector protein discovery.

## Author summary

Secreted effector proteins are central virulence factors used by many Gram-negative bacterial pathogens to execute infection strategies. Their functions are governed not only by secretion signals and short linear motifs in the amino acid sequence, but also by three-dimensional folds, local domains, and surface geometric patterns. However, current predictors mainly exploit sequence-contextual features, limiting their ability to model the correspondence between linear sequence signals and spatial conformational motifs, and thereby constraining accuracy and interpretability. Here, we present GeoEPred, a multimodal deep learning framework for secreted effector protein identification. GeoEPred couples sequence-semantic embeddings from a pretrained protein language model with structural representations learned by geometric vector perceptrons. A cross-modal alignment and interaction module uses contrastive learning to improve functional consistency between sequence and structure modalities, while feature-token attention captures fine-grained links between key linear and conformational motifs. Across benchmark datasets covering multiple effector types, GeoEPred outperforms existing state-of-the-art methods and provides interpretable evidence from sequence fragments, structural regions, and cross-modal associations, supporting functional annotation, pathogenic mechanism analysis, and experimental validation.

## Introduction

Gram-negative bacteria deploy specialized secretion systems to deliver virulence effector proteins that establish infection by modulating host signaling and evading immune responses [1–4]. Systematic discovery and functional profiling of these proteins are therefore essential for elucidating the molecular basis of pathogenesis and developing targeted antibacterial interventions. Although effectors are increasingly identified through experimental screening approaches, such as secretomic analyses [5,6], these methods are often labor-intensive and time-consuming, and their outcomes can be influenced by variation in protein expression and secretion levels. To address these limitations, a range of computational tools has been developed, providing more efficient and cost-effective alternatives for effector protein identification.

Early computational tools for effector prediction largely relied on conventional feature engineering. For example, Bastion3 [7] and Bastion6 [8] integrated evolutionary conservation represented by position-specific scoring matrices (PSSMs) with structural constraint information, enabling effective prediction of type III and type VI effectors, respectively. CNN-T4SE combined PSSMs, protein secondary structure, solvent accessibility, and one-hot encoding to improve T4SE identification [9]. However, further optimization of such models is limited by the scarcity of annotated samples and by the substantial heterogeneity of effectors in sequence composition, secretion signals, and functional motifs, which constrains their ability to learn robust features from limited data [10].

In recent years, protein language models (PLMs) have provided new opportunities for effector protein prediction. By learning contextual representations enriched with evolutionary and functional information from large-scale protein sequences, PLMs can improve model generalization in low-data settings [11,12]. Building on this advantage, DeepSecE [13] introduced a secretion-specific Transformer module to capture secretion-related patterns from PLM-derived sequence embeddings, whereas MoCETSE [14] combined a hybrid convolutional expert module with a Transformer incorporating relative positional encoding to further extract key discriminative features from PLM representations. Although these approaches have improved effector prediction, their inputs remain restricted to one-dimensional sequence information and do not explicitly exploit functional cues encoded in protein three-dimensional structures. Given that protein function is often jointly governed by tertiary conformation and spatial residue–residue interactions, sequence-based representations alone may be insufficient to fully characterize functionally diverse bacterial effectors [15–17].

A major reason for this limitation is the scarcity of experimentally resolved effector structures, which has hindered the systematic integration of structural features into prediction models. AlphaFold has transformed protein structure prediction [18], enabling large-scale access to high-quality structural models, and predicted structures have shown practical value in studies of intrinsically disordered proteins. However, the high computational cost of AlphaFold2 limits its application to genome-scale analyses. To address this issue, Lin et al. developed ESMFold [11], a structure prediction model that substantially improves inference efficiency while maintaining high prediction accuracy, accelerating structure prediction by up to approximately 60-fold. The efficiency of ESMFold makes large-scale protein structure analysis more feasible and provides a practical route for incorporating three-dimensional structural information into bacterial effector prediction [19,20].

In this study, we propose GeoEPred, an end-to-end framework for effector protein prediction, designed to overcome the limitations of existing methods that rely heavily on sequence-derived features and insufficiently exploit protein spatial conformational information. GeoEPred first employs the pretrained ESM-2 model to extract protein embeddings, which are subsequently refined by a feature projection network to learn discriminative representations associated with virulence-related functions. To incorporate structure-level functional information, GeoEPred uses a geometric vector perceptron (GVP) module to extract three-dimensional conformational features from protein structures predicted by ESMFold. Unlike conventional graph neural networks that are restricted to scalar feature representations, GVP jointly models scalar and vector information, thereby capturing residue-level spatial relationships and local geometric orientations with higher fidelity. Furthermore, GeoEPred introduces a contrastive learning module to enhance cross-modal consistency between sequence semantic representations and structural geometric representations. It also employs a feature-tokenized multi-head attention mechanism to enable fine-grained interaction modeling, explicitly learning the latent associations between linear sequence signals and three-dimensional conformational motifs. GeoEPred not only outperforms existing mainstream binary classification models, but also achieves greater precision than widely used multi-class classifiers. The proposed method is also applicable to practical scenarios involving multiple types of effector proteins, enabling the simultaneous identification of diverse effectors without relying on explicit parameterization of physicochemical properties. Overall, our model provides a new perspective on the application of three-dimensional structural information to effector prediction and may help deepen our understanding of pathogenic mechanisms associated with bacterial secretion systems.

## Materials and methods

### Dataset construction

The datasets used for model training and evaluation were compiled from datasets released with established effector prediction methods and from widely used biological databases.

The dataset construction and redundancy-removal procedures were designed with reference to DeepSecE [13], a representative study in the field. Specifically, T1SE and T2SE sequences were obtained from the TSE-ARF dataset [21]. The T3SE and T6SE datasets were compiled by integrating sequences from Bastion3 [7], DeepT3 [22], SecReT6 [23], Bastion6 [8], DeepT3_4 [24], and TSE-ARF [21]. The T4SE dataset was assembled from DeepSecE [13], OPT4e [25], T4SEfinder [26], iT4SE-EP [27], Bastion4 [28], T4SE-XGB [29], TSE-ARF [21], T4SEpp [30], and DeepT3_4 [24]. Following previous representative studies [31,32], the negative class was constructed using curated non-secreted proteins and homologous sequences from selected pathogenic bacteria reported in earlier studies. These candidate negative samples had been further screened against multiple bacterial genomes using BLASTp in the original studies, based on predefined thresholds for the *E*-value and sequence identity. To provide an objective assessment of model generalization, we additionally assembled an independent test set. The T3SE, T4SE, T6SE, and non-secreted protein samples were collected from the test datasets released with Bastion3, CNN-T4SE [9], and Bastion6, whereas the T1SE and T2SE samples were randomly selected from the original BastionHub dataset.

To minimize potential overestimation of model performance caused by sequence redundancy, CD-HIT v4.8.1 [33] was applied separately to the candidate training set and the independent test set. A global sequence identity threshold of 60% was used, and the aligned region was required to cover at least 80% of both the shorter and longer sequences. Within each dataset, sequences from all effector and non-effector classes were pooled before clustering, and only one representative sequence was retained from each cluster. Cross-dataset filtering was subsequently performed using CD-HIT-2D under the same identity and coverage criteria. The non-redundant independent test set was used as the reference dataset, and candidate training sequences showing at least 60% global sequence identity to any test sequence were removed. This procedure minimized the risk of information leakage between the training and test sets.

The data sources for each effector type and the corresponding sample numbers after CD-HIT filtering are summarized in S1 Table. The resulting training set comprised 1,575 non-effector proteins, 128 T1SEs, 65 T2SEs, 505 T3SEs, 623 T4SEs, and 259 T6SEs. The independent test set contained 150 non-effector proteins, 20 T1SEs, 10 T2SEs, 30 T3SEs, 30 T4SEs, and 20 T6SEs (S1 Fig A). Protein lengths ranged from fewer than 100 to more than 1,000 amino acids, with most sequences falling between 100 and 700 amino acids (S1 Fig B).

### The architecture of the model

We propose GeoEPred, a novel approach that incorporates structural features to enhance effector protein prediction (Fig 1A). Protein sequences are input into the pretrained protein language models ESMFold and ESM-2, producing predicted structural PDB files and 2,560-dimensional sequence embeddings, respectively. The sequence embeddings are projected to 256 dimensions through a feature projection network. Concurrently, based on the predicted backbone structures, proteins are represented as residue-level neighbor graphs, in which each node corresponds to an amino acid residue and its spatial position is defined by the Cα atom coordinates. Edges connect each residue to its nearest neighbors in three-dimensional space, with scalar and geometric vector features assigned to nodes and edges. A Geometric Vector Perceptron (GVP) is embedded in the graph neural network to jointly aggregate scalar and vector features from neighboring nodes and edges during message passing and feedforward updates, thereby capturing geometric relationships, local orientations, and residue interaction patterns. After aggregating these features into tokens, multimodal information is integrated via two modules: a contrastive learning module and a feature-token attention module. Contrastive learning aligns sequence semantic and structural geometric information in a unified representation space, enabling the model to capture their latent biological relationships. The feature-token mechanism partitions features into semantically distinct tokens and employs attention to model inter-token dependencies, allowing fine-grained interactions between sequence signals and structural conformations.

**Fig 1:**
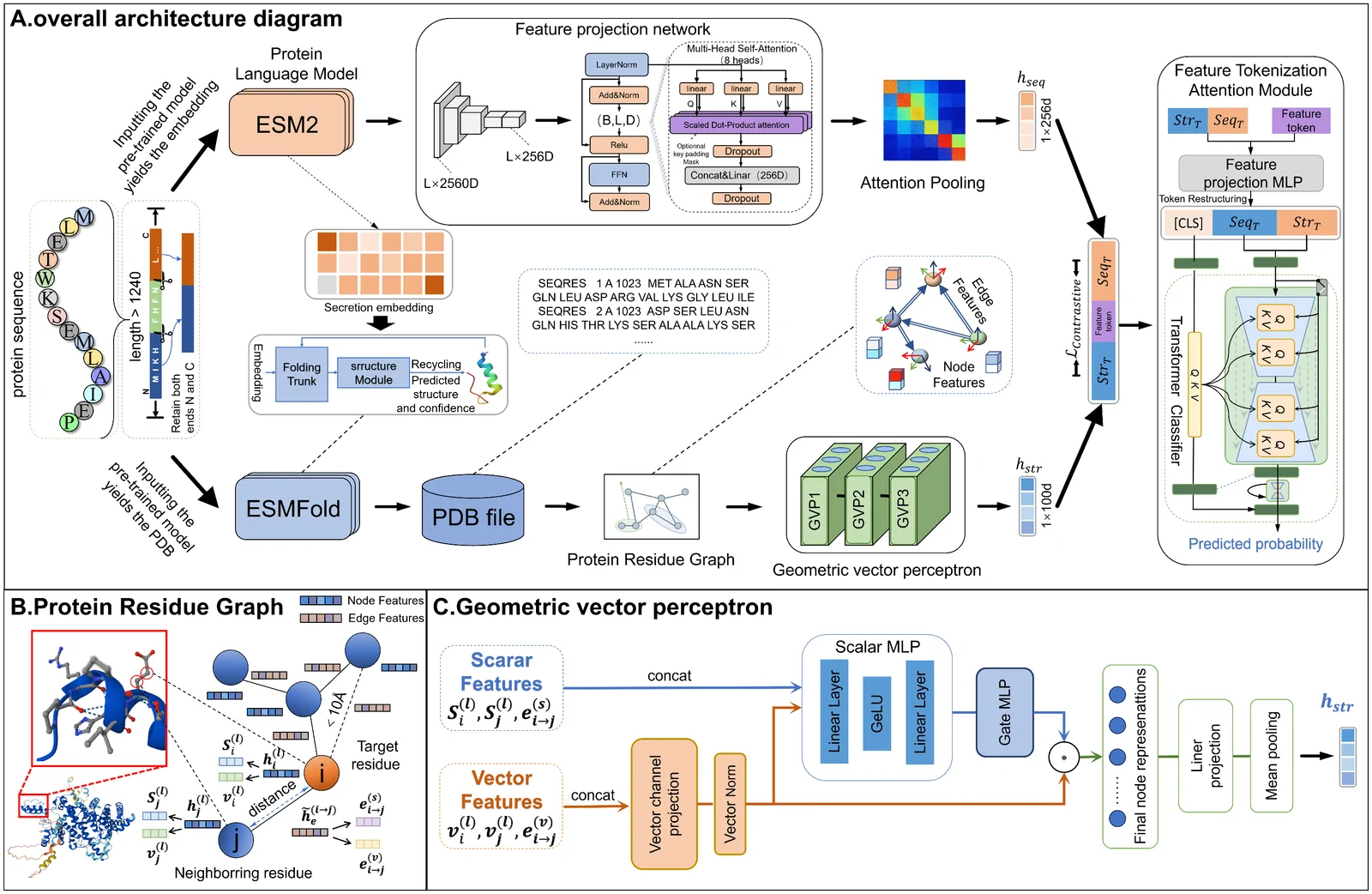
The overall workflow of GeoEPred. A: Effector protein sequences are initially encoded using a pretrained protein language model to extract contextual evolutionary embeddings, which are subsequently refined through a feature projection network to generate sequence-level semantic representations. Concurrently, the input sequences are submitted to ESMFold to predict their three-dimensional conformations. Based on the predicted structures, protein residue graphs are constructed and processed with GVP-based message passing to learn geometry-aware structural representations. The resulting sequence semantics and structural features are then organized into distinct modality-specific feature tokens for downstream cross-modal representation learning. B: Construction of protein residue graph. C: Geometric vector perceptron.

### Protein language model

Previous studies have demonstrated that embeddings generated by protein language models contain rich information about protein sequence patterns and properties [34,35]. This capability has promoted their widespread use in tasks involving protein sequence prediction and functional annotation. ESM-2 [11] is a Transformer-based protein language model comprising 33 layers and approximately 650 million parameters. It was pretrained using a self-supervised masked-language-modeling objective on approximately 65 million unique protein sequences sampled from approximately 43 million UniRef50 clusters derived from approximately 138 million UniRef90 sequences, enabling it to learn general evolutionary, biochemical, and structural patterns from unlabeled protein data. Building on ESM-2 representations, ESMFold [11] predicts atomic-level protein structures directly from individual amino acid sequences without requiring multiple sequence alignments or structural templates. In this study, the weights of both ESM-2 and ESMFold remained frozen throughout the subsequent training and evaluation processes.

To determine the optimal protein language model (PLM) for sequence semantics, we evaluated several state-of-the-art architectures (S3 Table). We employ ESM-2 (t36) [11] with 650 million parameters to generate initial sequence semantic representations. During downstream training, the ESM-2 weights remain frozen to preserve the general biological patterns acquired from large-scale pretraining while reducing computational overhead. The parameter sources for each PLM are listed in S4 Table.

Building on these advances, we incorporated an ESMFold-based structural branch into our model. Three-dimensional structures predicted directly from protein sequences were used as structural inputs to extract geometric and conformational features, enabling the model to characterize proteins from a spatial perspective. To assess the reliability of the structures predicted by ESMFold, we calculated the per-residue predicted Local Distance Difference Test (pLDDT) scores for all protein structures in the training and test sets. The pLDDT score ranges from 0 to 100 and provides a residue-level estimate of local structural confidence, with higher scores indicating more reliable predictions. Summary statistics for the structural confidence of the two datasets are presented in Table 1. The mean pLDDT score was approximately 77 for both the training and test sets, indicating that the predicted structures generally exhibited good confidence. Moreover, the pLDDT distributions were comparable between the two datasets, suggesting no evident shift in structural quality between the training and test sets. Approximately 40% of the residues fell within the high-confidence range (pLDDT ≥90), indicating that a substantial proportion of the predicted structures provided a reliable structural basis for subsequent geometric feature extraction and protein classification.

**Table 1:** Quality of ESMFold-predicted structures and their pLDDT distributions.

Metric	Training Set	Testing Set
pLDDT (Mean ± SD)	76.67 ± 17.65	78.57 ± 17.80
Median pLDDT	82.43	84.36
pLDDT ≥90 (High Confidence)	40.42%	44.17%
70≤ pLDDT < 90 (Medium Confidence)	26.41%	24.33%
pLDDT < 70 (Low Confidence)	26.64%	23.90%

### Feature projection network

The raw ESM-2 embeddings $H_{esm}\in {\mathbb{R}}^{L\times 2560}$ represent high-dimensional sequence features. We constructed a feature projection network to project these embeddings into a 256-dimensional latent space and model inter-residue dependencies:


$$\begin{array}{c} H_{esm}^{\prime }={Transformer}_{seq}((W_{proj}H_{esm}^{⊤}{)}^{⊤}),\;H_{esm}^{\prime }\in {\mathbb{R}}^{L\times 256} \end{array}$$ (1)

where $W_{proj}\in {\mathbb{R}}^{256\times 2560}$ is the projection matrix for dimensionality reduction, and ${Transformer}_{seq}$ captures long-range contextual patterns discriminative for effector identification.

Since the functional activity of effector proteins often relies on localized regions, such as N-terminal signal peptides or conserved motifs, global average pooling may dilute these critical site-specific signals. To address this, we develop a residue-adaptive attention pooling module that learns task-specific residue importance and selectively aggregates secretion- and virulence-related sequence representations. Specifically, let $H_{esm}^{\prime }=[h_{1}^{\prime },h_{2}^{\prime },...,h_{L}^{\prime }{]}^{⊤}$ denote the set of refined semantic vectors for a protein sequence of length *L*, where each $h_{i}^{\prime }\in {\mathbb{R}}^{d}$ is the *d*-dimensional column vector representing the *i*-th residue. The importance score $a_{i}$ for the *i*-th residue is defined as:


$$\begin{array}{c} a_{i}=w_{attn}^{⊤}\tanh(W_{attn}h_{i}^{\prime }+b_{attn}) \end{array}$$ (2)

where $W_{attn}\in {\mathbb{R}}^{k\times d}$ and $b_{attn}\in {\mathbb{R}}^{k}$ denote the weight matrix and bias vector that project the semantics into an attention space, $w_{attn}\in {\mathbb{R}}^{k}$ is the weight vector that produces the raw importance scores (*d* = 256 and *k* = 64). The final global sequence semantic representation $h_{seq}$ is obtained via a weighted sum of the residue features:


$$\begin{array}{c} h_{seq}=\sum_{i=1}^{L}\frac{\exp(a_{i})}{\sum_{j=1}^{L}\exp(a_{j})}h_{i}^{\prime } \end{array}$$ (3)

This mechanism prioritizes key functional segments while maintaining a comprehensive global context, providing a precise semantic input for subsequent multimodal fusion.

### Protein residue graph construction

To encode the three-dimensional structural information of proteins, each predicted protein structure is represented as a residue-level spatial graph *G*=(*V*,*E*) (Fig 1B). Each node corresponds to an amino acid residue, with its spatial position defined by the $C_{\alpha }$ atom coordinate, and its initial representation consists of scalar and vector features describing local residue geometry. Scalar features include normalized backbone distances, the $N-C_{\alpha }-C$ bond angle and its sine, and amino acid type index. Vector features capture local residue orientation, including the unit vector from $C_{\alpha }$ to *C*, the normal vector of the backbone plane, and the corresponding orthogonal direction. The initial node representation $h_{i}^{\left(0\right)}$ consists of scalar features $s_{i}^{\left(0\right)}$ and vector features $V_{i}^{\left(0\right)}$, serving as input to the subsequent GVP-based structural encoder.

Edges are constructed based on spatial proximity between residues. An edge is added if two residues are within 10 Å $C_{\alpha }-C_{\alpha }$ distance, and sequentially adjacent residues are always connected in both directions to preserve backbone continuity. Each directed edge $e_{j\to i}$ contains both scalar features $e_{j\to i}^{\left(s\right)}$ and vector features $e_{j\to i}^{\left(v\right)}$, with scalar and vector features defined as:


$$\begin{array}{cc} e_{j\to i}^{\left(s\right)} & =[\exp(-\frac{(d_{ij}-\mu_{k}{)}^{2}}{2}){]}_{k=1}^{32}, \end{array}$$ (4)

$$\begin{array}{cc} e_{j\to i}^{\left(v\right)} & =\frac{x_{j}-x_{i}}{\|x_{j}-x_{i}{\|}_{2}+\epsilon }, \end{array}$$ (5)

where $d_{ij}$ is the Euclidean distance between residues *i* and *j*, $\mu_{k}$ is the *k*-th radial basis function center evenly spaced between 0 and 20 Å, and ϵ is a small constant for numerical stability.

The resulting residue-level graph *G* includes node scalar features $s_{i}^{\left(0\right)}$, node vector features $V_{i}^{\left(0\right)}$, edge indices, and edge scalar and vector features.

### Geometric vector perceptron

To further encode the three-dimensional structural information of the residue graph, we implemented a Geometric Vector Perceptron (GVP)-based structural encoder [36]. By embedding GVPs into the graph message-passing process, the model generates messages from both node and edge geometric features, simultaneously updating scalar and vector node representations. This design enables the model to capture directional geometric information that is difficult to represent using scalar-only graph neural networks.

As illustrated in Fig 1C, each residue node at layer *l* is represented as $h_{i}^{\left(l\right)}=(s_{i}^{\left(l\right)},V_{i}^{\left(l\right)})$, where $s_{i}^{\left(l\right)}$ and $V_{i}^{\left(l\right)}$ denote the scalar and vector features, respectively:


$$\begin{array}{c} h_{i}^{\left(l\right)}=\left(s_{i}^{\left(l\right)},V_{i}^{\left(l\right)}\right). \end{array}$$ (6)

Each edge j→i has embedded features ${\tilde{h}}_{e}^{\left(j\to i\right)}$, which also contain scalar and vector components. In each GVP-based graph convolution layer, messages are generated from the source node, target node, and edge features. Specifically, these features are integrated into a joint representation and passed into a GVP-based message function $g^{\left(l\right)}(\cdot )$:


$$\begin{array}{c} h_{m}^{\left(j\to i,l\right)}=g^{\left(l\right)}(concat(h_{j}^{\left(l\right)},h_{i}^{\left(l\right)},{\tilde{h}}_{e}^{\left(j\to i\right)})), \end{array}$$ (7)

where $h_{m}^{\left(j\to i,l\right)}$ contains both scalar and vector messages. Within $g^{\left(l\right)}(\cdot )$, vector features are transformed and coupled with scalar features through vector norms and scalar-dependent gating, enabling the generated message to encode both invariant and directional geometric information. Therefore, the information propagated along each edge depends not only on scalar residue representations, but also on geometric vector features such as edge directions, distance encodings, and local residue coordinate frames.

Incoming messages are aggregated by mean pooling and added to the current node representation through a residual connection:


$$\begin{array}{c} h_{i}^{\left(l+1\right)}=h_{i}^{\left(l\right)}+\frac{1}{\left|𝒩(i)\right|}\sum_{j\in 𝒩(i)}h_{m}^{\left(j\to i,l\right)}, \end{array}$$ (8)

where 𝒩(i) denotes the set of incoming neighbors of node *i*. This mean aggregation reduces the influence of node-degree variation and enables stable integration of local neighborhood information.

After GVP-based message passing, the updated node representations encode geometric information through the joint propagation of scalar and vector channels. We then apply mean pooling over the final scalar node representations to obtain the protein-level structural embedding:


$$\begin{array}{c} h_{str}=W_{o}\left(\frac{1}{\left|V\right|}\sum_{i\in V}s_{i}^{\left(L_{g}\right)}\right), \end{array}$$ (9)

where $W_{o}$ denotes the output linear projection, *V* is the set of residue nodes, and $s_{i}^{\left(L_{g}\right)}$ is the final scalar representation of residue *i* after $L_{g}$ GVP-based graph convolution layers. Through the joint scalar–vector updates in GVP-based graph convolution layers, directional geometric information encoded by the vector channels is incorporated into the final node representations. Consequently, the structural embedding $h_{str}$ captures both residue-level topological relationships and three-dimensional geometric context, and is subsequently used as the structural representation for multimodal fusion and downstream prediction tasks.

### Cross-modal feature tokenization

After obtaining the protein structural embedding $h_{str}$ from the GVP module, the model integrates it with the sequence embedding $h_{seq}$ produced by the sequence encoder. To enhance the interaction between sequence and structure modalities, the two global embeddings are first normalized and projected into a shared *d*-dimensional latent space to construct modality-level feature tokens. In addition, independent cross-modal projection layers are used to generate a coupled interaction token through element-wise multiplication:


$$\begin{array}{cc} T_{seq} & =LN(h_{seq})W_{seq}, \end{array}$$ (10)

$$\begin{array}{cc} T_{str} & =LN(h_{str})W_{str}, \end{array}$$ (11)

$$\begin{array}{cc} T_{cross} & =\tanh\left(LN(h_{seq})W_{seq}^{c}\right)⊙\tanh\left(LN(h_{str})W_{str}^{c}\right), \end{array}$$ (12)

where $W_{seq}$ and $W_{str}$ are learnable projection matrices for the sequence and structure modalities, respectively. $W_{seq}^{c}$ and $W_{str}^{c}$ denote independent projection matrices for constructing the cross-modal interaction token, and ⊙ denotes element-wise multiplication. The resulting $T_{seq}$, $T_{str}$, and $T_{cross}$ represent the sequence feature token, structural feature token, and cross-modal interaction token, respectively.

A learnable classification token $T_{cls}$ is then concatenated with the three feature tokens. Positional embeddings $E_{pos}$ are added to the token sequence, which is subsequently fed into a multi-layer Transformer encoder. The Transformer self-attention mechanism models dependencies among the sequence-specific, structure-specific, and cross-modal interaction representations:


$$\begin{array}{c} Z_{L}=Transformer\left([T_{cls};T_{seq};T_{str};T_{cross}]+E_{pos}\right), \end{array}$$ (13)

where $Z_{L}$ denotes the final token representations after *L* Transformer encoder layers. The output corresponding to the classification token, denoted as $z_{cls}$, aggregates global cross-modal context and is passed to a classification head to generate the final prediction.

During training, the model is optimized using a joint objective that combines the classification loss and the cross-modal contrastive loss. The classification loss ${ℒ}_{cls}$ is implemented as Focal Loss with label smoothing, while the contrastive loss ${ℒ}_{con}$ adopts the NT-Xent objective to encourage consistency between sequence and structure representations in the projection space:


$$\begin{array}{c} {ℒ}_{total}={ℒ}_{cls}+\lambda {ℒ}_{con}, \end{array}$$ (14)

where λ controls the relative contribution of the contrastive learning objective.

By applying Transformer-based self-attention to the sequence, structure, and coupled interaction tokens, the model can emphasize discriminative modality-specific and cross-modal representations, thereby forming a unified protein representation for downstream effector classification and secretion-system type prediction.

### Model training

GeoEPred was trained using stratified five-fold cross-validation, with all experiments repeated using five different random seeds (42, 52, 62, 72, and 82). For each random seed, the available training data were partitioned into training and validation subsets within each fold, and the model checkpoint achieving the highest macro-F1 score on the validation set was selected and retained. The independent test set remained completely isolated throughout model training, hyperparameter determination, and model selection, and was used solely for the final performance evaluation [37].

The model was implemented in PyTorch v2.8.0 and executed on an NVIDIA A100 GPU with 40 GB of memory. The software environment consisted of Python 3.12.9 and CUDA 12.8. Model parameters were initialized using Xavier initialization and optimized using AdamW with a weight decay of $1\times 10^{-4}$. The initial learning rates were set to $2\times 10^{-5}$ for the sequence encoder, $3\times 10^{-4}$ for the structure encoder, and $1\times 10^{-4}$ for the remaining modules. The learning rates were updated after each epoch using cosine annealing with warm restarts, with an initial cycle length of 20 epochs and a cycle-length multiplier of 2 (*T*_0_ = 20 and $T_{mult}=2$).

The batch size and maximum number of training epochs were set to 32 and 30, respectively. Early stopping was performed by monitoring the macro-F1 score on the validation set, with a patience of five epochs, and the checkpoint achieving the best validation performance in each fold was retained. Automatic mixed precision and dynamic loss scaling were employed during training, and the global gradient norm was clipped to a maximum value of 1.0.

Class imbalance was addressed using inverse-class-frequency weighted random sampling. Focal loss was adopted as the primary classification objective, with a focusing parameter of 2.0 and a label-smoothing coefficient of 0.05. The sequence-modality dropout probability was set to 0.25, whereas a conventional dropout rate of 0.1 was applied within the sequence Transformer encoder, cross-modal attention module, and Transformer classifier.

## Results

### Evaluation of GeoEPred performance in effector protein prediction

GeoEPred was evaluated using five-fold cross-validation, with the validation sets split from the training set (Dataset S1). For the multi-class classification task, we adopted a one-vs-rest strategy to calculate the ROC curve for each class and aggregated the prediction results from the validation sets across all folds for overall evaluation. As shown in Fig 2A, the AUC values for the non-effector class and the five major effector protein classes ranged from 0.936 to 0.978, indicating that the model was able to discriminate among different classes. Fig 2B further presents the multi-class confusion matrix derived from the aggregated prediction results, with the percentages for each class rounded to one decimal place. GeoEPred achieved a recall of approximately 93.7% for non-effector proteins, indicating that the model could distinguish non-effectors from effectors. In comparison, the recall for T2SE was lower at 77.9%, which may be related to the relatively limited number of samples from this class in the training data.

**Fig 2:**
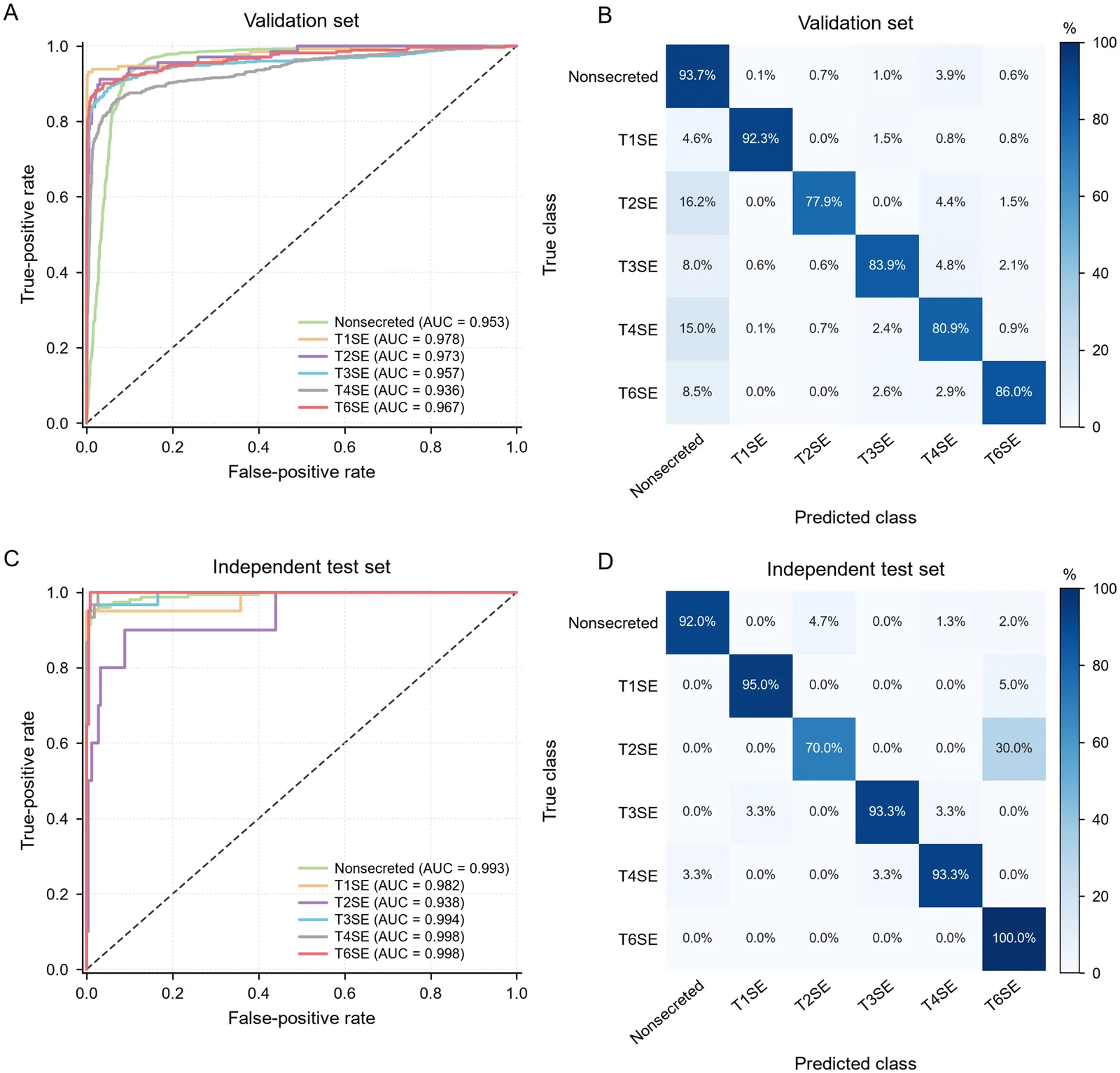
Assessing the predictive efficacy of GeoEPred in identifying bacterial secretion system effectors. A–D: Model performance assessed via five-fold cross-validation (A, B) and independent testing **(C, D)**. ROC curves illustrate the classification performance for each effector type. Sensitivity values for each class are indicated along the diagonals of the corresponding confusion matrices.

In addition, we evaluated the generalization ability of GeoEPred on an independent test set that was completely separated from the model training process. As shown in Fig 2C, the AUC values across the different classes ranged from 0.938 to 0.998, indicating that the model retained its ability to discriminate among classes on independent data. The confusion matrix further showed that GeoEPred achieved a recall of 100.0% for T6SE, while the recalls for T1SE, T2SE, T3SE, and T4SE were 95.0%, 70.0%, 93.3%, and 93.3%, respectively (Fig 2D). Overall, these results indicate that GeoEPred can identify different types of secreted effector proteins on the independent test set and exhibits generalization capability.

### Ablation study of characteristic sources and fusion strategies

To assess the contribution of each modality and evaluate the synergistic effect of the multimodal fusion module, we performed a comprehensive ablation study. All methodological variants were subjected to a uniform training-validation pipeline with standardized hyperparameters and metrics to ensure the integrity of cross-comparisons.

### Multimodal feature ablation

We systematically disentangled the contributions of the cross-modal architecture by evaluating the predictive utility of structure-only features, sequence-only features, refined sequence-semantic representations, and the final multimodal fusion representation. Table 2 presents a detailed quantitative ablation analysis of each feature configuration under five-fold cross-validation and on the independent test set.

**Table 2:** Performance evaluation of multimodal feature ablation.

Feature Type	ACC		F1		AUPRC	
	CV	Ind	CV	Ind	CV	Ind
Geometric Priors	0.615	0.527	0.541	0.459	0.561	0.454
Sequence only	0.681	0.723	0.639	0.664	0.681	0.683
Sequence Semantics	0.877	0.881	0.854	0.846	0.893	0.907
GeoEPred	**0.899**	**0.923**	**0.880**	**0.884**	**0.907**	**0.915**

Under five-fold cross-validation, models relying solely on sequence or structural features exhibited limited discriminative ability, achieving accuracies of 0.681 (95% CI: 0.668–0.694) and 0.615 (95% CI: 0.571–0.660), respectively, with corresponding F1 scores of 0.639 (95% CI: 0.616–0.663) and 0.541 (95% CI: 0.486–0.596). Incorporating refined sequence-semantic representations substantially improved the accuracy to 0.877 (95% CI: 0.860–0.895) and the F1 score to 0.854 (95% CI: 0.823–0.885). The multimodal fusion representation achieved the best overall performance, with an accuracy of 0.899 (95% CI: 0.878–0.920) and an F1 score of 0.880 (95% CI: 0.853–0.907).

When evaluated on the independent test set, the multimodal fusion features outperformed the second-best refined sequence-semantic features, improving accuracy from 0.881 to 0.923 (95% CI: 0.0115–0.0731, *p* = 0.008) and the F1 score from 0.846 to 0.884 (95% CI: 0.0069–0.0702, *p* = 0.014). Notably, by incorporating structural features, the multimodal collaborative framework achieved an MCC gain of 0.0604 over the sequence-semantic baseline (95% CI: 0.0194–0.1043, *p* = 0.002), providing evidence for the strong representational complementarity between evolutionary sequence semantics and spatial geometric features (S5 Table). By leveraging the combined strengths of both modalities, the fusion mechanism expanded the model’s decision boundary and enhanced its predictive robustness.

To validate the above quantitative findings, Fig 3 employs Uniform Manifold Approximation and Projection (UMAP) [38] to visualize the latent feature space of validation samples under different feature configurations. When sequence or structural features are used in isolation, sample embeddings exhibit substantial dispersion, with severe feature overlap between positive and negative classes, directly leading to high misclassification rates. Conversely, introducing ESM embedding alone markedly improves cluster homogeneity, but the centers of all clusters remain relatively close, causing samples near the decision boundary to be easily confused. After the embedded representations are refined to condense information, a clearer separation emerges between effector and non-effector proteins, although inter-effector classification remains suboptimal. In contrast, the joint fusion of sequence semantics and structural features yields the most discriminative topology: positive and negative samples form dense clusters in the high-dimensional embedding space with well-defined, mutually separated boundaries. This visual evidence supports the complementary role of cross-modal integration in enhancing representation separability and provides an intuitive explanation for the observed differences in ACC, MCC, and AUC. AUC evaluates the overall ranking capability across all classification thresholds, which is consistent with the observation that both models achieve a certain degree of class separation in the embedding space. In contrast, ACC and MCC reflect classification performance under a specific decision threshold and are therefore more sensitive to the clarity of class boundaries. Statistical significance analysis further showed that GeoEPred achieved improvements of 4.23% in ACC (*p* = 0.0080) and 6.04% in MCC (*p* = 0.0020) compared with the refined sequence semantic model, whereas only a minor difference was observed in AUC. These results suggest that incorporating structural features improves the discrimination of samples located near decision boundaries, leading to the observed improvements in ACC and MCC (S5 Table).

**Fig 3:**
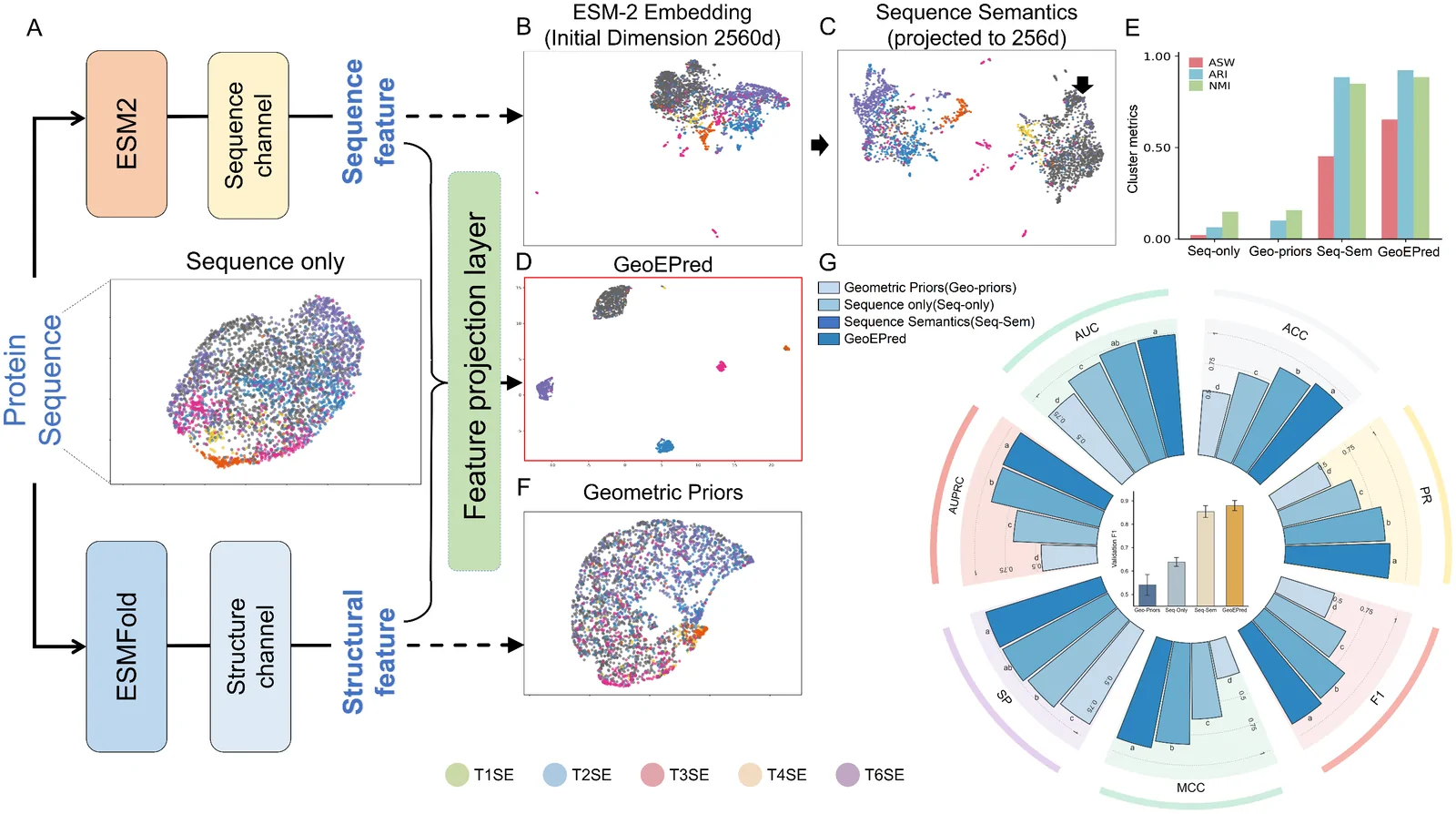
Ablation Analysis and Latent Space Visualization of GeoEPred. A: Multi-modal Input Streams: Schematic representation of the sequence features (ESM-2 embeddings and refined sequence semantics) and the structural features in isolation. B: UMAP visualizations of effector and non-effector protein clusters based on ESM-2 embedding vectors (2560 dimensions). C: Refined Sequence Semantics: UMAP visualization of protein clusters after sequence-channel projection and contextual encoding (256 dimensions). D: Multimodal UMAP of Effectors: UMAP visualization showing the precise clustering of effectors and non-effectors after integrating both sequence semantics and structural features. E: Bar plots compare clustering performance using the evaluation metrics NMI, ARI, and ASW. F: Structural features: UMAP visualization of effector and non-effector clusters derived from the GVP. G: A ring diagram compares ablation metrics, with the center representing the weighted F1.

Three clustering performance metrics—Adjusted Rand Index (ARI), Normalized Mutual Information (NMI), and Average Silhouette Width (ASW)—further indicate that modality fusion enhances the clustering capability of the output representations (Fig 3E). Compared with a single modality, GeoEPred shows improvements in ARI and NMI, suggesting a stronger ability to discriminate among different effectors.

### Precise classification of proteins with distant homology

In biochemistry, protein function is primarily determined by its three-dimensional structure rather than solely by its amino acid sequence [39]. Notably, some proteins can maintain highly similar spatial structures despite low sequence identity, thereby exhibiting similar functional properties. Compared with conventional sequence alignment methods, structural alignment is capable of capturing functional relationships across greater evolutionary distances [40]. In general, protein pairs with sequence identity below 0.3 and TM-score above 0.5 are considered to exhibit significant structural similarity and are often regarded as remote homologs [41].

To further validate the necessity of incorporating structural information, we performed a focused ablation study under the extreme “long-range homology” scenario. Specifically, we curated a dedicated benchmark of 107 proteins (S3 Fig), each satisfying the remote-homology criterion with respect to the training set. As shown in Fig 4, the structures of samples in the training set and the independent test set have a certain degree of overlap in 3D space.

**Fig 4:**
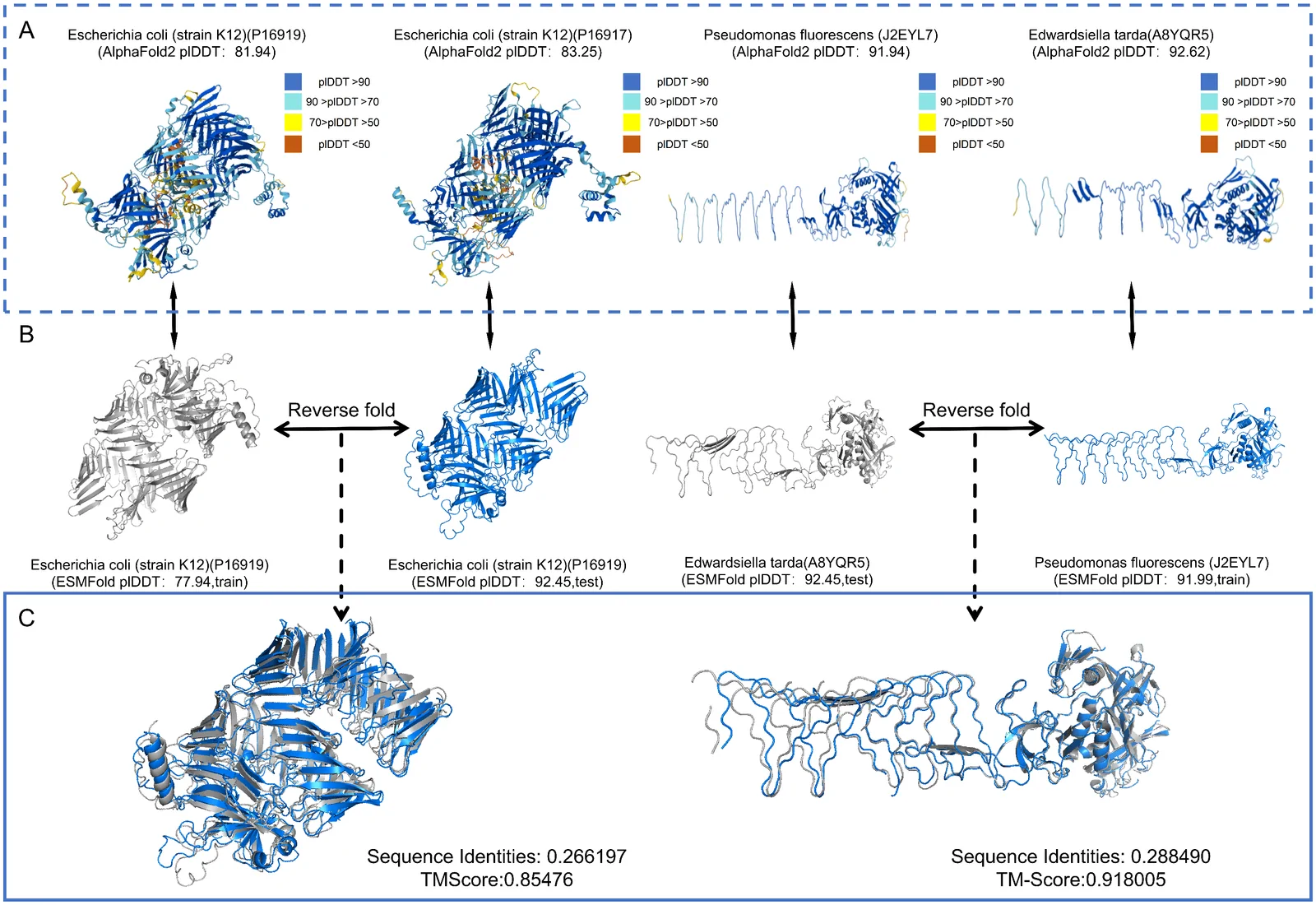
The degree of spatial overlap between the 3D structures of distant homologs. A: Three-dimensional protein structures predicted by AlphaFold2, colored according to pLDDT scores (higher scores indicate higher confidence). B: Three-dimensional protein structures predicted by ESMFold. C: The middle panel displays the structural alignment of two proteins from each group. “Train” indicates samples from the training set, while “Test” indicates samples from the test set. Sequence identity represents the percentage of aligned amino acid positions that are identical between two protein sequences. TM-score quantifies the structural similarity between two protein structures, with higher scores indicating greater similarity.

On this benchmark, we compared GeoEPred with two ablated variants: one relying on sequence semantics refined by the feature projection network alone, and the other using only raw sequence features. The raw-sequence variant performs poorly, reaching an accuracy of just 65.42%, and misclassifies 23 of the 38 T3SE samples into other categories. Substituting raw features with sequence semantics produces a marked improvement, raising the number of correctly classified samples to 88. This gain shows that contextual representations from pretrained protein language models, coupled with the feature projection network that distills fine-grained functional signals, markedly reduce the model’s reliance on superficial sequence similarity. GeoEPred further lifts the accuracy to 90.65% (S6 Table), delivering the best performance on this benchmark. The result confirms that integrating refined sequence semantics with geometric structural features is the principal driver of performance gains in this challenging scenario.

### Model component evaluation

We conducted a series of module ablation experiments to evaluate the contribution of each core component to the model’s overall performance. Specifically, we compared the complete GeoEPred model with three variants: (1) a model combining adaptive pooling and feature tokenization but excluding contrastive learning; (2) a model combining feature tokenization and contrastive learning but excluding adaptive pooling; and (3) a model combining adaptive pooling and contrastive learning but excluding feature tokenization.

As shown in Table 3, in five-fold cross-validation, the model combining adaptive attention pooling and contrastive learning achieved an accuracy (ACC) of 0.861 (95% CI: 0.842–0.880) and an F1 score of 0.829 (95% CI: 0.799–0.859). Upon introducing feature tokenization, the accuracy (ACC) and F1 score improved to 0.899 (95% CI: 0.878–0.920) and 0.880 (95% CI: 0.853–0.907), respectively. When adaptive attention pooling was replaced by mean pooling, the accuracy and F1 score dropped to 0.886 (95% CI: 0.868–0.904) and 0.863 (95% CI: 0.835–0.891), respectively. On the independent test set, removing contrastive learning resulted in decreases of 3.00% (95% CI: 0.00002–0.0602, *p* = 0.0519) in F1 and 4.85% (95% CI: 0.0094–0.0910, *p* = 0.0140) in MCC. Replacing adaptive attention pooling with mean pooling resulted in decreases of 3.38% (95% CI: −0.0071–0.0816, *p* = 0.0939) in F1 and 5.32% (95% CI: 0.0083–0.1014, *p* = 0.0220) in MCC. Regarding AUPRC—a metric particularly informative under class imbalance—GeoEPred achieved a score of 0.9146, representing a 3.94% improvement over the model without feature tokenization (0.8753; 95% CI: 0.0112–0.0888, *p* = 0.0040). F1 and MCC also improved by 4.61% (95% CI: 0.0181–0.0764, *p* = 0.0020) and 6.83% (95% CI: 0.0303–0.1085, *p* = 0.0020), respectively (S7 Table). These results indicate that feature tokenization helps maintain precision–recall performance and enhances the quality of probability predictions for the minority class.

**Table 3:** Performance comparison of model variants in the ablation study using ACC, F1, and AUPRC on cross-validation and independent test sets.

Model Variant	ACC		F1		AUPRC	
	CV	Ind	CV	Ind	CV	Ind
w/o Contrastive Learning	0.881	0.889	0.851	0.854	0.901	0.887
w/o Adaptive Attention Pooling	0.886	0.885	0.863	0.851	0.899	0.890
w/o Feature Tokenization	0.861	0.873	0.829	0.838	0.864	0.875
Full Model (Ours)	**0.899**	**0.923**	**0.880**	**0.884**	**0.907**	**0.915**

Building on the preceding results indicating that feature tokenization contributes to model performance, we further compared different feature integration strategies. Specifically, feature tokenization, the core fusion mechanism of GeoEPred, was compared with cross-attention fusion and feature concatenation.

As shown in Table 4, the feature tokenization method exhibited improved performance across the evaluated metrics. In five-fold cross-validation, it achieved an accuracy of 0.899 (95% CI: 0.878–0.920), outperforming direct concatenation (0.884; 95% CI: 0.849–0.919) and cross-attention fusion (0.886; 95% CI: 0.860–0.912) by 0.015 and 0.013, respectively. On the independent test set, our fusion strategy improved the AUPRC by 0.0521 and 0.0415 compared with feature concatenation and cross-attention fusion, respectively. In S8 Table, stratified bootstrap hypothesis testing confirmed that these AUPRC improvements were statistically significant (*p* = 0.002 vs. concatenation; *p* = 0.012 vs. cross-attention fusion). Although improvements in some threshold-dependent metrics were not statistically significant, this does not contradict the AUPRC findings, as these metrics capture different aspects of model performance. AUPRC evaluates the precision–recall trade-off across varying decision thresholds and is particularly informative under imbalanced class distributions. Collectively, these results suggest that token-level multimodal fusion provides an effective integration mechanism within the GeoEPred framework compared with attention-based interaction and simple feature concatenation.

**Table 4:** Performance evaluation metrics for different fusion strategies.

Method	ACC		F1		AUPRC	
	CV	Ind	CV	Ind	CV	Ind
Concatenation	0.884	0.889	0.860	0.848	0.854	0.863
Cross-Attention Fusion	0.886	0.908	0.863	0.862	0.901	0.873
Feature Tokenization	**0.899**	**0.923**	**0.880**	**0.884**	**0.907**	**0.915**

### Interpretable investigation into multimodal feature contributions

To further investigate the underlying mechanisms by which multimodal fusion improves performance over unimodal approaches, we conducted SHAP (SHapley Additive exPlanations) attribution analysis. Based on cooperative game theory, SHAP provides a unified framework for global and local interpretability by quantifying the importance of individual features, thereby enabling a clear characterization of how features from different modalities are utilized in model predictions.

The class-specific feature contribution patterns are illustrated in Fig 5A. Refined sequence semantics (Sequence Semantics), including esm_f211, esm_f165, esm_f117, and esm_f85, occupy many of the highly influential positions across the six classes, indicating that sequence representations constitute the major source of discriminative information. These ESM-derived latent features may capture sequence-context information and evolutionary constraints learned from large-scale protein sequences, thereby providing informative signals for effector classification. Structural features, such as struct_f68, struct_f30, struct_f61, and struct_f88, also exhibit substantial contributions across different classes, supporting the complementary role of structural information in multimodal prediction.

**Fig 5:**
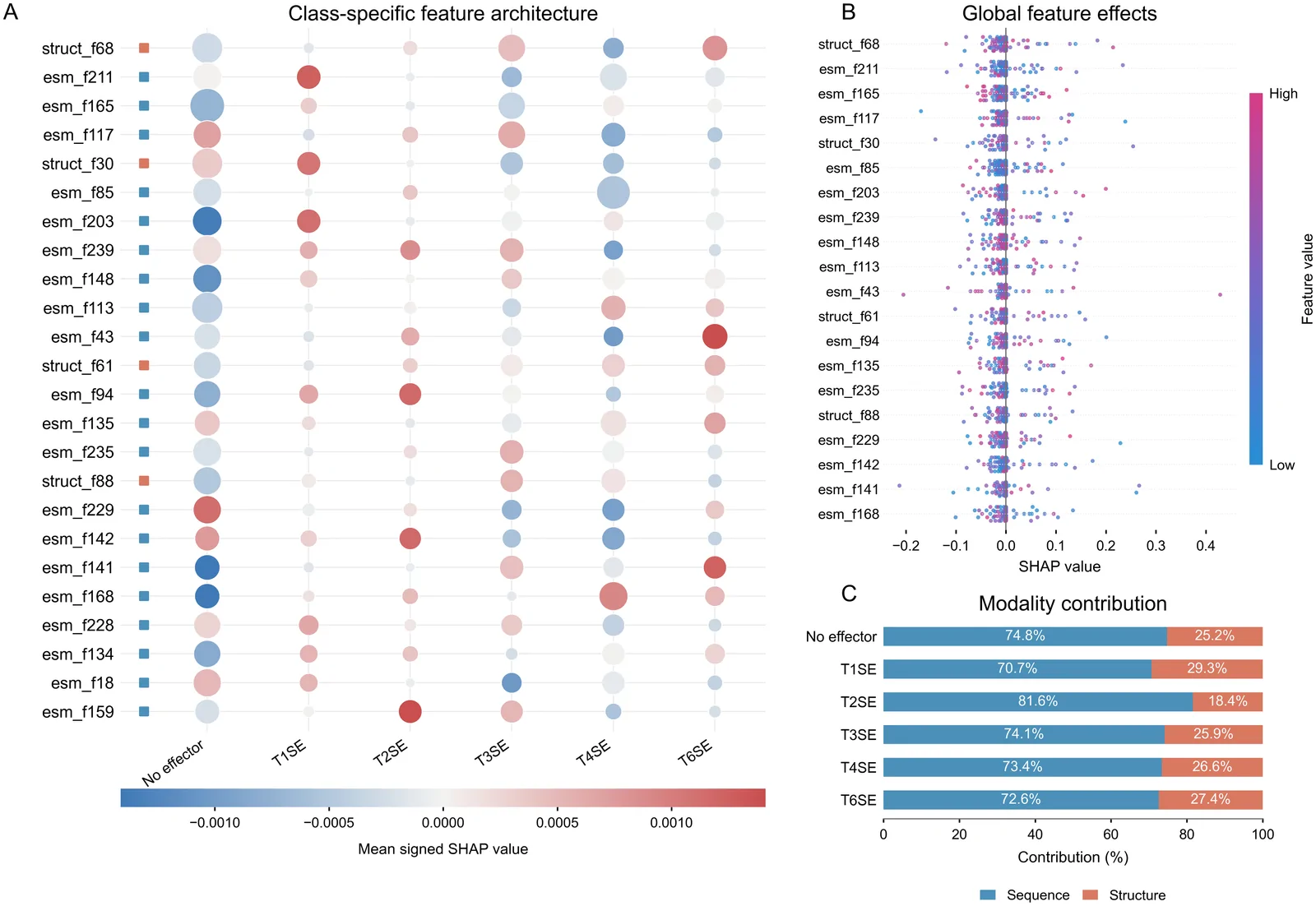
Interpretability analysis of GeoEPred. A: Class-specific feature attribution patterns across effector classes. B: Global feature effects summarized by SHAP values. C: Relative contributions of sequence and structural modalities across different classes.

The global SHAP summary plot (Fig 5B) further illustrates the relationship between feature values and their effects on model output. Several influential sequence features, such as esm_f117 and esm_f43, span both negative and positive SHAP-value regions, indicating that their effects on model predictions vary across samples and feature contexts. Structural features, including struct_f68, struct_f30, struct_f61, and struct_f88, also show non-negligible SHAP effects, suggesting that structural information provides additional decision-relevant signals beyond those captured by the sequence representation. Consistent with these observations, the modality-level analysis in Fig 5C shows that sequence features account for the majority of the overall SHAP contribution across all classes, whereas structural features provide 18.4%–29.3% of the total contribution.

To further examine class-specific feature utilization, we generated separate SHAP beeswarm plots for the six output classes (S4 Fig). Although ESM-derived features dominated the top-ranked features across all classes, their importance profiles varied substantially among classes. Structural features were particularly prominent for T1SE and T6SE, accounting for 6 of the top 15 features in each class, compared with 2–4 in the other classes. This pattern was consistent with the modality-level analysis in Fig 5C, where structural features contributed 29.3% and 27.4% of the total SHAP importance for T1SE and T6SE, respectively. These results indicate that GeoEPred employs distinct multimodal feature-utilization patterns across effector classes, with structural information providing especially important complementary signals for T1SE and T6SE prediction.

In summary, GeoEPred exhibits a clear division of labor between the two modalities: sequence representations provide the predominant discriminative signal, whereas structural information contributes complementary spatial cues that refine class-specific predictions. Together, these results support the complementary nature of sequence and structural representations in the multimodal prediction framework.

### Comparison with baseline methods

To comprehensively evaluate the architectural efficacy of GeoEPred, we performed a systematic benchmark against 11 baseline models under a uniform input configuration. These baselines encompass seven conventional machine learning algorithms (SVM, Random Forest, Gradient Boosting, KNN, Naive Bayes, Decision Tree, and LDA) and four representative deep learning architectures (MLP, BiLSTM-Attention, GRU, and a Vanilla Transformer).

As shown in Table 5, GeoEPred achieved overall superior performance in five-fold cross-validation compared with SVM, the best-performing conventional machine-learning model, and BiLSTM-Attention, the best-performing deep-learning model. Although SVM achieved an accuracy comparable to that of GeoEPred during cross-validation, GeoEPred demonstrated stronger generalization performance on the independent test set, with improvements of 1.5% in accuracy (95% CI: 0.003–0.031, *p* = 0.024) and 3.0% in AUPRC (95% CI: 0.020–0.058, *p* = 0.002) relative to SVM (S9 Table). GeoEPred also achieved a higher AUPRC than BiLSTM-Attention, with an improvement of 3.0% (95% CI: 0.018–0.060, *p* = 0.003). Collectively, these results indicate that GeoEPred can effectively learn discriminative feature representations while exhibiting more robust generalization to independent unseen data, particularly providing improved predictive accuracy and reliability under class-imbalanced conditions.

**Table 5:** Comparison of the proposed method with baseline methods.

Method	ACC		F1		AUPRC	
	CV	Ind	CV	Ind	CV	Ind
*Traditional Machine Learning*						
SVM	0.892	0.908	0.854	0.878	0.882	0.885
Random Forest	0.882	0.896	0.786	0.806	0.825	0.831
Gradient Boosting	0.865	0.869	0.770	0.749	0.805	0.794
KNN	0.857	0.831	0.809	0.804	0.828	0.821
Naive Bayes	0.804	0.815	0.744	0.757	0.768	0.778
Decision Tree	0.691	0.692	0.603	0.611	0.642	0.642
LDA	0.578	0.619	0.611	0.641	0.602	0.632
*Deep Learning Method*						
BiLSTM_Attention	0.862	0.900	0.852	0.864	0.879	0.885
MLP	0.875	0.900	0.856	0.850	0.880	0.871
GRU	0.884	0.900	0.848	0.860	0.883	0.886
Vanilla_Transformer	0.866	0.881	0.854	0.852	0.871	0.891
*Our method*						
GeoEPred	**0.899**	**0.923**	**0.880**	**0.884**	**0.907**	**0.915**

### Comparison with state-of-the-art models

To rigorously evaluate GeoEPred’s efficacy in identifying Gram-negative secretion effectors (T1SE–T4SE and T6SE), we benchmarked it against three multi-class frameworks—MoCETSE [14], DeepSecE [13], BastionX [42], and six specialized binary classifiers using datasets S3–S6. Under uniform testing conditions, predictions for baseline methods were retrieved from their official web servers. Specifically, binary models were assessed on their respective target datasets (S3 for T1SEstacker [43]; S4 for Bastion3 [7] and T3SEpp [44]; S5 for Bastion4 [28], T4SEfinder [26], and T4SEpp [30]; S6 for Bastion6 [8]), whereas all multi-class models were trained on the DeepSecE dataset and tested on the benchmark datasets strictly following the configurations reported in the original studies. Performance was quantified using ACC, REC, PR, F1-score, and MCC, with tool access details provided in S2 Table.

From the overall results, GeoEPred achieved predictive performance comparable to or better than that of current state-of-the-art methods, with a more evident advantage in the T6SE prediction task (Fig 6A–C, S10 Table). As shown in S11 Table, compared with MoCETSE, the second-best-performing method, GeoEPred achieved better performance across most evaluation metrics, particularly improving AUPRC, which is more suitable for class-imbalanced settings, by 3.62% (*p* = 0.042). Further analysis of the T6SE class (S12 Table) showed that GeoEPred exhibited strong discriminative ability. Both threshold-independent metrics, AUC and AUPRC, were significantly higher than those of DeepSecE (AUC: 95% CI: 0.0012–0.0233, *p* = 0.020; AUPRC: 95% CI: 0.0118–0.3631, *p* = 0.016). Compared with MoCETSE (S13 Table), GeoEPred also showed moderate improvements in both AUC and AUPRC (AUC: Δ=+0.0098, 95% CI: 0.0000–0.0279, *p* = 0.086; AUPRC: Δ=+0.0574, 95% CI: 0.0000–0.1457, *p* = 0.076). Although these differences were only marginally significant, the confusion matrix showed that GeoEPred correctly identified all T6SE samples in the test set (Fig 6D), further demonstrating its good predictive performance in T6SE identification.

**Fig 6:**
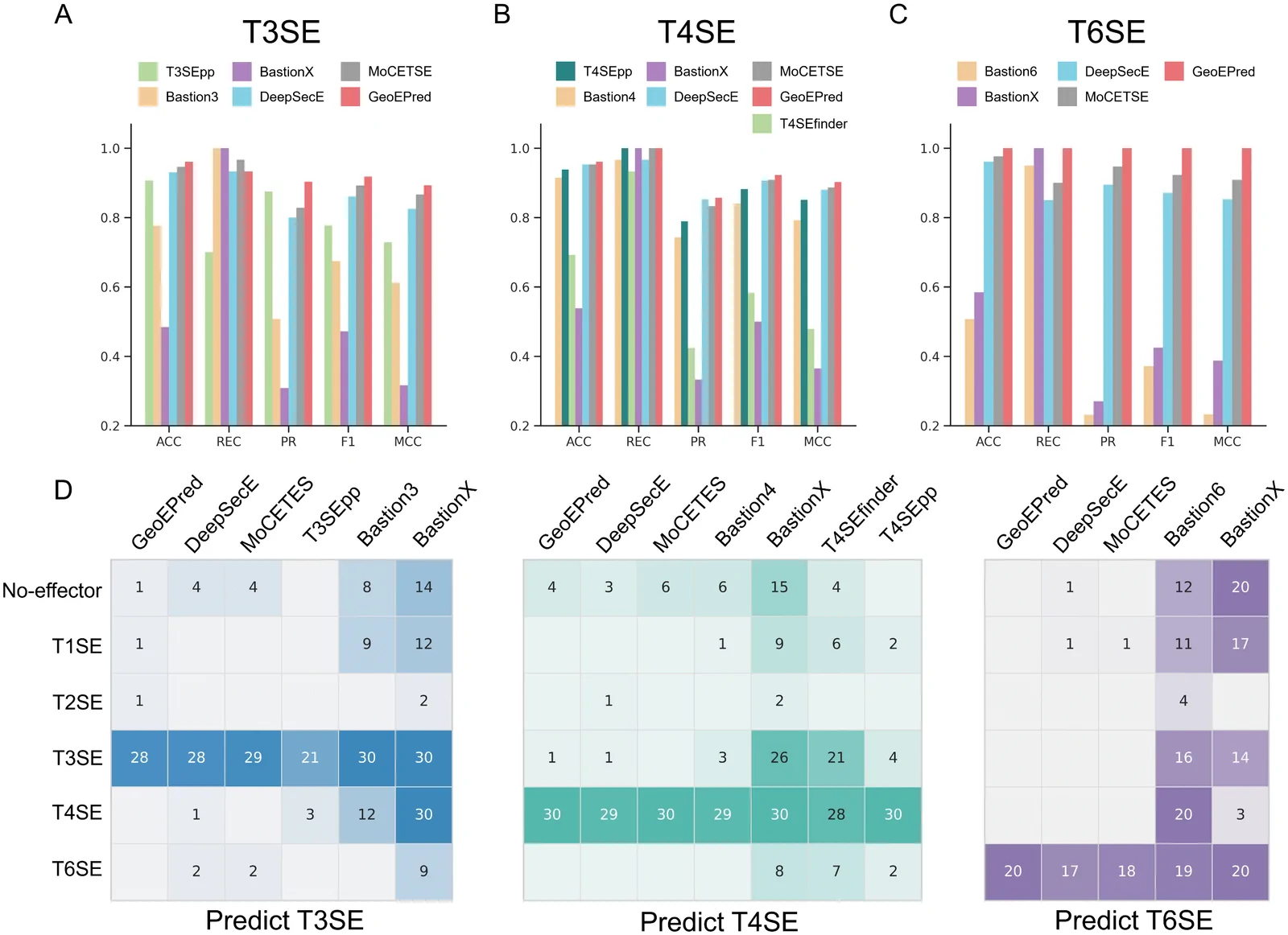
Performance evaluation of GeoEPred in comparison with mainstream models. A–C: Performance comparison of GeoEPred with mainstream prediction models on T3SE **(A)**, T4SE **(B)**, and T6SE (C) prediction tasks. D: Comparison of true-positive and false-positive predictions from different models on the independent test set (Dataset S2), which contains 150 non-effectors, 20 T1SEs, 10 T2SEs, 30 T3SEs, 30 T4SEs, and 20 T6SEs. The heatmaps illustrate the distribution of predicted samples across various effector protein types.

Although some binary classification methods may achieve higher sensitivity on specific single tasks, they often exhibit a higher risk of cross-class false positives when applied to multi-class independent test sets (Fig 6D). For example, Bastion3, a well-performing binary classifier for T3SEs, correctly identified all T3SE samples in the test set but also produced a number of false-positive predictions for other effector types. Similarly, T1SEstacker performed well in T1SE identification but also showed cross-class misclassification by incorrectly assigning some non-T1SE proteins to the T1SE class (S2 Fig). From a practical perspective, because a single bacterial genome typically encodes multiple types of secretion effectors, a multi-class model with more balanced performance across different classes may be more useful for genome-scale applications. In this regard, GeoEPred showed stable and balanced performance in multi-class identification, making it suitable for prediction in complex genomic backgrounds.

To ensure a fair comparison, we trained and evaluated GeoEPred using the same training datasets as those reported for the corresponding binary classification models. Across seven benchmark datasets covering T3SE (two datasets), T4SE (three datasets), and T6SE (two datasets), GeoEPred achieved performance comparable to that of the existing SOTA models on six of the seven test sets (Table 6). To further investigate the performance differences observed across datasets, we analyzed the corresponding pLDDT scores for each dataset (S14 Table). The results showed that GeoEPred tended to perform better on datasets with higher overall structural prediction quality, suggesting that its predictive performance may, to some extent, be influenced by the quality of the predicted input structures.

**Table 6:** This study compares GeoEPred with state-of-the-art (SOTA) binary classification models for T3SE, T4SE, and T6SE prediction using the same training and evaluation datasets reported in the corresponding studies.

Compared model	Task	Method	Performance metrics				
			REC	PR	ACC	F1	MCC
Bastion3	T3SE	GeoEPred	0.941	**0.976**	**0.959**	0.958	**0.918**
		Bastion3	**0.954**	0.963	0.958	0.958	0.917
T3SEpp	T3SE	GeoEPred	**0.995**	**0.798**	**0.858**	**0.886**	**0.735**
		T3SEpp	0.929	0.796	0.829	0.857	0.659
Bastion4	T4SE	GeoEPred	**1.000**	**0.880**	**0.976**	0.935	**0.924**
		Bastion4	0.967	0.942	0.953	**0.954**	0.907
T4SEpp	T4SE	GeoEPred	0.870	0.963	0.981	0.913	0.905
		T4SEpp	**0.900**	**1.000**	**0.988**	**0.947**	**0.942**
BastionX	T4SE	GeoEPred	0.923	**0.978**	**0.951**	**0.949**	**0.904**
		BastionX	**1.000**	0.882	0.933	0.938	0.874
Bastion6	T6SE	GeoEPred	0.950	**0.968**	**0.992**	**0.958**	**0.954**
		Bastion6	**1.000**	0.465	0.895	0.635	0.642
BastionX	T6SE	GeoEPred	0.979	**0.976**	**0.978**	**0.978**	**0.955**
		BastionX	**1.000**	0.935	0.975	0.967	0.933

### Influence of ESMFold structural confidence on model performance

Because our model uses ESMFold-predicted structures as inputs to the structural branch, we further investigated the association between predicted structural confidence and effector protein classification performance.

Based on the mean protein-level pLDDT score, the independent test set was divided using fixed thresholds into four confidence groups: *Very low* (pLDDT<50), *Low* (50≤pLDDT<70), *Confident* (70≤pLDDT<90), and *Very high* (pLDDT≥90). As shown in Fig 7A, the corresponding accuracies were 81.82%, 87.50%, 88.89%, and 96.47%, respectively, exhibiting an overall increase with structural confidence.

**Fig 7:**
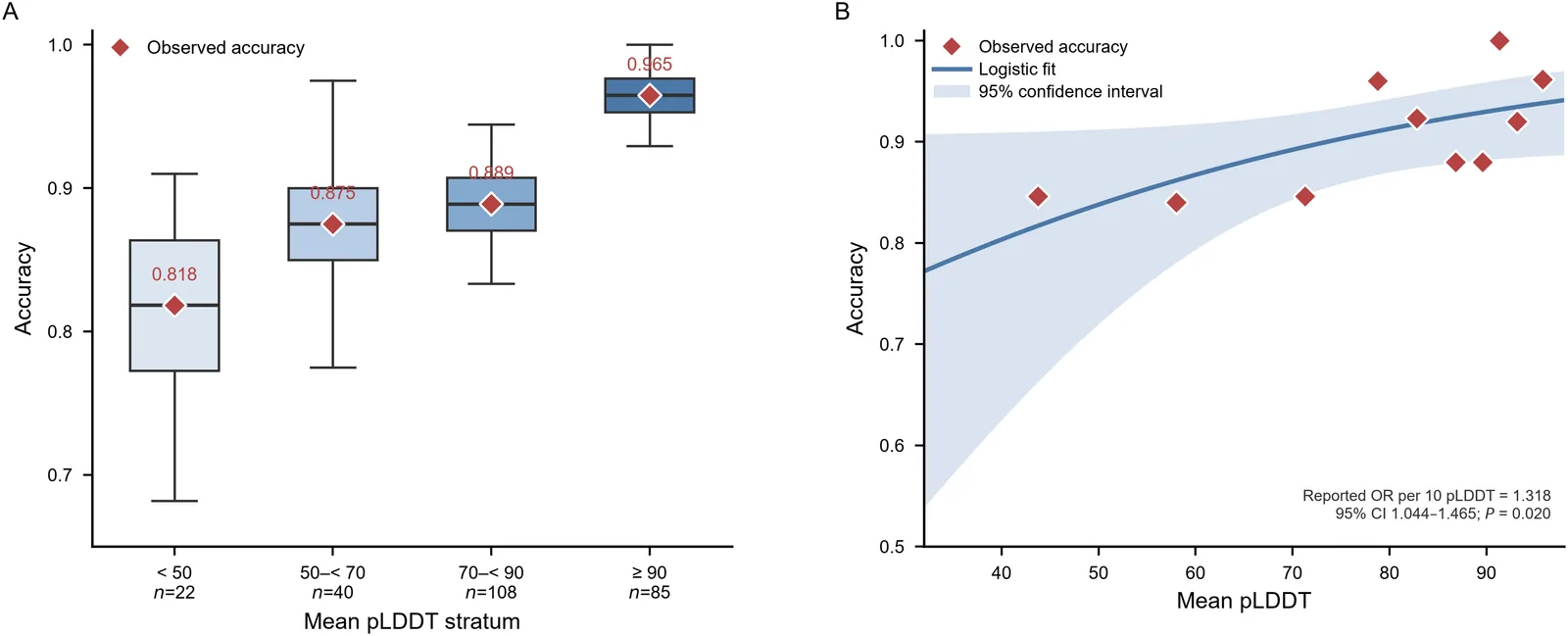
Association between ESMFold-predicted structural confidence and model classification performance. A: Classification accuracy across four structural-confidence groups defined by the mean protein-level pLDDT: *Very low* (pLDDT<50), *Low* (50≤pLDDT<70), *Confident* (70≤pLDDT<90), and *Very high* (pLDDT≥90). Error bars indicate 95% confidence intervals. B: Continuous association between mean pLDDT and classification accuracy. The 260 test proteins were divided into 10 equal-sized bins, each containing 26 proteins. Black points represent the mean pLDDT and observed accuracy within each bin. The red dashed curve and gray shaded 95% confidence band were estimated by logistic regression using the protein-level correct/incorrect outcomes of all 260 test samples rather than the binned observations. After adjustment for the true class label and aligned protein length, each 10-point increase in mean pLDDT was associated with higher odds of correct classification (OR = 1.798, 95% CI: 1.242–2.602, *p* = 0.002).

To quantify this association on a continuous scale, the 260 test proteins were further divided into 10 approximately equal-sized pLDDT bins, each containing 26 proteins. The points in Fig 7B represent the mean pLDDT and observed accuracy within each bin, whereas the fitted curve and its 95% confidence band were estimated from protein-level binary correctness outcomes for all 260 samples using logistic regression. The probability of correct classification increased with mean pLDDT, although the wider confidence band in the low-pLDDT region indicated greater estimation uncertainty. After adjusting for the true class label and aligned protein length, each 10-point increase in mean pLDDT was associated with a 79.8% increase in the odds of correct classification (OR = 1.798, 95% CI: 1.242–2.602, *p* = 0.002). Consistently, correctly classified proteins had a higher mean pLDDT than incorrectly classified proteins (79.88 vs. 71.54; Mann–Whitney *U* = 3522.0, *p* = 0.029, BH–FDR-adjusted *q* = 0.044, Cliff’s δ=0.271). Together, these results demonstrate a statistically significant positive association between predicted structural confidence and classification performance.

As a supplementary structural validation, we further compared ESMFold-predicted structures with experimentally resolved PDB structures for the subset of 29 proteins meeting stringent sequence-matching and structural-coverage criteria (S5 Fig). Mean pLDDT was strongly positively correlated with TM-score (Spearman’s ρ=0.922) and negatively correlated with RMSD (ρ=−0.905), providing preliminary structure-level support for the use of pLDDT as an indicator of structural reliability (S5 Fig). Given the limited number of matched structures, this analysis was considered supportive rather than definitive evidence.

Despite this association, the model retained good predictive performance for lower-confidence structures: the accuracy was 81.82% in the *Very low* group and approximately 84% in the lowest-pLDDT quantile bin. Together with the performance degradation observed after removing the sequence branch in the ablation study, these findings support the complementary roles of sequence and structural information. In particular, the sequence branch may provide additional discriminative cues when structural confidence is limited, thereby improving the model’s robustness to uncertainty in predicted structures.

### Case studies

Genome-wide prediction of secreted effector proteins in Gram-negative bacteria is a crucial approach for investigating their pathogenicity, distribution patterns, and evolutionary dynamics [45]. Compared with labor-intensive and time-consuming experimental methods, bioinformatics approaches enable more efficient screening of potential effector proteins, providing a practical solution for large-scale studies of their distribution, evolutionary trajectories, and pathogenic potential [10]. To assess the practical applicability of GeoEPred, we developed a computational pipeline that integrates MacSyFinder’s specialized secretion system models [46] with the GeoEPred framework. This integrated framework predicts secretion systems and their associated secreted proteins directly from bacterial genome data, thereby enabling genome-scale annotation of potential secretion substrates.

We applied GeoEPred to perform genome-wide screening of secreted proteins from 5,572 protein-coding sequences of *Pseudomonas aeruginosa PAO1* (NCBI accession: NC_002516.2), successfully identifying gene clusters corresponding to Type III and Type VI secretion systems. To further evaluate the capability of GeoEPred as a genome-wide prediction model, we specifically analyzed the predicted Type VI secreted effectors in *Pseudomonas aeruginosa PAO1*. GeoEPred correctly identified the majority of experimentally validated effectors (28 out of 30), achieving a recall of 93.3%. Meanwhile, high-confidence candidate proteins identified by GeoEPred, specifically T1SE, T3SE, and T6SE (S6 Fig), showed strong genomic proximity to their cognate secretion system loci detected by MacSyFinder (Fig 8), suggesting that these candidate effectors may be functionally and genomically associated with their corresponding secretion machinery. Permutation testing confirmed significant spatial co-localization near cognate gene clusters for T1SE, T3SE, and T6SE, as shown in S15 Table (*p* < 0.0001). This observation is consistent with previous reports showing that some experimentally validated effector proteins are frequently located near secretion system gene clusters or pathogenicity islands [47].

**Fig 8:**
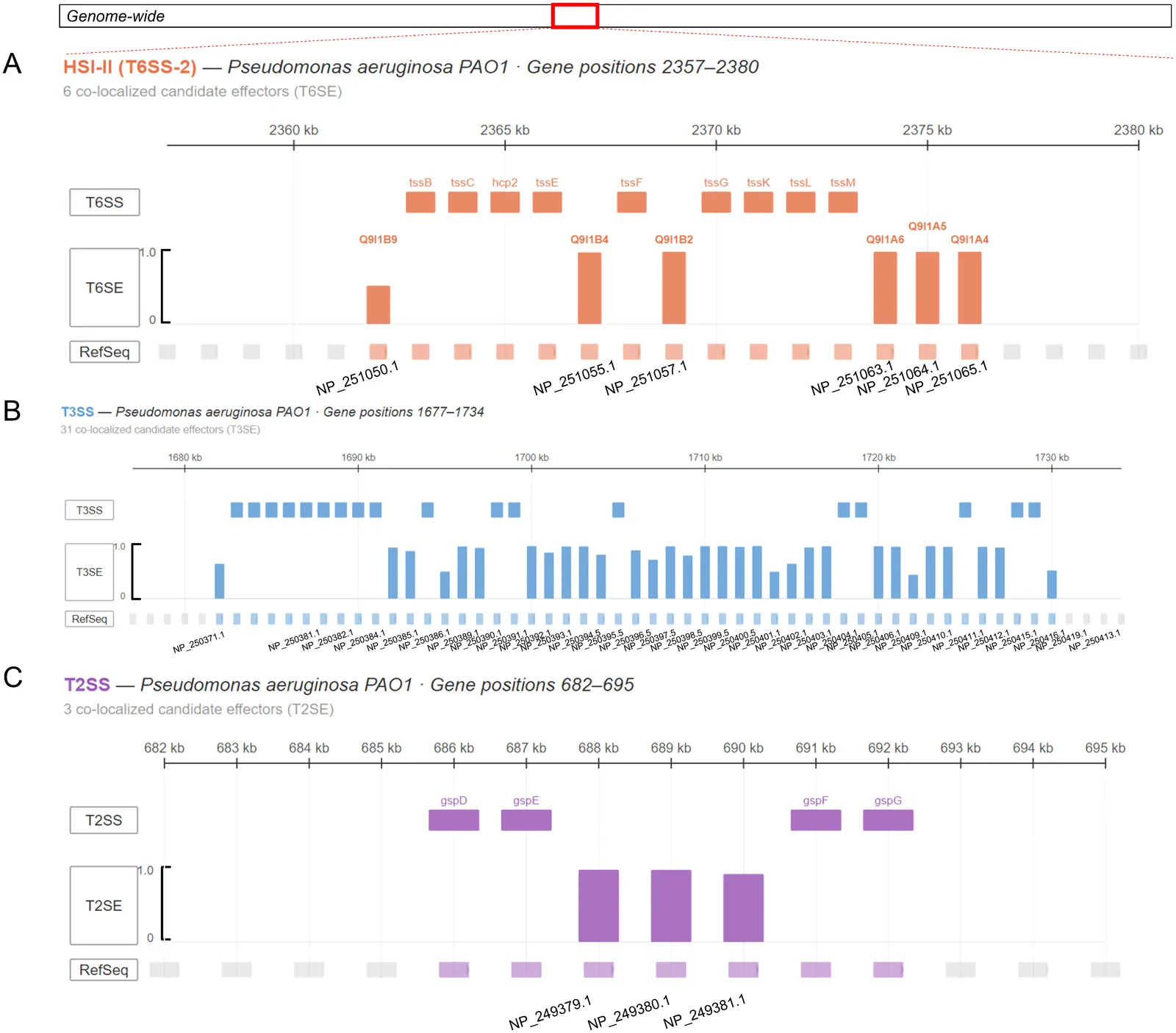
Genome-wide predictions indicate that effector proteins are distributed near the secretory system.

To further assess the biological relevance of the newly predicted candidates, we performed functional characterization using Gene Ontology (GO) enrichment analysis and protein domain annotation. Enrichment significance was assessed using a hypergeometric test followed by Benjamini–Hochberg correction for multiple testing. In *P. aeruginosa* PAO1, GO enrichment analysis revealed significant overrepresentation of secretion-associated functions, including protein secretion and Type VI secretion system complexes. Pfam domain analysis further identified enrichment of several effector-associated domains, including Sel1-like repeats, ADP-ribosyltransferase domains, and YopE-like GAP domains. Sel1-like repeats have been implicated in *Legionella* host-cell interactions and virulence-associated processes [48]. ADP-ribosyltransferase domains are characteristic of several bacterial toxins and secreted effectors involved in host cellular modulation, such as the T3SS effector ExoS from *Pseudomonas aeruginosa* [49]. Similarly, the YopE GAP domain represents a well-characterized effector module that manipulates host Rho GTPase signaling [50]. These observations provide additional functional context supporting the potential biological relevance of the predicted candidates.

In addition, we further evaluated the model on two other Gram-negative bacterial genomes: *Salmonella enterica subsp. enterica serovar Typhimurium str. LT2* (NC_003197.2) and *Legionella pneumophila subsp. pneumophila str. Philadelphia 1* (NC_002942.5). The predicted results were compared against experimentally validated data. GeoEPred reliably identified a large proportion of experimentally confirmed T3SE and T4SE proteins, achieving recall rates of 94.78% (36 out of 38) and 91.2% (280 out of 307), respectively. These results demonstrate that GeoEPred performs robustly across different biological contexts and maintains reliable performance for large-scale genome-wide annotation tasks.

Functional characterization of predicted candidates from these genomes further supported their biological relevance. In *Salmonella enterica* serovar Typhimurium LT2, GO enrichment analysis revealed significant associations with secretion-related and extracellular biological processes, including adhesion, biofilm formation, and extracellular enzymatic activities (Fig 9). In *Legionella pneumophila* Philadelphia-1, where GO annotation coverage was insufficient for reliable enrichment analysis, InterPro and Pfam domain analyses identified several enriched protein domains, including DUF4917, DUF8197, and Sel1-like repeats. Sel1-like repeat-containing proteins have previously been implicated in *Legionella* virulence and host-cell interaction processes, suggesting that these domain architectures may represent potential molecular features associated with host adaptation [48]. In addition, among the predicted T6SE candidates, enrichment of phage tail- and baseplate-associated domains was observed, which is consistent with the reported evolutionary relationship between the Type VI secretion system and contractile bacteriophage tail structures [51]. These findings suggest that the GeoEPred-predicted candidates exhibit distinct functional and structural characteristics associated with bacterial secretion and host interaction. Detailed functional enrichment and domain annotation results are provided in S7 Fig and S16 Table.

**Fig 9:**
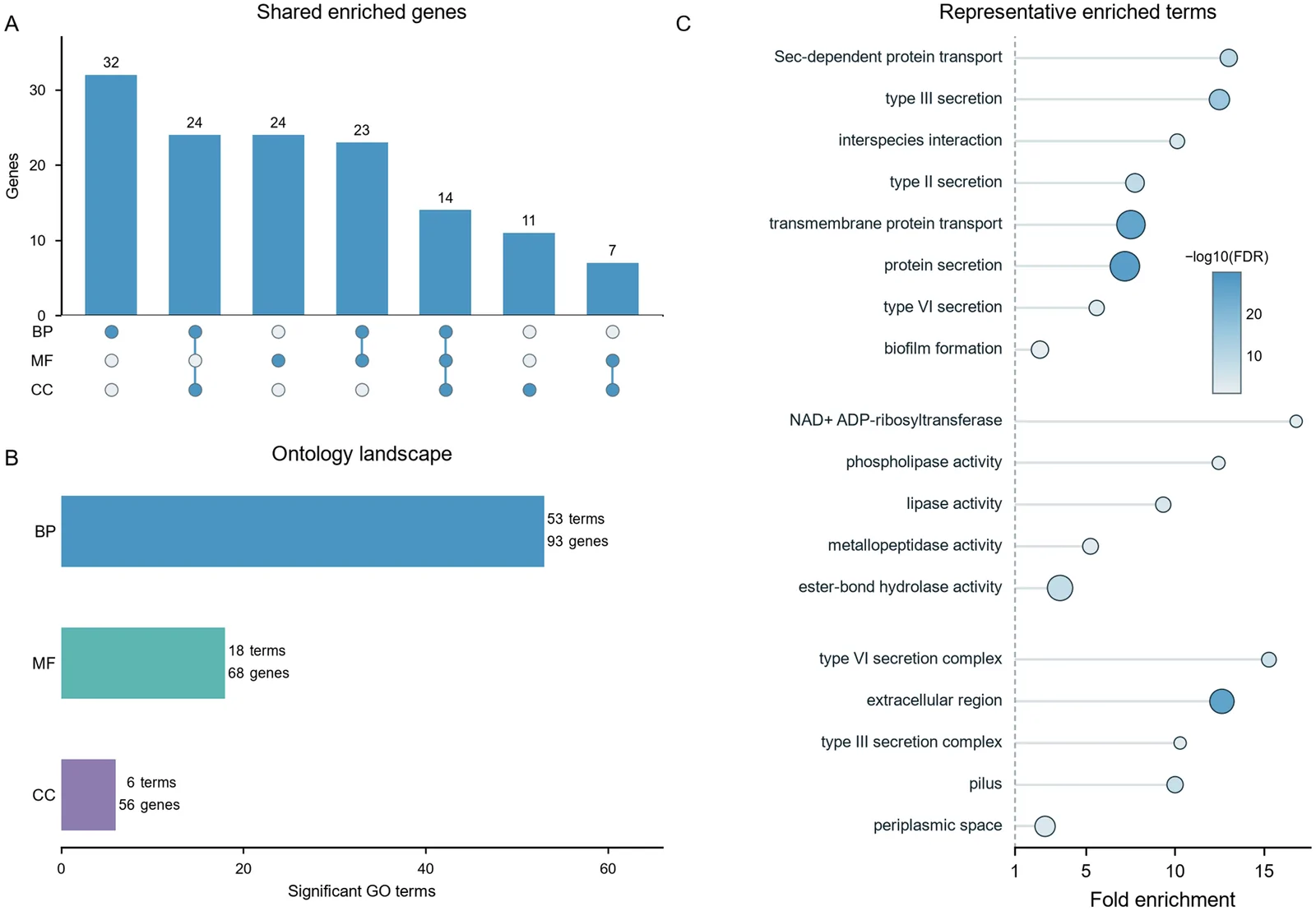
Gene Ontology (GO) functional enrichment analysis of predicted candidate effectors in Pseudomonas aeruginosa PAO1. **A:** The bar plot displays the number of genes shared among different combinations of Biological Process (BP), Molecular Function (MF), and Cellular Component (CC), as indicated by the connected dot matrix below. **B:** Ontology landscape. Overview of the significant GO terms and associated gene counts categorized by BP (blue), MF (teal), and CC (purple). **C:** Representative enriched terms. Lollipop charts illustrating representative enriched terms for BP, MF, and CC, respectively. The x-axis represents the fold enrichment, and the bubble size and color intensity correspond to the statistical significance scaled as $-\log_{10}(p_{adjust})$.

Finally, to evaluate the computational efficiency of GeoEPred for genome-scale applications, we retrained the model on a single NVIDIA RTX 4090 GPU and measured its runtime on protein subsets of different sizes (S8 Fig). Training required approximately 0.38 h for a single fold and approximately 1.44 h for the complete five-fold training, with a peak GPU memory usage of 23.86 GB. During inference, using a batch size of 64, GeoEPred processed proteins at an average rate of 153.38 proteins/s (6.52 ms/protein), with a peak GPU memory usage of 11.48 GB. GeoEPred maintained relatively stable computational performance across different dataset sizes, requiring approximately 6.52 s to process a subset of 1,000 proteins and approximately 36.3 s to process all 5,572 proteins in *Pseudomonas aeruginosa* PAO1. These results indicate that GeoEPred can be trained and deployed on a single high-end GPU and provides practical computational efficiency for bacterial genome-scale screening.

### Biological interpretability

To interpret the biological signals learned by GeoEPred, we visualized the prediction results using sequence significance maps and PyMOL [52], allowing us to examine the model’s decision patterns from both residue-level motif information and three-dimensional structural conformations. At the residue level, the feature projection network refined contextual information to identify key amino acid segments associated with effector secretion and delivery, particularly characteristic signal regions at the N- and C-termini. Complementarily, at the structural level, the GVP module extracted spatial residue proximity and local geometric orientation from ESMFold-predicted structures, enabling the model to capture three-dimensional functional cues that are difficult to infer from linear sequences alone.

For the T3SS effector SlrP from *Salmonella enterica* serovar Typhimurium, we quantitatively evaluated the correspondence between residue-level attribution patterns and experimentally characterized functional regions. The N-terminal region of SlrP (residues 1–50) has been experimentally associated with the T3SS translocation signal [53]. Among the top 5% highest-attribution residues identified by the sequence branch, 16 residues overlapped with this experimentally characterized region, corresponding to a 6.44-fold enrichment relative to the sequence-wide expectation (Fig 10D). This enrichment was supported by circular shift permutation testing (*p* = 0.0030, BH *q* = 0.0060) and hypergeometric enrichment analysis ($p=7.38\times 10^{-11}$, BH $q=1.48\times 10^{-10}$). These results indicate that sequence-derived attribution patterns preferentially correspond to the experimentally characterized T3SS translocation region of SlrP. In contrast, the structural branch exhibited an attribution pattern associated with the C-terminal E2 ubiquitin-conjugating enzyme interaction interface (residues 751–765), which mediates host interaction in NEL-family effectors [54,55]. Two residues within this region were included among the top 5% structural-attribution residues, corresponding to a 2.68-fold enrichment (Fig 10A). Although the enrichment did not reach statistical significance in permutation testing (*p* = 0.0830, BH *q* = 0.0830) or hypergeometric analysis (*p* = 0.1681, BH *q* = 0.1681), the consistent enrichment trend together with the structural localization pattern suggests an association between structural attribution and the experimentally characterized host-interaction interface of SlrP. Together, these observations indicate that the sequence and structural branches provide complementary residue-level attribution patterns corresponding to distinct functional regions of SlrP.

**Fig 10:**
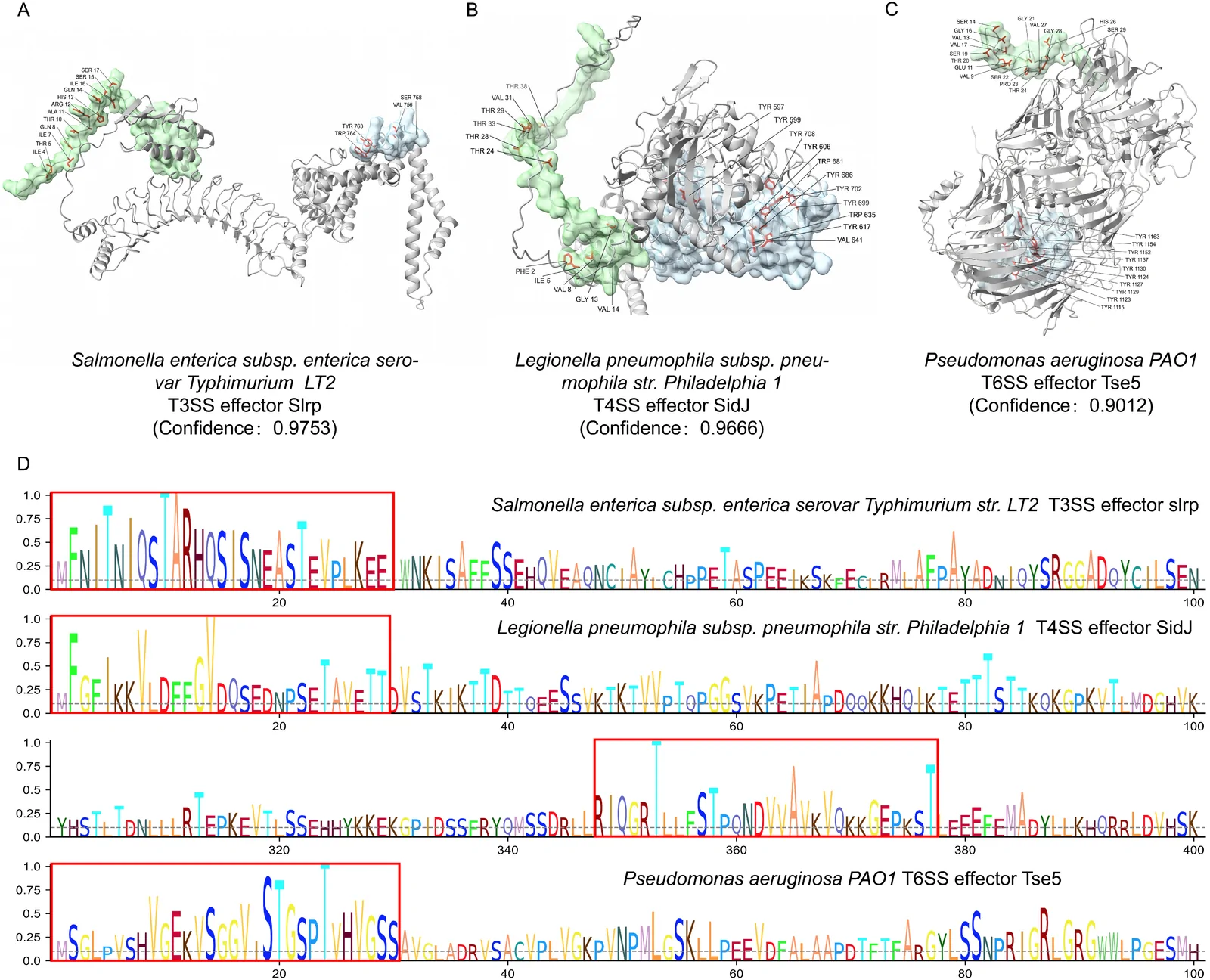
Structural and sequence-level interpretation of model attention for representative effector proteins. **A–C:** Visualization of the weights of E3 generalized enzyme SlrP **(A)**, calmodulin-dependent gamma-glutamyltransferase SidJ **(B)**, and toxin protein Tse5 **(C)**. **D:** Visualization of the N-terminal and C-terminal sequences of the three enzymes, showing the amino acid preferences in their secretion patterns.

For the T4SS effector SidJ from *Legionella pneumophila*, the sequence branch showed enrichment within regions associated with secretion and catalytic activity. Within the intrinsically disordered N-terminal region of SidJ (residues 1–50), the top 5% sequence-attribution residues showed a 4.37-fold enrichment relative to the sequence-wide expectation [56]. This overlap was supported by hypergeometric enrichment analysis ($p=1.32\times 10^{-5}$, BH $q=5.27\times 10^{-5}$), while the circular shift permutation test indicated a consistent enrichment trend (*p* = 0.0334, BH *q* = 0.1336). Similarly, the conserved catalytic motif region of SidJ (residues 348–380) showed enrichment among sequence-attributed residues (Fig 10D). At the top 10% attribution threshold, 15 residues overlapped with this experimentally characterized catalytic region, corresponding to a 4.56-fold enrichment. This overlap was supported by hypergeometric analysis ($p=7.00\times 10^{-8}$, BH $q=4.80\times 10^{-7}$) and circular shift permutation testing (*p* = 0.0250, BH *q* = 0.1336). These results suggest that sequence attribution patterns are associated with sequence features underlying SidJ secretion and catalytic activity. The structural branch showed correspondence with the experimentally characterized calmodulin (CaM)-binding interface (residues 597–708), which is required for activation of SidJ glutamylase activity [57,58]. Among the top 5% structural-attribution residues, 11 residues overlapped with this interface, corresponding to a 1.95-fold enrichment (Fig 10B). This enrichment was supported by circular shift permutation testing ($p=1.00\times 10^{-4}$, BH *q* = 0.0012) and hypergeometric enrichment analysis (*p* = 0.0179, BH *q* = 0.0430). These results support an association between structural attribution patterns and the experimentally characterized host-factor interaction interface of SidJ [57,59].

Regarding the *Pseudomonas aeruginosa PAO1* T6SS effector Tse5, within the N-terminal Hcp interaction region (residues 1–35), 16 residues overlapped with the top 5% sequence-attribution residues, corresponding to a 9.12-fold enrichment relative to the sequence-wide expectation (Fig 10D). This overlap showed a consistent enrichment pattern under circular shift permutation analysis (*p* = 0.0219, BH *q* = 0.0873), with additional support from hypergeometric enrichment analysis ($p=4.81\times 10^{-13}$, BH $q=4.33\times 10^{-12}$). These results indicate that sequence attribution patterns preferentially correspond to the experimentally characterized Hcp interaction region involved in Tse5 recruitment and T6SS-mediated secretion [60]. The structural branch exhibited an attribution pattern associated with the three-dimensional functional interface of Tse5. Within the experimentally characterized Rhs plug/gateway region (residues 1115–1163), 10 residues overlapped with the top 5% structural-attribution residues, corresponding to a 4.07-fold enrichment relative to the sequence-wide expectation (Fig 10C). This enrichment was supported by circular shift permutation analysis (*p* = 0.0011, BH *q* = 0.0099) and hypergeometric enrichment analysis ($p=8.72\times 10^{-5}$, BH $q=3.92\times 10^{-4}$). These results support the correspondence between structural attribution patterns and the experimentally characterized Rhs assembly interface involved in toxin processing and release [60].

Together, these analyses demonstrate that sequence and structural branches exhibit distinct residue-level attribution patterns associated with experimentally characterized functional regions. The sequence branch primarily corresponds to linear sequence features involved in secretion and catalytic functions, whereas the structural branch provides complementary information associated with spatially organized interaction interfaces and toxin-associated conformational regions.

## Discussion

Deep learning provides an efficient computational approach for predicting effector proteins in Gram-negative bacteria and offers new opportunities for investigating bacterial virulence mechanisms and supporting antibacterial drug design [10,45,61]. In this study, we propose GeoEPred, an end-to-end effector protein prediction model that jointly models linear sequence semantics and three-dimensional structural information. Unlike existing methods that mainly rely on sequence representations, GeoEPred employs a geometric vector perceptron to encode three-dimensional protein conformations, thereby complementing spatial relationships and geometric environments that are difficult to represent explicitly from sequence information alone. Benchmark results show that GeoEPred performs relatively well for the T6SE class and achieves performance comparable to or better than existing methods for the other classes (Fig 6), indicating that joint modeling of sequence and structural information can improve effector protein classification.

Modality ablation analysis further reveals the different roles of the two modalities in GeoEPred (Table 2). The refined sequence-semantic representation already provides relatively strong overall discriminative capability, whereas the UMAP results show that several effector protein classes still exhibit local overlap in the low-dimensional representation space (Fig 3). After structural information is incorporated, class separation and overall classification performance are further improved, suggesting that the structural modality complements sequence-semantic representations by providing residue-level spatial relationships, local geometric environments, and conformational context. Notably, the model shows relatively limited performance when structural information is used alone, indicating that the structural modality mainly plays a complementary role in this task. SHAP analysis shows that sequence-derived features account for 74.53% of the overall feature attribution, whereas structural features account for 25.47%, indicating that sequence information constitutes the primary basis for discrimination (Fig 5). Nevertheless, several structural features show relatively high importance across multiple classes, suggesting that structural information can provide important spatial and conformational information for specific classes. Therefore, the main value of the structural modality lies in complementing three-dimensional relationships that are difficult to describe directly using sequence representations and in forming a more complete protein representation together with sequence semantics.

Structural prediction quality may further affect the discriminative capability of the structural modality. After stratifying the samples according to the pLDDT scores of ESMFold-predicted structures, we observed that model performance generally improved with increasing structural confidence, suggesting that more reliable structures can provide more informative geometric representations (Fig 7). At the same time, GeoEPred maintained relatively stable performance for samples with lower structural confidence, indicating that the sequence branch can provide additional discriminative information when structural information becomes less reliable. We further constructed an alternative structural representation using the 3Di structural alphabet, and the structure-only model still showed relatively limited performance (S5 Table). This observation suggests that the limited performance may not be entirely attributable to a specific structural encoding scheme, but may also be related to the discriminative characteristics of structural information in effector protein classification. This phenomenon may reflect the complex relationship between protein structure and function. Proteins with different functions may share similar global folds or local structural patterns, whereas functional specificity also depends on key residue identities, local sequence environments, and their spatial organization [62]. Therefore, when structural information is used alone, shared geometric patterns among different classes may reduce class separability. In contrast, joint modeling of sequence and structural information enables the model to utilize residue identity, contextual information, and spatial relationships simultaneously, resulting in a more discriminative representation.

To further determine an appropriate fusion strategy for this task, we compared the feature tokenization strategy used in GeoEPred with conventional feature concatenation and cross-attention. The results show that feature tokenization achieves better performance on most evaluation metrics. This indicates that converting sequence and structural representations into unified feature tokens for joint modeling can help preserve modality-specific information while learning cross-modal associations (Table 4). Therefore, the performance improvement of GeoEPred is not only related to the incorporation of structural information, but also depends on the effective integration of complementary sequence and structural information.

The ROC curves and confusion matrices further show that GeoEPred maintains stable and relatively good predictive performance across different effector protein classes (Fig 2). GeoEPred also maintains good performance in the evaluation involving remotely homologous proteins (S6 Table), indicating that the learned sequence-semantic and structural representations can still provide useful discriminative information when sequence similarity is reduced. These results support the potential of GeoEPred to identify effector proteins beyond closely related sequence similarity.

In addition to predictive performance, GeoEPred provides interpretation evidence from both sequence and structural perspectives that is consistent with known functional regions. The model can identify important N-terminal or C-terminal sequence regions in several effector proteins, as well as structural regions associated with known virulence-related functions (Fig 10). Quantitative attribution analysis further shows that high-contribution residues correspond to several experimentally reported secretion, translocation, or functional regions, while the sequence and structural branches exhibit different attribution patterns (S17 Table). The sequence modality mainly captures functional information encoded in linear sequences, whereas the structural modality complements information associated with three-dimensional spatial organization. Although model attribution cannot replace direct experimental validation, it can provide computational evidence for the subsequent prioritization of key residues and functional regions.

At the genome scale, secreted protein prediction commonly requires the integration of multiple homology-based and feature-driven approaches [45]. In contrast, GeoEPred can directly predict effector proteins associated with different secretion systems based on protein sequence and structural information. We applied GeoEPred to the genomes of three representative Gram-negative pathogenic bacteria. The results show that GeoEPred can identify a large proportion of experimentally validated effector proteins while also screening an additional set of potential candidate proteins (Fig 8, S18 Table). Further GO enrichment and Pfam domain analyses show that these candidate proteins contain several significantly enriched functional terms and domains (S7 Fig, S16 Table), including some domains associated with effector proteins or with functions that remain insufficiently characterized, providing additional support for the biological plausibility of some candidates. Although these analyses cannot directly demonstrate effector functions for individual candidate proteins, they can provide auxiliary evidence for candidate prioritization and support the use of GeoEPred for potential effector discovery and experimental candidate screening.

Despite the predictive performance and potential applicability of GeoEPred, several limitations remain. First, experimentally validated effector proteins are unevenly distributed across different secretion system classes, which may affect model training and performance evaluation for classes with fewer samples. In addition, because of the current availability of data, GeoEPred is mainly applicable to the secretion systems covered in this study and has not yet been extended to other secretion systems or effector proteins associated with Gram-positive bacteria. As more high-quality experimentally validated data become available, the framework can be gradually extended to additional secretion systems and a broader range of bacterial groups.

Second, the structural modality is, to some extent, constrained by structural quality. For proteins that are highly dynamic, intrinsically disordered, or predicted with low structural confidence, the reliability of geometric representations may therefore decrease. Future work may incorporate higher-quality experimentally determined structures, improved structural predictions, and information on conformational dynamics and protein complexes to enhance the representation capability of the structural modality.

Third, the current evaluation primarily supports the sequence-level generalization of GeoEPred rather than strict species-level generalization. Although homologous sequences between the training and independent test sets were strictly controlled, complete species-level separation was not feasible because experimentally validated effectors remain limited and unevenly distributed across species. To preliminarily address this issue, we collected experimentally validated effectors reported in recent years from genomes absent from our current dataset [63–66]. GeoEPred successfully identified 8 T3SEs and all T6SEs in this external evaluation (S18 Table), providing preliminary support for its applicability to previously unseen genomic backgrounds. As more experimentally validated effectors become available, additional external datasets without species overlap with the training data will be incorporated to further assess cross-species generalization. Moreover, because pretrained protein language models are typically trained on large-scale public databases, potential overlap or indirect association with independent test data cannot be completely excluded. Future evaluations using newly available external datasets will therefore provide a more stringent assessment of model generalization.

Finally, although this study supports the biological plausibility of the predicted candidate proteins through predictive performance, model interpretation, functional annotation, and genome-scale analyses, the newly predicted candidates have not yet been directly validated experimentally. Therefore, these proteins should currently be regarded as computationally prioritized candidate effectors. Future work may further validate high-priority candidates and systematically evaluate the applicability of GeoEPred to practical effector protein discovery.

## Supporting information

S1 Appendix Evaluation indicators. (PDF)

S1 Fig Visualization of protein sequence length distributions in the dataset. (PDF)

S2 Fig Performance comparison of GeoEPred and mainstream models in type I (T1SE), type II (T2SE), and non-effector prediction tasks. A–C: Model performance for T1SE, T2SE, and non-effector prediction was evaluated using accuracy (ACC), recall (REC), precision (PR), F1 score (F1), and Matthews correlation coefficient (MCC). D–F: Comparison of true-positive and false-positive predictions for T1SE, T2SE, and non-effectors across different models. The heatmaps show the sample distributions predicted as T1SE, T2SE, and non-effector.
(PDF)

S3 Fig The dataset contains samples with distant homology, including 2 T1SE, 42 T3SE, 34 T4SE, 9 T6SE, and 20 non-effector proteins. (PDF)

S4 Fig Class-stratified SHAP summary beeswarm plot for six target categories. (PDF)

S5 FigComparison of ESMFold-predicted structures with experimentally resolved protein structures.A: Mean pLDDT versus TM-score. B: Mean pLDDT versus RMSD. C: Comparison of TM-scores between low-confidence (pLDDT<70) and high-confidence (pLDDT≥70) predictions. Each point represents one protein.(PDF)

S6 Fig Genome-wide prediction visualization shows that GeoEPred not only successfully identifies T6SEs in *Pseudomonas aeruginosa*, but also predicts other candidate effectors in the genome (A–E). (PDF)

S7 Fig Gene Ontology (GO) functional enrichment analysis of predicted candidate effectors in *Salmonella enterica* serovar Typhimurium LT2. (PDF)

S8 Fig Model inference time scaling with protein dataset sizes. (PDF)

S1 Table Summary of the training, testing, and benchmarking datasets used in the comparative experiments. (PDF)

S2 Table Web server links of the comparative models used in this study. (PDF)

S3 Table Comparison of the performance of GeoEPred on independent test sets based on ESM-1b, ESM-2, and other protein language models. (PDF)

S4 Table GitHub repositories of protein language models evaluated for GeoEPred. (PDF)

S5 Table Performance improvements of the proposed model compared with the single-modal model, with 95% confidence intervals and statistical significance analysis. (PDF)

S6 Table Comparison of predictive performance among different models under remote-homology scenarios. (PDF)

S7 Table Performance gains, 95% confidence intervals, and statistical significance of the full model compared with different ablated model variants. (PDF)

S8 Table Performance improvements, 95% confidence intervals, and statistical significance analysis of the proposed model compared with different fusion strategies. (PDF)

S9 Table Performance gain, 95% confidence intervals, and statistical significance analysis of the proposed model compared to the best traditional machine learning and deep learning baseline methods. (PDF)

S10 Table Performance comparison between GeoEPred and existing popular methods on benchmark datasets. (PDF)

S11 Table Overall performance comparison among GeoEPred, DeepSecE, and MoCETSE. (PDF)

S12 Table Class-wise statistical significance analysis of performance differences between GeoEPred and DeepSecE on the independent test set. (PDF)

S13 Table Class-wise statistical significance analysis of performance differences between GeoEPred and MoCETSE on the independent test set. (PDF)

S14 Table Sample-level pLDDT statistics across different benchmark datasets, ranked by the mean pLDDT of the training set. (PDF)

S15 Table Genomic co-localization analysis of predicted effectors and corresponding secretion system gene clusters in *Pseudomonas aeruginosa* PAO1. (PDF)

S16 Table Pfam domain enrichment analysis across candidate effector proteins in three bacterial genomes. (PDF)

S17 Table Quantitative residue-level attribution enrichment analysis of representative effector proteins using circular shift permutation and hypergeometric tests. (PDF)

S18 Table GeoEPred predictions for recently experimentally validated bacterial effector proteins. (PDF)
