# Supplementary material for: GeoEPred: A multimodal structure-aware geometric deep learning framework for Gram-negative bacterial secreted effector prediction with sequence semantics
Source: PLoS Comput Biol. 2026 Sep 30;22(9):e1014344. doi: 10.1371/journal.pcbi.1014344 (PMC13641719; doi:10.1371/journal.pcbi.1014344)
Supplement: Supplementary file 1 [file pcbi.1014344.s001.pdf]

S1 Appendix. Evaluation indicators

This study employs a multi-metric approach to evaluate the performance of the model across both cross-validation and independent testing. Specifically, we utilize macro-averaged accuracy, F1-score, the Area Under the Receiver Operating Characteristic curve (AUC), and the Matthews Correlation Coefficient (MCC) for comparative analysis. Macro-averaged accuracy is defined as the arithmetic mean of the accuracy across all categories. By assigning equal weight to each class, this metric effectively mitigates bias resulting from class imbalance and provides an objective reflection of the model's classification performance on minority classes.

In the multi-class classification task involving five types of secreted effectors, the ROC curve visually illustrates the model's capability to distinguish between positive and negative samples across various threshold settings. Furthermore, a multi-class confusion matrix provides a granular view of the classification outcomes for each protein category. A higher AUC (approaching 1) signifies superior discriminative power. To ensure a comprehensive performance comparison with existing binary and multi-class methods on benchmark datasets, we adopted several standard metrics, including Accuracy (ACC), Recall (REC), Precision (PR), F1-score (F1), and the Matthews Correlation Coefficient (MCC).

The evaluation metrics are defined as follows:

$$ACC = \frac{TP + TN}{TP + FP + TN + FN} \tag{1}$$

$$REC = \frac{TP}{TP + FN} \tag{2}$$

$$PR = \frac{TP}{TP + FP} \tag{3}$$

$$F1 = 2 \times \frac{PR \times REC}{PR + REC} \tag{4}$$

$$MCC = \frac{TP \times TN - FP \times FN}{\sqrt{(TP + FP)(TP + FN)(TN + FP)(TN + FN)}} \tag{5}$$
