# Supplementary material for: GeoEPred: A multimodal structure-aware geometric deep learning framework for Gram-negative bacterial secreted effector prediction with sequence semantics
Source: PLoS Comput Biol. 2026 Sep 30;22(9):e1014344. doi: 10.1371/journal.pcbi.1014344 (PMC13641719; doi:10.1371/journal.pcbi.1014344)
Supplement: Supplementary file 2 [file pcbi.1014344.s002.pdf]

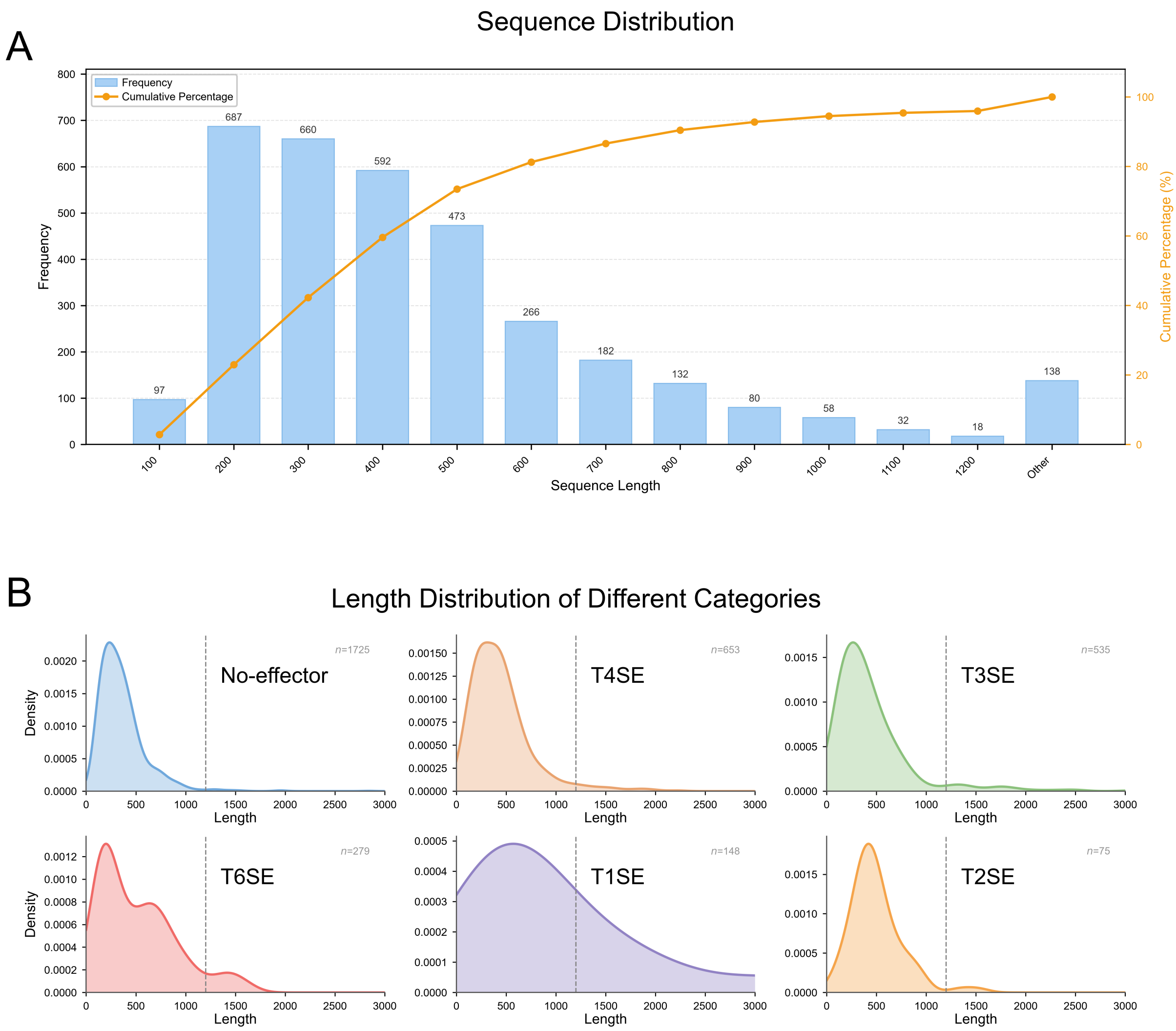

S1 Fig. Visualization of protein sequence length distributions in the dataset. A: Histogram and cumulative distribution of sequence lengths across all samples. The blue bars represent the frequency of sequences in each length bin, while the orange line indicates the cumulative percentage. B: Kernel density estimation plots of sequence lengths for different protein classes: non-effector, T4SE, T3SE, T1SE, T2SE, and T6SE. The vertical dashed line in each panel represents the 1,000-residue cutoff. Sample sizes (n) for each class are indicated in the top-right corner of each plot.
