# Supplementary material for: GeoEPred: A multimodal structure-aware geometric deep learning framework for Gram-negative bacterial secreted effector prediction with sequence semantics
Source: PLoS Comput Biol. 2026 Sep 30;22(9):e1014344. doi: 10.1371/journal.pcbi.1014344 (PMC13641719; doi:10.1371/journal.pcbi.1014344)
Supplement: Supplementary file 3 [file pcbi.1014344.s003.pdf]

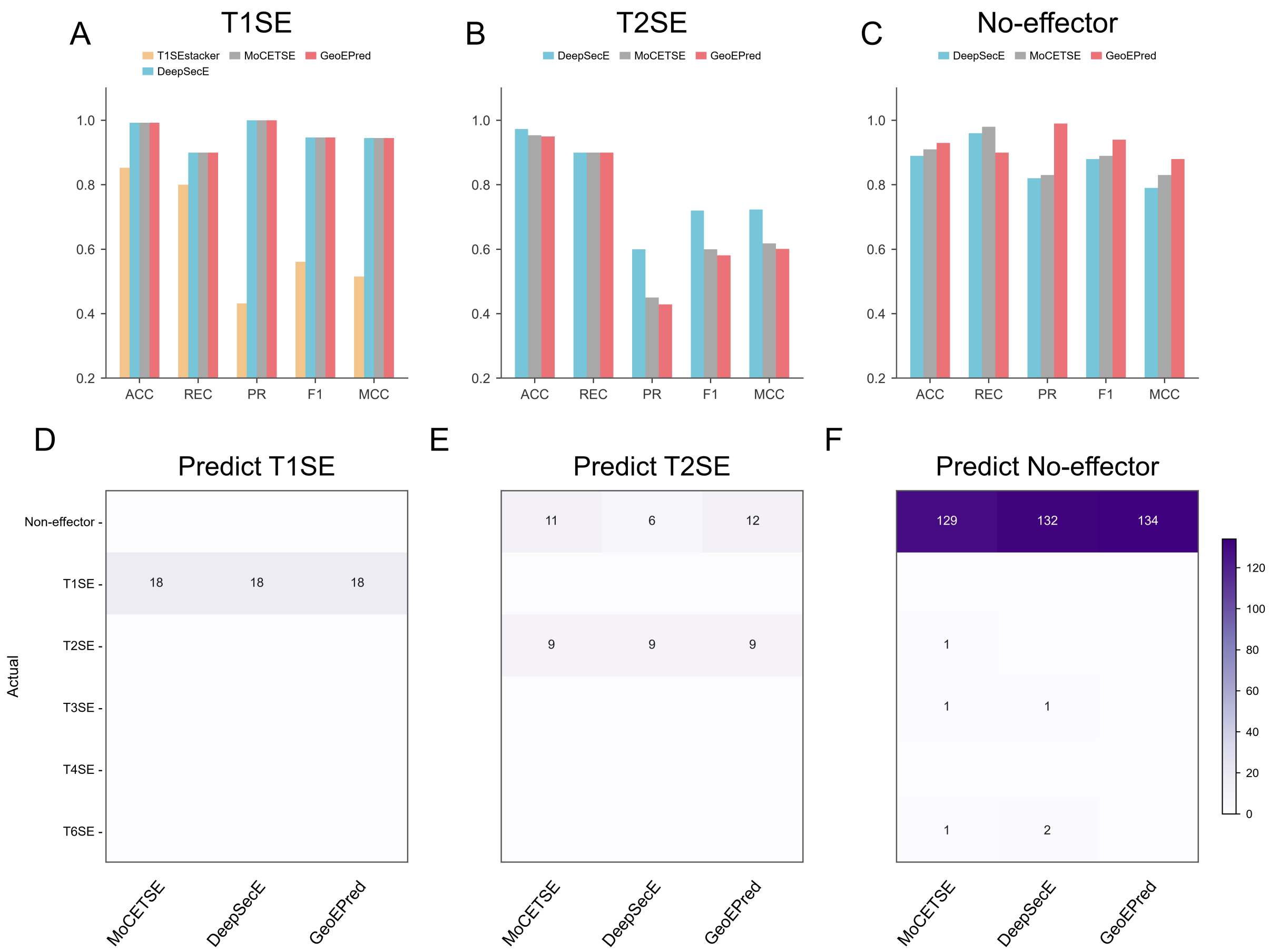

S2 Fig. Performance comparison of GeoEPred and mainstream models in type I (T1SE) and type II (T2SE) secretion effector protein prediction tasks. A–B: Model performance was evaluated on the benchmark set (Dataset S3) and a dataset containing 150 non-effector proteins and 10 T2SEs, using accuracy (ACC), recall (REC), precision (PR), F1 score (F1), and Matthews correlation coefficient (MCC). D–F: Comparison of true-positive and false-positive predictions for T1SE, T2SE, and non-effectors across different models. The heatmaps show the sample distributions predicted as T1SE, T2SE, non-effector, and other effector protein types.
