# Supplementary material for: GeoEPred: A multimodal structure-aware geometric deep learning framework for Gram-negative bacterial secreted effector prediction with sequence semantics
Source: PLoS Comput Biol. 2026 Sep 30;22(9):e1014344. doi: 10.1371/journal.pcbi.1014344 (PMC13641719; doi:10.1371/journal.pcbi.1014344)
Supplement: Supplementary file 4 [file pcbi.1014344.s004.pdf]

Description of datasets with distant homology

To assess the generalization of GeoEPred to remote homologs, we constructed a dedicated benchmark using the final training set as the reference dataset. The candidate evaluation pool consisted of two components: 260 proteins from the fixed independent test set and additional structurally similar candidate proteins retrieved from the PDB100, AFDB-SWISSPROT, and AFDB-PROTEOME databases [1], [2] using the training-set structures as Foldseek queries [3].

Among the external search results, we retained only entries with clearly defined protein names, functional descriptions, taxonomic origins, database accession numbers, and sufficient annotation or experimental evidence to support class assignment. Entries annotated solely as "putative proteins" or "uncharacterized proteins" were excluded. The annotations of PDB100 hits were further cross-checked against the corresponding PDB records and original publications [4], whereas annotations from AFDB-SWISSPROT and AFDB-PROTEOME were independently verified against their corresponding UniProt records [5].

All candidate proteins were subjected to the same two-stage filtering procedure. First, their sequences were compared against the complete training set using CD-HIT-2D [6], and only candidates with a maximum sequence identity below 30% to every training-set protein were retained. For each sequence-filtered candidate, Foldseek [3] was then used to search against the complete training structure set to identify structurally similar training proteins, and the resulting candidate--training structure pairs were subsequently realigned using TM-align [8]. The TM-score was calculated as:

$$\text{TM-score} = \max\left[\frac{1}{L_{\text{target}}}\sum_{i=1}^{L_{\text{ali}}}\frac{1}{1+\left(\frac{d_i}{d_0(L_{\text{target}})}\right)^2}\right],$$

where

$$d_0(L_{\text{target}}) = 1.24\,(L_{\text{target}} - 15)^{1/3} - 1.8.$$

Here, L_{target} denotes the length of the candidate benchmark protein used for normalization, L_{ali} represents the number of aligned residue pairs, and d_i is the distance between the i-th pair of aligned residues after structural superposition. The maximization is performed over alternative structural superpositions to obtain the optimal TM-score. TM-score was introduced by Zhang and Skolnick as a length-normalized measure of protein structural similarity [7], and TM-align optimizes structural alignment based on TM-score [8]. Throughout benchmark construction, the TM-score normalized by the length of the candidate protein was used. Following commonly used criteria in previous studies [8], [9], we retained as remote-homology benchmark samples proteins that shared less than 30% sequence identity with every protein in the training set but exhibited a maximum TM-score greater than 0.5 to at least one training-set structure.

Before establishing the final benchmark, duplicate entries across all candidate sources and proteins overlapping with the training set were removed. We further confirmed that no identical protein sequences were shared between the final benchmark and the training set and that every retained benchmark protein had less than 30% sequence identity to every training-set protein. The final class distribution is presented in S3 Fig. No T2SE proteins simultaneously satisfied the sequence-divergence, structural-similarity, and annotation criteria; therefore, T2SE was not represented in the final benchmark.

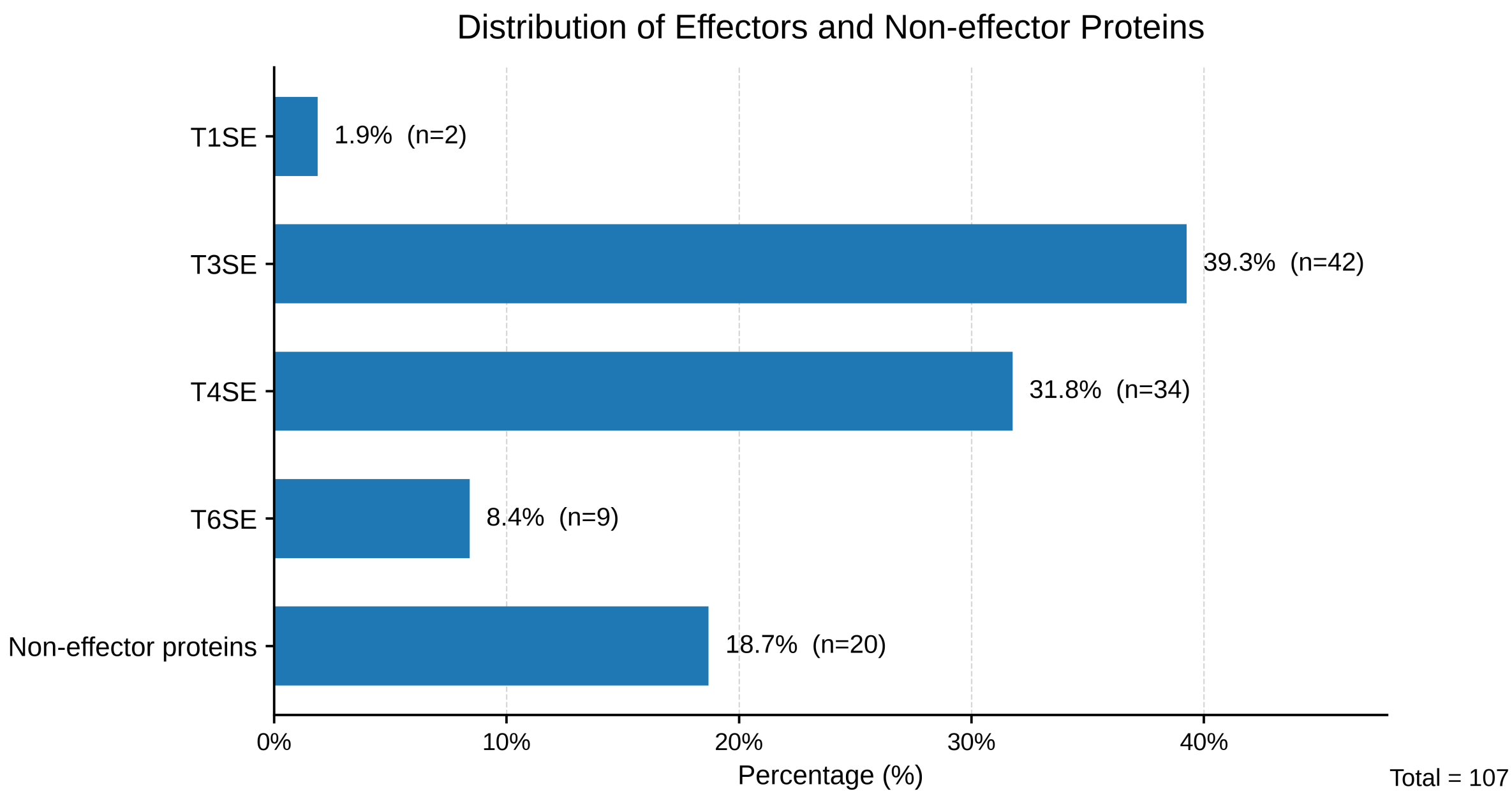

S3 Fig. The dataset contains samples with distant homology, including 2 T1SE, 42 T3SE, 34 T4SE, 9 T6SE, and 20 non-effector proteins.

Reference

[1] Jumper J, Evans R, Pritzel A, et al. Highly accurate protein structure prediction with AlphaFold. *Nature*. 2021;596:583–589. https://doi.org/10.1038/s41586-021-03819-2
[2] Varadi M, Bertoni D, Magana P, et al. AlphaFold Protein Structure Database in 2024: providing structure coverage for over 214 million protein sequences. *Nucleic Acids Research*. 2024;52:D368–D375. https://doi.org/10.1093/nar/gkad1011
[3] van Kempen M, Kim SS, Tumescheit C, et al. Fast and accurate protein structure search with Foldseek. *Nature Biotechnology*. 2024;42:243–246. https://doi.org/10.1038/s41587-023-01773-0
[4] Burley SK, Bhikadiya C, Bi C, et al. RCSB Protein Data Bank: powerful new tools for exploring 3D structures of biological macromolecules for basic and applied research and education in fundamental biology, biomedicine, biotechnology, bioengineering and energy sciences. *Nucleic Acids Research*. 2021;49:D437–D451. https://doi.org/10.1093/nar/gkaa1038
[5] UniProt Consortium. UniProt: the universal protein knowledgebase in 2023. *Nucleic Acids Research*. 2023;51:D523–D531. https://doi.org/10.1093/nar/gkac1052
[6] Li W, Godzik A. CD-HIT: a fast program for clustering and comparing large sets of protein or nucleotide sequences. *Bioinformatics*. 2006;22:1658–1659. https://doi.org/10.1093/bioinformatics/btl158
[7] Zhang Y, Skolnick J. Scoring function for automated assessment of protein structure template quality. *Proteins*. 2004;57:702–710. https://doi.org/10.1002/prot.20264
[8] Zhang Y, Skolnick J. TM-align: a protein structure alignment algorithm based on the TM-score. *Nucleic Acids Research*. 2005;33:2302–2309. https://doi.org/10.1093/nar/gki524
[9] Xu J, Zhang Y. How significant is a protein structure similarity with TM-score = 0.5? *Bioinformatics*. 2010;26:889–895. https://doi.org/10.1093/bioinformatics/btq066
