# Supplementary material for: GeoEPred: A multimodal structure-aware geometric deep learning framework for Gram-negative bacterial secreted effector prediction with sequence semantics
Source: PLoS Comput Biol. 2026 Sep 30;22(9):e1014344. doi: 10.1371/journal.pcbi.1014344 (PMC13641719; doi:10.1371/journal.pcbi.1014344)
Supplement: Supplementary file 6 [file pcbi.1014344.s006.pdf]

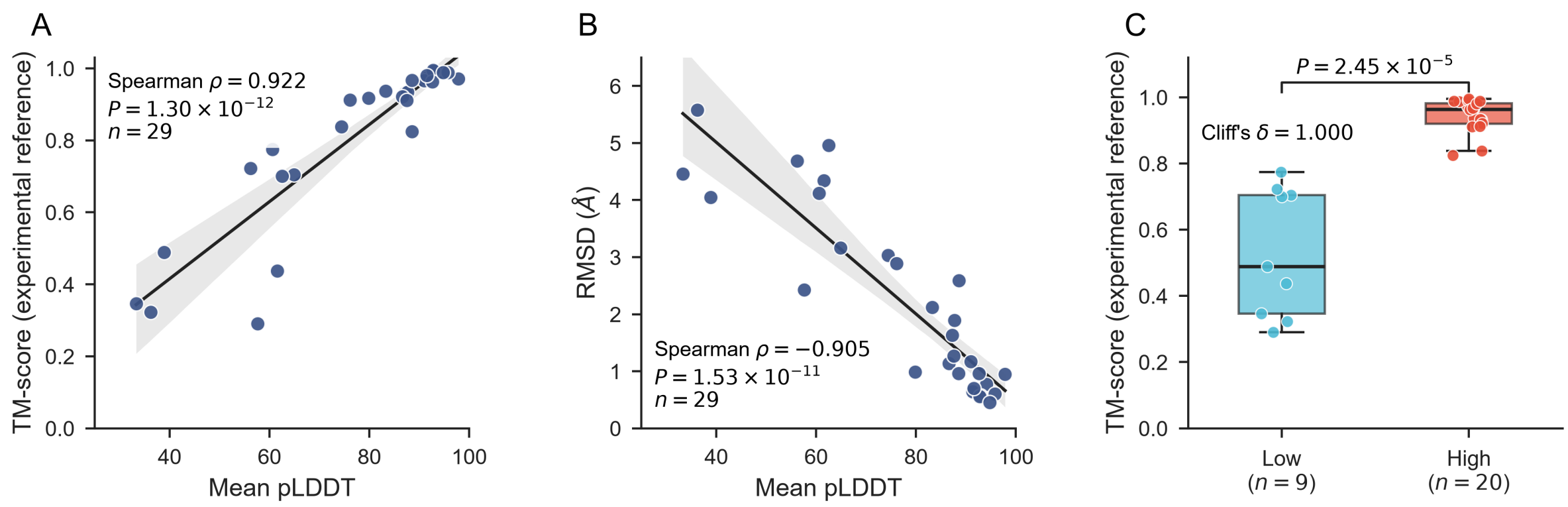

S5 Fig. Comparison of ESMFold-predicted structures with experimentally resolved protein structures. A: Mean pLDDT versus TM-score. B: Mean pLDDT versus RMSD. C: Comparison of TM-scores between low-confidence (pLDDT < 70) and high-confidence (pLDDT ≥ 70) predictions. Each point represents one protein.
