# Supplementary material for: GeoEPred: A multimodal structure-aware geometric deep learning framework for Gram-negative bacterial secreted effector prediction with sequence semantics
Source: PLoS Comput Biol. 2026 Sep 30;22(9):e1014344. doi: 10.1371/journal.pcbi.1014344 (PMC13641719; doi:10.1371/journal.pcbi.1014344)
Supplement: Supplementary file 7 [file pcbi.1014344.s007.pdf]

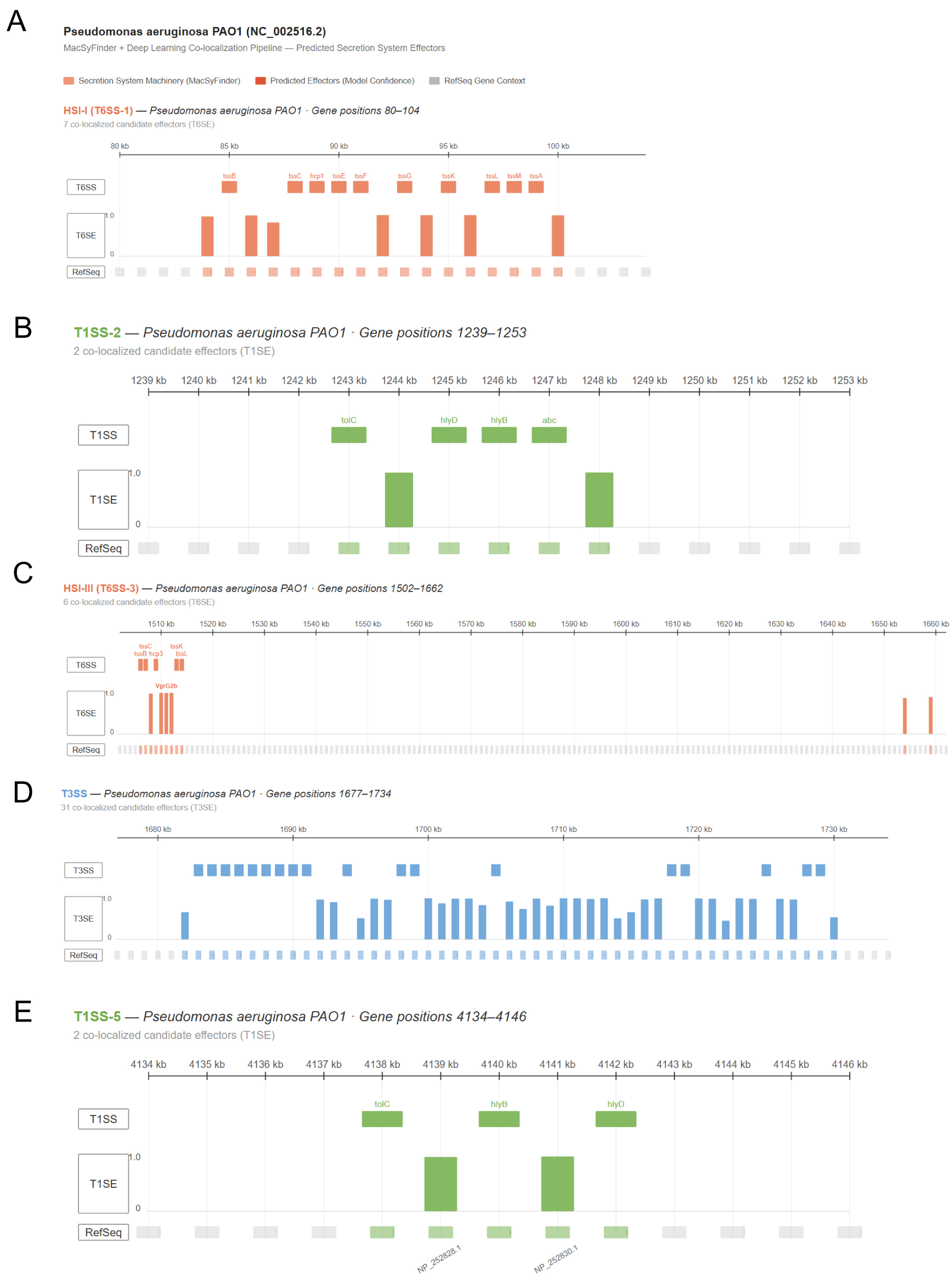

S6 Fig. Genome-wide prediction visualization shows that GeoEPred not only successfully identifies T6SEs in *Pseudomonas aeruginosa*, but also predicts other candidate effectors in the genome (A–E).
