# Supplementary material for: GeoEPred: A multimodal structure-aware geometric deep learning framework for Gram-negative bacterial secreted effector prediction with sequence semantics
Source: PLoS Comput Biol. 2026 Sep 30;22(9):e1014344. doi: 10.1371/journal.pcbi.1014344 (PMC13641719; doi:10.1371/journal.pcbi.1014344)
Supplement: Supplementary file 8 [file pcbi.1014344.s008.pdf]

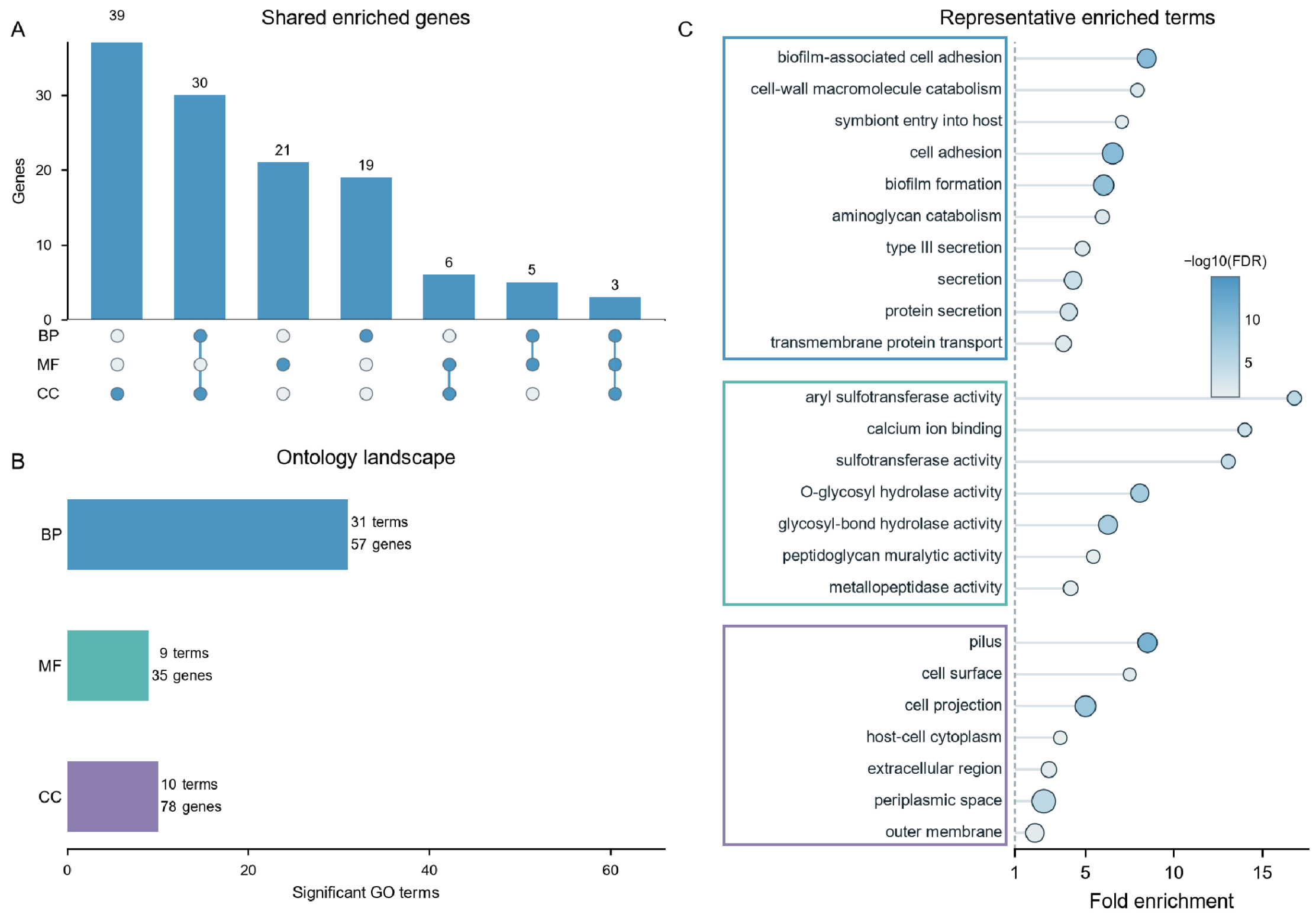

S7 Fig. Gene Ontology (GO) functional enrichment analysis of predicted candidate effectors in *Salmonella Typhimurium* LT2. A: The bar plot displays the number of genes shared among different combinations of Biological Process (BP), Molecular Function (MF), and Cellular Component (CC), as indicated by the connected dot matrix below. B: Ontology landscape. Overview of the significant GO terms and associated gene counts categorized by BP (blue), MF (teal), and CC (purple). C: Representative enriched terms. Lollipop charts illustrating representative enriched terms for BP, MF, and CC, respectively. The x-axis represents the fold enrichment, and the bubble size and color intensity correspond to the statistical significance scaled.
