# Supplementary material for: GeoEPred: A multimodal structure-aware geometric deep learning framework for Gram-negative bacterial secreted effector prediction with sequence semantics
Source: PLoS Comput Biol. 2026 Sep 30;22(9):e1014344. doi: 10.1371/journal.pcbi.1014344 (PMC13641719; doi:10.1371/journal.pcbi.1014344)
Supplement: Supplementary file 9 [file pcbi.1014344.s009.pdf]

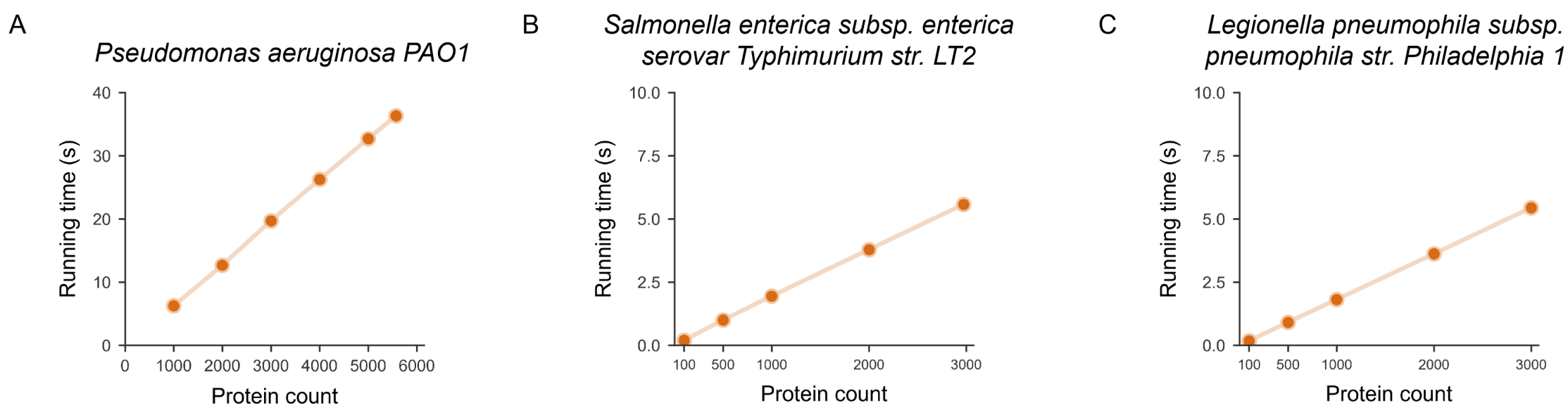

S8 Fig. Model inference time scaling with protein dataset sizes. Evaluation of the model's running time across different protein counts for three bacterial strains. A: Pseudomonas aeruginosa PAO1, B: Salmonella enterica subsp. enterica serovar Typhimurium str. LT2, and C: Legionella pneumophila subsp. pneumophila str. Philadelphia 1. The orange dots and line represent the linear relationship between the running time (in seconds) and the number of processed proteins, demonstrating robust processing efficiency.
