# Supplementary material for: GeoEPred: A multimodal structure-aware geometric deep learning framework for Gram-negative bacterial secreted effector prediction with sequence semantics
Source: PLoS Comput Biol. 2026 Sep 30;22(9):e1014344. doi: 10.1371/journal.pcbi.1014344 (PMC13641719; doi:10.1371/journal.pcbi.1014344)
Supplement: Supplementary file 10 [file pcbi.1014344.s010.pdf]

S1 Table. Summary of the training, testing, and benchmarking datasets used in the comparative experiments

Datasets	Sample information	Source
S1	Train dataset used to train GeoEPred. Containing 1,575 non-effector proteins, 130 T1SEs, 68 T2SEs, 521 T3SEs, 675 T4SEs, and 272 T6SEs. Specifically, T1SE and T2SE sequences were derived from the TSE-ARF dataset [1]. Data for T3SE and T6SE were collected from Bastion3 [2], DeepT3 [3], SecReT6 [4], Bastion6 [5], DeepT3_4 [5], and TSE-ARF [1]. For T4SE, sequences were compiled from DeepSecE [6], OPT4e [7], T4SEfinder [8], iT4SE-EP [9], Bastion4 [10], T4SE-XGB [11], TSE-ARF [1], T4SEpp [12], and DeepT3_4 [5].	Zhang et al. [6]
S2	Independent test dataset used for model testing. Containing 150 non-effector proteins, 20 T1SEs, 10 T2SEs, 30 T3SEs, 30 T4SEs, and 20 T6SEs. T1SEs and T2SEs were obtained by random sampling from the raw data in the BastionHub database [13], while T3SEs, T4SEs, T6SEs, and non-secretory proteins were derived from the test datasets of Bastion3 [2], CNN-T4SE [14], and Bastion6 [5].	Zhang et al. [6]
S3	Test dataset used for T1SEs benchmarking. Containing 150 non-T1SEs and 20 T1SEs. Both positive and negative samples were derived from the independent test dataset (Dataset S2).	Zhang et al. [6]
S4	Test dataset used for T3SEs benchmarking. Containing 108 non-T3SEs and 30 T3SEs. The positive samples were obtained from recent literature, while the negative samples were sourced from non-T3SE proteins of different bacterial species.	Wang et al. [2]
S5	Test dataset used for T4SEs benchmarking. Containing 150 non-T4SEs and 30 T4SEs. The positive samples were obtained from the UniProt database [15] and the study by Meyer et al. [16], while the negative samples were sourced from the *Vibrio parahaemolyticus* O3:K6 (strain RIMD 2210633) serotype dataset [17].	Hong et al. [14]

Datasets	Sample information	Source
S6	Test dataset used for T6SEs benchmarking. Containing 200 non-T6SEs and 20 T6SEs. The positive samples were obtained from relevant literature, while the negative samples were sourced from Vibrio parahaemolyticus strains.	Wang et al. [5]

Reference

[1] Tang X, Luo L, Wang S. TSE-ARF: An adaptive prediction method of effectors across secretion system types. *Analytical Biochemistry*. 2024;686:115407. https://doi.org/10.1016/j.ab.2023.115407

[2] Wang J, Li J, Yang B, et al. Bastion3: a two-layer ensemble predictor of type III secreted effectors. *Bioinformatics*. 2019;35:2017–2028. https://doi.org/10.1093/bioinformatics/bty937

[3] Xue L, Tang B, Chen W, Luo J. DeepT3: deep convolutional neural networks accurately identify Gram-negative bacterial type III secreted effectors using the N-terminal sequence. *Bioinformatics*. 2019;35:2051–2057. https://doi.org/10.1093/bioinformatics/bty933

[4] Zhang J, Guan J, Wang M, et al. SecReT6 update: a comprehensive resource of bacterial Type VI Secretion Systems. *Science China Life Sciences*. 2023;66:626–634. https://doi.org/10.1007/s11427-022-2180-2

[5] Wang J, Yang B, Leier A, et al. Bastion6: a bioinformatics approach for accurate prediction of type VI secreted effectors. *Bioinformatics*. 2018;34:2546–2555. https://doi.org/10.1093/bioinformatics/bty155

[6] Zhang Y, Guan J, Li C, et al. DeepSecE: A Deep-Learning-Based Framework for Multiclass Prediction of Secreted Proteins in Gram-Negative Bacteria. *Research*. 2023;6:0258. https://doi.org/10.34133/research.0258

[7] Esna Ashari Z, Brayton KA, Broschat SL. Prediction of T4SS effector proteins for *Anaplasma phagocytophilum* using OPT4e. *Frontiers in Microbiology*. 2019;10:1391. https://doi.org/10.3389/fmicb.2019.01391

[8] Zhang Y, Zhang Y, Xiong Y, et al. T4SEfinder: genome-scale prediction of bacterial type IV secreted effectors using a pre-trained protein language model. *Briefings in Bioinformatics*. 2022;23:bbab420. https://doi.org/10.1093/bib/bbab420

[9] Han H, Ding C, Cheng X, et al. iT4SE-EP: Accurate identification of bacterial type IV secreted effectors. *Molecules*. 2021;26:2487. https://doi.org/10.3390/molecules26092487

[10] Wang J, Yang B, An Y, et al. Systematic analysis and prediction of type IV secreted effector proteins by machine learning approaches. *Briefings in Bioinformatics*. 2019;20:931–951. https://doi.org/10.1093/bib/bbx164

[11] Chen T, Wang X, Chu Y, et al. T4SE-XGB: interpretable sequence-based prediction of type IV secreted effectors. *Frontiers in Microbiology*. 2020;11:580382. https://doi.org/10.3389/fmicb.2020.580382

[12] Hu Y, Wang Y, Hu X, et al. T4SEpp: A pipeline integrating protein language models to predict bacterial type IV secreted effectors. *Computational and Structural Biotechnology Journal*. 2024;23:801–812. https://doi.org/10.1016/j.csbj.2024.01.002

[13] Wang J, Li J, Hou Y, et al. BastionHub: a universal platform for integrating and analyzing substrates secreted by Gram-negative bacteria. *Nucleic Acids Research*. 2021;49:D651–D659. https://doi.org/10.1093/nar/gkaa899
[14] Hong J, Luo Y, Mou M, et al. Convolutional neural network-based annotation of bacterial type IV secretion system effectors. *Briefings in Bioinformatics*. 2020;21:1825–1836. https://doi.org/10.1093/bib/bbz120
[15] UniProt Consortium. The Universal Protein Resource (UniProt) in 2010. *Nucleic Acids Research*. 2010;38:D142–D148. https://doi.org/10.1093/nar/gkp846
[16] Meyer DF, Noroy C, Moumène A, et al. Searching algorithm for type IV secretion system effectors 1.0. *Nucleic Acids Research*. 2013;41:9218–9229. https://doi.org/10.1093/nar/gkt718
[17] Makino K, Oshima K, Kurokawa K, et al. Genome sequence of *Vibrio parahaemolyticus*: a pathogenic mechanism distinct from that of *V. cholerae*. *Lancet*. 2003;361:743–749. https://doi.org/10.1016/S0140-6736(03)12659-1
