# Supplementary material for: GeoEPred: A multimodal structure-aware geometric deep learning framework for Gram-negative bacterial secreted effector prediction with sequence semantics
Source: PLoS Comput Biol. 2026 Sep 30;22(9):e1014344. doi: 10.1371/journal.pcbi.1014344 (PMC13641719; doi:10.1371/journal.pcbi.1014344)
Supplement: Supplementary file 11 [file pcbi.1014344.s011.pdf]

S2 Table. Web server links of the comparative models used in this study.

Tools	Resource	Link
DeepSecE	Zhang et al. [3]	https://tool2-mml.sjtu.edu.cn/DeepSecEdb/
BastionX	Wang et al. [7]	https://bastionx.erc.monash.edu/
T1SEstacker	Chen et al. [9]	http://61.160.194.165:3080/T1SEstacker/index.html
Bastion3	Wang et al. [1]	https://bastion3.erc.monash.edu/
T3SEpp	Hui et al. [10]	http://www.szu-bioinf.org/T3SEpp/
EP3	Li et al. [11]	http://lab.malab.cn/~lijing/EP3.html
Bastion4	Wang et al. [5]	https://bastion4.erc.monash.edu/
CNN-T4SE	Hong et al. [8]	https://idrblab.org/cnnt4se/
T4SEfinder	Zhang et al. [4]	https://tool2-mml.sjtu.edu.cn/T4SEfinder_TAPE/
T4SEpp	Hu et al. [6]	https://bis.zju.edu.cn/T4SEpp/
Bastion6	Wang et al. [2]	https://bastion6.erc.monash.edu/

Reference
[1] Wang J, Li J, Yang B, et al. Bastion3: a two-layer ensemble predictor of type III secreted effectors. *Bioinformatics*. 2019;35:2017–2028. https://doi.org/10.1093/bioinformatics/bty937
[2] Wang J, Yang B, Leier A, et al. Bastion6: a bioinformatics approach for accurate prediction of type VI secreted effectors. *Bioinformatics*. 2018;34:2546–2555. https://doi.org/10.1093/bioinformatics/bty155
[3] Zhang Y, Guan J, Li C, et al. DeepSecE: A Deep-Learning-Based Framework for Multiclass Prediction of Secreted Proteins in Gram-Negative Bacteria. *Research*. 2023;6:0258. https://doi.org/10.34133/research.0258
[4] Zhang Y, Zhang Y, Xiong Y, et al. T4SEfinder: genome-scale prediction of bacterial type IV secreted effectors using a pre-trained protein language model. *Briefings in Bioinformatics*. 2022;23:bbab420. https://doi.org/10.1093/bib/bbab420
[5] Wang J, Yang B, An Y, et al. Systematic analysis and prediction of type IV secreted effector proteins by machine learning approaches. *Briefings in Bioinformatics*. 2019;20:931–951. https://doi.org/10.1093/bib/bbx164
[6] Hu Y, Wang Y, Hu X, et al. T4SEpp: A pipeline integrating protein language models to predict bacterial type IV secreted effectors. *Computational and Structural Biotechnology Journal*. 2024;23:801–812. https://doi.org/10.1016/j.csbj.2024.01.002
[7] Wang J, Li J, Hou Y, et al. BastionHub: a universal platform for integrating and analyzing substrates secreted by Gram-negative bacteria. *Nucleic Acids Research*. 2021;49:D651–D659. https://doi.org/10.1093/nar/gkaa899

[8] Hong J, Luo Y, Mou M, et al. Convolutional neural network-based annotation of bacterial type IV secretion system effectors. *Briefings in Bioinformatics*. 2020;21:1825–1836. https://doi.org/10.1093/bib/bbz120
[9] Chen Z, Zhao Z, Hui X, et al. T1SEstacker: A tri-layer stacking model for bacterial type 1 secreted proteins. *Frontiers in Microbiology*. 2022;12:813094. https://doi.org/10.3389/fmicb.2021.813094
[10] Hui X, Chen Z, Lin M, et al. T3SEpp: an integrated prediction pipeline for bacterial type III secreted effectors. *mSystems*. 2020;5:e00624–20. https://doi.org/10.1128/mSystems.00624-20
[11] Li J, Wei L, Guo F, Zou Q. EP3: an ensemble predictor that accurately identifies type III secreted effectors. *Briefings in Bioinformatics*. 2021;22:1918–1928. https://doi.org/10.1093/bib/bbaa032
