# Supplementary material for: GeoEPred: A multimodal structure-aware geometric deep learning framework for Gram-negative bacterial secreted effector prediction with sequence semantics
Source: PLoS Comput Biol. 2026 Sep 30;22(9):e1014344. doi: 10.1371/journal.pcbi.1014344 (PMC13641719; doi:10.1371/journal.pcbi.1014344)
Supplement: Supplementary file 12 [file pcbi.1014344.s012.pdf]

S3 Table. Comparison of the performance of GeoEPred on independent test sets based on ESM-1b, ESM-2, and other protein language models.

PLM	ACC (%)	F1-Macro	F1-Weight	Precision	Recall	MCC
ESM-2 (t36, 3B)	0.923	0.884	0.931	0.858	0.943	0.888
ESM-2 (t33, 650M)	0.896	0.861	0.902	0.831	0.919	0.848
ESM-2 (t30, 150M)	0.888	0.852	0.892	0.827	0.896	0.830
ESM-1b (t33,650M)	0.888	0.851	0.891	0.838	0.880	0.833
ProtT5-XL-U50	0.873	0.849	0.878	0.805	0.927	0.822
ProtBERT-BFD	0.861	0.826	0.867	0.791	0.903	0.803

To select the optimal protein language model (PLM) for GeoEPred, we evaluated several state-of-the-art PLMs, including ESM-2 (three sizes) [1], ESM-1b [2], ProtT5-XL-U50, and ProtBERT-BFD [3], on benchmark datasets. Performance metrics included Accuracy (ACC), F1-Macro, F1-Weight, Precision, Recall, and Matthews Correlation Coefficient (MCC). As shown in S3 Table, ESM-2 (t36, 3B) consistently achieved high scores across multiple metrics, particularly in Recall and MCC, which are crucial for accurate effector protein prediction. Therefore, we selected ESM-2 (t36, 3B) as the backbone model for GeoEPred to balance predictive performance and computational efficiency.

Reference

[1] Lin Z, Akin H, Rao R, et al. Evolutionary-scale prediction of atomic-level protein structure with a language model. *Science*. 2023;379:1123–1130. https://doi.org/10.1126/science.ade2574

[2] Rives A, Meier J, Sercu T, et al. Biological structure and function emerge from scaling unsupervised learning to 250 million protein sequences. *Proceedings of the National Academy of Sciences USA*. 2021;118:e2016239118. https://doi.org/10.1073/pnas.2016239118

[3] Elnaggar A, Heinzinger M, Dallago C, et al. ProtTrans: Toward understanding the language of life through self-supervised learning. *IEEE Transactions on Pattern Analysis and Machine Intelligence*. 2022;44:7112–7127. https://doi.org/10.1109/TPAMI.2021.3095381
