# Supplementary material for: GeoEPred: A multimodal structure-aware geometric deep learning framework for Gram-negative bacterial secreted effector prediction with sequence semantics
Source: PLoS Comput Biol. 2026 Sep 30;22(9):e1014344. doi: 10.1371/journal.pcbi.1014344 (PMC13641719; doi:10.1371/journal.pcbi.1014344)
Supplement: Supplementary file 13 [file pcbi.1014344.s013.pdf]

S4 Table. GitHub repositories of protein language models evaluated for GeoEPred.

PLM	Resource	GitHub Link
ESM-2 (t36, 3B)	Facebook Research	https://github.com/facebookresearch/esm
ESM-2 (t33, 650M)	Facebook Research	https://github.com/facebookresearch/esm
ESM-2 (t30, 150M)	Facebook Research	https://github.com/facebookresearch/esm
ESM-1b (t33, 650M)	Facebook Research	https://github.com/facebookresearch/esm
ProtT5-XL-U50	Agemagician / ProtTrans	https://github.com/agemagician/ProtTrans
ProtBERT-BFD	Agemagician / ProtTrans	https://github.com/agemagician/ProtTrans
