# Supplementary material for: GeoEPred: A multimodal structure-aware geometric deep learning framework for Gram-negative bacterial secreted effector prediction with sequence semantics
Source: PLoS Comput Biol. 2026 Sep 30;22(9):e1014344. doi: 10.1371/journal.pcbi.1014344 (PMC13641719; doi:10.1371/journal.pcbi.1014344)
Supplement: Supplementary file 14 [file pcbi.1014344.s014.pdf]

S5 Table. Compared with the single-modal model, the proposed model shows improvements in performance, 95% confidence intervals, and statistical significance analysis.

Baseline Model	Metric	Δ	95% CI	*p*-values
Geometric Priors	ACC	+0.3962	[0.3308, 0.4577]	0.0020
	F1	+0.4258	[0.3512, 0.5025]	0.0020
	MCC	+0.5742	[0.4860, 0.6646]	0.0020
	AUC	+0.1659	[0.1349, 0.1969]	0.0020
	AUPRC	+0.4606	[0.3743, 0.5462]	0.0020
Sequence only	ACC	+0.2000	[0.1499, 0.2538]	0.0020
	F1	+0.2203	[0.1555, 0.2893]	0.0020
	MCC	+0.2658	[0.1969, 0.3371]	0.0020
	AUC	+0.0599	[0.0420, 0.0809]	0.0020
	AUPRC	+0.2315	[0.1589, 0.3152]	0.0020
Sequence Semantics	ACC	+0.0423	[0.0115, 0.0731]	0.0080
	F1	+0.0379	[0.0069, 0.0702]	0.0140
	MCC	+0.0604	[0.0194, 0.1043]	0.0020
	AUC	+0.0082	[0.0000, 0.0180]	0.0540
	AUPRC	+0.0081	[-0.0257, 0.0563]	0.5035
