# Supplementary material for: GeoEPred: A multimodal structure-aware geometric deep learning framework for Gram-negative bacterial secreted effector prediction with sequence semantics
Source: PLoS Comput Biol. 2026 Sep 30;22(9):e1014344. doi: 10.1371/journal.pcbi.1014344 (PMC13641719; doi:10.1371/journal.pcbi.1014344)
Supplement: Supplementary file 15 [file pcbi.1014344.s015.pdf]

S6 Table. Comparison of different models for effector protein prediction.

Model	N_{total}	N_{correct}	Accuracy
GeoEPred	107	97	90.7%
Sequence semantics	107	88	82.2%
Only sequence	107	70	65.4%
