# Supplementary material for: GeoEPred: A multimodal structure-aware geometric deep learning framework for Gram-negative bacterial secreted effector prediction with sequence semantics
Source: PLoS Comput Biol. 2026 Sep 30;22(9):e1014344. doi: 10.1371/journal.pcbi.1014344 (PMC13641719; doi:10.1371/journal.pcbi.1014344)
Supplement: Supplementary file 16 [file pcbi.1014344.s016.pdf]

S7 Table. Performance gains, 95% confidence intervals, and statistical significance of the full model compared with different ablated model variants.

Model Variant	Metric	Δ	95% CI	*p*-values
w/o Contrastive Learning	ACC	+0.0346	[0.0038, 0.0692]	0.0320
	F1	+0.0300	[0.00002, 0.0602]	0.0519
	MCC	+0.0485	[0.0094, 0.0910]	0.0140
	AUC	+0.0064	[-0.0047, 0.0217]	0.2917
	AUPRC	+0.0278	[-0.0043, 0.0686]	0.1019
w/o Adaptive Attention Pooling	ACC	+0.0385	[0.0038, 0.0769]	0.0380
	F1	+0.0338	[-0.0071, 0.0816]	0.0939
	MCC	+0.0532	[0.0083, 0.1014]	0.0220
	AUC	+0.0034	[-0.0077, 0.0134]	0.5554
	AUPRC	+0.0249	[-0.0091, 0.0789]	0.1379
w/o Feature Tokenization	ACC	+0.0500	[0.0231, 0.0808]	0.0020
	F1	+0.0461	[0.0181, 0.0764]	0.0020
	MCC	+0.0683	[0.0303, 0.1085]	0.0020
	AUC	+0.0007	[-0.0118, 0.0102]	0.8352
	AUPRC	+0.0394	[0.0112, 0.0888]	0.0040
