# Supplementary material for: GeoEPred: A multimodal structure-aware geometric deep learning framework for Gram-negative bacterial secreted effector prediction with sequence semantics
Source: PLoS Comput Biol. 2026 Sep 30;22(9):e1014344. doi: 10.1371/journal.pcbi.1014344 (PMC13641719; doi:10.1371/journal.pcbi.1014344)
Supplement: Supplementary file 17 [file pcbi.1014344.s017.pdf]

S8 Table. Performance improvements, 95% confidence intervals, and statistical significance analysis of the proposed model compared with different fusion strategies.

Method	Metric	Δ	95% CI	*p*-values
Concatenation	ACC	+0.0346	[0.0038, 0.0692]	0.0360
	F1	+0.0368	[-0.0006, 0.0792]	0.0559
	AUC	+0.0039	[-0.0087, 0.0146]	0.4795
	AUPRC	+0.0521	[0.0195, 0.1043]	0.0020
Cross-Attention Fusion	ACC	+0.0154	[-0.0154, 0.0462]	0.3916
	F1	+0.0224	[-0.0152, 0.0670]	0.2717
	AUC	+0.0054	[-0.0046, 0.0159]	0.2877
	AUPRC	+0.0415	[0.0070, 0.0908]	0.0120
