# Supplementary material for: GeoEPred: A multimodal structure-aware geometric deep learning framework for Gram-negative bacterial secreted effector prediction with sequence semantics
Source: PLoS Comput Biol. 2026 Sep 30;22(9):e1014344. doi: 10.1371/journal.pcbi.1014344 (PMC13641719; doi:10.1371/journal.pcbi.1014344)
Supplement: Supplementary file 18 [file pcbi.1014344.s018.pdf]

S9 Table. Performance gain, 95% confidence intervals, and statistical significance analysis of the proposed model compared to the best traditional machine learning and deep learning baseline methods.

| Method | Metric | Δ | 95% CI | *p*-values |
|---|---|---|---|---|
| SVM | ACC | +0.015 | [0.003, 0.031] | 0.024 |
| | F1 | +0.002 | [-0.003, 0.007] | 0.420 |
| | AUC | +0.021 | [0.005, 0.037] | 0.009 |
| | AUPRC | +0.030 | [0.020, 0.058] | 0.002 |
| BiLSTM_Attention | ACC | +0.023 | [0.008, 0.042] | 0.008 |
| | F1 | +0.016 | [0.014, 0.052] | 0.028 |
| | AUC | +0.028 | [0.010, 0.046] | 0.002 |
| | AUPRC | +0.030 | [0.018, 0.060] | 0.003 |
