# Supplementary material for: GeoEPred: A multimodal structure-aware geometric deep learning framework for Gram-negative bacterial secreted effector prediction with sequence semantics
Source: PLoS Comput Biol. 2026 Sep 30;22(9):e1014344. doi: 10.1371/journal.pcbi.1014344 (PMC13641719; doi:10.1371/journal.pcbi.1014344)
Supplement: Supplementary file 19 [file pcbi.1014344.s019.pdf]

S10 Table. Performance comparison between GeoEPred and existing popular methods on benchmark datasets

Task	Method	ACC	REC	PR	F1	MCC
T1SE	MoCETSE	0.992	0.900	1.000	0.947	0.945
	DeepSecE	0.992	0.900	1.000	0.947	0.945
	T1SEstacker	0.853	0.800	0.432	0.561	0.515
	GeoEPred	**0.992**	**0.900**	**1.000**	**0.947**	**0.945**
T2SE	MoCETSE	0.954	0.900	0.450	0.600	0.618
	DeepSecE	0.973	0.900	0.600	0.720	0.723
	GeoEPred	0.950	**0.900**	0.429	0.581	0.601
T3SE	Bastion3	0.790	1.000	0.508	0.674	0.610
	T3SEpp	0.913	0.700	0.875	0.778	0.732
	BastionX	0.514	1.000	0.309	0.473	0.343
	DeepSecE	0.969	0.967	0.806	0.879	0.866
	MoCETSE	0.981	0.933	0.903	0.918	0.916
	GeoEPred	**0.992**	0.967	**0.967**	**0.967**	**0.962**
T4SE	Bastion3	0.939	0.967	0.744	0.841	0.814
	T4SEfinder	0.778	0.933	0.424	0.583	0.526
	T4SEpp	0.956	1.000	0.790	0.882	0.865
	BastionX	0.667	1.000	0.333	0.500	0.447
	DeepSecE	0.962	0.967	0.763	0.853	0.839
	MoCETSE	0.954	1.000	0.714	0.833	0.823
	GeoEPred	**0.985**	**1.000**	**0.882**	**0.938**	**0.931**
T6SE	Bastion6	0.709	0.950	0.232	0.373	0.378
	BastionX	0.755	1.000	0.270	0.426	0.444
	DeepSecE	0.977	0.800	0.889	0.842	0.831
	MoCETSE	0.989	0.850	1.000	0.918	0.916
	GeoEPred	**0.989**	**1.000**	0.867	**0.930**	**0.927**

ACC: Accuracy; REC: Recall; PR: Precision; F1: F1-score; MCC: Matthews Correlation Coefficient.
