# Supplementary material for: GeoEPred: A multimodal structure-aware geometric deep learning framework for Gram-negative bacterial secreted effector prediction with sequence semantics
Source: PLoS Comput Biol. 2026 Sep 30;22(9):e1014344. doi: 10.1371/journal.pcbi.1014344 (PMC13641719; doi:10.1371/journal.pcbi.1014344)
Supplement: Supplementary file 20 [file pcbi.1014344.s020.pdf]

S11 Table. Overall performance comparison among GeoEPred, DeepSecE, and MoCETSE.

Metric	GeoEPred	DeepSecE	MoCETSE	*p*-value (vs DeepSecE)	*p*-value (vs MoCETSE)
Accuracy	92.31%	89.62%	88.85%	0.098	0.048
Macro Recall	94.33%	87.05%	86.10%	0.018	0.012
Macro AUPRC	0.9146	0.8872	0.8784	0.088	0.042
Macro F1-score	88.43%	86.60%	85.54%	0.182	0.092
Macro AUC	0.9859	0.9832	0.9818	0.145	0.082
Macro Precision	85.79%	86.35%	85.12%	0.620	0.450
