# Supplementary material for: GeoEPred: A multimodal structure-aware geometric deep learning framework for Gram-negative bacterial secreted effector prediction with sequence semantics
Source: PLoS Comput Biol. 2026 Sep 30;22(9):e1014344. doi: 10.1371/journal.pcbi.1014344 (PMC13641719; doi:10.1371/journal.pcbi.1014344)
Supplement: Supplementary file 21 [file pcbi.1014344.s021.pdf]

S12 Table. Class-wise statistical significance analysis of performance differences between GeoEPred and DeepSecE on the independent test set.

Model Variant	Metric	Δ	95% CI	*p*-values
No-effector (n=150)	Precision	+0.0022	[0.0000, 0.0493]	0.0820
	Recall	+0.0133	[-0.0467, 0.0668]	0.7780
	F1	+0.0173	[-0.0168, 0.0518]	0.3280
	AUC	+0.0131	[0.0014, 0.0279]	0.0200
	AUPRC	+0.0115	[0.0014, 0.0253]	0.0180
T1SE (n=20)	Precision	+0.0000	[0.0000, 0.0000]	1.0000
	Recall	+0.0000	[-0.1500, 0.1500]	1.0000
	F1	+0.0000	[-0.0855, 0.0855]	1.0000
	AUC	-0.0306	[-0.1044, 0.0500]	0.6480
	AUPRC	-0.0150	[-0.0957, 0.0407]	0.7720
T2SE (n=10)	Precision	-0.1714	[-0.3462, -0.0557]	0.0040
	Recall	+0.0000	[0.0000, 0.0000]	1.0000
	F1	-0.1394	[-0.2557, -0.0474]	0.0040
	AUC	-0.0160	[-0.0652, 0.0136]	0.7080
	AUPRC	+0.0961	[-0.0738, 0.3794]	0.3100
T3SE (n=30)	Precision	+0.3611	[0.0300, 0.2927]	0.0160
	Recall	+0.0000	[-0.3000, 0.3000]	1.0000
	F1	+0.0679	[-0.0017, 0.1775]	0.0660
	AUC	+0.0079	[-0.0096, 0.0777]	0.4420
	AUPRC	+0.0059	[-0.0350, 0.1031]	0.4460
T4SE (n=150)	Precision	+0.1192	[-0.0286, 0.2580]	0.1380
	Recall	+0.0333	[0.0000, 0.1000]	0.7160
	F1	+0.0846	[-0.0116, 0.3699]	0.0940
	AUC	+0.0020	[-0.0026, 0.0085]	0.4640
	AUPRC	+0.0077	[-0.0245, 0.0424]	0.6740
T6SE (n=20)	Precision	-0.0193	[-0.3642, 0.1265]	0.8960
	Recall	+0.2000	[0.0500, 0.4000]	0.0180
	F1	+0.0681	[-0.0231, 0.2433]	0.1340
	AUC	+0.0096	[0.0012, 0.0233]	0.0200
	AUPRC	+0.0715	[0.0118, 0.3631]	0.0160
