# Supplementary material for: GeoEPred: A multimodal structure-aware geometric deep learning framework for Gram-negative bacterial secreted effector prediction with sequence semantics
Source: PLoS Comput Biol. 2026 Sep 30;22(9):e1014344. doi: 10.1371/journal.pcbi.1014344 (PMC13641719; doi:10.1371/journal.pcbi.1014344)
Supplement: Supplementary file 22 [file pcbi.1014344.s022.pdf]

S13 Table. Class-wise statistical significance analysis of performance differences between GeoEPred and MoCETSE on the independent test set.

Model Variant	Metric	Δ	95% CI	*p*-values
No-effector (n=150)	Precision	+0.0227	[0.0000, 0.0496]	0.0820
	Recall	+0.0333	[-0.0200, 0.0867]	0.2500
	F1	+0.0288	[-0.0012, 0.0603]	0.0800
	AUC	+0.0065	[-0.0007, 0.0162]	0.0720
	AUPRC	+0.0053	[-0.0000, 0.0127]	0.0540
T1SE (n=20)	Precision	+0.0000	[0.0000, 0.0000]	1.0000
	Recall	+0.0000	[-0.1500, 0.1500]	1.0000
	F1	+0.0000	[-0.0855, 0.0855]	1.0000
	AUC	-0.0260	[-0.0908, 0.0017]	0.6500
	AUPRC	-0.0056	[-0.0399, 0.0210]	0.7960
T2SE (n=10)	Precision	-0.0214	[-0.1250, 0.0794]	0.8580
	Recall	+0.0000	[0.0000, 0.0000]	1.0000
	F1	-0.0194	[-0.1042, 0.0686]	0.8580
	AUC	-0.0112	[-0.0556, 0.0172]	0.7780
	AUPRC	+0.1313	[-0.0225, 0.4114]	0.1040
T3SE (n=30)	Precision	+0.0634	[-0.0358, 0.1765]	0.2800
	Recall	+0.0333	[-0.0667, 0.1667]	0.8020
	F1	+0.0486	[-0.0339, 0.1312]	0.2720
	AUC	+0.0090	[-0.0087, 0.0341]	0.4260
	AUPRC	+0.0324	[-0.0258, 0.1044]	0.3080
T4SE (n=150)	Precision	+0.1681	[0.0000, 0.3011]	0.0540
	Recall	+0.0000	[0.0000, 0.0000]	1.0000
	F1	+0.1042	[0.0000, 0.1914]	0.0540
	AUC	-0.0001	[-0.0038, 0.0032]	0.9760
	AUPRC	-0.0035	[-0.0316, 0.0184]	0.8520
T6SE (n=20)	Precision	-0.1304	[-0.2593, 0.0000]	0.1180
	Recall	+0.1500	[0.0000, 0.3000]	0.0720
	F1	+0.0113	[-0.0910, 0.1521]	0.8280
	AUC	+0.0098	[0.0000, 0.0279]	0.0860
	AUPRC	+0.0574	[0.0000, 0.1457]	0.0760
