# Supplementary material for: GeoEPred: A multimodal structure-aware geometric deep learning framework for Gram-negative bacterial secreted effector prediction with sequence semantics
Source: PLoS Comput Biol. 2026 Sep 30;22(9):e1014344. doi: 10.1371/journal.pcbi.1014344 (PMC13641719; doi:10.1371/journal.pcbi.1014344)
Supplement: Supplementary file 23 [file pcbi.1014344.s023.pdf]

S14 Table. Sample-level pLDDT statistics across different benchmark datasets, ranked by the mean pLDDT of the training set.

| Rank | Prediction Task | Dataset | Training Set pLDDT (Mean $\pm$ SD) | Test Set pLDDT (Mean $\pm$ SD) |
|---|---|---|---|---|
| 1 | T6SE | Bastion6 | 86.02 $\pm$ 12.38 | 83.82 $\pm$ 14.04 |
| 2 | T6SE | BastionX T6SE | 85.47 $\pm$ 12.65 | 82.19 $\pm$ 13.03 |
| 3 | T4SE | BastionX T4SE | 85.19 $\pm$ 13.61 | 74.97 $\pm$ 18.25 |
| 4 | T4SE | Bastion4 | 81.71 $\pm$ 16.02 | 84.10 $\pm$ 13.21 |
| 5 | T3SE | Bastion3 | 81.35 $\pm$ 16.45 | 75.75 $\pm$ 17.65 |
| 6 | T4SE | T4SEpp | 80.78 $\pm$ 21.18 | 84.20 $\pm$ 13.88 |
| 7 | T3SE | T3SEpp | 75.54 $\pm$ 17.99 | 74.54 $\pm$ 17.99 |
