# Supplementary material for: GeoEPred: A multimodal structure-aware geometric deep learning framework for Gram-negative bacterial secreted effector prediction with sequence semantics
Source: PLoS Comput Biol. 2026 Sep 30;22(9):e1014344. doi: 10.1371/journal.pcbi.1014344 (PMC13641719; doi:10.1371/journal.pcbi.1014344)
Supplement: Supplementary file 24 [file pcbi.1014344.s024.pdf]

Genome-constrained permutation analysis of effector co-localization

To quantitatively assess the genomic co-localization between predicted effector proteins and their compatible secretion systems, we performed a genome-constrained permutation analysis using the *Pseudomonas aeruginosa* PAO1 proteome. The analysis included all 5,572 protein-coding sequences in the PAO1 genome [1]. Secretion-system loci were annotated from the ordered proteome using MacSyFinder v2.1.6 with the TXSScan v1.1.4 diderm models [2], and GeoEPred-predicted effectors were mapped to their corresponding genomic positions using the same genome annotation. Genomic co-localization was defined according to the number of intervening coding sequences (CDSs) between a predicted effector gene and a compatible secretion-system gene cluster. A window of $k = 10$ CDSs was used for the main analysis, such that an effector was considered co-localized when no more than 10 CDSs separated it from the corresponding secretion-system locus. For each secretion-system class, an empirical null distribution was generated using 10,000 random permutations. In each permutation, the same number of proteins as the observed predicted effectors of that class was sampled without replacement from the eligible PAO1 proteome, while the genomic positions of the secretion-system loci were kept fixed. The number of randomly sampled proteins satisfying the same co-localization criterion was then recorded.
The one-sided empirical p-value was calculated as

$$p_{\text{emp}} = \frac{1 + \sum_{b=1}^{B} I\left(X_b \geq X_{\text{obs}}\right)}{B + 1},$$

where $B = 10{,}000$, X_{obs} denotes the observed number of co-localized predicted effectors, X_b denotes the corresponding number obtained in the b-th permutation, and $I(\cdot)$ is the indicator function [3]. Sensitivity analyses were additionally performed using $k = 0$, 5, 20, and 50 CDS windows to evaluate the dependence of the results on the neighborhood definition. The resulting p-values were adjusted for multiple testing using the Benjamini--Hochberg procedure to control the false discovery rate, yielding the corresponding q-values [4].

S15 Table. Genomic co-localization analysis of predicted effectors and corresponding secretion system gene clusters in *Pseudomonas aeruginosa* PAO1.

type	N_{pred}	k_{cds}	X_{obs}	X_{exp}	Enrichment Fold	p-value	FDR q-value
T1SE	12	≤ 5	5	0.1163	42.9914x	<0.0001	<0.0002
		≤ 10	5	0.2024	24.6986x	<0.0001	<0.0002
		≤ 20	5	0.3747	13.3429x	<0.0001	<0.0002
T2SE	113	≤ 5	3	1.2983	2.3108x	0.1372	0.1747
		≤ 10	3	1.9063	1.5737x	0.2970	0.3305
		≤ 20	5	3.1231	1.6009x	0.2037	0.2377
T3SE	343	≤ 5	23	2.2150	10.3837x	<0.0001	<0.0001
		≤ 10	29	2.8306	10.2452x	<0.0001	<0.0001
		≤ 20	34	4.0628	8.3686x	<0.0001	<0.0001
T6SE	163	≤ 5	14	2.4280	5.7660x	<0.0001	<0.0001
		≤ 10	15	3.3056	4.5377x	<0.0001	<0.0001
		≤ 20	17	5.0590	3.3603x	0.0002	0.0003

Reference

[1] Stover CK, Pham XQ, Erwin AL, et al. Complete genome sequence of *Pseudomonas aeruginosa* PAO1, an opportunistic pathogen. *Nature*. 2000;406:959–964. https://doi.org/10.1038/35023079

[2] Néron B, Denise R, Coluzzi C, et al. MacSyFinder v2: improved modelling and search engine to identify molecular systems in genomes. *Peer Community Journal*. 2023;3:e28. https://doi.org/10.24072/pcjournal.250

[3] Phipson B, Smyth GK. Permutation p-values should never be zero: calculating exact p-values when permutations are randomly drawn. *Statistical Applications in Genetics and Molecular Biology*. 2010;9:Article 39. https://doi.org/10.2202/1544-6115.1585

[4] Benjamini Y, Hochberg Y. Controlling the false discovery rate: a practical and powerful approach to multiple testing. *Journal of the Royal Statistical Society: Series B (Methodological)*. 1995;57:289–300. https://doi.org/10.1111/j.2517-6161.1995.tb02031.x
