# Supplementary material for: GeoEPred: A multimodal structure-aware geometric deep learning framework for Gram-negative bacterial secreted effector prediction with sequence semantics
Source: PLoS Comput Biol. 2026 Sep 30;22(9):e1014344. doi: 10.1371/journal.pcbi.1014344 (PMC13641719; doi:10.1371/journal.pcbi.1014344)
Supplement: Supplementary file 25 [file pcbi.1014344.s025.pdf]

S16 Table. Pfam domain enrichment analysis across candidate effector proteins in three bacterial genomes.

Strain	Class	Pfam ID	Domain description	p_{adjust}
NC_002516.2	T6SE	PF00126	LysR family HTH domain	4.95e-06
	T6SE	PF03466	LysR substrate-binding domain	6.60e-07
	T6SE	PF05954	Phage baseplate hub (GPD)	3.43e-06
	T6SE	PF04717	T6SS/phage baseplate tail tube OB	1.76e-05
	T6SE	PF22178	Phage T4 gp5 trimerisation domain	8.82e-05
NC_002942.5	T6SE	PF16263	Domain of unknown function(DUF4917)	1.47e-02
	T3SE	PF26620	Domain of unknown function(DUF8197)	1.96e-02
NC_003197.2	T4SE	PF08238	Sel1 repeat	2.39e-02
	T6SE	PF05638	T6SS effector protein Hcp	4.21e-02
	T6SE	PF04717	T6SS/phage baseplate OB	4.21e-02
	T6SE	PF02368	Bacterial Ig-like domain	4.21e-02
	T6SE	PF05954	Phage baseplate hub	4.21e-02
	T6SE	PF16461	Lambda phage tail tube protein TTP	4.21e-02
