# Supplementary material for: GeoEPred: A multimodal structure-aware geometric deep learning framework for Gram-negative bacterial secreted effector prediction with sequence semantics
Source: PLoS Comput Biol. 2026 Sep 30;22(9):e1014344. doi: 10.1371/journal.pcbi.1014344 (PMC13641719; doi:10.1371/journal.pcbi.1014344)
Supplement: Supplementary file 26 [file pcbi.1014344.s026.pdf]

S17 Table. Quantitative residue-level attribution enrichment analysis of representative effector proteins using circular shift permutation and hypergeometric tests.

Protein	Branch	Functional region	Residue range	FE	Circular shift permutation *p*	Circular shift BH *q*	Hypergeometric *p*	Hypergeometric BH *q*
SlrP	Sequence	T3SS translocation signal	1–50	6.44×	0.0030	0.0060	7.38×10^{-11}	1.48×10^{-10}
SlrP	Structure	E2 interaction interface	751–765	2.68×	0.0830	0.0830	0.1681	0.1681
Tse5	Sequence	Hcp interaction region	1–35	9.12×	0.0219	0.0873	4.81×10^{-13}	4.33×10^{-12}
Tse5	Structure	Rhs plug/gateway interface	1115–1163	4.07×	0.0011	0.0099	8.72×10^{-5}	3.92×10^{-4}
SidJ	Sequence	T4SS translocation signal	1–50	4.37×	0.0334	0.1336	1.32×10^{-5}	5.27×10^{-5}
SidJ	Sequence	Conserved catalytic motif	348–380	4.56×	0.0250	0.1336	7.00×10^{-8}	8.40×10^{-7}
SidJ	Structure	CaM-binding interface	597–708	1.95×	1.00×10^{-4}	0.0012	0.0179	0.0430
