# Supplementary material for: GeoEPred: A multimodal structure-aware geometric deep learning framework for Gram-negative bacterial secreted effector prediction with sequence semantics
Source: PLoS Comput Biol. 2026 Sep 30;22(9):e1014344. doi: 10.1371/journal.pcbi.1014344 (PMC13641719; doi:10.1371/journal.pcbi.1014344)
Supplement: Supplementary file 27 [file pcbi.1014344.s027.pdf]

S18 Table. GeoEPred predictions for recently experimentally validated bacterial effector proteins.

Effector	Strain	Secretion system	GeoEPred prediction	Confidence	Reference
AopI	*Aeromonas schubertii* ATCC 43700	T3SE	T3SE	0.983	[1]
AopJ	*Aeromonas schubertii* ATCC 43700	T3SE	T3SE	0.975	[1]
AopL	*Aeromonas schubertii* ATCC 43700	T3SE	T3SE	0.984	[1]
AopT	*Aeromonas schubertii* ATCC 43700	T3SE	T3SE	0.994	[1]
AopU	*Aeromonas schubertii* ATCC 43700	T3SE	T3SE	0.994	[1]
TseI-like	*Aeromonas jandaei* DSM 7311	T6SE	T6SE	0.984	[2]
Tle1-like	*Aeromonas jandaei* DSM 7311	T6SE	T6SE	0.996	[2]
Awe1	*Aeromonas jandaei* DSM 7311	T6SE	T6SE	0.990	[2]
DUF3289	*Aeromonas jandaei* DSM 7311	T6SE	T6SE	0.989	[2]
XopBB	*X. hortorum* pv. *pelargonii* 305	T3SE	T3SE	0.994	[3]
XopBC	*X. hortorum* pv. *pelargonii* 305	T3SE	T3SE	0.990	[3]
XopBD	*X. hortorum* pv. *pelargonii* 305	T3SE	T3SE	0.984	[3]
XopBE	*X. hortorum* pv. *pelargonii* 305	T3SE	Non-Effector	0.982	[3]
Tse1	*A. veronii* C4	T6SE	T6SE	0.993	[4]
Tse2	*A. veronii* C4	T6SE	T6SE	0.991	[4]
Tse3	*A. veronii* C4	T6SE	T6SE	0.970	[4]

Reference

[1] Michova H, Pliva J, Jirsova A, Jurnecka D, Kamanova J. Cytotoxicity induced by *Aeromonas schubertii* is orchestrated by a unique set of type III secretion system effectors. *Veterinary Research*. 2025;56:113. https://doi.org/10.1186/s13567-025-01548-2

[2] Fridman CM, Keppel K, Rudenko V, et al. A new class of type VI secretion system effectors can carry two toxic domains and are recognized through the WHIX motif for export. *PLOS Biology*. 2025;23:e3003053. https://doi.org/10.1371/journal.pbio.3003053

[3] Wagner N, Ben-Meir D, Teper D, Pupko T. Complete genome sequence of an Israeli isolate of *Xanthomonas hortorum* pv. *pelargonii* strain 305 and novel type III effectors identified in *Xanthomonas*. *Frontiers in Plant Science*. 2023;14:1155341. https://doi.org/10.3389/fpls.2023.1155341

[4] Jiang X, Li H, Ma J, et al. Role of type VI secretion system in pathogenic remodeling of host gut microbiota during *Aeromonas veronii* infection. *The ISME Journal*. 2024;18:wrae053. https://doi.org/10.1093/ismejo/wrae053
