# Supplementary material for: GeoEPred: A multimodal structure-aware geometric deep learning framework for Gram-negative bacterial secreted effector prediction with sequence semantics
Source: PLoS Comput Biol. 2026 Sep 30;22(9):e1014344. doi: 10.1371/journal.pcbi.1014344 (PMC13641719; doi:10.1371/journal.pcbi.1014344)
Supplement: Supplementary file 28 [file pcbi.1014344.s029.pdf]

Response to the Editor and Reviewers

Submission ID: PCOMPBIOL-D-26-01167

Manuscript Title: "GeoEPred: A Multimodal Structure-Aware Geometric Deep Learning Framework for Gram-Negative Bacterial Secreted Effector Prediction with Sequence Semantics"

Dear Editor and Reviewers,

We would like to express our sincerest gratitude to the Editor and all the Reviewers for devoting their valuable time and effort to evaluating our manuscript and for providing detailed, insightful, and constructive comments. These valuable comments helped us identify aspects of the original manuscript that required further clarification and improvement, and prompted us to undertake a more thorough and comprehensive revision. As a result, the scientific rigor, clarity of presentation, and overall quality of the manuscript have been further strengthened.

We carefully considered every comment and revised the manuscript accordingly. In the following response, all comments are numbered and addressed point by point, together with detailed explanations of the corresponding revisions and their rationale. For ease of reference, the reviewers' comments are presented in black italics, our responses are shown in blue, and the corresponding changes in the revised manuscript are highlighted in red. We have also specified the relevant page and line numbers for each revision. In addition, we updated and redrew the figures in accordance with the revised content and standardized the font sizes across all figures.

Once again, we sincerely thank the Editor and the Reviewers for their careful evaluation, insightful comments, and constructive suggestions. We hope that the revisions and additional analyses have adequately addressed all the comments and concerns and the revised manuscript is now suitable for publication in PLOS Computational Biology.

Kind regards,

Hua Shi

On behalf of all authors

Reviewer #1:

This manuscript proposes GeoEPred, a multimodal deep learning framework for Gram-negative bacterial effector prediction by combining protein language model embeddings with structure-aware geometric learning. The manuscript is generally well organized and supported by extensive experiments. However, several concerns should be addressed to strengthen the scientific rigor and reliability of the study before accepting it for publication. The major comments are listed below.

1. The manuscript does not clearly demonstrate that the training, validation, and independent benchmark datasets are completely non-overlapping. The authors should provide evidence of redundancy removal across datasets and ensure that no homologous proteins are shared between training and test sets.

Response: We thank the reviewer for raising this important concern. We fully agree that the original manuscript did not provide a sufficiently clear description of the sequence redundancy removal procedure or adequate supporting evidence regarding the independence of the training, validation, and independent test sets. In response to the reviewer's comment, we thoroughly re-examined all dataset partitions and have added the complete data-processing workflow, parameter settings, and verification results to the revised manuscript and Supplementary Materials.

To minimize the potential overestimation of model performance caused by sequence redundancy, we followed the dataset construction strategy adopted in DeepSecE [1] and used CD-HIT v4.8.1 to remove redundancy separately within the candidate training set and the independent test set. Specifically, we applied a sequence identity threshold of 60% using the global sequence identity criterion. In addition, we required the aligned region to cover at least 80% of both the shorter and longer sequences. Within each dataset, sequences from all effector classes and the non-effector class were pooled for redundancy removal, and only one representative sequence was retained from each cluster. This procedure prevented identical or highly similar protein sequences from being represented multiple times within the datasets.

After removing redundancy within the candidate training set and the independent test set, we further performed a cross-dataset comparison using CD-HIT-2D with the same criteria. The non-redundant independent test set was used as the reference dataset, whereas the candidate training set was used as the dataset to be filtered. Any training sequence sharing ≥60% sequence identity with a sequence in the independent test set, with the aligned region covering at least 80% of both the shorter and longer sequences, was removed from the candidate training set. During this cross-dataset filtering step, the independent test set was kept unchanged, and only highly similar

sequences that could potentially cause information leakage were removed from the training set.

After the above procedures, the final training set contained 3,155 sequences, whereas the independent test set contained 260 sequences. To further verify the independence of these datasets, we reran CD-HIT-2D to compare the final training set against the independent test set. No cross-dataset sequence pairs satisfying the predefined sequence identity and alignment coverage criteria were detected (n = 0). Therefore, the final training and independent test sets contained neither identical protein sequences nor highly similar sequence pairs satisfying the predefined criteria.

The validation sets used in this study were generated from the final non-redundant training set through stratified five-fold cross-validation. Because five-fold partitioning was performed only after redundancy removal from the training set at the 60% sequence identity threshold, the training and validation subsets within each fold likewise contained no highly similar sequence pairs satisfying the above criteria. Moreover, across repeated experiments using different random seeds, we kept the five-fold data partitions fixed and varied only model parameter initialization, minibatch ordering, and other stochastic aspects of the training process. This design enabled us to assess the stability of the model while controlling for variation arising from different dataset partitions.

We have added these details to the dataset construction section of the revised manuscript. S1 Fig now reports the number of sequences in each class before and after redundancy removal, together with the results of the cross-dataset redundancy assessment. To enhance the transparency and reproducibility of this study, we have made the processed datasets and the relevant files generated during data processing publicly available at:

https://github.com/shouzhensong/GeoEPred.

We thank the reviewer again for this important comment. These additional analyses and documentation have enabled us to more rigorously demonstrate dataset independence and substantially improve the transparency and reproducibility of the study.

Reference:

[1] Zhang Y, Guan J, Li C, Wang Z, Deng Z, Gasser RB, Song J, Ou HY. DeepSecE: A deep-learning-based framework for multiclass prediction of secreted proteins in Gram-negative bacteria. Research (Wash D C). 2023;6:0258. doi:10.34133/research.0258.

The following content provides supplementary additions to the main text.

1) Corresponding revisions have been made in the "Dataset construction" section (Page 4, Lines 59–88).

The datasets used for model training and evaluation were compiled from datasets released with established effector prediction methods and from widely used biological databases.

The dataset construction and redundancy-removal procedures were designed with reference to DeepSecE [13], a representative study in the field. Specifically, T1SE and T2SE sequences were obtained from the TSE-ARF dataset [21]. The T3SE and T6SE datasets were compiled by integrating sequences from Bastion3 [7], DeepT3 [22], SecReT6 [23], Bastion6 [8], DeepT3_4 [24], and TSE-ARF [21]. The T4SE dataset was assembled from DeepSecE [13], OPT4e [25], T4SEfinder [26], iT4SE-EP [27], Bastion4 [28], T4SE-XGB [29], TSE-ARF [21], T4SEpp [30], and DeepT3_4 [24]. Following previous representative studies [31, 32], the negative class was constructed using curated non-secreted proteins and homologous sequences from selected pathogenic bacteria reported in earlier studies. These candidate negative samples had been further screened against multiple bacterial genomes using BLASTp in the original studies, based on predefined thresholds for the *E*-value and sequence identity. To provide an objective assessment of model generalization, we additionally assembled an independent test set. The T3SE, T4SE, T6SE, and non-secreted protein samples were collected from the test datasets released with Bastion3, CNN-T4SE [33], and Bastion6, whereas the T1SE and T2SE samples were randomly selected from the original BastionHub dataset.

To minimize potential overestimation of model performance caused by sequence redundancy, CD-HIT v4.8.1 [33] was applied separately to the candidate training set and the independent test set. A global sequence identity threshold of 60% was used, and the aligned region was required to cover at least 80% of both the shorter and longer sequences. Within each dataset, sequences from all effector and non-effector classes were pooled before clustering, and only one representative sequence was retained from each cluster. Cross-dataset filtering was subsequently performed using CD-HIT-2D under the same identity and coverage criteria. The non-redundant independent test set was used as the reference dataset, and candidate training sequences showing at least 60% global sequence identity to any test sequence were removed. This procedure minimized the risk of information leakage between the training and test sets.

The data sources for each effector type and the corresponding sample numbers after CD-HIT filtering are summarized in S1 Table. The resulting training set comprised 1,575 non-effector proteins, 128 T1SEs, 65 T2SEs, 505 T3SEs, 623 T4SEs, and 259 T6SEs. The independent test set contained 150 non-effector proteins, 20 T1SEs, 10 T2SEs, 30 T3SEs, 30 T4SEs, and 20 T6SEs (S1 Fig A). Protein lengths

ranged from fewer than 100 to more than 1,000 amino acids, with most sequences falling between 100 and 700 amino acids (S1 Fig B).

2. The study relies heavily on ESMFold-predicted structures. The authors should assess how structural confidence (e.g., pLDDT scores) influences model performance and validate the findings using experimentally resolved protein structures if available.

Response: We sincerely thank the reviewer for this important and constructive comment. We fully agree that, because our model relies on structures predicted by ESMFold, it is essential to assess whether structural confidence affects model performance. Following the reviewer's suggestion, we performed additional analyses to systematically investigate the relationship between ESMFold structural confidence and predictive performance, and we also examined the availability of experimentally resolved structures for the proteins included in our datasets.

Specifically, we systematically evaluated the impact of structural confidence, as estimated by ESMFold, on model performance. We first analyzed the distributions of pLDDT scores in the training and test sets, which supported the overall suitability of the ESMFold-predicted structures for subsequent structural feature extraction (Table 1). We then stratified the proteins in the independent test set into four groups according to structural confidence: *very low confidence* (pLDDT < 50), *low confidence* (50 ≤ pLDDT < 70), *high confidence* (70 ≤ pLDDT < 90), and *very high confidence* (pLDDT ≥ 90). With all model weights and hyperparameters kept unchanged, we evaluated key performance metrics, including ACC, macro-F1, and MCC, within each group. As shown in Fig.7A, the classification accuracies of the four groups were 81.82%, 87.50%, 88.89%, and 96.47%, respectively, indicating a clear monotonic increase in accuracy with increasing structural confidence.

To further quantify this continuous relationship, we divided the 260 test proteins into 10 approximately equal-sized pLDDT bins, each containing 26 proteins. The data points in Fig.7B represent the mean pLDDT and observed accuracy for each bin, whereas the fitted curve and corresponding 95% confidence interval were derived from a protein-level logistic regression model using the binary classification outcome (correct vs. incorrect) for all 260 proteins. The analysis showed that the probability of correct classification increased with increasing mean pLDDT, although the wider confidence intervals in the low-pLDDT region indicate greater uncertainty in this range. After adjustment for covariates including the true class label and protein length, each 10-unit increase in mean pLDDT was associated with a 79.8% increase in the odds of correct classification (OR = 1.798, 95% CI: 1.242-2.602, p = 0.002). Consistent with this finding, correctly classified proteins exhibited a higher mean

pLDDT than misclassified proteins (79.88 vs. 71.54; Mann-Whitney U = 3522.0, p = 0.029); BH-FDR-adjusted (q = 0.044; Cliff's δ = 0.271). Together, these results support a positive association between structural confidence and predictive accuracy. Importantly, despite this association, the model retained reasonable predictive robustness for proteins with lower structural confidence: the accuracy remained 81.82% in the very-low-confidence group.

We further investigated the agreement between ESMFold-predicted structures and experimentally resolved protein structures. Because the benchmark datasets used in this study primarily provide protein sequences and class labels without consistent database accession numbers, we performed sequence-based mapping against protein structures available in the RCSB Protein Data Bank (PDB). To ensure reliable correspondence, only matches with 100% sequence identity, 100% query and target sequence coverage, and identical sequence lengths were retained, yielding 57 proteins with exact full-length sequence matches to PDB entries. We further selected proteins for which experimentally resolved coordinates covered at least 90% of the corresponding full-length sequence, resulting in 29 matched protein pairs for structural comparison. For these proteins, the ESMFold-predicted structures were aligned with the corresponding experimentally resolved structures using US-align. Structural agreement was evaluated using the TM-score normalized to the experimentally resolved structure and the root-mean-square deviation (RMSD). Mean pLDDT showed a strong positive correlation with TM-score (Spearman's ρ = 0.922, $p = 1.30 \times 10^{-12}$) and a strong negative correlation with RMSD ($\rho = -0.905$, $p = 1.53 \times 10^{-11}$). The median TM-score was 0.921, and the median RMSD was 1.63 Å (S5 Fig). These results provide additional structure-level evidence that higher ESMFold confidence is associated with greater agreement between predicted and experimentally resolved structures, supporting the use of pLDDT as an indicator of structural reliability in our analysis. The corresponding findings and the current availability of matched experimentally resolved structures have instead been discussed in the revised Discussion section, together with the value of further validation using larger experimentally characterized structure sets.

We have made the corresponding revisions in the revised manuscript. We sincerely thank the reviewer again for this important suggestion. This comment highlighted the important role of structural validation in assessing model reliability and prompted us to further strengthen our analysis of predicted structural confidence and its relationship with model performance, thereby providing a more comprehensive and rigorous understanding of the reliability and applicability of the structural representations used by our model.

The following content provides supplementary additions to the main text.

1) We have supplemented the "Protein Language Model" subsection with an evaluation of the pLDDT scores for all protein structures in the training and test sets(Page 5, Lines 123–135).

Building on these advances, we incorporated an ESMFold-based structural branch into our model. Three-dimensional structures predicted directly from protein sequences were used as structural inputs to extract geometric and conformational features, enabling the model to characterize proteins from a spatial perspective. To assess the reliability of the structures predicted by ESMFold, we calculated the per-residue predicted Local Distance Difference Test (pLDDT) scores for all protein structures in the training and test sets. The pLDDT score ranges from 0 to 100 and provides a residue-level estimate of local structural confidence, with higher scores indicating more reliable predictions. Summary statistics for the structural confidence of the two datasets are presented in Table 1. The mean pLDDT score was approximately 77 for both the training and test sets, indicating that the predicted structures generally exhibited good confidence. Moreover, the pLDDT distributions were comparable between the two datasets, suggesting no evident shift in structural quality between the training and test sets. Approximately 40% of the residues fell within the high-confidence range (pLDDT ≥ 90), indicating that a substantial proportion of the predicted structures provided a reliable structural basis for subsequent geometric feature extraction and protein classification.

2) We have added Table 1 to clearly and systematically present the predicted structural quality of GeoEPred on both the training and test sets, thereby further assessing the reliability of the model-generated structures.

Table 1. Quality of ESMFold-Predicted Structures and Their pLDDT Distributions.

Metric	Training Set	Testing Set
pLDDT (Mean ± SD)	76.67 ± 17.65	78.57 ± 17.80
Median pLDDT	82.43	84.36
pLDDT ≥ 90 (High Confidence)	40.42%	44.17%
70 ≤ pLDDT ≤ 90 (Medium Confidence)	26.41%	24.33%
pLDDT < 70 (Low Confidence)	26.64%	23.90%

3) A new subsection titled "Influence of ESMFold structural confidence on model performance" has been added to the "Results" section (Page 15-16, Lines 471–502).

Because our model uses ESMFold-predicted structures as inputs to the structural branch, we further investigated the association between predicted structural confidence and effector protein classification performance.

Based on the mean protein-level pLDDT score, the independent test set was

divided using fixed thresholds into four confidence groups: *Very low* (pLDDT < 50), *Low* (50 ≤ pLDDT < 70), *Confident* (70 ≤ pLDDT< 90), and *Very high* (pLDDT ≥ 90). As shown in Fig.7A, the corresponding accuracies were 81.82%, 87.50%, 88.89%, and 96.47%, respectively, exhibiting an overall increase with structural confidence.

To quantify this association on a continuous scale, the 260 test proteins were further divided into 10 approximately equal-sized pLDDT bins, each containing 26 proteins. The points in Fig.7B represent the mean pLDDT and observed accuracy within each bin, whereas the fitted curve and its 95% confidence band were estimated from protein-level binary correctness outcomes for all 260 samples using logistic regression. The probability of correct classification increased with mean pLDDT, although the wider confidence band in the low-pLDDT region indicated greater estimation uncertainty. After adjusting for the true class label and aligned protein length, each 10-point increase in mean pLDDT was associated with a 79.8% increase in the odds of correct classification (OR = 1.798, 95% CI: 1.242-2.602, p = 0.002). Consistently, correctly classified proteins had a higher mean pLDDT than incorrectly classified proteins (79.88 vs 71.54; Mann--Whitney U = 3522.0, p = 0.029, BH--FDR-adjusted q = 0.044, Cliff's δ=0.271). Together, these results demonstrate a statistically significant positive association between predicted structural confidence and classification performance.

As a supplementary structural validation, we further compared ESMFold-predicted structures with experimentally resolved PDB structures for the subset of 29 proteins meeting stringent sequence-matching and structural-coverage criteria. Mean pLDDT was strongly positively correlated with TM-score (Spearman's ρ = 0.922) and negatively correlated with RMSD (ρ = −0.905), providing preliminary structure-level support for the use of pLDDT as an indicator of structural reliability (S5 Fig). Given the limited number of matched structures, this analysis was considered supportive rather than definitive evidence.

Despite this association, the model retained good predictive performance for lower-confidence structures: the accuracy was 81.82% in the *Very low* group and approximately 84% in the lowest-pLDDT quantile bin. Together with the performance degradation observed after removing the sequence branch in the ablation study, these findings support the complementary roles of sequence and structural information. In particular, the sequence branch may provide additional discriminative cues when structural confidence is limited, thereby improving the model's robustness to uncertainty in predicted structures.

4) We have added Fig.7 to investigate how structural confidence, quantified by pLDDT scores, influences model performance, providing further insights into the

relationship between predicted structural reliability and predictive capability.

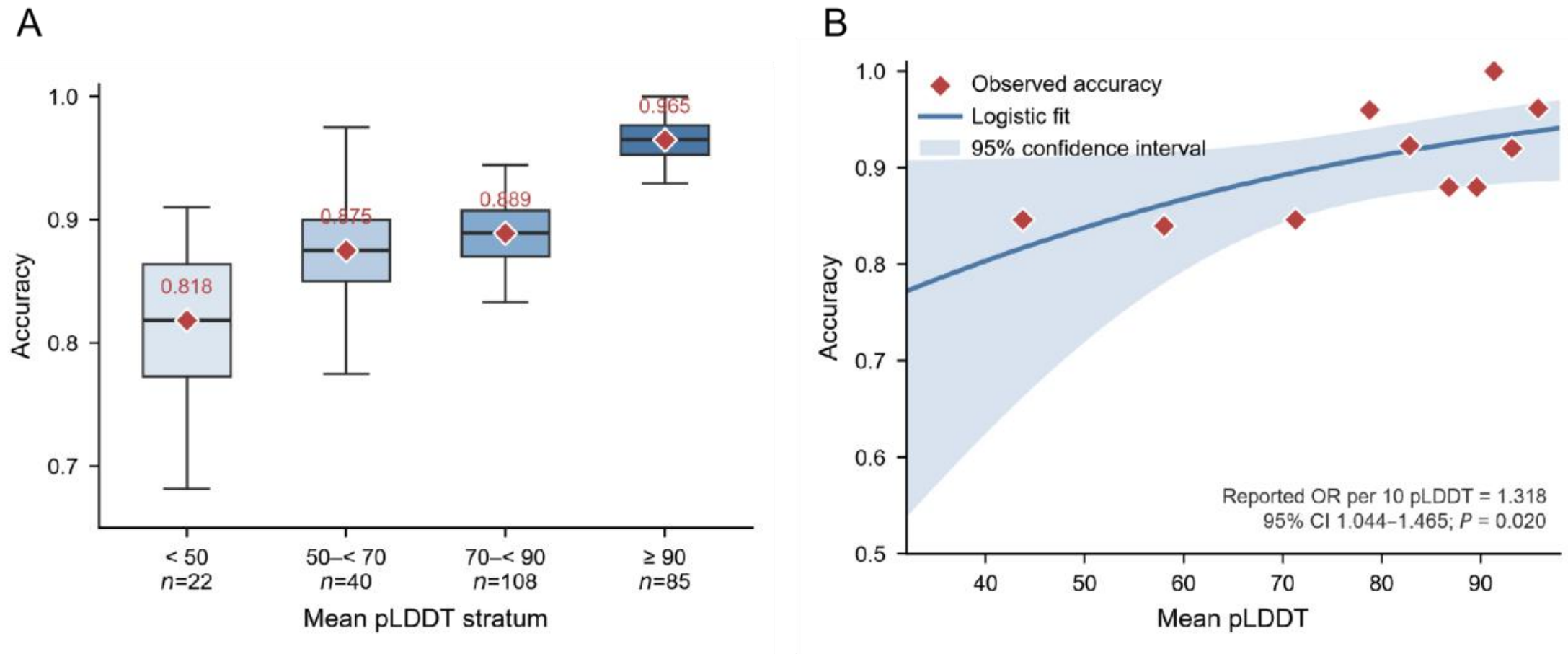

Fig 7. Model performance across ESMFold confidence levels. A: Classification accuracy across four structural-confidence groups defined by the mean protein-level pLDDT: *Very low* (<50), *Low* (50-<70), *Confident* (70-<90), and *Very high* (≥90). Error bars indicate 95% confidence intervals. B: Continuous association between mean pLDDT and classification accuracy. The 260 test proteins were divided into 10 approximately equal-sized bins, each containing 26 proteins. Black points represent the mean pLDDT and observed accuracy within each bin. The red dashed curve and gray shaded 95% confidence band were estimated by logistic regression using the protein-level correct/incorrect outcomes of all 260 test samples rather than the binned observations. After adjustment for the true class label and aligned protein length, each 10-point increase in mean pLDDT was associated with higher odds of correct classification (OR = 1.798, 95% CI: 1.242-2.602, p = 0.002).

5) To clearly visualize these structure-level validation results, we have added S5 Fig to the revised Supplementary Materials, which presents the relationships of mean pLDDT with TM-score and RMSD, together with a comparison of TM-scores between low- and high-confidence ESMFold predictions.

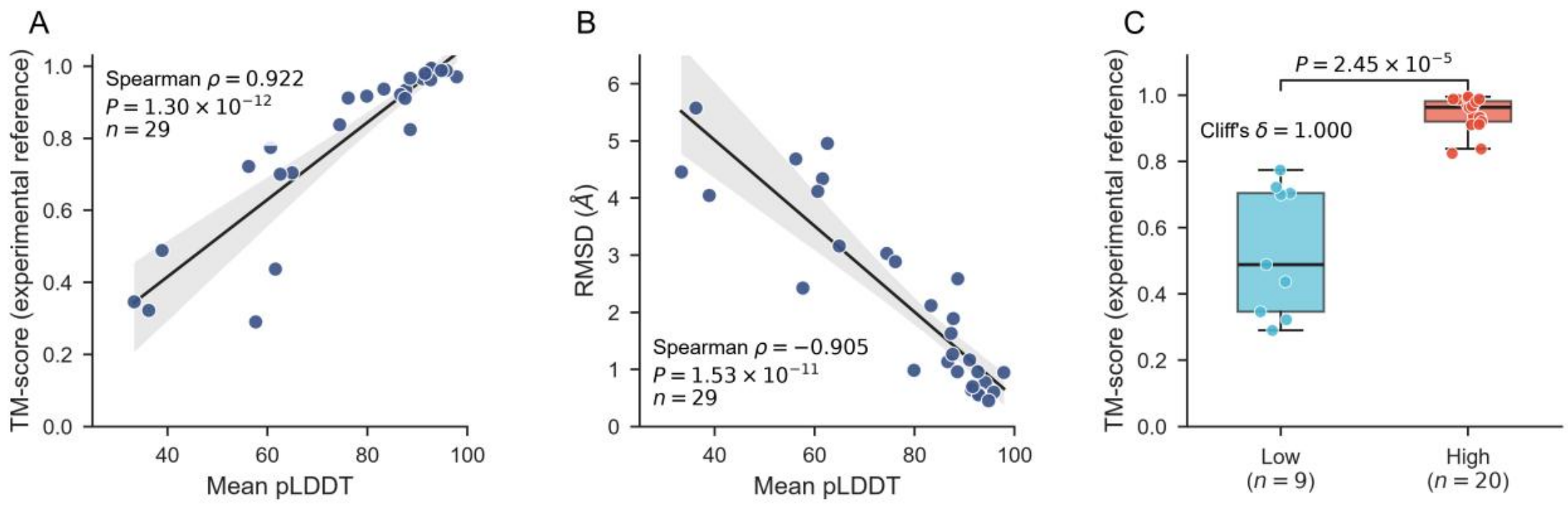

S5 Fig. Comparison of ESMFold-predicted structures with experimentally resolved protein structures. A: Mean pLDDT versus TM-score. B: Mean pLDDT versus RMSD. C: Comparison of TM-scores between low-confidence (pLDDT<70) and high-confidence (pLDDT≥70) predictions. Each point represents one protein;

statistical results are shown in the figure.

3. The computational complexity of the proposed framework is not discussed. The authors should report training time, inference speed, GPU requirements etc. for genome-wide applications.

Response: We sincerely thank the reviewer for this important comment. We agree that computational efficiency is a critical factor for the practical application of this framework. In response to the reviewer's suggestion, we have added an analysis of training time, inference speed, and GPU requirements to the "Case Study" section of the revised manuscript.

Specifically, to further evaluate the model's operational efficiency in real-world scenarios, we retrained our model on a single NVIDIA RTX 4090 GPU. The hardware and software configuration included an AMD EPYC 7543 32-Core processor, 62 GB of system memory, CUDA 12.8, PyTorch 2.8.0, and a batch size of 16. The training process took approximately 0.38 hours for a single fold and approximately 1.44 hours for the complete five-fold training. Peak GPU memory usage during training was 23.86 GB. We then tested the execution time of the trained GeoEPred model using data subsets ranging from 1000 to 5,572 proteins, derived from the whole-genome datasets of *Pseudomonas aeruginosa PAO1*, *Salmonella enterica subsp. enterica serovar Typhimurium str. LT2*, and *Legionella pneumophila subsp. pneumophila str. Philadelphia 1* (see S8 Fig). During the inference phase, with a batch size of 64, the trained model processed proteins at an average rate of 153.38 proteins/second (6.52 ms/protein), with a peak GPU memory usage of 11.48 GB. GeoEPred demonstrated stable performance across all subset sizes, taking approximately 0.2 seconds for a subset of 100 proteins and approximately 36.3 seconds for the full set of 5,572 proteins.

To avoid conflating the computational cost of the proposed classifier with that of the upstream feature generation process, we measured these two stages separately. The process of generating ESMFold structures requires approximately 6.52 seconds per protein, while generating ESM embedding vectors requires approximately 6.2 seconds per protein. In contrast, making predictions using the precomputed features takes only 36.3 seconds. Since the extracted features can be cached and reused, the feature generation step for each protein needs to be performed only once. These results demonstrate that the proposed framework can be trained and deployed using a single modern GPU and that its prediction phase is computationally feasible for genome-scale applications. We have revised the manuscript accordingly and included details regarding runtime, throughput, peak GPU memory usage, and hardware configuration to facilitate reproducibility and practical assessment.

We once again extend our sincere gratitude to the reviewers. Your constructive feedback not only prompted us to significantly refine the methodological details but also enabled us to demonstrate—through robust data—the framework's potential for practical application in genome-wide screening, thereby substantially enhancing the study's practical value.

The following content provides supplementary additions to the main text.

1) We have expanded the "Case Study" section in the revised manuscript to include additional information regarding the computational requirements of genome-wide applications, including model training time, inference speed, and GPU resource consumption. (Page 17, Lines 560–569)

Finally, to evaluate the computational efficiency of GeoEPred for genome-scale applications, we retrained the model on a single NVIDIA RTX 4090 GPU and measured its runtime on protein subsets of different sizes (S8 Fig). Training required approximately 0.38 h for a single fold and approximately 1.44 h for the complete five-fold training, with a peak GPU memory usage of 23.86 GB. During inference, using a batch size of 64, GeoEPred processed proteins at an average rate of 153.38 proteins/s (6.52 ms/protein), with a peak GPU memory usage of 11.48 GB. GeoEPred maintained relatively stable computational performance across different dataset sizes, requiring approximately 6.52 s to process a subset of 1,000 proteins and approximately 36.3 s to process all 5,572 proteins in *Pseudomonas aeruginosa PAO1*. These results indicate that GeoEPred can be trained and deployed on a single high-end GPU and provides practical computational efficiency for bacterial genome-scale screening.

2) We have added S8 Fig to provide a clear evaluation of the relationship between protein dataset size and model inference time, demonstrating the computational efficiency and scalability of GeoEPred for protein datasets of different scales.

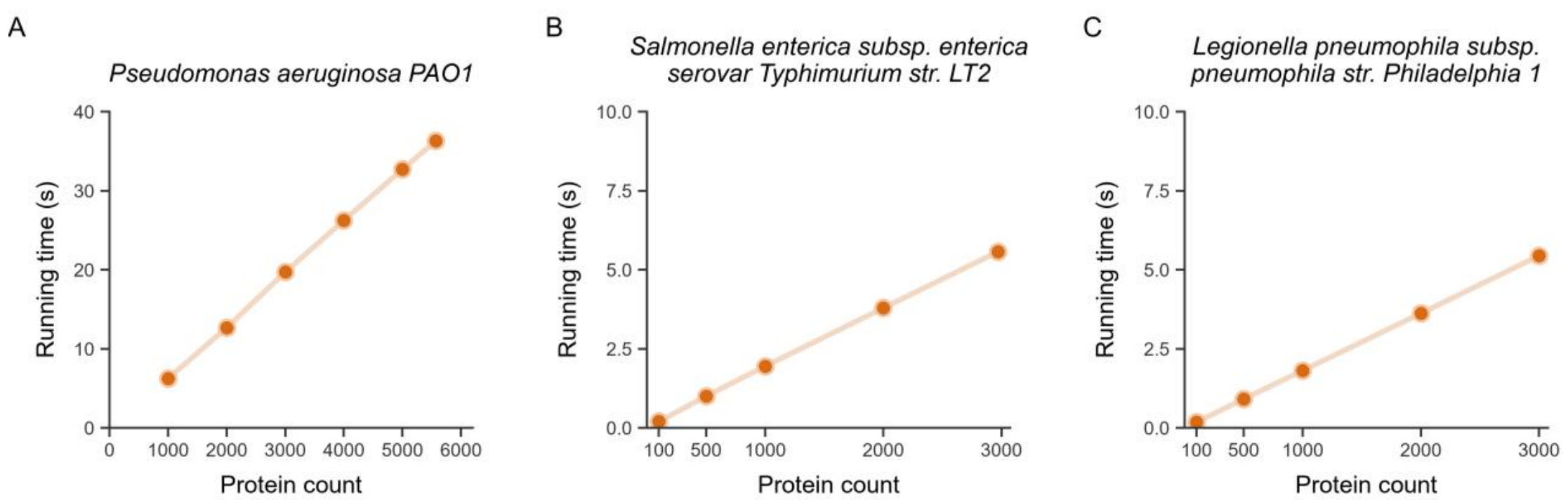

S8 Fig. Model inference time scaling with protein dataset sizes. Evaluation of the model's running time across different protein counts for three bacterial strains. A:

Pseudomonas aeruginosa PAO1, B: *Salmonella enterica subsp. enterica serovar Typhimurium str. LT2*, and C: *Legionella pneumophila subsp. pneumophila str. Philadelphia 1*. The orange dots and line represent the linear relationship between the running time (in seconds) and the number of processed proteins, demonstrating robust processing efficiency.

4. Statistical significance tests should be performed to demonstrate that the observed performance gains are meaningful.

Response: We sincerely thank the reviewer for this important and rigorous comment. We fully agree that systematic statistical significance testing is essential for assessing whether the observed performance improvements are statistically meaningful and robust. Following the reviewer's suggestion, we conducted additional statistical significance analyses to compare, on a class-by-class basis, the performance differences between GeoEPred and the two best-performing baseline models in the benchmark evaluation. In addition, we performed corresponding significance tests for the ablation experiments involving the internal modules and different modalities of our model (please refer to our detailed response to Comment 8).

Specifically, to ensure a fair comparison, we retrained GeoEPred using the same training set as the two best-performing baseline models with publicly available implementations, namely DeepSecE and MoCETSE, and evaluated all models on the same benchmark test set. For each model, we obtained the predicted probabilities, predicted labels, and ground-truth labels for all samples in the independent test set. To assess whether the performance improvements of our method over the baseline models were statistically significant, we performed paired stratified bootstrap analysis with 1,000 resampling iterations and estimated the 95% confidence intervals (95% CIs) and corresponding p-values for differences in both overall and class-specific performance metrics. The corresponding statistical results, including class-specific performance comparisons, confidence intervals, and p-values, have been incorporated into the revised manuscript.

As shown in S11 Table, GeoEPred demonstrated highly competitive and overall superior predictive performance compared with the current state-of-the-art methods across the evaluated tasks. In terms of overall accuracy, GeoEPred improved upon MoCETSE by 3.46% (p = 0.048), indicating a consistent overall performance advantage. At the class level, GeoEPred showed a particularly notable advantage in T6SE prediction. Compared with DeepSecE, GeoEPred achieved significant improvements in both threshold-independent metrics, AUC and AUPRC (AUC: 95% CI: 0.0012–0.0233, p = 0.020; AUPRC: 95% CI: 0.0118–0.3631, p = 0.016). GeoEPred also achieved higher AUC and AUPRC than MoCETSE, with

improvements of Δ = +0.0098 (95% CI: 0.0000–0.0279, p = 0.086) and Δ = +0.0574 (95% CI: 0.0000–0.1457, p = 0.076), respectively. Although these differences showed only marginal statistical significance, together with the perfect identification of all T6SE samples in the independent test set, these results provide strong support for the robust discriminative performance of GeoEPred in T6SE prediction. Based on these statistical analyses, we revised the corresponding statements in the manuscript to more accurately characterize the performance advantages of GeoEPred while maintaining an objective interpretation of the observed differences.

We thank the reviewer again for this rigorous comment. These additional analyses have enabled us to more rigorously distinguish statistically supported improvements from numerical performance differences and have been incorporated into the revised manuscript.

The following content provides supplementary additions to the main text.

1) We have added statistical significance analysis to the "Comparison with State-of-the-Art Models" section of the revised manuscript to further validate the reliability of performance differences among different models. (Page 14, Lines 441–452)

From the overall results, GeoEPred achieved predictive performance comparable to or better than that of current state-of-the-art methods, with a more evident advantage in the T6SE prediction task (Fig. 6A–C, S10 Table). As shown in S11 Table, compared with MoCETSE, the second-best-performing method, GeoEPred achieved better performance across most evaluation metrics, particularly improving AUPRC, which is more suitable for class-imbalanced settings, by 3.62% (p = 0.042). Further analysis of the T6SE class (S12 Table) showed that GeoEPred exhibited strong discriminative ability. Both threshold-independent metrics, AUC and AUPRC, were significantly higher than those of DeepSecE (AUC: 95% CI: 0.0012–0.0233, p = 0.020; AUPRC: 95% CI: 0.0118–0.3631, p = 0.016). Compared with MoCETSE (S13 Table), GeoEPred also showed moderate improvements in both AUC and AUPRC (AUC: Δ = +0.0098, 95% CI: 0.0000–0.0279, p = 0.086; AUPRC: Δ = +0.0574, 95% CI: 0.0000–0.1457, p = 0.076). Although these differences were only marginally significant, the confusion matrix showed that GeoEPred correctly identified all T6SE samples in the test set (Fig. 6D), further demonstrating its good predictive performance in T6SE identification.

2) Newly added S11 Table.

S11 Table. Overall Performance Comparison among GeoEPred, DeepSecE, and MoCETSE.

Metric	GeoEPred	DeepSecE	MoCETSE	p -value (vs DeepSecE)	p -value (vs MoCETSE)
Accuracy	0.923	0.896	0.889	0.098	0.048
Macro Recall	0.943	0.871	0.861	0.018	0.012
Macro AUPRC	0.915	0.887	0.878	0.088	0.042
Macro F1-score	0.884	0.866	0.855	0.182	0.092
Macro AUC	0.986	0.983	0.982	0.145	0.082
Macro Precision	0.858	0.864	0.851	0.620	0.450

3) Newly added S12 Table.

S12 Table. Class-wise statistical significance analysis of performance differences between GeoEPred and DeepSecE on the independent test set.

Model Variant	Metric	Δ	95% CI	p-values
No-effector (n=150)	Precision	+0.0022	[0.0000, 0.0493]	0.0820
	Recall	+0.0133	[-0.0467, 0.0668]	0.7780
	F1	+0.0173	[-0.0168, 0.0518]	0.3280
	AUC	**+0.0131**	**[0.0014, 0.0279]**	**0.0200**
	AUPRC	**+0.0115**	**[0.0014, 0.0253]**	**0.0180**
T1SE (n=20)	Precision	+0.0000	[0.0000, 0.0000]	1.0000
	Recall	+0.0000	[-0.1500, 0.1500]	1.0000
	F1	+0.0000	[-0.0855, 0.0855]	1.0000
	AUC	-0.0306	[-0.1044, 0.0500]	0.6480
	AUPRC	-0.0150	[-0.0957, 0.0407]	0.7720
T2SE (n=10)	Precision	**-0.1714**	**[-0.3462, -0.0557]**	**0.0040**
	Recall	+0.0000	[0.0000, 0.0000]	1.0000
	F1	**-0.1394**	**[-0.2557, -0.0474]**	**0.0040**
	AUC	-0.0160	[-0.0652, 0.0136]	0.7080
	AUPRC	+0.0961	[-0.0738, 0.3794]	0.3100
T3SE (n=30)	Precision	**+0.3611**	**[0.0300, 0.2927]**	**0.0160**
	Recall	+0.0000	[-0.3000, 0.3000]	1.0000
	F1	+0.0679	[-0.0017, 0.1775]	0.0660
	AUC	+0.0079	[-0.0096, 0.0777]	0.4420
	AUPRC	+0.0059	[-0.0350, 0.1031]	0.4460
T4SE (n=150)	Precision	+0.1192	[-0.0286, 0.2580]	0.1380
	Recall	+0.0333	[0.0000, 0.1000]	0.7160
	F1	+0.0846	[-0.0116, 0.3699]	0.0940
	AUC	+0.0020	[-0.0026, 0.0085]	0.4640
	AUPRC	+0.0077	[-0.0245, 0.0424]	0.6740
T6SE (n=20)	Precision	-0.0193	[-0.3642, 0.1265]	0.8960
	Recall	**+0.2000**	**[0.0500, 0.4000]**	**0.0180**
	F1	+0.0681	[-0.0231, 0.2433]	0.1340
	AUC	**+0.0096**	**[0.0012, 0.0233]**	**0.0200**
	AUPRC	**+0.0715**	**[0.0118, 0.3631]**	**0.0160**

4) Newly added S13 Table.

S13 Table. Class-wise statistical significance analysis of performance differences between GeoEPred and MoCETSE on the independent test set.

Model Variant	Metric	Δ	95% CI	*p*-values
No-effector (n=150)	Precision	+0.0227	[0.0000, 0.0496]	0.0820
	Recall	+0.0333	[-0.0200, 0.0867]	0.2500
	F1	+0.0288	[-0.0012, 0.0603]	0.0800
	AUC	+0.0065	[-0.0007, 0.0162]	0.0720
	AUPRC	+0.0053	[-0.0000, 0.0127]	0.0540
T1SE (n=20)	Precision	+0.0000	[0.0000, 0.0000]	1.0000
	Recall	+0.0000	[-0.1500, 0.1500]	1.0000
	F1	+0.0000	[-0.0855, 0.0855]	1.0000
	AUC	-0.0260	[-0.0908, 0.0017]	0.6500
	AUPRC	-0.0056	[-0.0399, 0.0210]	0.7960
T2SE (n=10)	Precision	-0.0214	[-0.1250, 0.0794]	0.8580
	Recall	+0.0000	[0.0000, 0.0000]	1.0000
	F1	-0.0194	[-0.1042, 0.0686]	0.8580
	AUC	-0.0112	[-0.0556, 0.0172]	0.7780
	AUPRC	+0.1313	[-0.0225, 0.4114]	0.1040
T3SE (n=30)	Precision	+0.0634	[-0.0358, 0.1765]	0.2800
	Recall	+0.0333	[-0.0667, 0.1667]	0.8020
	F1	+0.0486	[-0.0339, 0.1312]	0.2720
	AUC	+0.0090	[-0.0087, 0.0341]	0.4260
	AUPRC	+0.0324	[-0.0258, 0.1044]	0.3080
T4SE (n=150)	Precision	+0.1681	[0.0000, 0.3011]	0.0540
	Recall	+0.0000	[0.0000, 0.0000]	1.0000
	F1	+0.1042	[0.0000, 0.1914]	0.0540
	AUC	-0.0001	[-0.0038, 0.0032]	0.9760
	AUPRC	-0.0055	[-0.0316, 0.0184]	0.8520
T6SE (n=20)	Precision	-0.1304	[-0.2593, 0.0000]	0.1180
	Recall	+0.1500	[0.0000, 0.3000]	0.0720
	F1	+0.0113	[-0.0910, 0.1521]	0.8280
	AUC	+0.0098	[0.0000, 0.0279]	0.0860
	AUPRC	+0.0574	[0.0000, 0.1457]	0.0760

5. The construction of the remote homology benchmark is insufficiently described. The manuscript should explain how homologs were identified, how TM-scores were calculated, which structural alignment tools were used, and how benchmark independence was ensured.

Response: We sincerely thank you for raising this important and rigorous point. We fully agree that clearly describing the construction of the remote-homology

benchmark, the criteria used to determine homologous relationships, the structural alignment methods, and the independence between the training and benchmark sets is essential for accurately assessing the model's generalizability and ensuring the reproducibility of this study. Following your comment, we have further expanded and clarified the revised manuscript by providing detailed information on the construction of the remote-homology benchmark, the tools and the criteria for determining homologous relationships, the calculation of the relevant metrics, and the procedures used to verify dataset independence.

Specifically, we used the final training set as the reference dataset. The candidate evaluation pool consisted of proteins from the fixed independent test set and additional structures retrieved from PDB100, AFDB-SWISSPROT, and AFDB-PROTEOME using the training-set structures as Foldseek queries. For externally retrieved proteins, only entries with sufficiently clear functional annotations and supporting information for class assignment were retained. PDB100 annotations were cross-checked against the corresponding PDB records and original publications, whereas AFDB-SWISSPROT and AFDB-PROTEOME entries were verified against their UniProt records.

All candidates were then subjected to a two-stage filtering procedure. First, candidate sequences were compared against the complete training set using CD-HIT-2D, and candidates sharing ≥ 30% sequence identity with any training-set protein were removed. Second, Foldseek was used to identify structurally similar proteins in the training set, followed by refined pairwise structural alignment using TM-align. For benchmark construction, the TM-score normalized by the length of the candidate protein was used, and the maximum TM-score against the training set was recorded for each candidate. Following commonly used criteria in previous studies, we retained as remote-homology benchmark samples proteins that shared less than 30% sequence identity with every protein in the training set but exhibited a maximum TM-score greater than 0.5 to at least one training-set structure.

Before establishing the final benchmark, duplicate entries and proteins overlapping with the training set were removed. Benchmark independence was therefore ensured at the sample and sequence levels, whereas structural similarity to the training set was intentionally retained as the defining characteristic of the remote-homology benchmark. The final class distribution is shown in S3 Fig. No T2SE protein simultaneously satisfied the sequence-divergence, structural-similarity, and annotation criteria; therefore, T2SE was not represented in the final benchmark.

We once again sincerely thank you for your rigorous and careful review. Your comment prompted us to further improve the description of the construction and validation of the remote-homology benchmark, thereby enhancing the rigor,

transparency, and reproducibility of this part of our study.

The following content provides supplementary additions to the main text.

1) We added a new subsection entitled "Remote Homology Benchmark Construction and Structural Similarity Assessment" to the Supplementary Materials, in which we describe in detail the construction of the remote homology test set and the calculation of the TM-score (the "Description of datasets with distant homology" section in S3 Fig).

To assess the generalization of GeoEPred to remote homologs, we constructed a dedicated benchmark using the final training set as the reference dataset. The candidate evaluation pool consisted of two components: 260 proteins from the fixed independent test set and additional structurally similar candidate proteins retrieved from the PDB100, AFDB-SWISSPROT, and AFDB-PROTEOME databases [1], [2] using the training-set structures as Foldseek queries [3].

Among the external search results, we retained only entries with clearly defined protein names, functional descriptions, taxonomic origins, database accession numbers, and sufficient annotation or experimental evidence to support class assignment. Entries annotated solely as "putative proteins" or "uncharacterized proteins" were excluded. The annotations of PDB100 hits were further cross-checked against the corresponding PDB records and original publications [4], whereas annotations from AFDB-SWISSPROT and AFDB-PROTEOME were independently verified against their corresponding UniProt records [5].

All candidate proteins were subjected to the same two-stage filtering procedure. First, their sequences were compared against the complete training set using CD-HIT-2D [6], and only candidates with a maximum sequence identity below 30% to every training-set protein were retained. For each sequence-filtered candidate, Foldseek [3] was then used to search against the complete training structure set to identify structurally similar training proteins, and the resulting candidate–training structure pairs were subsequently realigned using TM-align [8]. The TM-score was calculated as:

$$\text{TM-score}=\max\left[\frac{1}{L_{target}}\sum_{i=1}^{L_{ali}}\frac{1}{1+\left(\frac{d_i}{d_0\left(L_{target}\right)}\right)^2}\right]$$

where

$$d_0\left(L_{target}\right)=1.24\left(L_{target}-15\right)^{1/3}-1.8$$

Here, L_{target} denotes the length of the candidate benchmark protein used for

normalization, L_{ali} represents the number of aligned residue pairs, and d_i is the distance between the *i*-th pair of aligned residues after structural superposition. The maximization is performed over alternative structural superpositions to obtain the optimal TM-score. TM-score was introduced by Zhang and Skolnick as a length-normalized measure of protein structural similarity [7], and TM-align optimizes structural alignment based on TM-score [8]. Throughout benchmark construction, the TM-score normalized by the length of the candidate protein was used. Following commonly used criteria in previous studies [8,9], we retained as remote-homology benchmark samples proteins that shared less than 30% sequence identity with every protein in the training set but exhibited a maximum TM-score greater than 0.5 to at least one training-set structure.

Before establishing the final benchmark, duplicate entries across all candidate sources and proteins overlapping with the training set were removed. We further confirmed that no identical protein sequences were shared between the final benchmark and the training set and that every retained benchmark protein had less than 30% sequence identity to every training-set protein. The final class distribution is presented in S3 Fig. No T2SE proteins simultaneously satisfied the sequence-divergence, structural-similarity, and annotation criteria; therefore, T2SE was not represented in the final benchmark.

6. The biological interpretability analysis remains largely qualitative. The highlighted residues and structural regions should be quantitatively compared with experimentally validated conserved motifs, catalytic residues, or known functional regions.

Response: We sincerely thank the reviewer for this constructive suggestion. We agree that quantitatively comparing model-highlighted regions with experimentally characterized conserved motifs, catalytic residues, and known functional regions provides a more comprehensive assessment of biological interpretability. Following the reviewer's suggestion, we performed a residue-level quantitative interpretability analysis to evaluate whether the regions highlighted by GeoEPred were associated with experimentally characterized functional elements. Specifically, for representative effector proteins, we compared high-attribution residues identified by the sequence and structural branches with experimentally validated functional regions, including secretion-associated motifs, host-interaction interfaces, and annotated functional domains. We quantified the overlap between model-highlighted residues and known functional regions and assessed enrichment using both hypergeometric enrichment analysis and circular-shift permutation tests, with the latter preserving the local organization of the attribution profile while randomizing its positional correspondence

with the annotated functional regions.

For the T3SS effector SlrP, we quantitatively compared residue-level attribution patterns generated by the sequence and structural branches with experimentally characterized functional regions. The N-terminal region of SlrP (residues 1-50) has been experimentally associated with the T3SS translocation signal. Among the top 5% highest-attribution residues identified by the sequence branch, 16 residues overlapped with this experimentally characterized region, corresponding to a 6.44-fold enrichment relative to the sequence-wide expectation. This enrichment was supported by circular shift permutation testing ($p = 0.0030$, BH $q = 0.0060$) and hypergeometric enrichment analysis ($p = 7.38\times10^{-11}$, BH $q = 1.48\times10^{-10}$). These results indicate that the sequence branch preferentially assigns attribution to the experimentally characterized T3SS translocation region of SlrP. The structural branch showed a distinct attribution pattern at the C-terminal E2 ubiquitin-conjugating enzyme interaction interface (residues 751-765). Two residues within this region were included among the top 5% structural-attribution residues, corresponding to a 2.68-fold enrichment. Although this enrichment did not reach statistical significance after multiple-testing correction (circular-shift permutation: $p = 0.0830$, BH $q = 0.0830$; hypergeometric test: $p = 0.1681$, BH $q = 0.1681$), the observed attribution pattern showed a tendency to localize toward this experimentally characterized host-interaction interface. Together with the structural localization pattern, these results suggest that the structural branch captures spatial features associated with the C-terminal host-interaction region of SlrP.

For the T4SS effector SidJ, we further quantified the correspondence between residue-level attribution patterns and experimentally characterized functional regions. The sequence branch showed preferential attribution toward linear sequence features associated with SidJ secretion and catalytic activity. Within the intrinsically disordered N-terminal region of SidJ (residues 1-50), the top 5% sequence-attribution residues showed a 4.37-fold enrichment relative to the sequence-wide expectation [56]. This overlap was supported by hypergeometric enrichment analysis ($p = 1.32\times10^{-5}$, BH $q = 5.27\times10^{-5}$), while the circular shift permutation test indicated an enrichment trend ($p = 0.0334$, BH $q = 0.1336$). Similarly, the conserved catalytic motif region (residues 348-380) showed enrichment among sequence-attributed residues. At the top 10% attribution threshold, 15 residues overlapped with this experimentally characterized catalytic region, corresponding to a 4.56-fold enrichment. This overlap was supported by hypergeometric analysis ($p = 7.00\times10^{-8}$, BH $q = 8.40\times10^{-7}$), while the circular shift permutation test indicated a consistent enrichment pattern ($p = 0.0250$, BH $q = 0.1336$). Together, these results indicate that the sequence branch captures residue-level sequence features associated with SidJ

secretion and catalytic function. The structural branch showed correspondence with the experimentally characterized calmodulin (CaM)-binding interface (residues 597-708). Among the top 5% structural-attribution residues, 11 residues overlapped with this interface, corresponding to a 1.95-fold enrichment. This enrichment was supported by circular shift permutation testing (p = 1.00×10^{-4}, BH q = 0.0012) and hypergeometric enrichment analysis (p = 0.0179, BH q = 0.0430). These results support the association between structural attribution and the experimentally characterized host-factor interaction interface of SidJ.

For the T6SS effector Tse5, the sequence branch showed preferential attribution toward experimentally characterized linear functional regions associated with secretion and toxin processing. Within the N-terminal Hcp interaction region (residues 1-35), 16 residues overlapped with the top 5% sequence-attribution residues, corresponding to a 9.12-fold enrichment relative to the sequence-wide expectation. This overlap showed a consistent enrichment pattern under circular shift permutation analysis (p = 0.0219, BH q = 0.0873), with additional support from hypergeometric enrichment analysis (p = 4.81×10^{-13}, BH q = 4.33×10^{-12}). These results indicate that the sequence branch preferentially assigns attribution to the experimentally characterized Hcp interaction region involved in Tse5 recruitment and secretion through the T6SS machinery. The structural branch exhibited a distinct attribution pattern associated with the three-dimensional functional interface of Tse5. Within the experimentally characterized Rhs plug/gateway region (residues 1115-1163), 10 residues overlapped with the top 5% structural-attribution residues, corresponding to a 4.07-fold enrichment relative to the sequence-wide expectation. This enrichment was supported by circular shift permutation analysis (p = 0.0011, BH q = 0.0099) and hypergeometric enrichment analysis (p = 8.72×10^{-5}, BH q = 3.92×10^{-4}). These results support the association between structural attribution and the experimentally characterized Rhs assembly interface involved in toxin processing and release. Together, these results demonstrate that the sequence and structural branches capture complementary functional information in Tse5, with the sequence branch emphasizing linear secretion-associated features and the structural branch highlighting spatially organized functional interfaces.

We have incorporated the corresponding revisions and supplementary data into the revised manuscript. We sincerely thank the reviewer again for this constructive suggestion, which has guided us to further refine our biological interpretability analysis framework and improve the rigor and completeness of the related evaluation.

The following content provides supplementary additions to the main text.

1) We have updated Fig 10 to more clearly illustrate the correspondence between residues/structural regions and experimentally validated conserved

motifs, catalytic residues, or known functional regions, as well as the positions within these key regions that receive high attention weights from our model.

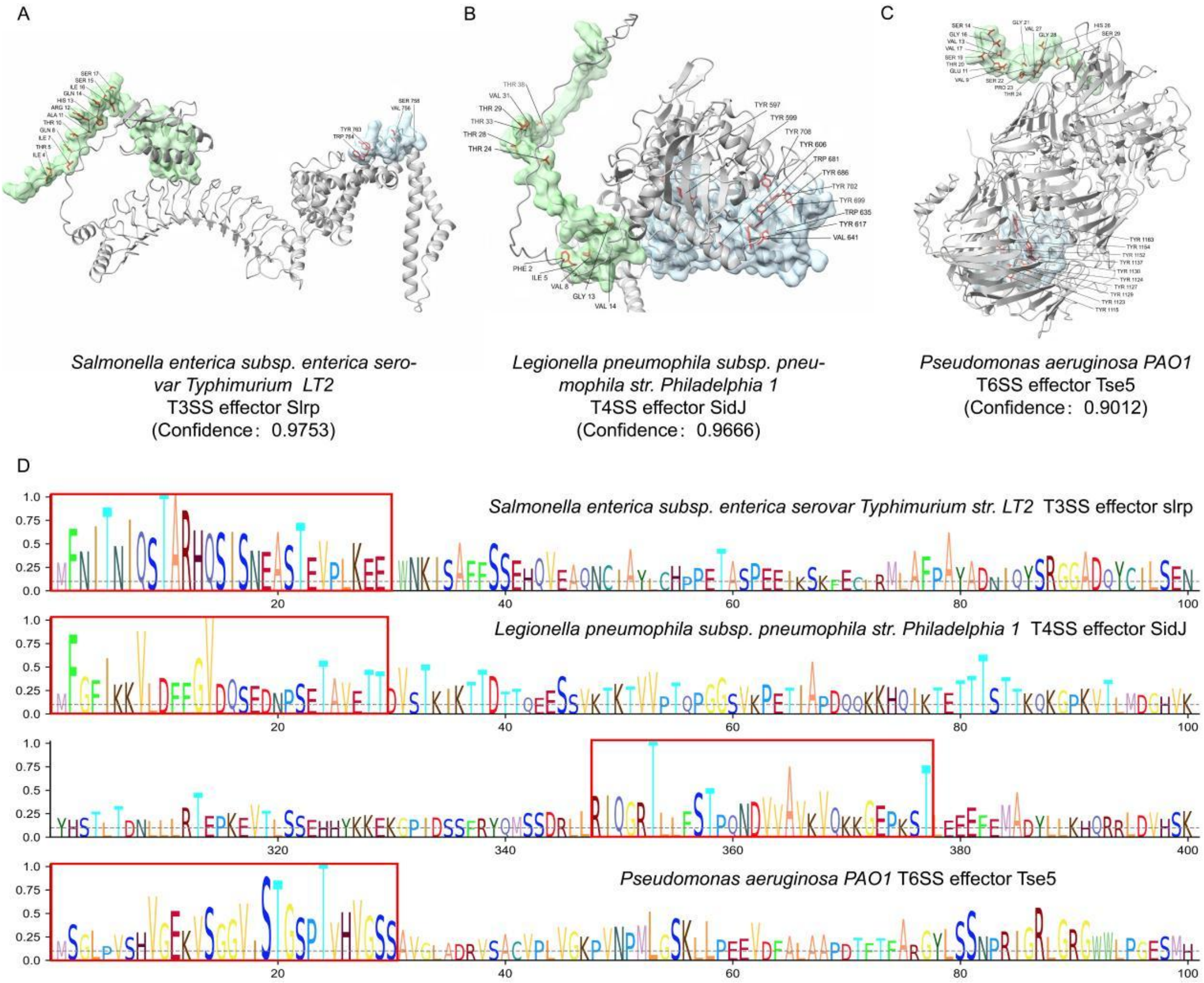

Fig 10. Structural and sequence-level interpretation of model attention for representative effector proteins. A-C: Visualization of the weights of E3 generalized enzyme SlrP (A), calmodulin-dependent gamma-glutamyltransferase SidJ (B), and toxin protein Tse5 (C). D: Sequence-level visualization of attention-weighted residues and conserved sequence patterns for the corresponding proteins. Red boxes indicate regions enriched with high-attention residues, highlighting the sequence segments preferentially recognized by the model.

2) Corresponding revisions have been made in the "Biological interpretability" section (Page 18–19, Lines 579–628).

For the T3SS effector SlrP from *Salmonella typhimurium*, we quantitatively evaluated the correspondence between residue-level attribution patterns and experimentally characterized functional regions. The N-terminal region of SlrP (residues 1–50) has been experimentally associated with the T3SS translocation signal [53]. Among the top 5% highest-attribution residues identified by the sequence branch, 16 residues overlapped with this experimentally characterized region, corresponding to a 6.44-fold enrichment relative to the sequence-wide expectation. This enrichment was supported by circular shift permutation testing (p = 0.0030, BH q = 0.0060) and

hypergeometric enrichment analysis ($p = 7.38 \times 10^{-11}$, BH $q = 1.48 \times 10^{-10}$). These results indicate that sequence-derived attribution patterns preferentially correspond to the experimentally characterized T3SS translocation region of SlrP. In contrast, the structural branch exhibited an attribution pattern associated with the C-terminal E2 ubiquitin-conjugating enzyme interaction interface (residues 751–765), which mediates host interaction in NEL-family effectors [54, 55]. Two residues within this region were included among the top 5% structural-attribution residues, corresponding to a 2.68-fold enrichment. Although the enrichment did not reach statistical significance in permutation testing ($p = 0.0830$, BH $q = 0.0830$) or hypergeometric analysis ($p = 0.1681$, BH $q = 0.1681$), the consistent enrichment trend together with the structural localization pattern suggests an association between structural attribution and the experimentally characterized host-interaction interface of SlrP. Together, these observations indicate that the sequence and structural branches provide complementary residue-level attribution patterns corresponding to distinct functional regions of SlrP.

For the T4SS effector SidJ from *Legionella pneumophila*, the sequence branch showed enrichment within regions associated with secretion and catalytic activity. Within the intrinsically disordered N-terminal region of SidJ (residues 1-50), the top 5% sequence-attribution residues showed a 4.37-fold enrichment relative to the sequence-wide expectation [56]. This overlap was supported by hypergeometric enrichment analysis ($p = 1.32 \times 10^{-5}$, BH $q = 5.27 \times 10^{-5}$), while the circular shift permutation test indicated a consistent enrichment trend ($p = 0.0334$, BH $q = 0.1336$). Similarly, the conserved catalytic motif region of SidJ (residues 348–380) showed enrichment among sequence-attributed residues. At the top 10% attribution threshold, 15 residues overlapped with this experimentally characterized catalytic region, corresponding to a 4.56-fold enrichment. This overlap was supported by hypergeometric analysis ($p = 7.00 \times 10^{-8}$, BH $q = 4.80 \times 10^{-7}$) and circular shift permutation testing ($p = 0.0250$, BH $q = 0.1336$). These results suggest that sequence attribution patterns are associated with sequence features underlying SidJ secretion and catalytic activity. The structural branch showed correspondence with the experimentally characterized calmodulin (CaM)-binding interface (residues 597–708), which is required for activation of SidJ glutamylase activity [57, 58]. Among the top 5% structural-attribution residues, 11 residues overlapped with this interface, corresponding to a 1.95-fold enrichment. This enrichment was supported by circular shift permutation testing ($p = 1.00 \times 10^{-4}$, BH $q = 0.0012$) and hypergeometric enrichment analysis ($p = 0.0179$, BH $q = 0.0430$). These results support an association between structural attribution patterns and the experimentally characterized host-factor interaction interface of SidJ [57, 59].

Regarding the *Pseudomonas aeruginosa PAO1* T6SS effector Tse5, within the N-terminal Hcp interaction region (residues 1–35), 16 residues overlapped with the top 5% sequence-attribution residues, corresponding to a 9.12-fold enrichment relative to the sequence-wide expectation. This overlap showed a consistent enrichment pattern under circular shift permutation analysis (p = 0.0219, BH q = 0.0873), with additional support from hypergeometric enrichment analysis ($p = 4.81 \times 10^{-13}$, BH $q = 4.33 \times 10^{-12}$). These results indicate that sequence attribution patterns preferentially correspond to the experimentally characterized Hcp interaction region involved in Tse5 recruitment and T6SS-mediated secretion [60]. The structural branch exhibited an attribution pattern associated with the three-dimensional functional interface of Tse5. Within the experimentally characterized Rhs plug/gateway region (residues 1115–1163), 10 residues overlapped with the top 5% structural-attribution residues, corresponding to a 4.07-fold enrichment relative to the sequence-wide expectation. This enrichment was supported by circular shift permutation analysis (p = 0.0011, BH q = 0.0099) and hypergeometric enrichment analysis ($p = 8.72 \times 10^{-5}$, BH $q = 3.92 \times 10^{-4}$). These results support the correspondence between structural attribution patterns and the experimentally characterized Rhs assembly interface involved in toxin processing and release [60].

2) Newly added S17 Table.

S17 Table. Quantitative residue-level attribution enrichment analysis of representative effector proteins using circular shift permutation and hypergeometric tests.

Protein	Branch	Functional region	Residue range	FE	Circular shift permutation p	Circular shift BH q	Hypergeometric p	Hypergeometric BH q
SlrP	Sequence	T3SS translocation signal	1–50	6.44×	0.0030	0.0060	7.38×10^{-11}	1.48×10^{-10}
SlrP	Structure	E2 interaction interface	751–765	2.68×	0.0830	0.0830	0.1681	0.1681
Tse5	Sequence	Hcp interaction region	1–35	9.12×	0.0219	0.0873	4.81×10^{-13}	4.33×10^{-12}
Tse5	Structure	Rhs plug/gateway interface	1115–1163	4.07×	0.0011	0.0099	8.72×10^{-5}	3.92×10^{-4}
SidJ	Sequence	T4SS translocation signal	1–50	4.37×	0.0334	0.1336	1.32×10^{-5}	5.27×10^{-5}
SidJ	Sequence	Conserved catalytic motif	348–380	4.56×	0.0250	0.1336	7.00×10^{-8}	8.40×10^{-7}
SidJ	Structure	CaM-binding interface	597–708	1.95×	1.00×10^{-4}	0.0012	0.0179	0.0430

7. The comparison with existing methods may not be entirely fair. Several competing methods were evaluated using predictions from web servers rather than under identical preprocessing and evaluation conditions. The authors should discuss this limitation or perform standardized comparisons whenever possible.

Response: We sincerely thank the reviewer for raising this important and rigorous

concern. We fully agree that comparisons based on publicly available web servers do not constitute fully standardized head-to-head evaluations, as the internal preprocessing procedures, model versions, default parameters, and decision thresholds of these servers cannot be completely controlled. Following the reviewer's suggestion, we carefully examined the availability of source code, model weights, and datasets for all competing methods. Because the binary classification methods provide only web servers and do not release complete training code or reusable model weights, these models could not be retrained under exactly the same computational conditions. To make the comparison as fair and standardized as possible, we therefore conducted additional dataset-matched benchmark experiments. For each benchmark, GeoEPred was trained from scratch using only the corresponding published training set, while the associated independent test set was kept completely separate and used exclusively for final evaluation. No samples from the independent test set were used for model training or model selection. We then compared the final performance of GeoEPred with the performance reported for the corresponding method in the original publication under the same benchmark setting.

As shown in Table 6, GeoEPred achieved performance comparable to or better than that of the competing models on their corresponding benchmark datasets. Notably, our model performed particularly well on the T6SE datasets, although its performance was slightly lower on the T4SEpp dataset. In light of your observation in Comment 3 that structural confidence may influence model performance, we further analyzed the structural confidence of the samples in these datasets (S14 Table). We found that the training sets Bastion6 and BastionX_T6SE had the highest mean structural confidence scores, with mean pLDDT values of 86.02 and 85.47, respectively, together with relatively small standard deviations of 12.38 and 12.65. These results suggest that the structural features in these training sets are of higher and more consistent quality, which may enable the model to learn more reliable structural patterns. In contrast, the T4SEpp training set had a lower mean pLDDT value (80.78) and a larger standard deviation (21.18), indicating a greater proportion of proteins with low-confidence predictions or intrinsically disordered regions. These differences in training data quality may largely contribute to the observed variation in model performance across datasets. This analysis further supports your important insight that confidence in predicted protein structures can influence structure-based representation learning. We have incorporated this analysis into the revised manuscript.

In the comparison with existing methods described previously, we provided the following clarification. GeoEPred, DeepSecE, and MoCETSE were trained under a unified experimental setting using the DeepSecE dataset, while retaining the

configurations reported in their respective original studies. For the binary classification models, because their source code was not publicly available, predictions were obtained using their publicly accessible web servers, which we considered the most reliable means of preserving the performance reported in the original studies. All models were subsequently evaluated on the same benchmark dataset containing multiple types of effector proteins, which has been widely used in this field to ensure consistency across comparisons. Because a single genome may encode multiple classes of effector proteins, this evaluation setting more closely reflects practical whole-genome prediction scenarios and further demonstrates the ability of GeoEPred to simultaneously identify different classes of effector proteins.

We once again sincerely thank you for your rigorous and meticulous review and for carefully examining the fairness of our comparative evaluation. Your comments prompted us not only to provide a more comprehensive explanation of the rationale and limitations of our original comparison strategy, but also to conduct additional dataset-matched experiments using the original training and independent test datasets of the competing methods. These improvements have further strengthened the rigor, transparency, and credibility of our comparative evaluation.

The following content provides supplementary additions to the main text.

1) We have added a discussion based on a more rigorous and fair comparison in the "Comparison with State-of-the-Art Models" subsection of the revised manuscript (Page 14–15, Lines 453–470).

Although some binary classification methods may achieve higher sensitivity on specific single tasks, they often exhibit a higher risk of cross-class false positives when applied to multi-class independent test sets (Fig.6D). For example, Bastion3, a well-performing binary classifier for T3SEs, correctly identified all T3SE samples in the test set but also produced a number of false-positive predictions for other effector types. Similarly, T1SEstacker performed well in T1SE identification but also showed cross-class misclassification by incorrectly assigning some non-T1SE proteins to the T1SE class (S2 Fig). From a practical perspective, because a single bacterial genome typically encodes multiple types of secretion effectors, a multi-class model with more balanced performance across different classes may be more useful for genome-scale applications. In this regard, GeoEPred showed stable and balanced performance in multi-class identification, making it suitable for prediction in complex genomic backgrounds.

To ensure a fair comparison, we trained and evaluated GeoEPred using the same training datasets as those reported for the corresponding binary classification models. Across seven benchmark datasets covering T3SE (two datasets), T4SE (three datasets), and T6SE (two datasets), GeoEPred achieved performance comparable to that of the

existing SOTA models on six of the seven test sets (Table 6). To further investigate the performance differences observed across datasets, we analyzed the corresponding pLDDT scores for each dataset (S14 Table). The results showed that GeoEPred tended to perform better on datasets with higher overall structural prediction quality, suggesting that its predictive performance may, to some extent, be influenced by the quality of the predicted input structures.

2) We have added Table 6 to provide a clear comparison of GeoEPred's performance against the corresponding binary classification models on the respective datasets.

Table 6. This study compares GeoEPred with state-of-the-art (SOTA) binary classification models for T3SE, T4SE, and T6SE prediction using the same training and evaluation datasets reported in the corresponding studies.

Compared model	Task	Method	Performance metrics				
			REC	PR	ACC	F1	MCC
Bastion3	T3SE	GeoEPred	0.941	**0.976**	**0.959**	0.958	**0.918**
		Bastion3	**0.954**	0.963	0.958	0.958	0.917
T3SEpp	T3SE	GeoEPred	**0.995**	**0.798**	**0.858**	**0.886**	**0.735**
		T3SEpp	0.929	0.796	0.829	0.857	0.659
Bastion4	T4SE	GeoEPred	**1.000**	0.880	**0.976**	0.935	**0.924**
		Bastion4	0.967	**0.942**	0.953	**0.954**	0.907
T4SEpp	T4SE	GeoEPred	0.870	0.963	0.981	0.913	0.905
		T4SEpp	**0.900**	**1.000**	**0.988**	**0.947**	**0.942**
BastionX	T4SE	GeoEPred	0.923	**0.978**	**0.951**	**0.949**	**0.904**
		BastionX	**1.000**	0.882	0.933	0.938	0.874
Bastion6	T6SE	GeoEPred	0.950	**0.968**	**0.992**	**0.958**	**0.954**
		Bastion6	**1.000**	0.465	0.895	0.635	0.642
BastionX	T6SE	GeoEPred	0.979	**0.976**	**0.978**	**0.978**	**0.955**
		BastionX	**1.000**	0.935	0.975	0.967	0.933

3) We have added S14 Table to provide a detailed summary of the structural prediction quality for the datasets associated with the corresponding binary classification models.

S14 Table. Sample-level pLDDT statistics across different benchmark datasets, ranked by the mean pLDDT of the training set.

Rank	Prediction Task	Dataset	Training Set pLDDT (Mean ± SD)	Test Set pLDDT (Mean ± SD)
1	T6SE	Bastion6	86.02 ± 12.38	83.82 ± 14.04
2	T6SE	BastionX T6SE	85.47 ± 12.65	82.19 ± 13.03
3	T4SE	BastionX T4SE	85.19 ± 13.61	74.97 ± 18.25
4	T4SE	Bastion4	81.71 ± 16.02	84.10 ± 13.21
5	T3SE	Bastion3	81.35 ± 16.45	75.75 ± 17.65
6	T4SE	T4SEpp	80.78 ± 21.18	84.20 ± 13.88
7	T3SE	T3SEpp	75.54 ± 17.99	74.54 ± 17.99

8. No statistical analysis accompanies the reported performance metrics. Confidence intervals, repeated experiments with different random seeds should be included for all major comparisons.

Response: We sincerely thank the reviewer for this insightful comment. We completely agree that demonstrating statistical significance is crucial to prove that the performance gains achieved by our method over competing baselines are robust, reproducible, and not the result of random experimental variations. According to your suggestion, In the revised manuscript, we have conducted comprehensive and rigorous statistical significance tests for all experiments involving performance comparisons in the revised manuscript.

Specifically, we repeated all experiments using five different random seeds. Model training was conducted using five-fold cross-validation. During each fold, we saved the model weights corresponding to the highest F1 score on the validation set. The selected model weights were then fixed and applied to an independent test set that was not used during either model training or model selection and was reserved exclusively for final performance evaluation. In all revised performance comparison tables, we report the mean, standard deviation, and confidence interval of the five-fold cross-validation results, together with the performance of the best-performing model on the independent test set. Finally, to rigorously assess the performance improvement of the proposed model on the independent test set, we performed a paired stratified bootstrap hypothesis test with (N = 1000) resampling iterations. Resampling was stratified according to the ground-truth labels to preserve the proportions of all six classes in each bootstrap sample. In each iteration, the proposed model and the baseline model were evaluated using the same resampled observations, and the paired difference in performance was calculated. The corresponding p-values and 95% confidence intervals are reported in the revised manuscript.

We have systematically revised and updated all comparative sections, tables, and figures throughout the manuscript. The relevant statistical analysis results have been fully incorporated into the main text and the supplementary materials. We once again

express our sincere gratitude to the reviewer for their constructive and professional feedback; your valuable suggestions have enabled us to strengthen the experimental design, substantiate our arguments, and ensure the reliability of our conclusions.

The following content provides supplementary additions to the main text.

1) Corresponding revisions have been made in the "Multimodal feature ablation" section (Page 9–10, Lines 275–294).

We systematically disentangled the contributions of the cross-modal architecture by evaluating the predictive utility of structure-only features, sequence-only features, refined sequence-semantic representations, and the final multimodal fusion representation. Table 2 presents a detailed quantitative ablation analysis of each feature configuration under five-fold cross-validation and on the independent test set.

Under five-fold cross-validation, models relying solely on sequence or structural features exhibited limited discriminative ability, achieving accuracies of 0.681 (95% CI: 0.668–0.694) and 0.615 (95% CI: 0.571–0.660), respectively, with corresponding F1 scores of 0.639 (95% CI: 0.616–0.663) and 0.541 (95% CI: 0.486–0.596). Incorporating refined sequence-semantic representations substantially improved the accuracy to 0.877 (95% CI: 0.860–0.895) and the F1 score to 0.854 (95% CI: 0.823–0.885). The multimodal fusion representation achieved the best overall performance, with an accuracy of 0.899 (95% CI: 0.878–0.920) and an F1 score of 0.880 (95% CI: 0.853–0.907).

When evaluated on the independent test set, the multimodal fusion features outperformed the second-best refined sequence-semantic features, improving accuracy from 0.881 to 0.923 (95% CI: 0.0115–0.0731, p = 0.008) and the F1 score from 0.846 to 0.884 (95% CI: 0.0069–0.0702, p = 0.014). Notably, by incorporating structural features, the multimodal collaborative framework achieved an MCC gain of 0.0604 over the sequence-semantic baseline (95% CI: 0.0194–0.1043, p =0.0002), providing evidence for the strong representational complementarity between evolutionary sequence semantics and spatial geometric features (S5 Table). By leveraging the complementary information provided by both modalities, the fusion mechanism improved class discrimination and enhanced predictive robustness.

2) Updated Table 2.

Table 2. Performance evaluation of multimodal feature ablation.

Feature Type	ACC		F1		AUPRC	
	CV	Ind	CV	Ind	CV	Ind
Geometric Priors	0.615	0.527	0.541	0.459	0.561	0.454
Sequence only	0.681	0.723	0.639	0.664	0.681	0.683
Sequence Semantics	0.877	0.881	0.854	0.846	0.893	0.907
GeoEPred	**0.899**	**0.923**	**0.880**	**0.884**	**0.907**	**0.915**

3) Newly added S5 Table.

S5 Table. Compared with the single-modal model, the proposed model shows improvements in performance, 95% confidence intervals, and statistical significance analysis.

Baseline Model	Metric	Δ	95% CI	*p*-values
Geometric Priors	ACC	+0.3962	[0.3308, 0.4577]	0.0020
	F1	+0.4258	[0.3512, 0.5025]	0.0020
	MCC	+0.5742	[0.4860, 0.6646]	0.0020
	AUC	+0.1659	[0.1349, 0.1969]	0.0020
	AUPRC	+0.4606	[0.3743, 0.5462]	0.0020
Sequence only	ACC	+0.2000	[0.1499, 0.2538]	0.0020
	F1	+0.2203	[0.1555, 0.2893]	0.0020
	MCC	+0.2658	[0.1969, 0.3371]	0.0020
	AUC	+0.0599	[0.0420, 0.0809]	0.0020
	AUPRC	+0.2315	[0.1589, 0.3152]	0.0020
Sequence Semantics	ACC	+0.0423	[0.0115, 0.0731]	0.0080
	F1	+0.0379	[0.0069, 0.0702]	0.0140
	MCC	+0.0604	[0.0194, 0.1043]	0.0020
	AUC	+0.0082	[0.0000, 0.0180]	0.0540
	AUPRC	+0.0081	[-0.0257, 0.0563]	0.5035

4) A new subsection titled "Model component evaluation" has been added to the "Materials and methods" section (Page 11–12, Lines 342–362).

We conducted a series of ablation experiments to evaluate the contribution of each core component to the model's overall performance. Specifically, we compared the complete GeoEPred model with three variants, each lacking one component: (1) a model combining adaptive pooling and feature tokenization but excluding contrastive learning; (2) a model combining feature tokenization and contrastive learning but excluding adaptive pooling; and (3) a model combining adaptive pooling and contrastive learning but excluding feature tokenization.

As shown in Table 3, in five-fold cross-validation, the model combining adaptive attention pooling and contrastive learning achieved an accuracy (ACC) of 0.861 (95% CI: 0.842–0.880) and an F1 score of 0.829 (95% CI: 0.799–0.859). Upon introducing feature tokenization, the accuracy (ACC) and F1 score improved to 0.899 (95% CI: 0.878–0.920) and 0.88 (95% CI: 0.853–0.907), respectively. When adaptive attention pooling was replaced by mean pooling, the accuracy and F1 score dropped to 0.886 (95% CI: 0.868–0.904) and 0.863 (95% CI: 0.835–0.891), respectively. On the independent test set, removing contrastive learning resulted in decreases of 3% (95% CI: 0.00002–0.0602, p = 0.0519) in F1 and 4.85% (95% CI: 0.0094–0.0910, p = 0.0140) in MCC. Replacing adaptive attention pooling with mean pooling resulted in

decreases of 3.38% (95% CI: −0.0071–0.0816, $p = 0.0939$) in MCC and 5.32% (95% CI: 0.0083–0.1014, $p = 0.0220$) in F1. Regarding AUPRC—a metric particularly informative under class imbalance—GeoEPred achieved a score of 0.9146, representing a 3.94% improvement over the model without feature tokenization (0.8753; 95% CI: 0.0112–0.0888, $p = 0.0040$). F1 and MCC also improved by 4.58% (95% CI: 0.0181–0.0764, $p = 0.0020$) and 6.81% (95% CI: 0.0303–0.1085, $p = 0.0020$), respectively (S7 Table). These results indicate that feature tokenization helps maintain precision-recall performance and enhances the quality of probability predictions for the minority class.

5) Newly added Table 3.

Table 3. Performance comparison of model variants in the ablation study using ACC, F1, and AUPRC on cross-validation and independent test sets.

Model Variant	ACC		F1		AUPRC	
	CV	Ind	CV	Ind	CV	Ind
w/o Contrastive Learning	0.881	0.889	0.851	0.854	0.901	0.887
w/o Adaptive Attention Pooling	0.886	0.885	0.863	0.851	0.899	0.890
w/o Feature Tokenization	0.861	0.873	0.829	0.838	0.864	0.875
Full Model (Ours)	**0.899**	**0.923**	**0.880**	**0.884**	**0.907**	**0.915**

6) Newly added S7 Table.

S7 Table. Performance gains, 95% confidence intervals, and statistical significance of the full model compared with different ablated model variants.

Model Variant	Metric	Δ	95% CI	*p*-values
w/o Contrastive Learning	ACC	+0.0346	[0.0038, 0.0692]	0.0320
	F1	+0.0300	[0.00002, 0.0602]	0.0519
	MCC	+0.0485	[0.0094, 0.0910]	0.0140
	AUC	+0.0064	[-0.0047, 0.0217]	0.2917
	AUPRC	+0.0278	[-0.0043, 0.0686]	0.1019
w/o Adaptive Attention Pooling	ACC	+0.0385	[0.0038, 0.0769]	0.0380
	F1	+0.0338	[-0.0071, 0.0816]	0.0939
	MCC	+0.0532	[0.0083, 0.1014]	0.0220
	AUC	+0.0034	[-0.0077, 0.0134]	0.5554
	AUPRC	+0.0249	[-0.0091, 0.0789]	0.1379
w/o Feature Tokenization	ACC	+0.0500	[0.0231, 0.0808]	0.0020
	F1	+0.0461	[0.0181, 0.0764]	0.0020
	MCC	+0.0683	[0.0303, 0.1085]	0.0020
	AUC	+0.0007	[-0.0118, 0.0102]	0.8352
	AUPRC	+0.0394	[0.0112, 0.0888]	0.0040

7) Corresponding revisions have been made in the "Ablation research on fusion mechanism" section (Page 12, Lines 363–378).

Building on the preceding results indicating the contribution of feature tokenization to model performance, we further compared different feature integration strategies. Specifically, feature tokenization, the core fusion mechanism of GeoEPred, was compared with cross-attention fusion and direct feature concatenation.

As shown in Table 4, feature tokenization yielded the strongest overall performance. In five-fold cross-validation, it achieved an accuracy of 0.899 (95% CI: 0.878–0.920), exceeding direct concatenation (0.884; 95% CI: 0.862–0.906) and cross-attention fusion (0.886; 95% CI: 0.870–0.902) by 1.5 and 1.3 percentage points, respectively. On the independent test set, feature tokenization further improved AUPRC by 0.0521 and 0.0415 relative to feature concatenation and cross-attention fusion, respectively. As shown in S8 Table, paired stratified bootstrap analysis indicated that both AUPRC improvements were statistically significant ($p = 0.002$ vs. concatenation; $p = 0.012$ vs. cross-attention fusion). Given that AUPRC evaluates performance across varying decision thresholds and is particularly informative under class imbalance, these findings suggest that feature tokenization provides more robust discrimination across the full precision–recall spectrum. Overall, the results support feature tokenization as an effective multimodal integration mechanism within GeoEPred, with its most pronounced advantage observed in AUPRC on the independent test set.

8) Updated Table 4.

Table 4. Performance evaluation metrics for different fusion strategies.

Method	ACC		F1		AUPRC	
	CV	Ind	CV	Ind	CV	Ind
Concatenation	0.884	0.889	0.860	0.848	0.854	0.863
Cross-Attention Fusion	0.886	0.908	0.863	0.862	0.901	0.873
Feature Tokenization	**0.899**	**0.923**	**0.880**	**0.884**	**0.907**	**0.915**

9) Newly added S8 Table.

S8 Table. Performance improvements, 95% confidence intervals, and statistical significance analysis of the proposed model compared with different fusion strategies.

Method	Metric	Δ	95% CI	*p*-values
Concatenation	ACC	+0.0346	[0.0038, 0.0692]	0.0360
	F1	+0.0368	[-0.0006, 0.0792]	0.0559
	AUC	+0.0039	[-0.0087, 0.0146]	0.4795
	AUPRC	+0.0521	[0.0195, 0.1043]	0.0020
Cross-Attention Fusion	ACC	+0.0154	[-0.0154, 0.0462]	0.3916
	F1	+0.0224	[-0.0152, 0.0670]	0.2717
	AUC	+0.0054	[-0.0046, 0.0159]	0.2877
	AUPRC	+0.0415	[0.0070, 0.0908]	0.0120

10) Corresponding revisions have been made in the "Comparison with baseline methods" section (Page 13, Lines 416–430).

To comprehensively evaluate the architectural performance of GeoEPred, we systematically benchmarked it against 11 baseline models under a unified input configuration. These baselines comprised seven conventional machine-learning algorithms (SVM, Random Forest, Gradient Boosting, KNN, Naive Bayes, Decision Tree, and LDA) and four representative deep-learning architectures (MLP, BiLSTM-Attention, GRU, and Vanilla Transformer).

As shown in Table 5, GeoEPred achieved overall superior performance in five-fold cross-validation compared with SVM, the best-performing conventional machine-learning model, and BiLSTM-Attention, the best-performing deep-learning model. Although SVM achieved an accuracy comparable to that of GeoEPred during cross-validation, GeoEPred demonstrated stronger generalization performance on the independent test set, with improvements of 1.5% in accuracy (95% CI: 0.003-0.031, $p = 0.024$) and 3.0% in AUPRC (95% CI: 0.020–0.058, $p = 0.002$) relative to SVM (S9 Table). GeoEPred also achieved a higher AUPRC than BiLSTM-Attention, with an improvement of 3.0% (95% CI: 0.018-0.060, $p = 0.003$). Collectively, these results indicate that GeoEPred can effectively learn discriminative feature representations while exhibiting more robust generalization to independent unseen data, particularly providing improved predictive accuracy and reliability under class-imbalanced conditions.

11) Updated Table 5.

Table 5. Comparison of the proposed method with baseline methods.

Method	ACC		F1		AUPRC	
	CV	Ind	CV	Ind	CV	Ind
Traditional Machine Learning						
SVM	0.892	0.908	0.854	0.878	0.882	0.885
Random Forest	0.882	0.896	0.786	0.806	0.825	0.831
Gradient Boosting	0.865	0.869	0.770	0.749	0.805	0.794
KNN	0.857	0.831	0.809	0.804	0.828	0.821
Naive Bayes	0.804	0.815	0.744	0.757	0.768	0.778
Decision Tree	0.691	0.692	0.603	0.611	0.642	0.642
LDA	0.578	0.619	0.611	0.641	0.602	0.632
Deep Learning Method						
BiLSTM_Attention	0.862	0.900	0.852	0.864	0.879	0.885
MLP	0.875	0.900	0.856	0.850	0.880	0.871
GRU	0.884	0.900	0.848	0.860	0.883	0.886
Vanilla_Transformer	0.866	0.881	0.854	0.852	0.871	0.891
Our method						
GeoEPred	**0.899**	**0.923**	**0.880**	**0.884**	**0.907**	**0.915**

12) Newly added S9 Table.

S9 Table. Performance gain, 95% confidence intervals, and statistical significance analysis of the proposed model compared to the best traditional machine learning and deep learning baseline methods.

Method	Metric	Δ	95% CI	*p*-values
SVM	ACC	+0.015	[0.003, 0.031]	0.024
	F1	+0.002	[-0.003, 0.007]	0.420
	AUC	+0.021	[0.005, 0.037]	0.009
	AUPRC	+0.030	[0.020, 0.058]	0.002
BiLSTM_Attention	ACC	+0.023	[0.008, 0.042]	0.008
	F1	+0.016	[0.014, 0.052]	0.028
	AUC	+0.028	[0.010, 0.046]	0.002
	AUPRC	+0.030	[0.018, 0.060]	0.003

9. The manuscript does not adequately demonstrate the generalizability of GeoEPred beyond the evaluated Gram-negative secretion systems. Additional discussion or validation on other secretion systems or independent external datasets would strengthen the claims of broad applicability.

Response: We sincerely thank the reviewer for this rigorous comment. We fully agree that adequately evaluating the generalization ability of the model on independent data and clearly defining its scope of applicability are important prerequisites for establishing a reliable bioinformatics prediction tool.

Following the reviewer's suggestion, we conducted an additional external evaluation of GeoEPred using experimentally validated effectors from bacterial genomes that were not included in the original dataset, in order to assess its generalization ability on recently validated samples. Accordingly, we searched for experimentally validated effectors reported in recently sequenced bacterial genomes, focusing on genomes that were not represented in our original dataset, and performed an additional evaluation. Specifically, we collected experimentally validated effectors from Aeromonas schubertii ATCC 43700 [1], Aeromonas jandaei DSM 7311 [2], Xanthomonas hortorum pv. pelargonii 305 [3], and Aeromonas veronii C4 [4]. Among these, the genomes of A. veronii C4 and X. hortorum pv. pelargonii 305 were newly sequenced and released after 2025. Although experimentally validated effectors from other secretion-system classes have also been reported in the past two years, the corresponding genomes had been sequenced earlier and were already represented in our dataset. We therefore did not include those samples in this additional evaluation. After excluding sequences sharing more than 60% sequence identity with proteins in the training set, we obtained 9 experimentally validated T3SEs and 7 experimentally validated T6SEs. On this small, genome-level unseen test set, GeoEPred showed

encouraging generalization performance, correctly identifying 8 of the 9 T3SEs and all 7 T6SEs, generally with high prediction confidence, as shown in the table below. Given the relatively small size of this external validation set, we have included this analysis in the revised manuscript as an exploratory external validation experiment and have interpreted the findings cautiously to avoid overstating the broad generalizability of GeoEPred.

We again thank the reviewer for this constructive suggestion. This comment prompted us to add an exploratory external validation based on independent, newly sequenced genomes and to further clarify the generalization conclusions that can currently be supported for GeoEPred and the boundaries of its applicability, thereby improving the rigor and completeness of the revised manuscript.

Reference:

[1] Michova H, Pliva J, Jirsova A, Jurnecka D, Kamanova J. Cytotoxicity induced by Aeromonas schubertii is orchestrated by a unique set of type III secretion system effectors. Vet Res. 2025;56(1):113. doi:10.1186/s13567-025-01548-2.

[2] Fridman CM, Keppel K, Rudenko V, Altuna-Alvarez J, Albesa-Jové D, Bosis E, Salomon D.A new class of type VI secretion system effectors can carry two toxic domains and are recognized through the WHIX motif for export. PLoS Biol. 2025;23(3):e3003053. doi:10.1371/journal.pbio.3003053.

[3] Wagner N, Ben-Meir D, Teper D, Pupko T. Complete genome sequence of an Israeli isolate of Xanthomonas hortorum pv. pelargonii strain 305 and novel type III effectors identified in Xanthomonas. Front Plant Sci. 2023;14:1155341. doi:10.3389/fpls.2023.1155341.

[4] Jiang X, Li H, Ma J, Li H, Ma X, Tang Y, Li J, Chi X, Deng Y, Zeng S, Liu Z. Role of Type VI secretion system in pathogenic remodeling of host gut microbiota during Aeromonas veronii infection. ISME J. 2024;18(1):wrae053. doi:10.1093/ismejo/wrae053.

The following content provides supplementary additions to the main text.

1) New content has been added to the subsection "Model training" (Page 8, Lines 228–233).

GeoEPred was trained using stratified five-fold cross-validation, with all experiments repeated using five different random seeds (42, 52, 62, 72, and 82). For each random seed, the available training data were partitioned into training and validation subsets within each fold, and the model checkpoint achieving the highest macro-F1 score on the validation set was selected and retained. The independent test set remained completely isolated throughout model training, hyperparameter determination, and model selection, and was used solely for the final performance evaluation.

2) New content has been added to the Discussion section (Page 21, Lines 713–719).

Despite the predictive performance and potential applicability of GeoEPred, several limitations remain. First, experimentally validated effector proteins are unevenly distributed across different secretion system classes, which may affect model training and performance evaluation for classes with fewer samples. In addition, because of the current availability of data, GeoEPred is mainly applicable to the secretion systems covered in this study and has not yet been extended to other secretion systems or effector proteins associated with Gram-positive bacteria. As more high-quality experimentally validated data become available, the framework can be gradually extended to additional secretion systems and a broader range of bacterial groups.

3) We added S18 table to provide a more comprehensive evaluation of GeoEPred on recently experimentally validated effector proteins.

S18 Table. GeoEPred predictions for recently experimentally validated bacterial effector proteins.

Effector	Strain	Secretion system	GeoEPred prediction	Confidence	Reference
AopI	*Aeromonas schubertii ATCC 43700*	T3SE	T3SE	0.99202687	[3]
AopJ	*Aeromonas schubertii ATCC 43700*	T3SE	T3SE	0.9745239	[3]
AopL	*Aeromonas schubertii ATCC 43700*	T3SE	T3SE	0.9941506	[3]
AopT	*Aeromonas schubertii ATCC 43700*	T3SE	T3SE	0.99421096	[3]
AopU	*Aeromonas schubertii ATCC 43700*	T3SE	T3SE	0.993871	[3]
TseI-like	*Aeromonas jandaei DSM 7311*	T6SE	T6SE	0.99375856	[4]
Tle1-like	*Aeromonas jandaei DSM 7311*	T6SE	T6SE	0.9903679	[4]
Awe1	*Aeromonas jandaei DSM 7311*	T6SE	T6SE	0.990186	[4]
DUF3289	*Aeromonas jandaei DSM 7311*	T6SE	T6SE	0.98906076	[4]
XopBB	*X. hortorum pv. pelargonii 305*	T3SE	T3SE	0.99400467	[5]
XopBC	*X. hortorum pv. pelargonii 305*	T3SE	T3SE	0.99064296	[5]
XopBD	*X. hortorum pv. pelargonii 305*	T3SE	T3SE	0.99371815	[5]
XopBE	*X. hortorum pv. pelargonii 305*	T3SE	Non-Effector	0.9620185	[5]
Tse1	*A. veronii C4*	T6SE	T6SE	0.99262416	[6]
Tse2	*A. veronii C4*	T6SE	T6SE	0.9911902	[6]
Tse3	*A. veronii C4*	T6SE	T6SE	0.9931722	[6]

10. Several implementation details required for reproducibility are missing. The manuscript should provide complete information regarding optimizer settings, learning rate schedule, batch size, early stopping criteria, random seed initialization, hardware configuration, and hyperparameter optimization strategy.

Response: We sincerely thank you for this important comment and for the rigor with which you have evaluated our work. We fully agree that implementation details—including the optimizer settings, learning-rate scheduling strategy, batch size, early-stopping criterion, random-seed initialization, hardware environment, and hyperparameter optimization strategy—are essential for ensuring the reproducibility of this study. We sincerely apologize for not providing a sufficiently comprehensive

and detailed description of these aspects in the original manuscript. Following your suggestion, we carefully re-examined our source code, configuration files, and experimental records and have systematically added the relevant information to the revised manuscript.

We once again sincerely thank you for your rigorous and meticulous review and for placing such strong emphasis on research reproducibility. Your careful scrutiny of the model implementation details prompted us to report the experimental settings and training procedures more systematically and comprehensively, thereby further enhancing the transparency, rigor, and reproducibility of our study.

The following content provides supplementary additions to the main text.

1) To address this concern, we have added a new subsection entitled "Model training" to Section 2 of the revised manuscript (Page 8–9, Lines 227–250).

GeoEPred was trained using stratified five-fold cross-validation, with all experiments repeated using five different random seeds (42, 52, 62, 72, and 82). For each random seed, the available training data were partitioned into training and validation subsets within each fold, and the model checkpoint achieving the highest macro-F1 score on the validation set was selected and retained. The independent test set remained completely isolated throughout model training, hyperparameter determination, and model selection, and was used solely for the final performance evaluation.

The model was implemented in PyTorch v2.8.0 and executed on an NVIDIA A100 GPU with 40 GB of memory. The software environment consisted of Python 3.12.9 and CUDA 12.8. Model parameters were initialized using Xavier initialization and optimized using AdamW with a weight decay of 1×10^{-4}. The initial learning rates were set to 2×10^{-5} for the sequence encoder, 3×10^{-4} for the structure encoder, and 1×10^{-4} for the remaining modules. The learning rates were updated after each epoch using cosine annealing with warm restarts, with an initial cycle length of 20 epochs and a cycle-length multiplier of 2 ($T_0 = 20$ and $T_{mult} = 2$).

The batch size and maximum number of training epochs were set to 32 and 30, respectively. Early stopping was performed by monitoring the macro-F1 score on the validation set, with a patience of five epochs, and the checkpoint achieving the best validation performance in each fold was retained. Automatic mixed precision and dynamic loss scaling were employed during training, and the global gradient norm was clipped to a maximum value of 1.0.

Class imbalance was addressed using inverse-class-frequency weighted random sampling. Focal loss was adopted as the primary classification objective, with a focusing parameter of 2.0 and a label-smoothing coefficient of 0.05. The sequence-modality dropout probability was set to 0.25, whereas a conventional

dropout rate of 0.1 was applied within the sequence Transformer encoder, cross-modal attention module, and Transformer classifier.

Reviewer #2:

1. ***The manuscript presents a promising multimodal framework, but the dataset construction lacks sufficient detail to ensure reproducibility. The authors should explicitly describe how positive and negative samples were selected, whether homologous proteins were removed between training and test sets, and how data leakage was prevented beyond the reported 60% CD-HIT filtering.***

Response: We thank the reviewer for this rigorous and important comment. We fully agree that the sequence-redundancy removal procedure was not described with sufficient clarity in the original manuscript, and we sincerely apologize for this omission. In response to the reviewer's comment, we thoroughly re-examined the partitioning of all datasets and have added a complete description of the data-processing workflow, model-training procedure, and validation results to the revised manuscript and Supplementary Materials.

To minimize the possibility that sequence redundancy could lead to an overestimation of model performance, we adopted the widely used dataset-construction strategy proposed by Zhang et al.[1] and used CD-HIT v4.8.1 to remove redundant sequences from the candidate datasets. Specifically, we applied a global sequence identity threshold of 60%. In addition, the aligned region was required to cover at least 80% of the lengths of both the shorter and longer sequences. We first pooled all candidate samples from the training and independent test sets and performed redundancy removal, retaining only one representative sequence from each cluster. This procedure ensured that identical or highly similar protein sequences were not represented more than once in the combined dataset.

Following this initial redundancy-removal step, we performed an additional cross-dataset comparison using CD-HIT-2D with the same criteria. The redundancy-reduced independent test set was used as the reference dataset, whereas the candidate training set was used as the query dataset to be filtered. Any training sequence sharing ≥60% sequence identity with a sequence in the independent test set, with the aligned region covering at least 80% of the lengths of both the shorter and longer sequences, was removed from the candidate training set. During this cross-dataset filtering step, the independent test set remained unchanged; only highly similar sequences in the training set that could potentially lead to information leakage were removed. After these procedures, the final training set contained 3,155 sequences, and the independent test set contained 260 sequences. Furthermore, no protein identifiers were shared between the training and independent test sets. Thus, the final training and independent test sets shared neither identical protein sequences nor highly similar sequence pairs meeting the predefined criteria.

We have added these details to the "Dataset Construction" section of the revised manuscript. To further enhance the transparency and reproducibility of this study, we have also made the following materials publicly available: the FASTA files before and after redundancy removal; the representative sequence files and ".clstr" clustering files generated by CD-HIT; the CD-HIT-2D output files; the five-fold cross-validation split files; all commands used in the analyses; and the corresponding statistical scripts. All data and code are available at the following URL:
https://github.com/shouzhensong/GeoEPred.

Reference:

[1] Zhang Y, Guan J, Li C, Wang Z, Deng Z, Gasser RB, Song J, Ou HY. DeepSecE: A deep-learning-based framework for multiclass prediction of secreted proteins in Gram-negative bacteria. Research (Wash D C). 2023;6:0258. doi:10.34133/research.0258.

The following content provides supplementary additions to the main text.

1) Corresponding revisions have been made in the "Dataset construction" section (Page 4, Lines 59–88).

The datasets used for model training and evaluation were compiled from datasets released with established effector prediction methods and from widely used biological databases.

The dataset construction and redundancy-removal procedures were designed with reference to DeepSecE [13], a representative study in the field. Specifically, T1SE and T2SE sequences were obtained from the TSE-ARF dataset [21]. The T3SE and T6SE datasets were compiled by integrating sequences from Bastion3 [7], DeepT3 [22], SecReT6 [23], Bastion6 [8], DeepT3_4 [24], and TSE-ARF [21]. The T4SE dataset was assembled from DeepSecE [13], OPT4e [25], T4SEfinder [26], iT4SE-EP [27], Bastion4 [28], T4SE-XGB [29], TSE-ARF [21], T4SEpp [30], and DeepT3_4 [24]. Following previous representative studies [31, 32], the negative class was constructed using curated non-secreted proteins and homologous sequences from selected pathogenic bacteria reported in earlier studies. These candidate negative samples had been further screened against multiple bacterial genomes using BLASTp in the original studies, based on predefined thresholds for the *E*-value and sequence identity. To provide an objective assessment of model generalization, we additionally assembled an independent test set. The T3SE, T4SE, T6SE, and non-secreted protein samples were collected from the test datasets released with Bastion3, CNN-T4SE [33], and Bastion6, whereas the T1SE and T2SE samples were randomly selected from the original BastionHub dataset.

To minimize potential overestimation of model performance caused by sequence redundancy, CD-HIT v4.8.1 [33] was applied separately to the candidate training set and the independent test set. A global sequence identity threshold of 60% was used,

and the aligned region was required to cover at least 80% of both the shorter and longer sequences. Within each dataset, sequences from all effector and non-effector classes were pooled before clustering, and only one representative sequence was retained from each cluster. Cross-dataset filtering was subsequently performed using CD-HIT-2D under the same identity and coverage criteria. The non-redundant independent test set was used as the reference dataset, and candidate training sequences showing at least 60% global sequence identity to any test sequence were removed. This procedure minimized the risk of information leakage between the training and test sets.

The data sources for each effector type and the corresponding sample numbers after CD-HIT filtering are summarized in S1 Table. The resulting training set comprised 1,575 non-effector proteins, 128 T1SEs, 65 T2SEs, 505 T3SEs, 623 T4SEs, and 259 T6SEs. The independent test set contained 150 non-effector proteins, 20 T1SEs, 10 T2SEs, 30 T3SEs, 30 T4SEs, and 20 T6SEs (S1 Fig A). Protein lengths ranged from fewer than 100 to more than 1,000 amino acids, with most sequences falling between 100 and 700 amino acids (S1 Fig B). Detailed information on all datasets used in this study is summarized in S1 Table.

2. ***The manuscript reports superior performance across multiple benchmarks, yet the statistical significance of the improvements is not evaluated. The authors should perform statistical tests to demonstrate that the reported performance gains over competing methods are statistically meaningful.***

Response: We sincerely thank the reviewer for this insightful comment. We completely agree that demonstrating statistical significance is crucial to prove that the performance gains achieved by our method over competing baselines are robust, reproducible, and not the result of random experimental variations. According to your suggestion, we have conducted comprehensive and rigorous statistical significance tests for all experiments involving performance comparisons in the revised manuscript.

Specifically, we repeated all experiments using five different random seeds. Model training was conducted using five-fold cross-validation. During each fold, we saved the model weights corresponding to the highest F1 score on the validation set. The selected model weights were then fixed and applied to an independent test set that was not used during either model training or model selection and was reserved exclusively for final performance evaluation. In all revised performance comparison tables, we report the mean, standard deviation, and confidence interval of the five-fold cross-validation results, together with the performance of the best-performing model on the independent test set. Finally, to rigorously assess the performance

improvements achieved by the proposed model on the independent test set, we performed a paired stratified bootstrap hypothesis test with 1,000 resampling iterations. Resampling was stratified according to the ground-truth labels to preserve the proportions of all six classes in each bootstrap sample. In each iteration, the proposed model and the baseline model were evaluated on the same resampled observations, and the paired difference in performance between the two models was calculated. The corresponding p-values and 95% confidence intervals were then obtained for each comparison.

We have systematically revised and updated all comparative sections, tables, and figures throughout the manuscript. The relevant statistical analysis results have been fully incorporated into the main text and the supplementary materials. We once again express our sincere gratitude to the reviewer for their constructive and professional feedback; your valuable suggestions have enabled us to strengthen the experimental design, substantiate our arguments, and ensure the reliability of our conclusions.

The following content provides supplementary additions to the main text.

1) Corresponding revisions have been made in the "Multimodal feature ablation" section (Page 10, Lines 281–299).

We systematically disentangled the contributions of the cross-modal architecture by evaluating the predictive utility of structure-only features, sequence-only features, refined sequence-semantic representations, and the final multimodal fusion representation. Table 2 presents a detailed quantitative ablation analysis of each feature configuration under five-fold cross-validation and on the independent test set.

Under five-fold cross-validation, models relying solely on sequence or structural features exhibited limited discriminative ability, achieving accuracies of 0.681 (95% CI: 0.668–0.694) and 0.615 (95% CI: 0.571–0.660), respectively, with corresponding F1 scores of 0.639 (95% CI: 0.616–0.663) and 0.541 (95% CI: 0.486–0.596). Incorporating refined sequence-semantic representations substantially improved the accuracy to 0.877 (95% CI: 0.860–0.895) and the F1 score to 0.854 (95% CI: 0.823–0.885). The multimodal fusion representation achieved the best overall performance, with an accuracy of 0.899 (95% CI: 0.878–0.920) and an F1 score of 0.880 (95% CI: 0.853–0.907).

When evaluated on the independent test set, the multimodal fusion features outperformed the second-best refined sequence-semantic features, improving accuracy from 0.881 to 0.923 (95% CI: 0.0115–0.0731, p = 0.008) and the F1 score from 0.846 to 0.884 (95% CI: 0.0069–0.0702, p = 0.014). Notably, by incorporating structural features, the multimodal collaborative framework achieved an MCC gain of 0.0604 over the sequence-semantic baseline (95% CI: 0.0194, 0.1043, p =0.002), providing evidence for the strong representational complementarity between

evolutionary sequence semantics and spatial geometric features (S5 Table). By leveraging the complementary information provided by both modalities, the fusion mechanism improved class discrimination and enhanced predictive robustness.

2) Updated Table 2.

Table 2. Performance evaluation of multimodal feature ablation.

Feature Type	ACC		F1		AUPRC	
	CV	Ind	CV	Ind	CV	Ind
Geometric Priors	0.615	0.527	0.541	0.459	0.561	0.454
Sequence only	0.681	0.723	0.639	0.664	0.681	0.683
Sequence Semantics	0.877	0.881	0.854	0.846	0.893	0.907
GeoEPred	**0.899**	**0.923**	**0.880**	**0.884**	**0.907**	**0.915**

3) Newly added S5 Table.

S5 Table. Compared with the single-modal model, the proposed model shows improvements in performance, 95% confidence intervals, and statistical significance analysis.

Baseline Model	Metric	Δ	95% CI	*p*-values
Geometric Priors	ACC	+0.3962	[0.3308, 0.4577]	0.0020
	F1	+0.4258	[0.3512, 0.5025]	0.0020
	MCC	+0.5742	[0.4860, 0.6646]	0.0020
	AUC	+0.1659	[0.1349, 0.1969]	0.0020
	AUPRC	+0.4606	[0.3743, 0.5462]	0.0020
Sequence only	ACC	+0.2000	[0.1499, 0.2538]	0.0020
	F1	+0.2203	[0.1555, 0.2893]	0.0020
	MCC	+0.2658	[0.1969, 0.3371]	0.0020
	AUC	+0.0599	[0.0420, 0.0809]	0.0020
	AUPRC	+0.2315	[0.1589, 0.3152]	0.0020
Sequence Semantics	ACC	+0.0423	[0.0115, 0.0731]	0.0080
	F1	+0.0379	[0.0069, 0.0702]	0.0140
	MCC	+0.0604	[0.0194, 0.1043]	0.0020
	AUC	+0.0082	[0.0000, 0.0180]	0.0540
	AUPRC	+0.0081	[-0.0257, 0.0563]	0.5035

4) A new subsection titled "Model component evaluation" has been added to the "Materials and methods" section (Page 11–12, Lines 343–362).

We conducted a series of ablation experiments to evaluate the contribution of each core component to the model's overall performance. Specifically, we compared the complete GeoEPred model with three variants, each lacking one component: (1) a model combining adaptive pooling and feature tokenization but excluding contrastive learning; (2) a model combining feature tokenization and contrastive learning but excluding adaptive pooling; and (3) a model combining adaptive pooling and

contrastive learning but excluding feature tokenization.

As shown in Table 3, in five-fold cross-validation, the model combining adaptive attention pooling and contrastive learning achieved an accuracy (ACC) of 0.861 (95% CI: 0.842-0.880) and an F1 score of 0.829 (95% CI: 0.799-0.859). Upon introducing feature tokenization, the accuracy (ACC) and F1 score improved to 0.899 (95% CI: 0.878-0.920) and 0.88 (95% CI: 0.853-0.907), respectively. When adaptive attention pooling was replaced by mean pooling, the accuracy and F1 score dropped to 0.886 (95% CI: 0.868-0.904) and 0.863 (95% CI: 0.835-0.891), respectively. On the independent test set, removing contrastive learning resulted in decreases of 3% (95% CI: 0.00002-0.0602, p = 0.0519) in F1 and 4.85% (95% CI: 0.0094-0.0910, p = 0.0140) in MCC. Replacing adaptive attention pooling with mean pooling resulted in decreases of 3.38% (95% CI: −0.0071–0.0816, p = 0.0939) in MCC and 5.32% (95% CI: 0.0083-0.1014, p = 0.0220) in F1. Regarding AUPRC—a metric particularly informative under class imbalance—GeoEPred achieved a score of 0.9146, representing a 3.94% improvement over the model without feature tokenization (0.8753; 95% CI: 0.0112–0.0888, p = 0.0040). F1 and MCC also improved by 4.58% (95% CI: 0.0181-0.0764, p = 0.0020) and 6.81% (95% CI: 0.0303-0.1085, p = 0.0020), respectively (S7 Table). These results indicate that feature tokenization helps maintain precision-recall performance and enhances the quality of probability predictions for the minority class.

5) Newly added Table 3.

Table 3. Performance comparison of model variants in the ablation study using ACC, F1, and AUPRC on cross-validation and independent test sets.

Model Variant	ACC		F1		AUPRC	
	CV	Ind	CV	Ind	CV	Ind
w/o Contrastive Learning	0.881	0.889	0.851	0.854	0.901	0.887
w/o Adaptive Attention Pooling	0.886	0.885	0.863	0.851	0.899	0.890
w/o Feature Tokenization	0.861	0.873	0.829	0.838	0.864	0.875
Full Model (Ours)	**0.899**	**0.923**	**0.880**	**0.884**	**0.907**	**0.915**

6) Newly added S7 Table.

S7 Table. Performance gains, 95% confidence intervals, and statistical significance of the full model compared with different ablated model variants.

Model Variant	Metric	Δ	95% CI	p-values
w/o Contrastive Learning	ACC	+0.0346	[0.0038, 0.0692]	0.0320
	F1	+0.0300	[0.00002, 0.0602]	0.0519
	MCC	+0.0485	[0.0094, 0.0910]	0.0140
	AUC	+0.0064	[-0.0047, 0.0217]	0.2917
	AUPRC	+0.0278	[-0.0043, 0.0686]	0.1019

w/o Adaptive Attention Pooling	ACC	+0.0385	[0.0038, 0.0769]	0.0380
	F1	+0.0338	[-0.0071, 0.0816]	0.0939
	MCC	+0.0532	[0.0083, 0.1014]	0.0220
	AUC	+0.0034	[-0.0077, 0.0134]	0.5554
	AUPRC	+0.0249	[-0.0091, 0.0789]	0.1379
w/o Feature Tokenization	ACC	+0.0500	[0.0231, 0.0808]	0.0020
	F1	+0.0461	[0.0181, 0.0764]	0.0020
	MCC	+0.0683	[0.0303, 0.1085]	0.0020
	AUC	+0.0007	[-0.0118, 0.0102]	0.8352
	AUPRC	+0.0394	[0.0112, 0.0888]	0.0040

7) Corresponding revisions have been made in the "Ablation research on fusion mechanism" section (Page 12, Lines 363–378).

Building on the preceding results indicating the contribution of feature tokenization to model performance, we further compared different feature integration strategies. Specifically, feature tokenization, the core fusion mechanism of GeoEPred, was compared with cross-attention fusion and direct feature concatenation.

As shown in Table 4, feature tokenization yielded the strongest overall performance. In five-fold cross-validation, it achieved an accuracy of 0.899 (95% CI: 0.878–0.920), exceeding direct concatenation (0.884; 95% CI: 0.862–0.906) and cross-attention fusion (0.886; 95% CI: 0.870–0.902) by 1.5 and 1.3 percentage points, respectively. On the independent test set, feature tokenization further improved AUPRC by 0.0521 and 0.0415 relative to feature concatenation and cross-attention fusion, respectively. As shown in S8 Table, paired stratified bootstrap analysis indicated that both AUPRC improvements were statistically significant ($p= 0.002$ vs. concatenation; $p=0.012$ vs. cross-attention fusion). Given that AUPRC evaluates performance across varying decision thresholds and is particularly informative under class imbalance, these findings suggest that feature tokenization provides more robust discrimination across the full precision–recall spectrum. Overall, the results support feature tokenization as an effective multimodal integration mechanism within GeoEPred, with its most pronounced advantage observed in AUPRC on the independent test set.

8) Updated Table 4.

Table 4. Performance evaluation metrics for different fusion strategies.

Method	ACC		F1		AUPRC	
	CV	Ind	CV	Ind	CV	Ind
Concatenation	0.884	0.889	0.860	0.848	0.854	0.863
Cross-Attention Fusion	0.886	0.908	0.863	0.862	0.901	0.873
Feature Tokenization	**0.899**	**0.923**	**0.880**	**0.884**	**0.907**	**0.915**

9) Newly added S8 Table.

S8 Table. Performance improvements, 95% confidence intervals, and statistical significance analysis of the proposed model compared with different fusion strategies.

Method	Metric	Δ	95% CI	*p*-values
Concatenation	ACC	+0.0346	[0.0038, 0.0692]	0.0360
	F1	+0.0368	[-0.0006, 0.0792]	0.0559
	AUC	+0.0039	[-0.0087, 0.0146]	0.4795
	AUPRC	+0.0521	[0.0195, 0.1043]	0.0020
Cross-Attention Fusion	ACC	+0.0154	[-0.0154, 0.0462]	0.3916
	F1	+0.0224	[-0.0152, 0.0670]	0.2717
	AUC	+0.0054	[-0.0046, 0.0159]	0.2877
	AUPRC	+0.0415	[0.0070, 0.0908]	0.0120

10) Corresponding revisions have been made in the "Comparison with baseline methods" section (Page 13, Lines 415–430).

To comprehensively evaluate the architectural performance of GeoEPred, we systematically benchmarked it against 11 baseline models under a unified input configuration. These baselines comprised seven conventional machine-learning algorithms (SVM, Random Forest, Gradient Boosting, KNN, Naive Bayes, Decision Tree, and LDA) and four representative deep-learning architectures (MLP, BiLSTM-Attention, GRU, and Vanilla Transformer).

As shown in Table 5, GeoEPred achieved overall superior performance in five-fold cross-validation compared with SVM, the best-performing conventional machine-learning model, and BiLSTM-Attention, the best-performing deep-learning model. Although SVM achieved an accuracy comparable to that of GeoEPred during cross-validation, GeoEPred demonstrated stronger generalization performance on the independent test set, with improvements of 1.5% in accuracy (95% CI: 0.003-0.031, p = 0.024) and 3.0% in AUPRC (95% CI: 0.020–0.058, p = 0.002) relative to SVM (S9 Table). GeoEPred also achieved a higher AUPRC than BiLSTM-Attention, with an improvement of 3.0% (95% CI: 0.018-0.060, p = 0.003). Collectively, these results indicate that GeoEPred can effectively learn discriminative feature representations while exhibiting more robust generalization to independent unseen data, particularly providing improved predictive accuracy and reliability under class-imbalanced conditions.

11) Updated Table 5.

Table 5. Comparison of the proposed method with baseline methods.

Method	ACC		F1		AUPRC	
	CV	Ind	CV	Ind	CV	Ind
Traditional Machine Learning						
SVM	0.892	0.908	0.854	0.878	0.882	0.885
Random Forest	0.882	0.896	0.786	0.806	0.825	0.831
Gradient Boosting	0.865	0.869	0.770	0.749	0.805	0.794
KNN	0.857	0.831	0.809	0.804	0.828	0.821
Naive Bayes	0.804	0.815	0.744	0.757	0.768	0.778
Decision Tree	0.691	0.692	0.603	0.611	0.642	0.642
LDA	0.578	0.619	0.611	0.641	0.602	0.632
Deep Learning Method						
BiLSTM_Attention	0.862	0.900	0.852	0.864	0.879	0.885
MLP	0.875	0.900	0.856	0.850	0.880	0.871
GRU	0.884	0.900	0.848	0.860	0.883	0.886
Vanilla_Transformer	0.866	0.881	0.854	0.852	0.871	0.891
Our method						
GeoEPred	**0.899**	**0.923**	**0.880**	**0.884**	**0.907**	**0.915**

12) Newly added S9 Table.

S9 Table. Performance gain, 95% confidence intervals, and statistical significance analysis of the proposed model compared to the best traditional machine learning and deep learning baseline methods.

Method	Metric	Δ	95% CI	*p*-values
SVM	ACC	+0.015	[0.003, 0.031]	0.024
	F1	+0.002	[-0.003, 0.007]	0.420
	AUC	+0.021	[0.005, 0.037]	0.009
	AUPRC	+0.030	[0.020, 0.058]	0.002
BiLSTM_Attention	ACC	+0.023	[0.008, 0.042]	0.008
	F1	+0.016	[0.014, 0.052]	0.028
	AUC	+0.028	[0.010, 0.046]	0.002
	AUPRC	+0.030	[0.018, 0.060]	0.003

13) We have added statistical significance analysis to the "Comparison with State-of-the-Art Models" section of the revised manuscript to further validate the reliability of performance differences among different models. (Page 14, Lines 441–462)

From the overall results, GeoEPred achieved predictive performance comparable to that of current state-of-the-art methods, with a more evident advantage in the T6SE prediction task (Fig.6A-C). In comparison with the multi-class models, GeoEPred achieved comparable predictive performance across most classes (S11 Table). In

terms of overall performance, GeoEPred improved accuracy by 2.69% (p = 0.098) and 3.46% (p = 0.048) compared with DeepSecE and MoCETSE, respectively, which were among the better-performing baseline models. For the T6SE class (S12 Table), GeoEPred showed favorable predictive performance, with both threshold-independent metrics, AUC and AUPRC, being significantly higher than those of DeepSecE (AUC: 95% CI: 0.0012-0.0233, p = 0.020; AUPRC: 95% CI: 0.0118-0.3631, p = 0.016). As shown in S13 Table, GeoEPred also showed a modest improvement in AUC and AUPRC compared with MoCETSE (AUC: Δ = +0.0098, 95% CI: 0.0000-0.0279, p = 0.086; AUPRC: Δ = +0.0098, 95% CI: 0.0000-0.1457, p = 0.076). These results suggest that GeoEPred has good discriminative capability for T6SE prediction and may have an advantage in identifying fine-grained features associated with T6SEs.

14) Newly added S11 Table.

S11 Table. Overall Performance Comparison among GeoEPred, DeepSecE, and MoCETSE.

Metric	GeoEPred	DeepSecE	MoCETSE	p-value (vs DeepSecE)	p-value (vs MoCETSE)
Accuracy	92.31%	89.62%	88.85%	0.098	0.048
Macro Recall	94.33%	87.05%	86.10%	0.018	0.012
Macro AUPRC	0.9146	0.8872	0.8784	0.088	0.042
Macro F1-score	88.43%	86.60%	85.54%	0.182	0.092
Macro AUC	0.9859	0.9832	0.9818	0.145	0.082
Macro Precision	85.79%	86.35%	85.12%	0.620	0.450

15) Newly added S12 Table.

S12 Table. Class-wise statistical significance analysis of performance differences between GeoEPred and DeepSecE on the independent test set.

Model Variant	Metric	Δ	95% CI	p-values
No-effector (n=150)	Precision	+0.0022	[0.0000, 0.0493]	0.0820
	Recall	+0.0133	[-0.0467, 0.0668]	0.7780
	F1	+0.0173	[-0.0168, 0.0518]	0.3280
	AUC	+0.0131	[0.0014, 0.0279]	0.0200
	AUPRC	+0.0115	[0.0014, 0.0253]	0.0180
T1SE (n=20)	Precision	+0.0000	[0.0000, 0.0000]	1.0000
	Recall	+0.0000	[-0.1500, 0.1500]	1.0000
	F1	+0.0000	[-0.0855, 0.0855]	1.0000
	AUC	-0.0306	[-0.1044, 0.0500]	0.6480
	AUPRC	-0.0150	[-0.0957, 0.0407]	0.7720
T2SE (n=10)	Precision	-0.1714	[-0.3462, -0.0557]	0.0040
	Recall	+0.0000	[0.0000, 0.0000]	1.0000
	F1	-0.1394	[-0.2557, -0.0474]	0.0040
	AUC	-0.0160	[-0.0652, 0.0136]	0.7080

	AUPRC	+0.0961	[-0.0738, 0.3794]	0.3100
T3SE (n=30)	Precision	+0.3611	[0.0300, 0.2927]	0.0160
	Recall	+0.0000	[-0.3000, 0.3000]	1.0000
	F1	+0.0679	[-0.0017, 0.1775]	0.0660
	AUC	+0.0079	[-0.0096, 0.0777]	0.4420
	AUPRC	+0.0059	[-0.0350, 0.1031]	0.4460
T4SE (n=150)	Precision	+0.1192	[-0.0286, 0.2580]	0.1380
	Recall	+0.0333	[0.0000, 0.1000]	0.7160
	F1	+0.0846	[-0.0116, 0.3699]	0.0940
	AUC	+0.0020	[-0.0026, 0.0085]	0.4640
	AUPRC	+0.0077	[-0.0245, 0.0424]	0.6740
T6SE (n=20)	Precision	-0.0193	[-0.3642, 0.1265]	0.8960
	Recall	+0.2000	[0.0500, 0.4000]	0.0180
	F1	+0.0681	[-0.0231, 0.2433]	0.1340
	AUC	+0.0096	[0.0012, 0.0233]	0.0200
	AUPRC	+0.0715	[0.0118, 0.3631]	0.0160

16) Newly added S13 Table.

S13 Table. Class-wise statistical significance analysis of performance differences between GeoEPred and MoCETSE on the independent test set.

Model Variant	Metric	Δ	95% CI	*p*-values
No-effector (n=150)	Precision	+0.0227	[0.0000, 0.0496]	0.0820
	Recall	+0.0333	[-0.0200, 0.0867]	0.2500
	F1	+0.0288	[-0.0012, 0.0603]	0.0800
	AUC	+0.0065	[-0.0007, 0.0162]	0.0720
	AUPRC	+0.0053	[-0.0000, 0.0127]	0.0540
T1SE (n=20)	Precision	+0.0000	[0.0000, 0.0000]	1.0000
	Recall	+0.0000	[-0.1500, 0.1500]	1.0000
	F1	+0.0000	[-0.0855, 0.0855]	1.0000
	AUC	-0.0260	[-0.0908, 0.0017]	0.6500
	AUPRC	-0.0056	[-0.0399, 0.0210]	0.7960
T2SE (n=10)	Precision	-0.0214	[-0.1250, 0.0794]	0.8580
	Recall	+0.0000	[0.0000, 0.0000]	1.0000
	F1	-0.0194	[-0.1042, 0.0686]	0.8580
	AUC	-0.0112	[-0.0556, 0.0172]	0.7780
	AUPRC	+0.1313	[-0.0225, 0.4114]	0.1040
T3SE (n=30)	Precision	+0.0634	[-0.0358, 0.1765]	0.2800
	Recall	+0.0333	[-0.0667, 0.1667]	0.8020
	F1	+0.0486	[-0.0339, 0.1312]	0.2720
	AUC	+0.0090	[-0.0087, 0.0341]	0.4260
	AUPRC	+0.0324	[-0.0258, 0.1044]	0.3080
T4SE	Precision	+0.1681	[0.0000, 0.3011]	0.0540

(n=150)	Recall	+0.0000	[0.0000, 0.0000]	1.0000
	F1	+0.1042	[0.0000, 0.1914]	0.0540
	AUC	-0.0001	[-0.0038, 0.0032]	0.9760
	AUPRC	-0.0035	[-0.0316, 0.0184]	0.8520
T6SE (n=20)	Precision	-0.1304	[-0.2593, 0.0000]	0.1180
	Recall	+0.1500	[0.0000, 0.3000]	0.0720
	F1	+0.0113	[-0.0910, 0.1521]	0.8280
	AUC	+0.0098	[0.0000, 0.0279]	0.0860
	AUPRC	+0.0574	[0.0000, 0.1457]	0.0760

3. *The manuscript relies entirely on ESMFold-predicted structures without discussing the influence of prediction confidence on downstream performance. The authors should investigate the effect of low-confidence structural regions to validate the robustness of the structural branch.*

Response: We sincerely thank the reviewer for raising this important and insightful concern. We fully agree that the influence of ESMFold-predicted structural quality on model performance should be explicitly evaluated. In response to the reviewer's comment, we conducted additional analyses of pLDDT scores and model performance to further examine and discuss the relationship between ESMFold prediction confidence and downstream classification performance.

First, we extracted residue-level predicted Local Distance Difference Test (pLDDT) scores from the ESMFold-predicted structures and analyzed their distributions across all proteins in the training and independent test sets. pLDDT scores range from 0 to 100 and provide a residue-level estimate of structural prediction confidence, with higher values indicating greater confidence in the predicted local structure. The results showed that the overall confidence of the predicted structures was moderate to high, with a mean pLDDT score of approximately 77 in both datasets. The highly consistent pLDDT distributions between the training and independent test sets indicated no evident confidence-related distributional shift. Moreover, approximately 40% of the residues fell within the high-confidence range (pLDDT > 90), providing a generally reliable structural basis for downstream feature extraction.

Next, we divided the 260 test proteins into four predefined structural-confidence groups according to their mean pLDDT scores: *Very low* (pLDDT < 50), *Low* (50 ≤ pLDDT < 70), *Confident* (70 ≤ pLDDT < 90), and *Very high* (pLDDT > 90). The corresponding classification accuracies were 81.82%, 87.50%, 88.89%, and 96.47%, respectively (Fig.7A). These results revealed an overall increase in predictive accuracy with increasing structural confidence. Nevertheless, the model retained an accuracy of 81.82% even in the very low-confidence group, suggesting that its

predictions did not depend exclusively on high-confidence structural information. Furthermore, we treated mean pLDDT as a continuous variable and fitted a sample-level logistic regression model using classification correctness as the outcome (Fig.7B). After adjustment for the true class label and protein sequence length, each 10-point increase in mean pLDDT was associated with a 79.8% increase in the odds of correct classification (OR = 1.798, 95% CI: 1.242-2.602, $p = 0.002$). Consistent with this finding, correctly classified proteins had a higher mean pLDDT than incorrectly classified proteins (79.88 vs 71.54; $p = 0.029$); BH-adjusted ($q = 0.044$; Cliff's $\delta = 0.271$). Collectively, these results indicate that structural prediction confidence is associated with model performance. Importantly, the model also maintained good predictive performance in the lower-confidence groups, suggesting that sequence features provide critical contextual constraints and thereby contribute to the overall stability of the prediction system.

Finally, we further investigated the agreement between ESMFold-predicted structures and experimentally resolved protein structures. Because the benchmark datasets used in this study primarily provide protein sequences and class labels but lack consistent database accession numbers, we performed sequence-based mapping to match the ESMFold-predicted structures with protein structures available in the RCSB Protein Data Bank (PDB). To ensure reliable correspondence, we retained only matches with 100% sequence identity, 100% coverage of both the query and target sequences, and identical sequence lengths, resulting in 57 proteins with exact full-length sequence matches to PDB entries. We further selected proteins for which the experimentally resolved coordinates covered at least 90% of the corresponding full-length sequence, yielding 29 matched protein pairs for structural comparison. For these proteins, the ESMFold-predicted structures were aligned with the corresponding experimentally resolved structures using US-align. Structural agreement was evaluated using the TM-score normalized to the experimentally resolved structure and the root-mean-square deviation (RMSD). Mean pLDDT showed a strong positive correlation with TM-score (Spearman's $\rho = 0.922$, $p = 1.30 \times 10^{-12}$) and a strong negative correlation with RMSD ($\rho = -0.905$, $p = 1.53 \times 10^{-11}$). The median TM-score was 0.921, and the median RMSD was 1.63 Å. These results provide additional structure-level evidence that higher ESMFold confidence is associated with greater agreement between predicted and experimentally resolved structures, supporting the use of pLDDT as an indicator of structural reliability in our analysis.

We have made the corresponding revisions in the revised manuscript. We sincerely thank the reviewer again for this important suggestion. This comment highlighted the important role of structural validation in assessing model reliability and prompted us to further strengthen our analysis of predicted structural confidence

and its relationship with model performance, thereby providing a more comprehensive and rigorous understanding of the reliability and applicability of the structural representations used by our model.

The following content provides supplementary additions to the main text.

1) New content has been added to the subsection "Protein language model" (Page 5, Lines 123–135).

Building on these advances, we incorporated an ESMFold-based structural branch into our model. Three-dimensional structures predicted directly from protein sequences were used as structural inputs to extract geometric and conformational features, enabling the model to characterize proteins from a spatial perspective. To assess the reliability of the structures predicted by ESMFold, we calculated the per-residue predicted Local Distance Difference Test (pLDDT) scores for all protein structures in the training and test sets. The pLDDT score ranges from 0 to 100 and provides a residue-level estimate of local structural confidence, with higher scores indicating more reliable predictions. Summary statistics for the structural confidence of the two datasets are presented in Table 1. The mean pLDDT score was approximately 77 for both the training and test sets, indicating that the predicted structures generally exhibited good confidence. Moreover, the pLDDT distributions were comparable between the two datasets, suggesting no evident shift in structural quality between the training and test sets. Approximately 40% of the residues fell within the high-confidence range (pLDDT ≥ 90), indicating that a substantial proportion of the predicted structures provided a reliable structural basis for subsequent geometric feature extraction and protein classification.

2) Newly added Table 1.

Table 1. Quality of ESMFold-Predicted Structures and Their pLDDT Distributions.

Metric	Training Set	Testing Set
pLDDT (Mean ± SD)	76.67 ± 17.65	78.57 ± 17.80
Median pLDDT	82.43	84.36
pLDDT ≥ 90 (High Confidence)	40.42%	44.17%
70 ≤ pLDDT ≤ 90 (Medium Confidence)	26.41%	24.33%
pLDDT < 70 (Low Confidence)	26.64%	23.90%

3) A new subsection titled "Influence of ESMFold structural confidence on model performance" has been added to the "Results" section (Page 15–16, Lines 472–502).

Because our model uses ESMFold-predicted structures as inputs to the structural branch, we further investigated the association between predicted structural confidence and effector protein classification performance.

Based on the mean protein-level pLDDT score, the independent test set was divided using fixed thresholds into four confidence groups: *Very low* (pLDDT < 50), *Low* (50 ≤ pLDDT < 70), *Confident* (70 ≤ pLDDT < 90), and *Very high* (pLDDT ≥ 90). As shown in Fig.7A, the corresponding accuracies were 81.82%, 87.50%, 88.89%, and 96.47%, respectively, exhibiting an overall increase with structural confidence.

To quantify this association on a continuous scale, the 260 test proteins were further divided into 10 approximately equal-sized pLDDT bins, each containing 26 proteins. The points in Fig.7B represent the mean pLDDT and observed accuracy within each bin, whereas the fitted curve and its 95% confidence band were estimated from protein-level binary correctness outcomes for all 260 samples using logistic regression. The probability of correct classification increased with mean pLDDT, although the wider confidence band in the low-pLDDT region indicated greater estimation uncertainty. After adjusting for the true class label and aligned protein length, each 10-point increase in mean pLDDT was associated with a 79.8% increase in the odds of correct classification (OR = 1.798, 95% CI: 1.242-2.602, p=0.002). Consistently, correctly classified proteins had a higher mean pLDDT than incorrectly classified proteins (79.88 vs 71.54; Mann-Whitney U = 3522.0, p = 0.029, BH--FDR-adjusted q = 0.044, Cliff's δ = 0.271). Together, these results demonstrate a statistically significant positive association between predicted structural confidence and classification performance.

Despite this association, the model retained good predictive performance for lower-confidence structures: the accuracy was 81.82% in the Very low group and approximately 84% in the lowest-pLDDT quantile bin. Together with the performance degradation observed after removing the sequence branch in the ablation study, these findings support the complementary roles of sequence and structural information. In particular, the sequence branch may provide additional discriminative cues when structural confidence is limited, thereby improving the model’s robustness to uncertainty in predicted structures.

4) Newly added Fig 7.

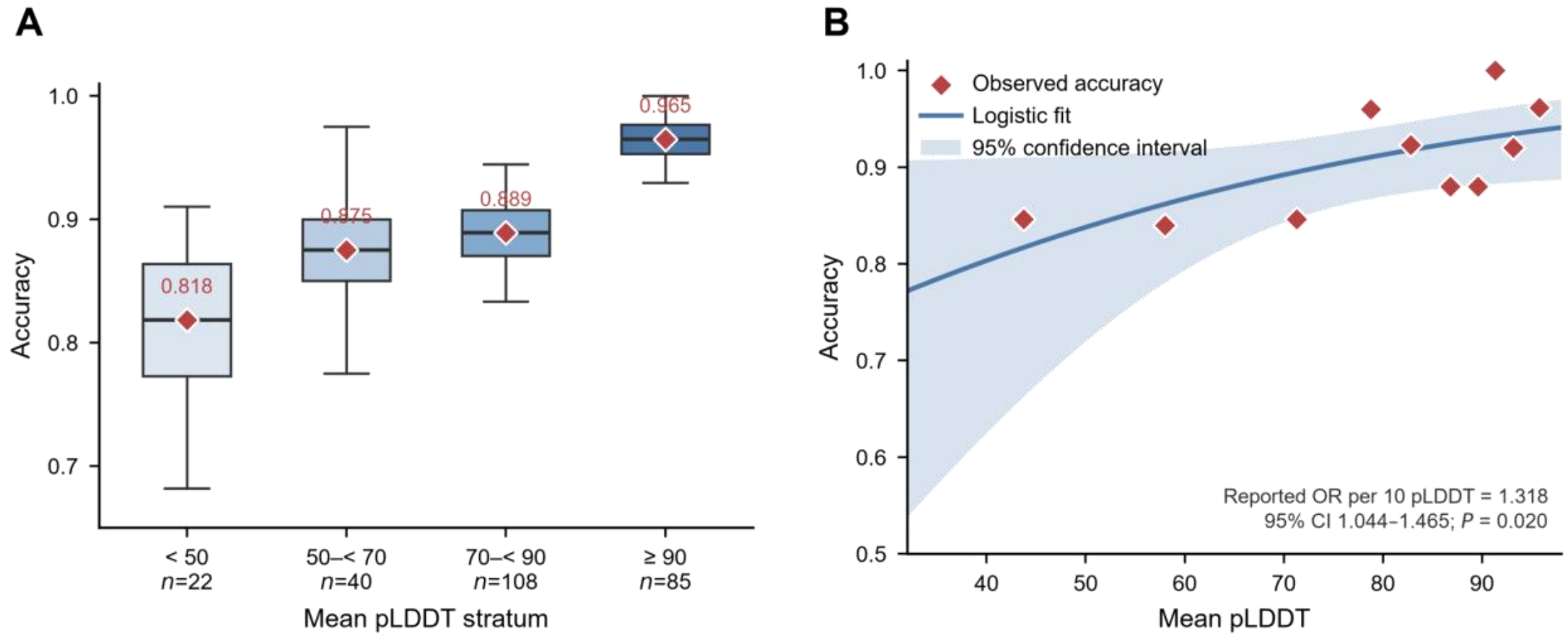

Fig 7. Model performance across ESMFold confidence levels. A: Classification accuracy across four structural-confidence groups defined by the mean protein-level pLDDT: Very low (<50), Low (50-<70), Confident (70-<90), and Very high (≥90). Error bars indicate 95% confidence intervals. B: Continuous association between mean pLDDT and classification accuracy. The 260 test proteins were divided into 10 approximately equal-sized bins, each containing 26 proteins. Black points represent the mean pLDDT and observed accuracy within each bin. The red dashed curve and gray shaded 95% confidence band were estimated by logistic regression using the protein-level correct/incorrect outcomes of all 260 test samples rather than the binned observations. After adjustment for the true class label and aligned protein length, each 10-point increase in mean pLDDT was associated with higher odds of correct classification (OR = 1.798, 95% CI: 1.242-2.602, p = 0.002).

5) To clearly visualize these structure-level validation results, we have added S5 Fig to the revised Supplementary Materials, which presents the relationships of mean pLDDT with TM-score and RMSD, together with a comparison of TM-scores between low- and high-confidence ESMFold predictions.

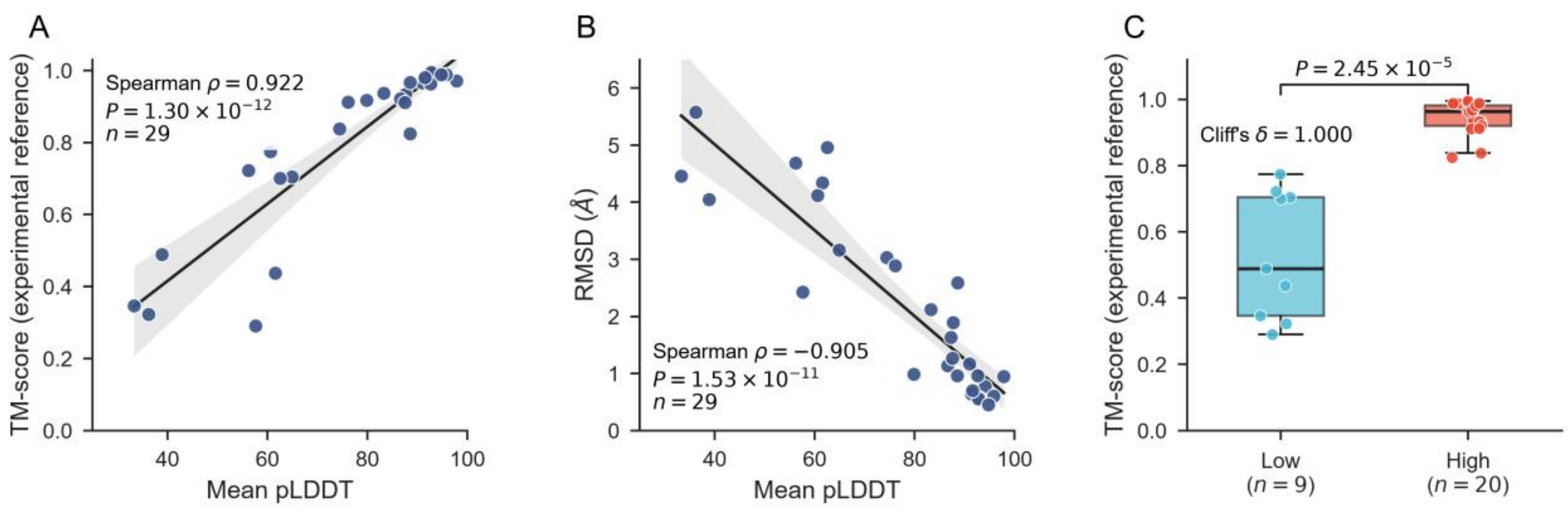

S5 Fig. Comparison of ESMFold-predicted structures with experimentally resolved protein structures. A: Mean pLDDT versus TM-score. B: Mean pLDDT versus RMSD. C: Comparison of TM-scores between low-confidence (pLDDT < 70) and high-confidence (pLDDT ≥ 70) predictions. Each point represents one protein; statistical results are shown in the figure.

4. ***The manuscript compares GeoEPred with state-of-the-art methods using predictions retrieved from official web servers, but it remains unclear whether all models were evaluated under identical datasets, thresholds, and preprocessing conditions. The authors should ensure a fair comparison by describing the evaluation protocol in detail or by reimplementing the competing methods under standardized conditions.***

Response: We sincerely thank the reviewer for raising this important concern regarding the fairness of model comparison. We fully agree that the test datasets, input preprocessing procedures, classification thresholds, and performance evaluation criteria should be clearly described to ensure rigorous and fair comparisons across different methods. In response to the reviewer's valuable suggestion, we retrained and evaluated our method using the datasets adopted by the comparison models. We have also refined and expanded the description of the evaluation procedure in the revised manuscript.

We carefully examined the availability of source code, model weights, and datasets for all competing methods. Because the studies on binary classification models provided only web servers and did not release complete training code or reusable model weights, these models could not be retrained under exactly the same computational conditions. To make the comparison as fair and standardized as possible, we therefore conducted additional dataset-matched benchmark experiments. For each benchmark, GeoEPred was trained from scratch using only the corresponding published training set, while the associated independent test set was kept completely separate and used exclusively for final evaluation. No samples from the independent test set were used for model training or model selection. We then compared the final performance of GeoEPred with the performance reported for the corresponding method in the original publication under the same benchmark setting.

As shown in Table 6, GeoEPred achieved performance comparable to or better than that of the competing models on their corresponding datasets. Notably, our model performed particularly well on the T6SE datasets, although its performance was slightly lower on the T4SEpp dataset. In light of your observation in Comment 3 that structural confidence may influence model performance, we further analyzed the structural confidence of the samples in these datasets (S14 Table). We found that the Bastion6 and BastionX_T6SE training sets had the highest mean structural confidence scores, with mean pLDDT values of 86.02 and 85.47, respectively, together with relatively small standard deviations of 12.38 and 12.65. These results suggest that the structural features in these training sets are of higher and more consistent quality, which may enable the model to learn more reliable structural patterns. In contrast, the T4SEpp training set had a lower mean pLDDT value of 80.78 and a larger standard

deviation of 21.18, suggesting that it contains a greater proportion of proteins with low-confidence predicted structures or intrinsically disordered regions. These differences in training data quality may contribute substantially to the observed variation in model performance across datasets. This analysis further supports your important insight that confidence in predicted protein structures can influence structure-based representation learning. We have incorporated this analysis into the revised manuscript.

In the comparison with existing methods described previously, we provided the following clarification. GeoEPred, DeepSecE, and MoCETSE were trained under a unified experimental setting using the DeepSecE dataset, while retaining the configurations reported in their respective original studies. For the binary classification models, because their source code was not publicly available, predictions were obtained using their publicly accessible web servers, which we considered the most reliable means of preserving the performance reported in the original studies. All models were subsequently evaluated on the same benchmark dataset containing multiple types of effector proteins, which has been widely used in this field to ensure consistency across comparisons. Because a single genome may encode multiple classes of effector proteins, this evaluation setting more closely reflects practical whole-genome prediction scenarios and further demonstrates the ability of GeoEPred to simultaneously identify different classes of effector proteins.

We sincerely thank the reviewer again for this rigorous comment. These comments prompted us to clarify the evaluation protocol and to conduct additional dataset-specific experiments, thereby improving the fairness, transparency, and reproducibility of the comparative evaluation.

The following content provides supplementary additions to the main text.

1) New content has been added to the subsection “Comparison with state-of-the-art model ” (Page 15, Lines 463–470).

To ensure a fair comparison, we trained and evaluated GeoEPred using the same training datasets as those reported for the corresponding binary classification models. Across seven benchmark datasets covering T3SE (two datasets), T4SE (three datasets), and T6SE (two datasets), GeoEPred achieved performance comparable to that of the existing SOTA models on six of the seven test sets (Table 6). To further investigate the performance differences observed across datasets, we analyzed the corresponding pLDDT scores for each dataset (S14 Table). The results showed that GeoEPred tended to perform better on datasets with higher overall structural prediction quality, suggesting that its predictive performance may, to some extent, be influenced by the quality of the predicted input structures.

2) Newly added Table 6.

Table 6. This study compares GeoEPred with state-of-the-art (SOTA) binary classification models for T3SE, T4SE, and T6SE prediction using the same training and evaluation datasets reported in the corresponding studies.

Compared model	Task	Method	Performance metrics				
			REC	PR	ACC	F1	MCC
Bastion3	T3SE	GeoEPred	0.941	**0.976**	**0.959**	0.958	**0.918**
		Bastion3	**0.954**	0.963	0.958	0.958	0.917
T3SEpp	T3SE	GeoEPred	**0.995**	**0.798**	**0.858**	**0.886**	**0.735**
		T3SEpp	0.929	0.796	0.829	0.857	0.659
Bastion4	T4SE	GeoEPred	**1.000**	0.880	**0.976**	0.935	**0.924**
		Bastion4	0.967	**0.942**	0.953	**0.954**	0.907
T4SEpp	T4SE	GeoEPred	0.870	0.963	0.981	0.913	0.905
		T4SEpp	**0.900**	**1.000**	**0.988**	**0.947**	**0.942**
BastionX	T4SE	GeoEPred	0.923	**0.978**	**0.951**	**0.949**	**0.904**
		BastionX	**1.000**	0.882	0.933	0.938	0.874
Bastion6	T6SE	GeoEPred	0.950	**0.968**	**0.992**	**0.958**	**0.954**
		Bastion6	**1.000**	0.465	0.895	0.635	0.642
BastionX	T6SE	GeoEPred	0.979	**0.976**	**0.978**	**0.978**	**0.955**
		BastionX	**1.000**	0.935	0.975	0.967	0.933

3) Newly added S14 Table.

S14 Table. Sample-level pLDDT statistics across different benchmark datasets, ranked by the mean pLDDT of the training set.

Rank	Prediction Task	Dataset	Training Set pLDDT (Mean ± SD)	Test Set pLDDT (Mean ± SD)
1	T6SE	Bastion6	86.02 ± 12.38	83.82 ± 14.04
2	T6SE	BastionX T6SE	85.47 ± 12.65	82.19 ± 13.03
3	T4SE	BastionX T4SE	85.19 ± 13.61	74.97 ± 18.25
4	T4SE	Bastion4	81.71 ± 16.02	84.10 ± 13.21
5	T3SE	Bastion3	81.35 ± 16.45	75.75 ± 17.65
6	T4SE	T4SEpp	80.78 ± 21.18	84.20 ± 13.88
7	T3SE	T3SEpp	75.54 ± 17.99	74.54 ± 17.99

5. ***The manuscript introduces several architectural components, including contrastive learning, feature tokenization, and adaptive attention pooling, but the contribution of each individual module is not fully isolated. The authors should include additional ablation experiments that independently remove each component to quantify its unique contribution to the overall performance.***

Response: We sincerely thank the reviewer for this important and constructive suggestion. We fully agree that independently evaluating each architectural component is necessary to clarify its contribution to the overall performance. In response, we conducted additional controlled ablation experiments in which

contrastive learning, feature tokenization, and adaptive attention pooling were removed individually from the complete model, while all remaining components were kept unchanged.

Specifically, we evaluated the following four configurations: (1) the complete multimodal model; (2) the model without contrastive learning, in which the contrastive loss was removed and training was performed using only the classification objective; (3) the model without feature tokenization, in which modality-specific representations were integrated by direct concatenation while retaining the original encoders and downstream classifier; and (4) the model without adaptive attention pooling, in which the learned attention-weighted pooling operation was replaced with parameter-free mean pooling. All models were trained using the same preprocessing pipeline, optimization settings, early-stopping criteria, random seeds, and data splits. We also fully recognize the importance of the reviewer's second point. Accordingly, we performed statistical analysis using a paired stratified Bootstrap procedure (N = 1000) and estimated the 95% confidence intervals and corresponding p-values for differences in both overall and class-specific performance metrics. The corresponding statistical results, including class-specific performance comparisons, confidence intervals, and p-values, have been incorporated into the revised manuscript.

As shown in Table 3, removing contrastive learning was associated with decreases of 3.46 percentage points in ACC (95% CI: 0.0038-0.0692, p = 0.032) and 4.85 percentage points in MCC (95% CI: 0.0094-0.0910, p = 0.014). A decrease of 3.00 percentage points was also observed for F1, although the corresponding p-value was slightly above 0.05 (95% CI: 0.00002-0.0602, p = 0.0519). Replacing adaptive attention pooling with mean pooling was associated with decreases of 3.85 percentage points in ACC (95% CI: 0.0038-0.0769, p = 0.038) and 5.32 percentage points in MCC (95% CI: 0.0083-0.1014, p = 0.022). Feature tokenization showed effects across multiple evaluation metrics. Compared with the variant without feature tokenization, GeoEPred showed improvements of 5.00 percentage points in ACC (95% CI: 0.0231-0.0808, p = 0.002), 4.61 percentage points in F1 (95% CI: 0.0181-0.0764, p = 0.002), 6.83 percentage points in MCC (95% CI: 0.0303-0.1085, p = 0.002), and 3.94 percentage points in AUPRC (95% CI: 0.0112-0.0888, p = 0.004). These results indicate that feature tokenization contributes to classification performance and precision--recall performance under class imbalance. Overall, the ablation results are consistent with the intended roles of the three components: contrastive learning contributes to discriminative representation learning, feature tokenization supports the integration of sequence and structural features, and adaptive attention pooling contributes to the aggregation of informative token-level representations.

We have incorporated these additional experiments into the revised manuscript

and added the corresponding results to Table 3 and S7 Table. The descriptions of the three components and their respective contributions have also been revised to avoid unsupported or overly strong conclusions. We once again sincerely thank the reviewer for this rigorous suggestion. It prompted us to provide a more controlled and transparent evaluation of the proposed architectural components, thereby strengthening the methodological rigor and interpretability of our study.

The following content provides supplementary additions to the main text.

1) Corresponding revisions have been made in the "Model component evaluation" section (Page 11–12, Lines 343–362).

We conducted a series of ablation experiments to evaluate the contribution of each core component to the model's overall performance. Specifically, we compared the complete GeoEPred model with three variants, each lacking one component: (1) a model combining adaptive pooling and feature tokenization but excluding contrastive learning; (2) a model combining feature tokenization and contrastive learning but excluding adaptive pooling; and (3) a model combining adaptive pooling and contrastive learning but excluding feature tokenization.

As shown in Table 3, in five-fold cross-validation, the model combining adaptive attention pooling and contrastive learning achieved an accuracy (ACC) of 0.861 (95% CI: 0.842-0.880) and an F1 score of 0.829 (95% CI: 0.799-0.859). Upon introducing feature tokenization, the accuracy (ACC) and F1 score improved to 0.899 (95% CI: 0.878-0.920) and 0.88 (95% CI: 0.853-0.907), respectively. When adaptive attention pooling was replaced by mean pooling, the accuracy and F1 score dropped to 0.886 (95% CI: 0.868-0.904) and 0.863 (95% CI: 0.835-0.891), respectively. On the independent test set, removing contrastive learning resulted in decreases of 3.00% (95% CI: 0.00002-0.0602, $p = 0.0519$) in F1 and 4.85% (95% CI: 0.0094-0.0910, $p = 0.0140$) in MCC. Regarding AUPRC - a metric particularly informative under class imbalance - GeoEPred achieved a score of 0.9146, representing a 3.94% improvement over the model without feature tokenization (0.8753; 95% CI: 0.0112-0.0888, $p = 0.0040$). F1 and MCC also improved by 4.61% (95% CI: 0.0181-0.0764, $p = 0.0020$) and 6.83% (95% CI: 0.0303-0.1085, $p = 0.0020$), respectively (S7 Table). These results indicate that feature tokenization helps maintain precision-recall performance and enhances the quality of probability predictions for the minority class.

2) Newly added Table 3.

Table 3. Performance comparison of model variants in the ablation study using ACC, F1, and AUPRC on cross-validation and independent test sets.

Model Variant	ACC		F1		AUPRC	
	CV	Ind	CV	Ind	CV	Ind
w/o Contrastive Learning	0.881	0.889	0.851	0.854	0.901	0.887
w/o Adaptive Attention Pooling	0.886	0.885	0.863	0.851	0.899	0.890
w/o Feature Tokenization	0.861	0.873	0.829	0.838	0.864	0.875
Full Model (Ours)	**0.899**	**0.923**	**0.880**	**0.884**	**0.907**	**0.915**

3) Newly added S7 Table.

S7 Table. Performance gains, 95% confidence intervals, and statistical significance of the full model compared with different ablated model variants.

Model Variant	Metric	Δ	95% CI	*p*-values
w/o Contrastive Learning	ACC	+0.0346	[0.0038, 0.0692]	0.0320
	F1	+0.0300	[0.00002, 0.0602]	0.0519
	MCC	+0.0485	[0.0094, 0.0910]	0.0140
	AUC	+0.0064	[-0.0047, 0.0217]	0.2917
	AUPRC	+0.0278	[-0.0043, 0.0686]	0.1019
w/o Adaptive Attention Pooling	ACC	+0.0385	[0.0038, 0.0769]	0.0380
	F1	+0.0338	[-0.0071, 0.0816]	0.0939
	MCC	+0.0532	[0.0083, 0.1014]	0.0220
	AUC	+0.0034	[-0.0077, 0.0134]	0.5554
	AUPRC	+0.0249	[-0.0091, 0.0789]	0.1379
w/o Feature Tokenization	ACC	+0.0500	[0.0231, 0.0808]	0.0020
	F1	+0.0461	[0.0181, 0.0764]	0.0020
	MCC	+0.0683	[0.0303, 0.1085]	0.0020
	AUC	+0.0007	[-0.0118, 0.0102]	0.8352
	AUPRC	+0.0394	[0.0112, 0.0888]	0.0040

6. ***The manuscript claims strong generalization capability; however, the evaluation is limited to benchmark datasets derived from similar sources. The authors should validate the model on completely independent external datasets or newly collected bacterial genomes to better demonstrate real-world applicability and generalization. Refer to this article: PMID-39657879 to improve the methodology, result, and discussion sections which will be more beneficial for the readership of the article and journal.***

Response: We sincerely thank the reviewer for this important and insightful comment. We agree that evaluating the model on completely unseen data is essential for assessing its generalization capability. We also apologize that our previous description of the training and testing procedures, as well as the presentation of the comparative results, was not sufficiently clear. We consider the five-fold cross-validation framework adopted in the study described in PMID: 39657879 to be a standard approach for model performance evaluation in bioinformatics. Following the

reviewer's suggestion, we adopted the evaluation protocol used in that study and cited the corresponding article in the revised "Data Evaluation Procedure" section. In the revised performance comparison section, we report both the model performance obtained from five-fold cross-validation and the performance of the final selected model on the independent test set, thereby evaluating both predictive performance and generalization ability.

Specifically, we performed five-fold cross-validation and further divided the training portion of each fold into a validation subset. Model and checkpoint selection was based exclusively on the F1 score obtained on the validation subset, after which the selected model was evaluated on the independent test set. The independent test set was not used for model training, hyperparameter optimization, checkpoint selection, or any other stage of model development. In response to the reviewer's second point, we also conducted additional statistical significance analyses under this evaluation framework to assess the robustness and statistical reliability of the observed performance differences.

To further investigate the performance of GeoEPred on entirely unseen genomes, we conducted an additional evaluation using experimentally validated effector proteins reported from recently sequenced bacterial genomes, with particular emphasis on genomes that were not included in our original dataset. Specifically, we collected experimentally validated effector proteins from *Aeromonas schubertii ATCC 43700* [2], *Aeromonas jandaei DSM 7311* [3], *Xanthomonas hortorum pv. pelargonii 305* [4], and *Aeromonas veronii C4* [5]. Among these, the genomes of *A. veronii C4* and *X. hortorum pv. pelargonii 305* were newly sequenced and released after 2023. Although experimentally validated effectors from other secretion system types have also been reported during the past two years, the corresponding genomes were sequenced earlier and homologous sequences from those genomes were already represented in our dataset. We therefore did not include those samples in this additional evaluation. After excluding sequences sharing more than 60% sequence identity with proteins in the training set, we retained 9 experimentally validated T3SEs and 7 experimentally validated T6SEs. On this small, genome-level unseen test set, GeoEPred showed encouraging generalization performance, correctly identifying 8 of the 9 T3SEs and all 7 T6SEs, with generally high prediction confidence, as shown in the table below. These preliminary results suggest that GeoEPred has promising generalization potential; however, given the limited number of newly available samples that satisfied our stringent inclusion criteria, we have interpreted these findings cautiously in the revised manuscript.

We sincerely thank the reviewer again for the careful and professional evaluation. Your suggestions helped us refine the model evaluation framework in a more rigorous

manner, incorporate statistical significance analyses, and further examine the practical generalizability of GeoEPred using data from newly sequenced genomes. These revisions have substantially strengthened the overall rigor and academic value of the study.

Reference:

[1] Daroch A, Purohit R. MDbDMRP: A novel molecular descriptor-based computational model to identify drug-miRNA relationships. Int J Biol Macromol. 2025;287:138580. doi:10.1016/j.ijbiomac.2024.138580.

[2] Michova H, Pliva J, Jirsova A, Jurnecka D, Kamanova J. Cytotoxicity induced by Aeromonas schubertii is orchestrated by a unique set of type III secretion system effectors. Vet Res. 2025;56(1):113. doi:10.1186/s13567-025-01548-2.

[3] Fridman CM, Keppel K, Rudenko V, Altuna-Alvarez J, Albesa-Jové D, Bosis E, Salomon D.A new class of type VI secretion system effectors can carry two toxic domains and are recognized through the WHIX motif for export. PLoS Biol. 2025;23(3):e3003053. doi:10.1371/journal.pbio.3003053.

[4] Wagner N, Ben-Meir D, Teper D, Pupko T. Complete genome sequence of an Israeli isolate of Xanthomonas hortorum pv. pelargonii strain 305 and novel type III effectors identified in Xanthomonas. Front Plant Sci. 2023;14:1155341. doi:10.3389/fpls.2023.1155341.

[5] Jiang X, Li H, Ma J, Li H, Ma X, Tang Y, Li J, Chi X, Deng Y, Zeng S, Liu Z. Role of Type VI secretion system in pathogenic remodeling of host gut microbiota during Aeromonas veronii infection. ISME J. 2024;18(1):wrae053. doi:10.1093/ismejo/wrae053.

The following content provides supplementary additions to the main text.

1) We have added a "Model Training" section to clearly explain the methods we use to train and evaluate the model. (Page 8, Lines 228–233).

GeoEPred was trained using stratified five-fold cross-validation, with all experiments repeated using five different random seeds (42, 52, 62, 72, and 82). For each random seed, the available training data were partitioned into training and validation subsets within each fold, and the model checkpoint achieving the highest macro-F1 score on the validation set was selected and retained. The independent test set remained completely isolated throughout model training, hyperparameter determination, and model selection, and was used solely for the final performance evaluation [37].

2) We have updated Table 2 to provide a clearer and more direct comparison of the performance of GeoEPred and the corresponding single-modality models in both five-fold cross-validation and the independent test set.

Table 2. Performance evaluation of multimodal feature ablation.

Feature Type	ACC		F1		AUPRC	
	CV	Ind	CV	Ind	CV	Ind
Geometric Priors	0.615	0.527	0.541	0.459	0.561	0.454

Sequence only	0.681	0.723	0.639	0.664	0.681	0.683
Sequence Semantics	0.877	0.881	0.854	0.846	0.893	0.907
GeoEPred	**0.899**	**0.923**	**0.880**	**0.884**	**0.907**	**0.915**

3) In accordance with your fifth suggestion, we have added Table 3 to provide a comprehensive comparison of the performance of different module ablation settings in both five-fold cross-validation and independent testing.

Table 3. Performance comparison of model variants in the ablation study using ACC, F1, and AUPRC on cross-validation and independent test sets.

Model Variant	ACC		F1		AUPRC	
	CV	Ind	CV	Ind	CV	Ind
w/o Contrastive Learning	0.881	0.889	0.851	0.854	0.901	0.887
w/o Adaptive Attention Pooling	0.886	0.885	0.863	0.851	0.899	0.890
w/o Feature Tokenization	0.861	0.873	0.829	0.838	0.864	0.875
Full Model (Ours)	**0.899**	**0.923**	**0.880**	**0.884**	**0.907**	**0.915**

4) We have revised Table 4 to provide a clearer and more comprehensive comparison of the performance of the feature tokenization strategy and other fusion strategies in both five-fold cross-validation and independent testing.

Table 4. Performance evaluation metrics for different fusion strategies.

Method	ACC		F1		AUPRC	
	CV	Ind	CV	Ind	CV	Ind
Concatenation	0.884	0.889	0.860	0.848	0.854	0.863
Cross-Attention Fusion	0.886	0.908	0.863	0.862	0.901	0.873
Feature Tokenization	**0.899**	**0.923**	**0.880**	**0.884**	**0.907**	**0.915**

5) We have revised Table 5 to provide a clearer and more comprehensive comparison between our proposed framework and conventional methods in terms of their performance in both five-fold cross-validation and independent testing.

Table 5. Comparison of the proposed method with baseline methods.

Method	ACC		F1		AUPRC	
	CV	Ind	CV	Ind	CV	Ind
Traditional Machine Learning						
SVM	0.892	0.908	0.854	0.878	0.882	0.885
Random Forest	0.882	0.896	0.786	0.806	0.825	0.831
Gradient Boosting	0.865	0.869	0.770	0.749	0.805	0.794
KNN	0.857	0.831	0.809	0.804	0.828	0.821
Naive Bayes	0.804	0.815	0.744	0.757	0.768	0.778
Decision Tree	0.691	0.692	0.603	0.611	0.642	0.642
LDA	0.578	0.619	0.611	0.641	0.602	0.632
Deep Learning Method						
BiLSTM_Attention	0.862	0.900	0.852	0.864	0.879	0.885
MLP	0.875	0.900	0.856	0.850	0.880	0.871

GRU	0.884	0.900	0.848	0.860	0.883	0.886
Vanilla_Transformer	0.866	0.881	0.854	0.852	0.871	0.891
Our method						
GeoEPred	**0.899**	**0.923**	**0.880**	**0.884**	**0.907**	**0.915**

6) We added S18 Table to provide a more comprehensive evaluation of GeoEPred on recently experimentally validated effector proteins.

S18 Table. GeoEPred predictions for recently experimentally validated bacterial effector proteins.

Effector	Strain	Secretion system	GeoEPred prediction	Confidence	Reference
AopI	*Aeromonas schubertii ATCC 43700*	T3SE	T3SE	0.99202687	[3]
AopJ	*Aeromonas schubertii ATCC 43700*	T3SE	T3SE	0.9745239	[3]
AopL	*Aeromonas schubertii ATCC 43700*	T3SE	T3SE	0.9941506	[3]
AopT	*Aeromonas schubertii ATCC 43700*	T3SE	T3SE	0.99421096	[3]
AopU	*Aeromonas schubertii ATCC 43700*	T3SE	T3SE	0.993871	[3]
TseI-like	*Aeromonas jandaei DSM 7311*	T6SE	T6SE	0.99375856	[4]
Tle1-like	*Aeromonas jandaei DSM 7311*	T6SE	T6SE	0.9903679	[4]
Awe1	*Aeromonas jandaei DSM 7311*	T6SE	T6SE	0.990186	[4]
DUF3289	*Aeromonas jandaei DSM 7311*	T6SE	T6SE	0.98906076	[4]
XopBB	*X. hortorum pv. pelargonii 305*	T3SE	T3SE	0.99400467	[5]
XopBC	*X. hortorum pv. pelargonii 305*	T3SE	T3SE	0.99064296	[5]
XopBD	*X. hortorum pv. pelargonii 305*	T3SE	T3SE	0.99371815	[5]
XopBE	*X. hortorum pv. pelargonii 305*	T3SE	Non-Effector	0.9620185	[5]
Tse1	*A. veronii C4*	T6SE	T6SE	0.99262416	[6]
Tse2	*A. veronii C4*	T6SE	T6SE	0.9911902	[6]
Tse3	*A. veronii C4*	T6SE	T6SE	0.9931722	[6]

7. The manuscript reports nearly perfect performance for some prediction tasks, particularly T6SE, which raises concerns regarding potential dataset bias or overfitting. The authors should discuss this unusually high performance, provide additional validation analyses, and examine whether class similarity, sample redundancy, or dataset composition contributed to these results.

Response: We sincerely thank the reviewer for highlighting this important issue. In response to this comment, we re-examined the dataset partitioning and model-training procedure and retrained the model. We specifically checked for potential dataset overlap and information leakage among the training, validation, and independent test sets. This re-examination confirmed that the three datasets were mutually exclusive and that no information leakage was identified.

During the reanalysis, we observed potential signs of overfitting during the later stages of training in Folds 2 and 5. We therefore introduced an early-stopping strategy using the validation-set F1 score as the monitoring metric, with a patience of five epochs. Training was terminated when the validation F1 score ceased to improve, and the checkpoint corresponding to the best validation performance was retained. As shown in the revised figure, the selected stopping points are indicated by gray vertical dashed lines, including Epoch 19 for Fold 2 and Epoch 9 for Fold 5.

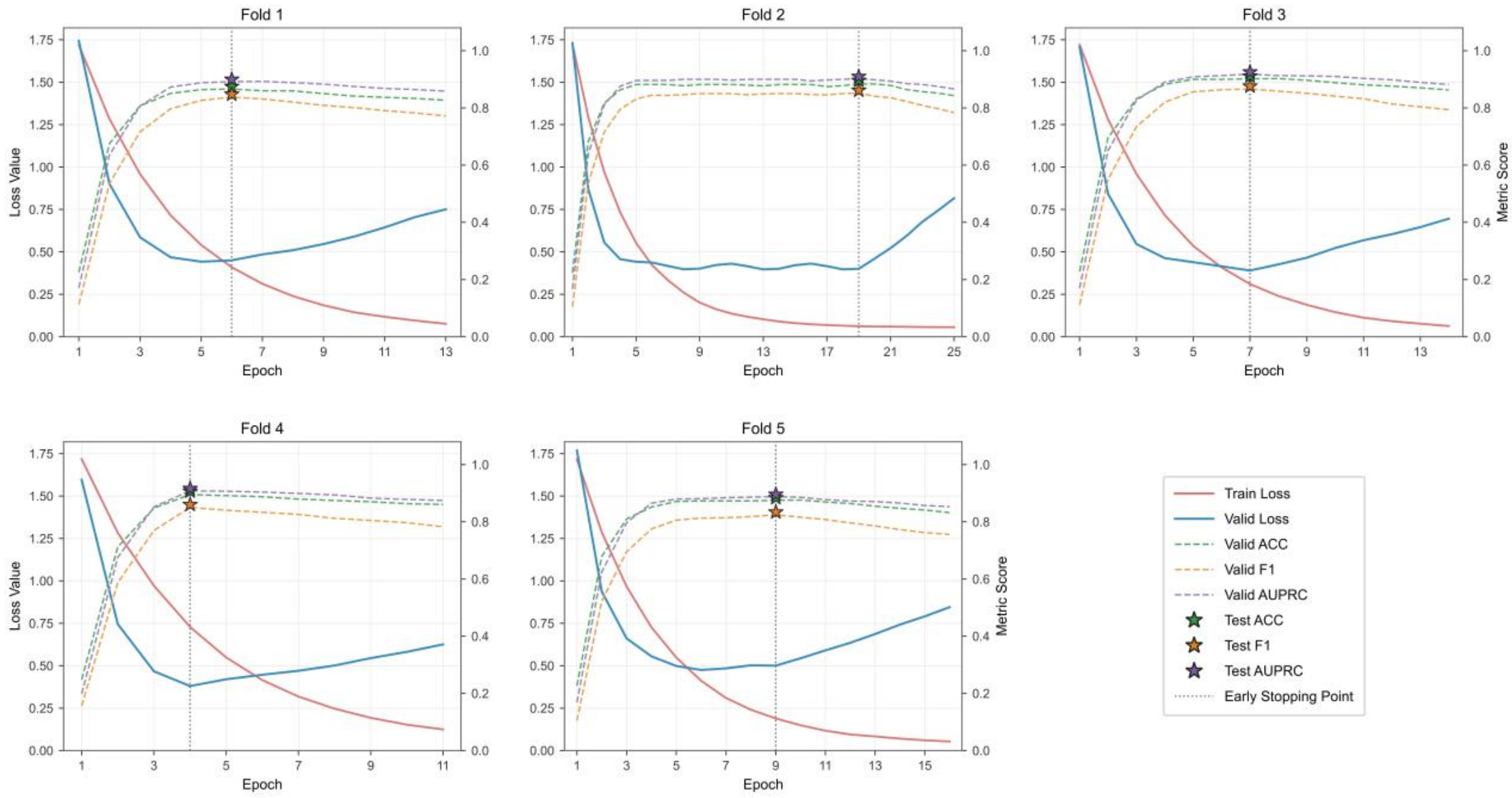

We then reevaluated the models selected using this strategy. At the corresponding checkpoints, the ACC, F1, and AUPRC values on the independent test set were 0.923, 0.884, and 0.915, respectively. The class-wise performance metrics were also recalculated. The performance of the T6SE class decreased after retraining, with ACC, F1, and MCC changing from 1.000 to 0.989, 0.930, and 0.927, respectively. The recalculated results showed relatively strong performance across multiple evaluation metrics.

We have accordingly revised Fig. 6 and updated the relevant sections of the manuscript to provide additional details on the early-stopping strategy, the criterion used for model selection, the revised class-wise results, and the corresponding independent-test results. We sincerely thank the reviewer again for this valuable comment, which prompted us to re-examine the training procedure and present the model-selection process and evaluation results more transparently.

The following content provides supplementary additions to the main text.

1) Corresponding revisions have been made in the "Comparison with state-of-the-art models" section (Page 14, Lines 441–452).

From the overall results, GeoEPred achieved predictive performance comparable to that of current state-of-the-art methods, with a more evident advantage in the T6SE prediction task (Fig.6A-C). In comparison with the multi-class models, GeoEPred achieved comparable predictive performance across most classes (S11 Table). In terms of overall performance, GeoEPred improved accuracy by 2.69% (p = 0.098) and 3.46% (p = 0.048) compared with DeepSecE and MoCETSE, respectively, which were among the better-performing baseline models. For the T6SE class (S12 Table), GeoEPred showed favorable predictive performance, with both threshold-independent metrics, AUC and AUPRC, being significantly higher than those of DeepSecE (AUC: 95% CI: 0.0012-0.0233, p = 0.020; AUPRC: 95% CI: 0.0118-0.3631, p = 0.016). As

shown in S13 Table, GeoEPred also showed a modest improvement in AUC and AUPRC compared with MoCETSE (AUC: Δ = +0.0098, 95% CI: 0.0000-0.0279, p = 0.086; AUPRC: Δ = +0.0098, 95% CI: 0.0000-0.1457, p = 0.076). These results suggest that GeoEPred has good discriminative capability for T6SE prediction and may have an advantage in identifying fine-grained features associated with T6SEs.

2) Updated Fig 6.

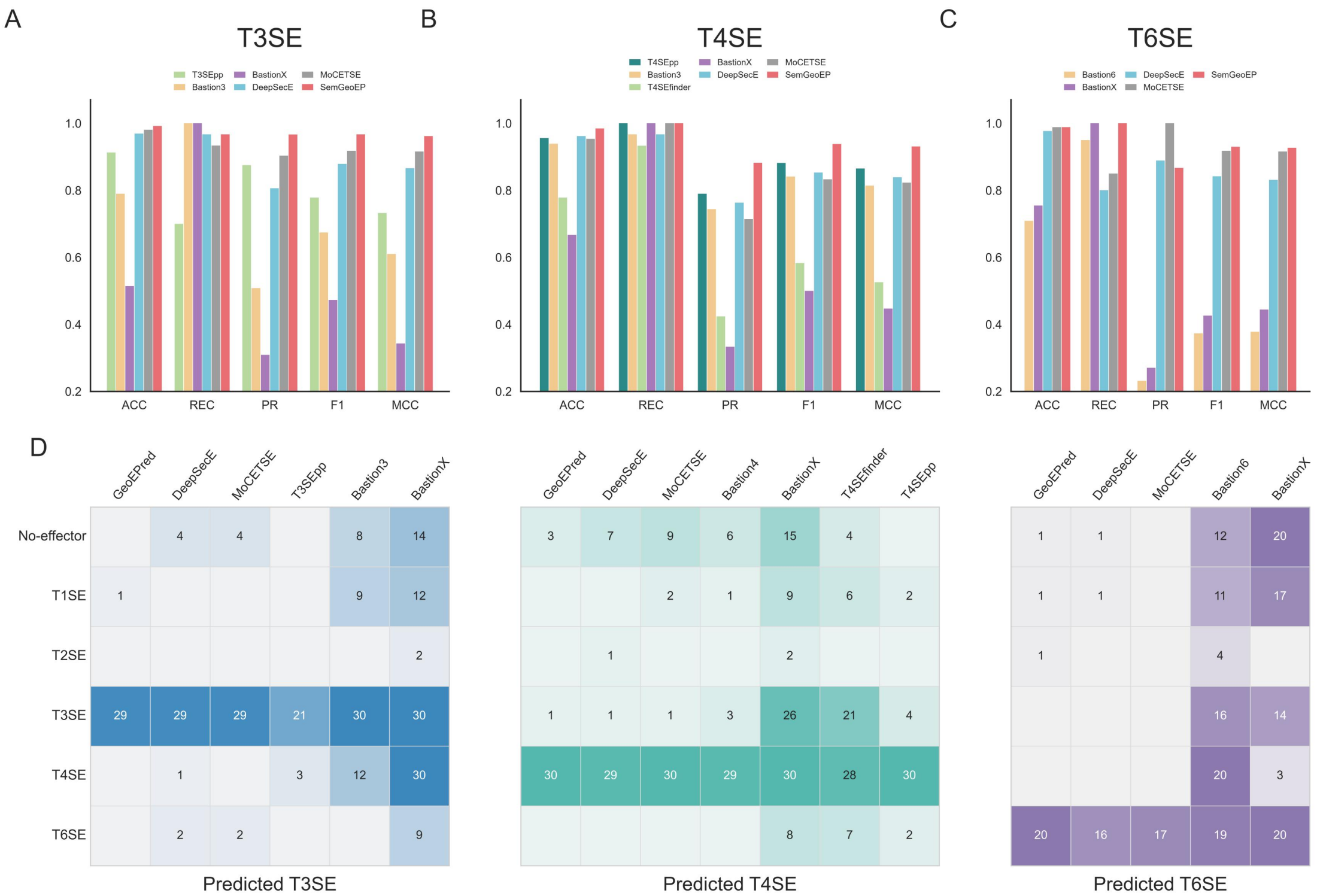

Fig 6. Performance evaluation of GeoEPred in comparison with mainstream models. A-C: Performance comparison of GeoEPred with mainstream prediction models on T3SE (A), T4SE (B), and T6SE (C) prediction tasks. D: Comparison of true positive and false positive predictions from different models on the independent test set (Dataset S2), which contains 150 non-effectors, 20 T1SEs, 10 T2SEs, 30 T3SEs, 30 T4SEs, and 20 T6SEs. The heatmaps illustrate the distribution of predicted samples across various effector protein types.

8. ***The manuscript would benefit from a more comprehensive description of the training procedure and computational settings. The authors should report essential implementation details such as optimizer, learning rate schedule, batch size, early stopping criteria, number of epochs, random seed, hardware configuration, and computational time to facilitate reproducibility.***

Response: We sincerely thank the reviewer for this important suggestion. We fully agree that a more comprehensive description of the training procedure and computational environment would improve the transparency and reproducibility of

our study. In response to this suggestion, we carefully reviewed the source code, configuration files, and experimental records. We have now added detailed information on the model training procedure and computational settings to the Methods section of the revised manuscript, including the optimizer, learning-rate scheduling strategy, batch size, early-stopping criteria, number of training epochs, random seeds, hardware configuration, and computational time.

Specifically, the model was optimized using AdamW, with an initial learning rate of 2×10^{-5} (for the sequence encoder), 3×10^{-4} (for the structure encoder), and 1×10^{-4} (for other modules), and a weight decay of 1×10^{-4}. During training, the learning rate was dynamically adjusted using a cosine annealing with warm restarts scheduler with an initial period of T_0= 20 and a multiplier of T_{mult}= 2. The batch size was set to 32, and the model was trained for a maximum of 30 epochs. We employed an early-stopping strategy based on the validation macro-F1 score. Training was terminated if this metric failed to improve for 5 consecutive epochs, and the model weights achieving the best macro-F1 score on the validation set were retained. A fixed random seed of 42 (with fold-specific offsets from 43 to 47) was used in all experiments to control randomness in data partitioning, parameter initialization, and mini-batch sampling.

We once again sincerely thank you for your rigorous and meticulous review, as well as for your strong emphasis on research reproducibility. Your careful attention to the details of the model implementation prompted us to present the experimental setup and training process in a more systematic and comprehensive manner, thereby further enhancing the transparency, rigor, and reproducibility of this study.

The following content provides supplementary additions to the main text.

1) To address this concern, we have added a new subsection entitled "Model training" to Section 2 of the revised manuscript (Page 8–9, Lines 228–250).

GeoEPred was trained using stratified five-fold cross-validation, with all experiments repeated using five different random seeds (42, 52, 62, 72, and 82). For each random seed, the available training data were partitioned into training and validation subsets within each fold, and the model checkpoint achieving the highest macro-F1 score on the validation set was selected and retained. The independent test set remained completely isolated throughout model training, hyperparameter determination, and model selection, and was used solely for the final performance evaluation [37].

The model was implemented in PyTorch v2.8.0 and executed on an NVIDIA A100 GPU with 40 GB of memory. The software environment consisted of Python 3.12.9 and CUDA 12.8. Model parameters were initialized using Xavier initialization and optimized using AdamW with a weight decay of 1×10^{-4}. The initial learning rates were set to 2×10^{-5} for the sequence encoder, 3×10^{-4} for the structure encoder, and

1×10^{-4} for the remaining modules. The learning rates were updated after each epoch using cosine annealing with warm restarts, with an initial cycle length of 20 epochs and a cycle-length multiplier of 2 (T_0= 20 and T_{mult}= 2).

The batch size and maximum number of training epochs were set to 32 and 30, respectively. Early stopping was performed by monitoring the macro-F1 score on the validation set, with a patience of five epochs, and the checkpoint achieving the best validation performance in each fold was retained. Automatic mixed precision and dynamic loss scaling were employed during training, and the global gradient norm was clipped to a maximum value of 1.0.

Class imbalance was addressed using inverse-class-frequency weighted random sampling. Focal loss was adopted as the primary classification objective, with a focusing parameter of 2.0 and a label-smoothing coefficient of 0.05. The sequence-modality dropout probability was set to 0.25, whereas a conventional dropout rate of 0.1 was applied within the sequence Transformer encoder, cross-modal attention module, and Transformer classifier.

Reviewer #3:

The manuscript presents an interesting computational framework and is generally well organized. However, several issues should be addressed to improve the study.

1. In my opinion authors should provide a clearer description of the dataset construction process, including the criteria for sequence inclusion/exclusion and the rationale for selecting the sequence identity threshold.

Response: We sincerely thank the reviewer for raising this important issue. We fully agree that a clearer description of the dataset construction process is essential. Following the reviewer's suggestion, we systematically reviewed and clarified the sequence sources, inclusion and exclusion criteria, redundancy-removal procedures, rationale for selecting the sequence identity threshold, and sequence similarity across different data partitions. We have also provided a more explicit description of the corresponding processing procedures. The complete data-processing workflow, parameter settings, and validation results have now been added to the revised manuscript and Supplementary Materials.

All samples included in our dataset were obtained from publicly available datasets in this field. After integrating these datasets, we followed the dataset construction strategy and criteria adopted in DeepSecE [1]. Specifically, a 60% sequence identity threshold was applied to remove redundant and highly homologous sequences within the training set and between the training and test sets. In addition, we required the aligned region to cover at least 80% of both the shorter and longer sequences. Within each dataset, sequences from all effector classes and the non-effector class were pooled together for redundancy removal, and only one representative sequence was retained from each cluster. This procedure was designed to prevent identical or highly similar protein sequences from being retained in the dataset.

To further exclude highly homologous sequences across datasets, after performing redundancy removal separately within the candidate training set and the independent test set, we additionally used CD-HIT-2D with the same criteria to perform cross-dataset comparison. The redundancy-reduced independent test set was used as the reference set, whereas the candidate training set was used as the set to be filtered. If a sequence in the training set shared at least 60% sequence identity with a sequence in the independent test set, with the aligned region covering at least 80% of both the shorter and longer sequences, that training sequence was removed. During this cross-dataset filtering step, the independent test set remained unchanged, and only highly similar sequences in the training set that could potentially introduce

information leakage were removed.

After the above processing steps, the final training set contained 3,155 sequences, and the independent test set contained 260 sequences. To further verify the independence of the two datasets, we reran CD-HIT-2D using the 60% sequence identity threshold to compare the final training set against the independent test set. No cross-dataset sequence pairs satisfying the predefined sequence identity and alignment coverage criteria were detected. Therefore, no highly similar sequence pairs meeting these criteria remained between the final training set and the independent test set, further supporting their sequence-level independence.

We have added these details to the "Dataset Construction" section of the revised manuscript. S1 Fig now presents the number of sequences in each class before and after redundancy removal, together with the results of the cross-dataset redundancy assessment. To further improve the transparency and reproducibility of this study, we have made the processed datasets and the relevant files generated during data processing publicly available at the following URL:

https://github.com/shouzhensong/GeoEPred.

We sincerely thank the reviewer again for this important comment. These additional analyses and clarifications have enabled us to demonstrate the independence of the datasets more rigorously and have substantially improved the transparency and reproducibility of the study.

Reference:

[1] Zhang Y, Guan J, Li C, Wang Z, Deng Z, Gasser RB, Song J, Ou HY. DeepSecE: A deep-learning-based framework for multiclass prediction of secreted proteins in Gram-negative bacteria. Research (Wash D C). 2023;6:0258. doi:10.34133/research.0258.

The following content constitutes revisions to the main text.

1) Corresponding revisions have been made in the "Dataset construction" section (Page 4, Lines 59–94).

The datasets used for model training and evaluation were compiled from datasets released with established effector prediction methods and from widely used biological databases.

The dataset construction and redundancy-removal procedures were designed with reference to DeepSecE [13], a representative study in the field. Specifically, T1SE and T2SE sequences were obtained from the TSE-ARF dataset [21]. The T3SE and T6SE datasets were compiled by integrating sequences from Bastion3 [7], DeepT3 [22], SecReT6 [23], Bastion6 [8], DeepT3_4 [24], and TSE-ARF [21]. The T4SE dataset was assembled from DeepSecE [13], OPT4e [25], T4SEfinder [26], iT4SE-EP [27], Bastion4 [28], T4SE-XGB [29], TSE-ARF [21], T4SEpp [30], and

DeepT3_4 [24]. Following previous representative studies [31, 32], the negative class was constructed using curated non-secreted proteins and homologous sequences from selected pathogenic bacteria reported in earlier studies. These candidate negative samples had been further screened against multiple bacterial genomes using BLASTp in the original studies, based on predefined thresholds for the *E*-value and sequence identity. To provide an objective assessment of model generalization, we additionally assembled an independent test set. The T3SE, T4SE, T6SE, and non-secreted protein samples were collected from the test datasets released with Bastion3, CNN-T4SE [33], and Bastion6, whereas the T1SE and T2SE samples were randomly selected from the original BastionHub dataset.

To minimize potential overestimation of model performance caused by sequence redundancy, CD-HIT v4.8.1 [33] was applied separately to the candidate training set and the independent test set. A global sequence identity threshold of 60% was used, and the aligned region was required to cover at least 80% of both the shorter and longer sequences. Within each dataset, sequences from all effector and non-effector classes were pooled before clustering, and only one representative sequence was retained from each cluster. Cross-dataset filtering was subsequently performed using CD-HIT-2D under the same identity and coverage criteria. The non-redundant independent test set was used as the reference dataset, and candidate training sequences showing at least 60% global sequence identity to any test sequence were removed. This procedure minimized the risk of information leakage between the training and test sets.

The data sources for each effector type and the corresponding sample numbers after CD-HIT filtering are summarized in S1 Table. The resulting training set comprised 1,575 non-effector proteins, 128 T1SEs, 65 T2SEs, 505 T3SEs, 623 T4SEs, and 259 T6SEs. The independent test set contained 150 non-effector proteins, 20 T1SEs, 10 T2SEs, 30 T3SEs, 30 T4SEs, and 20 T6SEs (S1 Fig A). Protein lengths ranged from fewer than 100 to more than 1,000 amino acids, with most sequences falling between 100 and 700 amino acids (S1 Fig B).

2. Pretrained protein language models and structure prediction methods are used, the authors should clarify whether proteins in the independent test set were represented in the pretraining datasets and discuss the potential impact on performance evaluation.

Response: Thank you very much for raising this thoughtful and academically important question. We fully agree that clarifying the extent of overlap between the independent test set and the pretraining data is essential for accurately defining test-set independence and for objectively interpreting model performance. We

sincerely apologize that this important context was not described in sufficient detail in the original manuscript, which may have led to ambiguity. Following your valuable suggestion, we quantitatively assessed the extent of overlap between the independent test set and the pretraining corpus and accordingly revised and expanded the relevant sections of the Results and Discussion. These revisions now more clearly describe the training objectives and data sources of the pretrained protein language model, as well as its specific role within the GeoEPred framework.

First, we re-examined the relationship between the independent test set and the ESM-2 pretraining dataset. Our analysis showed that, among the proteins in the current independent test set, only 56 had no exact sequence match in the pretraining corpus examined. When we further assessed sequence relatedness using the predefined similarity criterion, only 22 proteins had a maximum sequence identity below 60% to sequences in the pretraining corpus. These findings led us to recognize that, although the current independent test set was constructed with sequence-level redundancy control and was separated from the downstream supervised training data, it should not be regarded as a strictly "pretraining-independent test set". We have clarified this point in the revised manuscript and accordingly refined our description of the "independent test set." In other words, the independence referred to in the present study primarily denotes independence from the supervised data used for downstream GeoEPred model development, rather than strict independence from all data used to pretrain the underlying protein foundation models.

We also fully recognize that overlap with the pretraining corpus may influence downstream performance evaluation. Although ESM-2 is pretrained using a self-supervised learning objective and does not directly use effector-class labels such as T1SE, T2SE, T3SE, T4SE, or T6SE, the presence of a test protein itself, or of highly similar sequences, in the pretraining corpus may still result in more favorable learned representations for those proteins. Therefore, although pretraining-data overlap is distinct from direct supervised label leakage, it may nonetheless lead to an overly optimistic estimate of downstream generalization performance. We have explicitly acknowledged this potential effect in the revised Discussion and have avoided interpreting the current test results as evidence of generalization under a strictly pretraining-unseen setting. We also further clarified the role of the pretrained models in the original manuscript. In this study, ESM-2 was used as a frozen protein sequence representation model, with its parameters kept fixed throughout GeoEPred training. ESMFold was used to generate predicted protein structures from amino acid sequences and likewise did not participate in downstream parameter optimization. In light of the reviewer's comment and our additional analysis, we no longer regard parameter freezing as a basis for excluding potential effects arising from

pretraining-data exposure. Instead, we now describe freezing solely as an implementation detail of how these pretrained models were used within the framework, and we explicitly identify pretraining-data overlap as a potential limitation of the current evaluation setting in the revised manuscript.

The reviewer's suggestion was highly insightful and prompted us to consider it important to further examine how GeoEPred performs on proteins that were unseen in both the pretraining corpus and the downstream model-development dataset. To this end, we surveyed recent literature for newly experimentally validated bacterial effectors and prioritized proteins derived from genomes that were not represented in the pretraining dataset. We collected effectors that had been validated by wet-laboratory experiments in the original studies from *Aeromonas veronii C4* [1], *Aeromonas jandaei DSM 7311* [2], *Aeromonas schubertii ATCC 43700* [3], and *Xanthomonas hortorum pv. pelargonii 305* [4]. After further excluding proteins that directly overlapped with the examined pretraining corpus and removing samples sharing more than 60% sequence identity with proteins in the GeoEPred training set, we retained 9 experimentally validated T3SEs and 7 experimentally validated T6SEs. On this small exploratory external dataset, GeoEPred correctly identified 8 of the 9 T3SEs and all 7 T6SEs, with most correct predictions receiving relatively high model confidence. One T3SE was misclassified as a non-effector. Given that this dataset contains only 16 experimentally validated positive samples, is currently restricted primarily to T3SEs and T6SEs, and lacks corresponding non-effector negative controls as well as representatives of the other secretion-system classes, we consider these results to provide supplementary evidence of the model's transferability across data sources rather than a comprehensive benchmark of six-class generalization performance. We therefore regard this analysis as exploratory validation and report the results transparently, while avoiding broader generalization claims that would not be adequately supported by the limited sample size and class coverage.

In addition, we further reviewed recent studies addressing both the use of pretrained protein language models in downstream protein prediction tasks and the potential biases associated with their evaluation. Using protein language models as frozen, general-purpose representation extractors has become increasingly common in protein structure prediction, functional annotation, protein–protein interaction prediction, and other protein-related tasks. This strategy is particularly valuable for biological prediction problems with limited experimentally labeled data, because such models can learn transferable evolutionary, structural, and biochemical representations from large-scale unlabeled protein sequence corpora. Similar transfer-learning paradigms have also been adopted in bacterial effector prediction, including DeepSecE [5] and MoCETSE [6], both of which employ pretrained protein

language models to derive sequence representations. At the same time, we recognize that recent studies have increasingly drawn attention to the overlap between pretraining corpora and downstream test sets, as well as the possibility that such overlap may lead to overly optimistic performance estimates [7]. These studies further indicate that, even when pretraining is based on self-supervised objectives, ensuring only that the downstream supervised training set and independent test set do not contain highly similar sequences is not sufficient to fully exclude the potential influence of data exposure during foundation-model pretraining on subsequent performance evaluation.

Based on this consideration, we also explored whether it would be feasible to reconstruct a complete effector-protein prediction dataset after strictly excluding proteins that overlapped with, or showed high sequence similarity to, the pretraining corpus. Our analysis indicated that, given the currently available number and class distribution of experimentally validated effectors, implementing a strict "pretraining-aware separation" would substantially reduce the number of usable samples across secretion-system classes and further exacerbate class imbalance, thereby compromising both the biological representativeness and statistical robustness of the dataset. We therefore explicitly acknowledge this limitation in the revised manuscript and clarify that the performance reported in the present study primarily reflects model behavior under independence from the downstream supervised training data, rather than performance under a strictly pretraining-unseen evaluation setting.

We consider the reviewer's suggestion to highlight an important and forward-looking methodological issue in the application of protein foundation models. As experimentally validated effectors, complete bacterial genomes, and pretraining resources with clearly documented versions and provenance continue to accumulate, developing evaluation frameworks that rigorously implement pretraining-aware separation and systematically comparing performance across conventional independent testing, sequence-redundancy-controlled testing, and strictly pretraining-unseen testing will be an important direction for our future work.

We sincerely thank the reviewer again for this valuable suggestion. This comment prompted us to re-examine and more rigorously define what is meant by an "independent test set" in the present study, and helped us more clearly distinguish independence from downstream supervised training data from independence from protein foundation-model pretraining data. We believe that these additional analyses and corresponding revisions have improved the transparency and methodological rigor of the study with respect to dataset construction, interpretation of model performance, and the limitations of the current evaluation framework.

Reference:

[1] Michova H, Pliva J, Jirsova A, Jurnecka D, Kamanova J. Cytotoxicity induced by Aeromonas schubertii is orchestrated by a unique set of type III secretion system effectors. Vet Res. 2025;56(1):113. doi:10.1186/s13567-025-01548-2.
[2] Fridman CM, Keppel K, Rudenko V, Altuna-Alvarez J, Albesa-Jové D, Bosis E, Salomon D.A new class of type VI secretion system effectors can carry two toxic domains and are recognized through the WHIX motif for export. PLoS Biol. 2025;23(3):e3003053. doi:10.1371/journal.pbio.3003053.
[3] Wagner N, Ben-Meir D, Teper D, Pupko T. Complete genome sequence of an Israeli isolate of Xanthomonas hortorum pv. pelargonii strain 305 and novel type III effectors identified in Xanthomonas. Front Plant Sci. 2023;14:1155341. doi:10.3389/fpls.2023.1155341.
[4] Jiang X, Li H, Ma J, Li H, Ma X, Tang Y, Li J, Chi X, Deng Y, Zeng S, Liu Z. Role of Type VI secretion system in pathogenic remodeling of host gut microbiota during Aeromonas veronii infection. ISME J. 2024;18(1):wrae053. doi:10.1093/ismejo/wrae053.
[5] Zhang Y, Guan J, Li C, Wang Z, Deng Z, Gasser RB, Song J, Ou HY. DeepSecE: a deep-learning-based framework for multiclass prediction of secreted proteins in Gram-negative bacteria. Research (Wash D C). 2023;6:0258. doi:10.34133/research.0258.
[6] Shi H, Lin Y, Liu D, Zou Q. MoCETSE: a mixture-of-convolutional experts and transformer-based model for predicting Gram-negative bacterial secreted effectors. PLoS Comput Biol. 2026;22(3):e1013397. doi:10.1371/journal.pcbi.1013397.
[7] Koh HY, Zheng Y, Yang M, Arora R, Webb GI, Pan S, Li L, Church GM. AI-driven protein design. Nat Rev Bioeng. 2025;3(12):1034–1056. doi:10.1038/s44222-025-00349-8.

The following content constitutes revisions to the main text.

1) Corresponding revisions have been made in the “Protein language model” section (Page 5, Lines 108–118).

Previous studies have demonstrated that embeddings generated by protein language models contain rich information about protein sequence patterns and properties [34,35]. This capability has promoted their widespread use in tasks involving protein sequence prediction and functional annotation. ESM-2 [11] is a Transformer-based protein language model comprising 33 layers and approximately 650 million parameters. It was pretrained using a self-supervised masked-language-modeling objective on approximately 65 million unique protein sequences sampled from approximately 43 million UniRef50 clusters derived from approximately 138 million UniRef90 sequences, enabling it to learn general evolutionary, biochemical, and structural patterns from unlabeled protein data. Building on ESM-2 representations, ESMFold [11] predicts atomic-level protein structures directly from individual amino acid sequences without requiring multiple sequence alignments or structural templates. In this study, the weights of both ESM-2 and ESMFold remained frozen throughout the subsequent training and evaluation processes.

2) We have added a discussion of this limitation and the corresponding directions for future work to the revised Discussion section (Page 21, Lines 727-736).

Third, the current evaluation mainly supports the sequence-level generalization capability of GeoEPred and should not be considered equivalent to strict species-level generalization. Although homologous sequences between the training and independent test sets were strictly controlled in this study, complete species-level separation was not implemented because of the limited amount and uneven species distribution of experimentally validated data. As the number of experimentally validated effectors increases, external datasets without species overlap can be used to evaluate cross-species generalization. In addition, because pretrained protein language models are generally trained on large-scale public databases, some degree of potential association may exist between pretraining data and subsequent independent test data. Future evaluations using updated external data may further assess model generalization under more strictly independent conditions.

3) We added S18 Table to provide a more comprehensive evaluation of GeoEPred on recently experimentally validated effector proteins.

S18 Table. GeoEPred predictions for recently experimentally validated bacterial effector proteins.

Effector	Strain	Secretion system	GeoEPred prediction	Confidence	Reference
AopI	*Aeromonas schubertii ATCC 43700*	T3SE	T3SE	0.99202687	[3]
AopJ	*Aeromonas schubertii ATCC 43700*	T3SE	T3SE	0.9745239	[3]
AopL	*Aeromonas schubertii ATCC 43700*	T3SE	T3SE	0.9941506	[3]
AopT	*Aeromonas schubertii ATCC 43700*	T3SE	T3SE	0.99421096	[3]
AopU	*Aeromonas schubertii ATCC 43700*	T3SE	T3SE	0.993871	[3]
TseI-like	*Aeromonas jandaei DSM 7311*	T6SE	T6SE	0.99375856	[4]
Tle1-like	*Aeromonas jandaei DSM 7311*	T6SE	T6SE	0.9903679	[4]
Awe1	*Aeromonas jandaei DSM 7311*	T6SE	T6SE	0.990186	[4]
DUF3289	*Aeromonas jandaei DSM 7311*	T6SE	T6SE	0.98906076	[4]
XopBB	*X. hortorum pv. pelargonii 305*	T3SE	T3SE	0.99400467	[5]
XopBC	*X. hortorum pv. pelargonii 305*	T3SE	T3SE	0.99064296	[5]
XopBD	*X. hortorum pv. pelargonii 305*	T3SE	T3SE	0.99371815	[5]
XopBE	*X. hortorum pv. pelargonii 305*	T3SE	Non-Effector	0.9620185	[5]
Tse1	*A. veronii C4*	T6SE	T6SE	0.99262416	[6]
Tse2	*A. veronii C4*	T6SE	T6SE	0.9911902	[6]
Tse3	*A. veronii C4*	T6SE	T6SE	0.9931722	[6]

3. The T6SE prediction results show perfect performance (ACC, F1, and MCC = 1.000). Such exceptional performance is unusual for biological datasets and raises concerns regarding dataset redundancy, information leakage, or potential overfitting. Justification is required for this.

Response: We sincerely thank the reviewer for highlighting this important issue. In

response to this comment, we re-examined the dataset partitioning and model-training procedure and retrained the model. We specifically checked for potential dataset overlap and information leakage among the training, validation, and independent test sets. This re-examination confirmed that the three datasets were mutually exclusive and that no information leakage was identified.

During the reanalysis, we observed potential signs of overfitting during the later stages of training in Folds 2 and 5. We therefore introduced an early-stopping strategy using the validation-set F1 score as the monitoring metric, with a patience of five epochs. Training was terminated when the validation F1 score ceased to improve, and the checkpoint corresponding to the best validation performance was retained. As shown in the revised figure, the selected stopping points are indicated by gray vertical dashed lines, including Epoch 19 for Fold 2 and Epoch 9 for Fold 5.

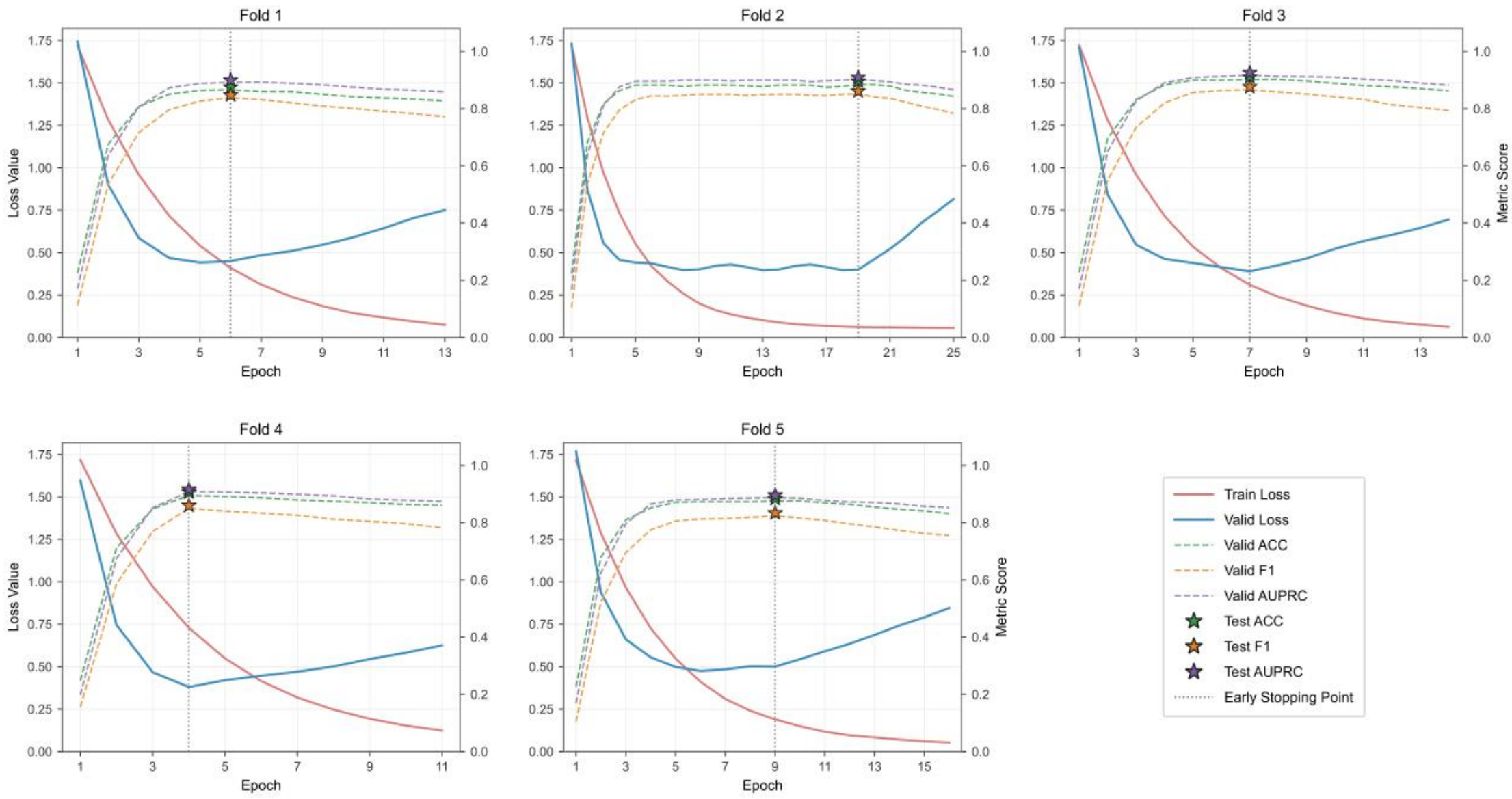

We then reevaluated the models selected using this strategy. At the corresponding checkpoints, the ACC, F1, and AUPRC values on the independent test set were 0.923, 0.884, and 0.915, respectively. The class-wise performance metrics were also recalculated. The performance of the T6SE class decreased after retraining, with ACC, F1, and MCC changing from 1.000 to 0.989, 0.930, and 0.927, respectively. The recalculated results showed relatively strong performance across multiple evaluation metrics.

We have accordingly revised Fig. 6 and updated the relevant sections of the manuscript to provide additional details on the early-stopping strategy, the criterion used for model selection, the revised class-wise results, and the corresponding independent-test results. We sincerely thank the reviewer again for this valuable comment, which prompted us to re-examine the training procedure and present the model-selection process and evaluation results more transparently.

The following content constitutes revisions to the main text.

1) We added a new "Model Training" subsection to the Methods section to provide a detailed description of the revised early-stopping strategy used during model training (Page 8–9, Lines 241–250).

The batch size and maximum number of training epochs were set to 32 and 30, respectively. Early stopping was performed by monitoring the macro-F1 score on the validation set, with a patience of five epochs, and the checkpoint achieving the best validation performance in each fold was retained. Automatic mixed precision and dynamic loss scaling were employed during training, and the global gradient norm was clipped to a maximum value of 1.0.

2) We revised the "Comparison with State-of-the-Art Models" subsection accordingly (Page 14, Lines 441–452).

From the overall results, GeoEPred achieved predictive performance comparable to that of current state-of-the-art methods, with a more evident advantage in the T6SE prediction task (Fig.6A-C, S11 Table). In comparison with the multi-class models, GeoEPred achieved comparable predictive performance across most classes (S11 Table). In terms of overall performance, GeoEPred improved accuracy by 2.69% ($p = 0.098$) and 3.46% ($p = 0.048$) compared with DeepSecE and MoCETSE, respectively, which were among the better-performing baseline models. For the T6SE class (S12 Table), GeoEPred showed favorable predictive performance, with both threshold-independent metrics, AUC and AUPRC, being significantly higher than those of DeepSecE (AUC: 95% CI: 0.0012-0.0233, $p = 0.020$; AUPRC: 95% CI: 0.0118-0.3631, $p = 0.016$). As shown in S13 Table, GeoEPred also showed a modest improvement in AUC and AUPRC compared with MoCETSE (AUC: $\Delta = +0.0098$, 95% CI: 0.0000-0.0279, $p = 0.086$; AUPRC: $\Delta = +0.0098$, 95% CI: 0.0000-0.1457, $p = 0.076$). These results suggest that GeoEPred has good discriminative capability for T6SE prediction and may have an advantage in identifying fine-grained features associated with T6SEs.

3) We have updated Fig. 6 to present the revised model prediction results in comparison with existing methods.

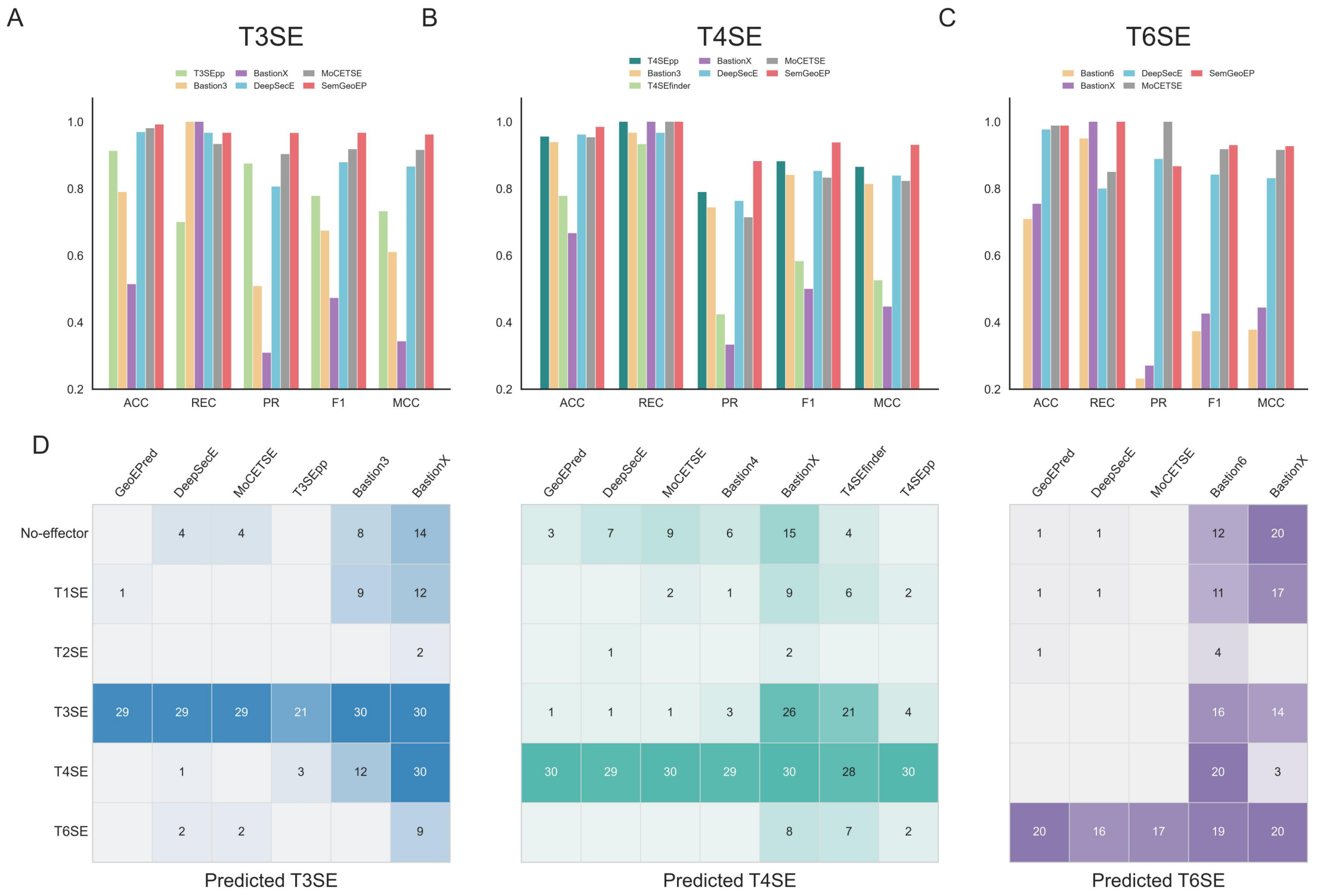

Fig 6. Performance evaluation of GeoEPred in comparison with mainstream models. A-C: Performance comparison of GeoEPred with mainstream prediction models on T3SE (A), T4SE (B), and T6SE (C) prediction tasks. D: Comparison of true positive and false positive predictions from different models on the independent test set (Dataset S2), which contains 150 non-effectors, 20 T1SEs, 10 T2SEs, 30 T3SEs, 30 T4SEs, and 20 T6SEs. The heatmaps illustrate the distribution of predicted samples across various effector protein types.

4) We have accordingly updated S6 Table.

Task	Method	ACC (%)	REC (%)	PR (%)	F1	MCC
T1SE	MoCETSE	99.23	90.0	100.0	0.947	0.945
	DeepSecE	99.23	90.0	100.0	0.947	0.945
	T1SEstacker	85.3	80.0	43.2	0.561	0.515
	**GeoEPred**	**99.23**	**90.0**	**100.0**	**0.947**	**0.945**
T2SE	MoCETSE	95.38	90.0	45.00	0.600	0.618
	**DeepSecE**	**97.3**	90.0	**60.00**	**0.720**	**0.723**
	GeoEPred	95.0	**90.0**	42.9	0.581	0.601
T3SE	Bastion3	79.0	100.0	50.8	0.674	0.610
	T3SEpp	91.3	70.0	87.5	0.778	0.732
	BastionX	51.4	100.0	30.9	0.473	0.343
	DeepSecE	96.9	96.7	80.6	0.879	0.866
	MoCETSE	98.08	93.33	90.32	0.918	0.916
	**GeoEPred**	**99.2**	96.7	**96.67**	**0.967**	**0.962**
T4SE	Bastion3	93.9	96.7	74.4	0.841	0.814
	T4SEfinder	77.8	93.3	42.4	0.583	0.526
	T4SEpp	95.6	100.0	79.0	0.882	0.865
	BastionX	66.7	100.0	33.3	0.500	0.447
	DeepSecE	96.2	96.7	76.3	0.853	0.839
	MoCETSE	95.38	100.0	71.43	0.833	0.823
	**GeoEPred**	**98.46**	**100.0**	**88.24**	**0.938**	**0.931**
T6SE	Bastion6	70.91	95.00	23.17	0.373	0.378
	BastionX	75.45	100.00	27.03	0.426	0.444
	DeepSecE	97.69	80.00	88.89	0.842	0.831
	MoCETSE	98.85	85.00	100.00	0.918	0.916
	**GeoEPred**	**98.85**	**100.0**	**86.69**	**0.930**	**0.927**

4. In Table 1, the "Structure only" model exhibits very poor accuracy (50.76%) despite the manuscript emphasizing the importance of structural information. This suggests that structural representations alone have limited discriminative power, and the authors should discuss why geometric features fail to perform independently.

Response: We sincerely thank the reviewer for this insightful and constructive comment. We fully agree that the limited performance of the "Structure only" model warrants further discussion, particularly with respect to the information captured by

the structural modality and its role within the multimodal framework. Following the reviewer's suggestion, we have expanded the corresponding analysis and discussion in the revised manuscript.

First, we examined the relationship between the quality of ESMFold-predicted structures and model performance. We used the pLDDT score to quantify the quality of the predicted structures. pLDDT is a residue-level confidence metric for protein structure prediction, ranging from 0 to 100, with higher scores indicating greater confidence in the predicted local structure of the corresponding residues. Our results showed that model performance generally improved as the quality of the predicted structures increased, indicating an association between structural prediction quality and the predictive performance of GeoEPred. This observation also suggests that more reliable structural information can provide more informative structural representations for the model. We also observed that GeoEPred retained relatively stable performance for proteins with lower-quality predicted structures. From this result, we infer that the language-model features introduced through the sequence branch provide additional predictive information when structural confidence is relatively low, allowing the model to rely more on sequence-derived representations under these conditions.

At the same time, to further examine whether the limited performance of the "Structure only" model was primarily related to the structural encoding scheme adopted in our framework, we explored an alternative representation based on the 3Di structural alphabet, which has been widely used in protein classification and function prediction. Specifically, we replaced the original structural encoding component with 3Di representations and evaluated the resulting structure-only model. We found that the 3Di-based single-modality model also showed relatively limited performance on the independent test set. From this observation, we considered that discretizing three-dimensional structures into 3Di representations may not fully retain all of the geometric and physicochemical information contained in higher-order protein conformations, which may limit the ability of structural representations alone to accurately distinguish effector proteins. This result also led us to further consider the distinct and complementary roles of sequence-derived semantic information and geometric structural information in effector prediction. To better understand this observation, we further examined related studies. We found that protein structure and function are closely associated, but their relationship is not strictly one-to-one. Similar or closely related structural frameworks can support different molecular functions, while functional specificity may also depend on the identities of key residues, their local sequence environments, and their spatial organization within the three-dimensional structure. From this perspective, proteins from different classes

may share similar global or local geometric patterns, whereas class-discriminative information may additionally depend on residue-level properties and sequence context. Accordingly, when the structural branch is used independently, shared geometric patterns across classes may reduce class separability. When structural information is integrated with sequence features, the geometric representation can provide additional spatial relationships and structural context for residue-level semantic representations. This interpretation is also consistent with the design and observations reported in previous sequence–structure integrated frameworks for protein function prediction.

method	AUC	ACC	Pre	F1	MCC
3Di tokens	0.760	0.412	0.307	0.339	0.189
Ours	0.827	0.542	0.505	0.488	0.365

Finally, we further extended the SHAP analysis to each individual class. We found that sequence-derived features contributed more strongly to the overall predictions, accounting for 74.53% of the total feature attribution, whereas structural features accounted for 25.47%. At the class-specific level, several structural features ranked among the most influential features. A closer examination of the SHAP attribution profiles showed that structural features such as struct_f30, struct_f68, and struct_f61 were consistently ranked highly for several effector classes, including T1SE, T2SE, T3SE, and T6SE. These observations suggest that, although structural representations contribute a smaller proportion of the overall feature attribution, they still provide meaningful class-specific information for the recognition of particular secretion-system effectors and for defining the corresponding decision boundaries. From these results, we understand that the sequence modality serves as the primary source of class-discriminative information, while the structural modality provides complementary spatial and relational context.

Based on these observations, we revised the relevant statements in the manuscript to describe the role of structural information more precisely. We sincerely thank the reviewer again for this important insight. The reviewer's comments were highly instructive and helped us develop a clearer and more objective understanding of the respective contributions of sequence and structural information, as well as present these contributions more accurately in the revised manuscript.

The following content constitutes revisions to the main text.

1) We added a new subsection entitled "Influence of ESMFold Structural Confidence on Model Performance" to the Results section to clearly present the quantitative relationship between predicted structural confidence and the final classification performance of the model (Page 15–16, Lines 472–502).

Because our model uses ESMFold-predicted structures as inputs to the structural branch, we further investigated the association between predicted structural

confidence and effector protein classification performance.

Based on the mean protein-level pLDDT score, the independent test set was divided using fixed thresholds into four confidence groups: *Very low* (pLDDT < 50), *Low* ($50 \leq$ pLDDT < 70), *Confident* ($70 \leq$ pLDDT < 90), and *Very high* (pLDDT $\geq$ 90). As shown in Fig.7A, the corresponding accuracies were 81.82%, 87.50%, 88.89%, and 96.47%, respectively, exhibiting an overall increase with structural confidence.

To quantify this association on a continuous scale, the 260 test proteins were further divided into 10 approximately equal-sized pLDDT bins, each containing 26 proteins. The points in Fig.7B represent the mean pLDDT and observed accuracy within each bin, whereas the fitted curve and its 95% confidence band were estimated from protein-level binary correctness outcomes for all 260 samples using logistic regression. The probability of correct classification increased with mean pLDDT, although the wider confidence band in the low-pLDDT region indicated greater estimation uncertainty. After adjusting for the true class label and aligned protein length, each 10-point increase in mean pLDDT was associated with a 79.8% increase in the odds of correct classification (OR = 1.798, 95% CI: 1.242-2.602, $p = 0.002$). Consistently, correctly classified proteins had a higher mean pLDDT than incorrectly classified proteins (79.88 vs 71.54; Mann-Whitney $U = 3522.0$, $p = 0.029$, BH-FDR-adjusted $q = 0.044$, Cliff's $\delta = 0.271$). Together, these results demonstrate a statistically significant positive association between predicted structural confidence and classification performance.

Despite this association, the model retained good predictive performance for lower-confidence structures: the accuracy was 81.82% in the Very low group and approximately 84% in the lowest-pLDDT quantile bin. Together with the performance degradation observed after removing the sequence branch in the ablation study, these findings support the complementary roles of sequence and structural information. In particular, the sequence branch may provide additional discriminative cues when structural confidence is limited, thereby improving the model's robustness to uncertainty in predicted structures

2) We have added Fig.7 to investigate how structural confidence, quantified by pLDDT scores, influences model performance, providing further insights into the relationship between predicted structural reliability and predictive capability.

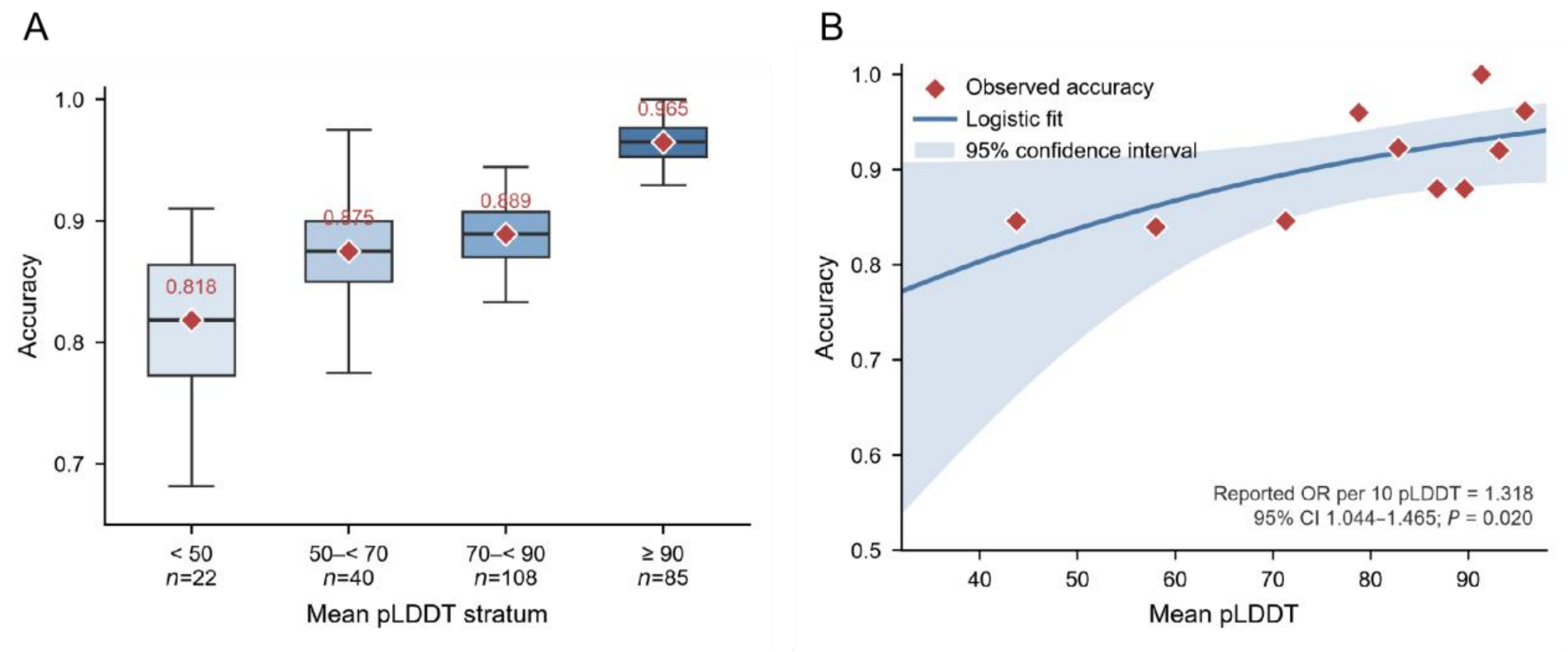

Fig 7. Model performance across ESMFold confidence levels. A: Classification accuracy across four structural-confidence groups defined by the mean protein-level pLDDT: *Very low* (<50), *Low* (50-<70), *Confident* (70-<90), and *Very high* (≥90). Error bars indicate 95% confidence intervals. B: Continuous association between mean pLDDT and classification accuracy. The 260 test proteins were divided into 10 approximately equal-sized bins, each containing 26 proteins. Black points represent the mean pLDDT and observed accuracy within each bin. The red dashed curve and gray shaded 95% confidence band were estimated by logistic regression using the protein-level correct/incorrect outcomes of all 260 test samples rather than the binned observations. After adjustment for the true class label and aligned protein length, each 10-point increase in mean pLDDT was associated with higher odds of correct classification (OR = 1.798, 95% CI: 1.242-2.602, $p = 0.002$).

3) We added a further analysis and discussion of the model performance when using the structural modality alone in the "Multimodal feature ablation" section (Page 10, Lines 295–315).

To validate the above quantitative findings, Fig.3 employs Uniform Manifold Approximation and Projection (UMAP) [38] to visualize the latent feature space of validation samples under different feature configurations. When sequence or structural features are used in isolation, sample embeddings exhibit substantial dispersion, with severe feature overlap between positive and negative classes, directly leading to high misclassification rates. Conversely, introducing ESM embedding alone markedly improves cluster homogeneity, but the centers of all clusters remain relatively close, causing samples near the decision boundary to be easily confused. Although after refining embedded representation to condense information, a clearer separation emerges between effector and non-effector proteins, inter-effector classification remains suboptimal. In contrast, the joint fusion of sequence semantics and structural features yields the most discriminative topology: positive and negative samples form dense clusters in the high-dimensional embedding space with well-defined, mutually separated boundaries. This visual evidence supports that

cross-modal integration provides complementary information and contributes to improved representation separability. Consistent with the UMAP visualization, the performance differences observed in ACC, MCC, and AUC can be interpreted from different perspectives. AUC evaluates the overall ranking capability across all classification thresholds, which is consistent with the observation that both models achieve a certain degree of class separation in the embedding space. In contrast, ACC and MCC reflect classification performance under a specific decision threshold and are therefore more sensitive to the clarity of class boundaries. Statistical significance analysis further showed that GeoEPred achieved improvements of 4.23% in ACC (p = 0.0080) and 6.04% in MCC (p = 0.0020) compared with the refined sequence semantic model, whereas only a minor difference was observed in AUC. These results suggest that incorporating structural features improves the discrimination of samples located near decision boundaries, leading to the observed improvements in ACC and MCC (S5 Table).

4) We also incorporated a corresponding analysis into the Discussion section (Page 19-20, Lines 645–660).

Modality ablation analysis further reveals the different roles of the two modalities in GeoEPred (Table 2). The refined sequence-semantic representation already provides relatively strong overall discriminative capability, whereas the UMAP results show that several effector protein classes still exhibit local overlap in the low-dimensional representation space (Fig.3). After structural information is incorporated, class separation and overall classification performance are further improved, suggesting that the structural modality complements sequence-semantic representations by providing residue-level spatial relationships, local geometric environments, and conformational context. Notably, the model shows relatively limited performance when structural information is used alone, indicating that the structural modality mainly plays a complementary role in this task. SHAP analysis shows that sequence-derived features account for 74.53% of the overall feature attribution, whereas structural features account for 25.47%, indicating that sequence information constitutes the primary basis for discrimination (Fig.5). Nevertheless, several structural features show relatively high importance across multiple classes, suggesting that structural information can provide important spatial and conformational information for specific classes. Therefore, the main value of the structural modality lies in complementing three-dimensional relationships that are difficult to describe directly using sequence representations and in forming a more complete protein representation together with sequence semantics.

5) We updated Fig.5 to more clearly present the SHAP analysis and the relative contributions of the sequence and structural modalities.

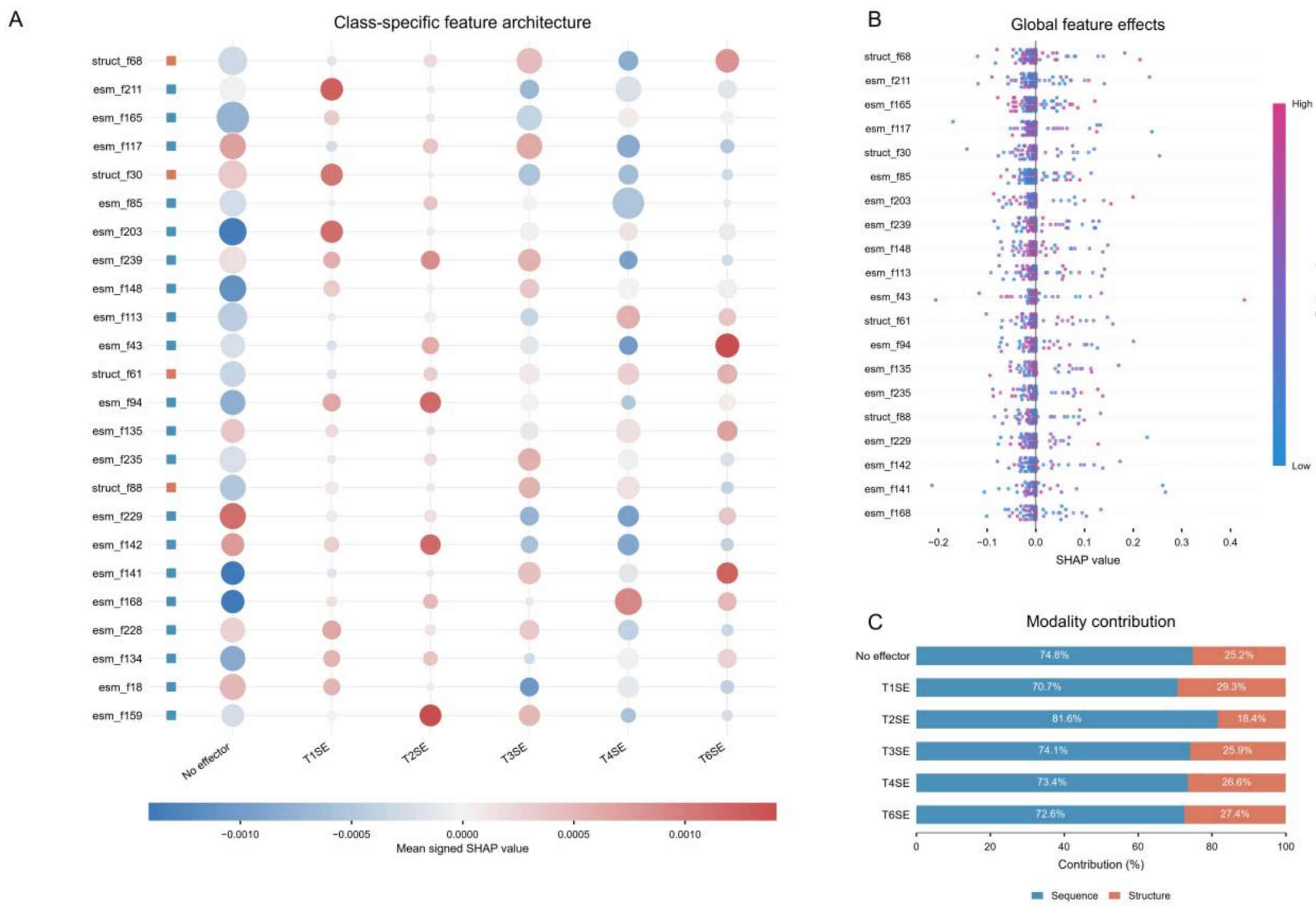

Fig 5. Interpretability analysis of GeoEPred. A: Class-specific feature attribution patterns across effector classes. B: Global feature effects summarized by SHAP values. C: Relative contributions of sequence and structural modalities across different classes.

6) We added S4 Fig to present the SHAP beeswarm plots for all six classes.

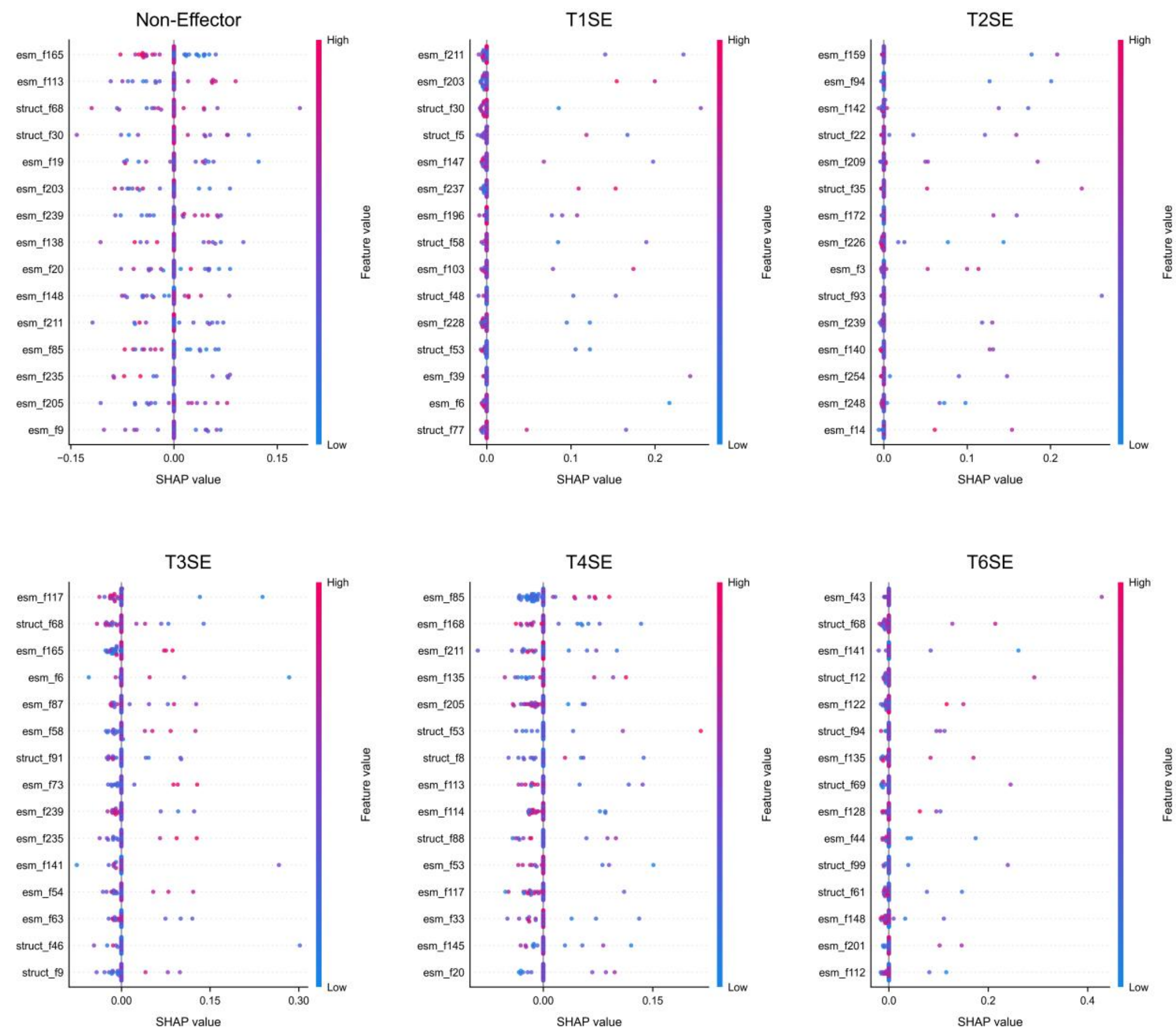

S4 Fig. Class-stratified SHAP summary beeswarm plots for six target categories.

5. The multimodal model achieves only a marginal improvement in AUC compared with the sequence semantic model (98.44% vs. 98.48%), despite a substantial increase in ACC and MCC. This discrepancy should be explained.

Response: We sincerely thank the reviewer for this rigorous comment. In response to the reviewer's comment, we have added further analysis and discussion of this difference in the revised manuscript.

To evaluate the discriminative capability of the protein embedding features learned by the model, we performed dimensionality reduction and visualization analyses on the embeddings generated by the refined sequence semantic model and GeoEPred, respectively (as shown below). UMAP was used to project the high-dimensional features generated by the refined sequence semantic model and the secretion embeddings generated by GeoEPred into a two-dimensional space, providing a visual representation of the distribution relationships among samples. The results showed that the refined sequence semantic model was already able to separate different classes to some extent in the two-dimensional space; however, the

boundaries between different categories remained relatively ambiguous. By incorporating structural features, GeoEPred further improved the separation between different classes and produced clearer class boundaries.

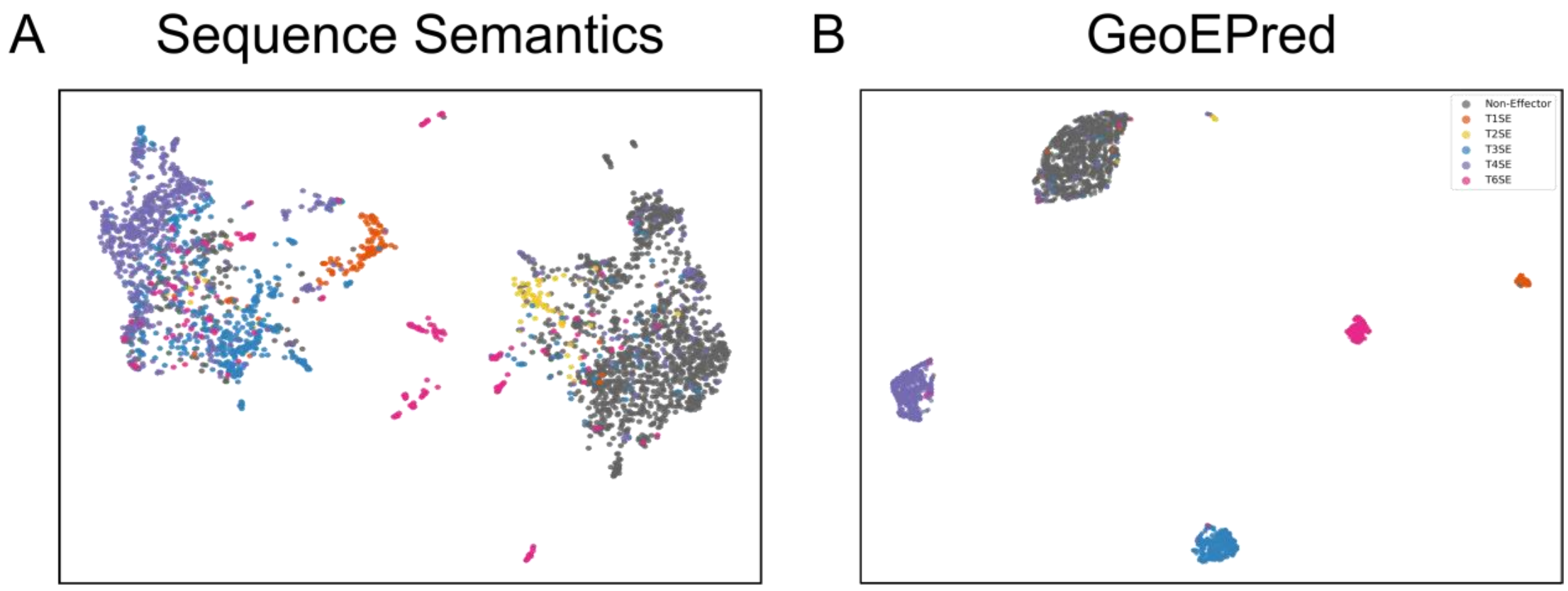

Considering the performance metrics together, AUC measures the overall ranking ability of the model across all classification thresholds, which is consistent with the observation that both models were able to achieve an initial clustering tendency among different classes in the embedding space. In contrast, ACC and MCC reflect the classification accuracy and class-balanced performance under a specific decision threshold, corresponding to the clearer class boundaries exhibited by GeoEPred in the embedding space. Therefore, we further performed statistical significance tests to evaluate the performance differences between the two models (as shown in the table below). The results showed that the difference between the two models in terms of AUC was minimal, whereas GeoEPred achieved statistically significant improvements over the refined sequence semantic model in ACC and MCC, with relative improvements of 4.23% in ACC (p = 0.0080) and 6.04% in MCC (p = 0.0020), respectively. These quantitative results are consistent with the UMAP visualization, suggesting that the incorporation of structural modalities provides complementary features beyond sequence-based representations alone, which helps improve the classification of samples near decision boundaries and consequently contributes to the observed improvements in ACC and MCC.

Evaluation Metric	p-value	Statistical Significance
ACC	0.0080	**
F1	0.0140	*
MCC	0.0020	**
AUC	0.0540	ns

We again sincerely thank the reviewer for raising this question, which helped us gain a deeper understanding of the representation mechanisms underlying the multimodal representations and made the discussion of model performance evaluation in the manuscript more rigorous and comprehensive.

The following content constitutes revisions to the main text.

1) We have added Table S5 to supplement the statistical analysis of the differences among different modalities.

S5 Table. Performance gain, 95% confidence intervals, and statistical significance analysis of the proposed model compared to baseline models.

Baseline Model	Metric	Δ	95% CI	*p*-values
Geometric Priors	ACC	+0.3962	[0.3308, 0.4577]	0.0020
	F1	+0.4258	[0.3512, 0.5025]	0.0020
	MCC	+0.5742	[0.4860, 0.6646]	0.0020
	AUC	+0.1659	[0.1349, 0.1969]	0.0020
	AUPRC	+0.4606	[0.3743, 0.5462]	0.0020
Sequence only	ACC	+0.2000	[0.1499, 0.2538]	0.0020
	F1	+0.2203	[0.1555, 0.2893]	0.0020
	MCC	+0.2658	[0.1969, 0.3371]	0.0020
	AUC	+0.0599	[0.0420, 0.0809]	0.0020
	AUPRC	+0.2315	[0.1589, 0.3152]	0.0020
Sequence Semantics	ACC	+0.0423	[0.0115, 0.0731]	0.0080
	F1	+0.0379	[0.0069, 0.0702]	0.0140
	MCC	+0.0604	[0.0194, 0.1043]	0.0020
	AUC	+0.0082	[0.0000, 0.0180]	0.0540
	AUPRC	+0.0081	[-0.0257, 0.0563]	0.5035

2) We have added further discussion in the "Multimodal feature ablation" section to provide a more comprehensive analysis of the contributions of different modalities (Page 10, Lines 295–315).

To validate the above quantitative findings, Fig.3 employs Uniform Manifold Approximation and Projection (UMAP) [38] to visualize the latent feature space of validation samples under different feature configurations. When sequence or structural features are used in isolation, sample embeddings exhibit substantial dispersion, with severe feature overlap between positive and negative classes, directly leading to high misclassification rates. Conversely, introducing ESM embedding alone markedly improves cluster homogeneity, but the centers of all clusters remain relatively close, causing samples near the decision boundary to be easily confused. Although after refining embedded representation to condense information, a clearer separation emerges between effector and non-effector proteins, inter-effector classification remains suboptimal. In stark contrast, the joint fusion of sequence semantics and structural features yields the most discriminative topology: positive and negative samples form dense clusters in the high-dimensional embedding space with well-defined, mutually separated boundaries. This visual evidence supports that cross-modal integration provides complementary information and contributes to

improved representation separability. Consistent with the UMAP visualization, the performance differences observed in ACC, MCC, and AUC can be interpreted from different perspectives. AUC evaluates the overall ranking capability across all classification thresholds, which is consistent with the observation that both models achieve a certain degree of class separation in the embedding space. In contrast, ACC and MCC reflect classification performance under a specific decision threshold and are therefore more sensitive to the clarity of class boundaries. Statistical significance analysis further showed that GeoEPred achieved relative improvements of 4.23% in ACC (p = 0.0080) and 6.04% in MCC (p = 0.0020) compared with the refined sequence semantic model, whereas only a minor difference was observed in AUC. These results suggest that incorporating structural features improves the discrimination of samples located near decision boundaries, leading to the observed improvements in ACC and MCC (S15 Table).

6. The authors claim that feature tokenization substantially outperforms cross-attention and concatenation strategies. However, the differences among fusion strategies are relatively modest, and no statistical tests are presented to establish significance. Explain why?

Response: We sincerely thank the reviewer for this valuable comment regarding the observed performance differences and the importance of statistical validation. We agree that the significance of the performance improvements among different fusion strategies should be evaluated more carefully. Following the reviewer's suggestion, we performed additional statistical analyses to assess the performance differences between the fusion strategies and revised the corresponding statements in the manuscript accordingly, to ensure that the conclusions accurately reflect the statistical evidence.

Specifically, for each of the three fusion strategies, we repeated the complete five-fold cross-validation procedure using five independent random seeds. To ensure a fair comparison, all fusion strategies used the same five-fold data partitioning under each random seed. For each strategy, we first calculated the mean performance across the five folds and then summarized the results obtained across repeated evaluations using the mean, standard deviation, and 95% confidence interval (CI) as the primary statistics. For the evaluation of the three models on the independent test set, we further performed a paired stratified bootstrap analysis with 1,000 resampling iterations. Resampling was stratified according to the ground-truth class labels to preserve the original distribution of all six classes in each bootstrap sample. For each bootstrap iteration, the same resampled set of samples was used for all compared models, and the corresponding difference in each evaluation metric was calculated

directly between the paired model predictions. The resulting bootstrap distribution was then used to estimate the 95% percentile confidence interval and the corresponding p-value.

The statistical analyses provided a more balanced interpretation of the performance differences among the fusion strategies. For AUC, no statistically significant differences were observed between our method and the other two fusion strategies, with p-values of 0.4795 and 0.2877, respectively. Therefore, we have revised the original statements regarding "significant improvement" to more accurately reflect the statistical evidence. Following the reviewer's eighth comment, we also included AUPRC in the model comparison. AUPRC provides complementary information to AUC when evaluating models under class imbalance, as it reflects the trade-off between precision and recall. Compared with feature concatenation and cross-attention fusion, our fusion strategy achieved relative AUPRC improvements of 5.21% and 4.15%, respectively. The paired bootstrap analyses of the corresponding AUPRC differences yielded 95% CIs of 0.0195–0.1043 (p = 0.0020) and 0.0070–0.0908 (p = 0.0120), respectively. These results show that the statistical evidence differed across evaluation metrics: the differences in AUC were not statistically significant, whereas the improvements in AUPRC were supported by the paired bootstrap analysis.

We further analyzed the performance differences from the perspective of different evaluation metrics. AUC and AUPRC are both threshold-independent metrics but characterize different aspects of model performance. AUC reflects the overall ranking ability of the model across different classification thresholds, whereas AUPRC summarizes the balance between precision and recall and is particularly informative under class-imbalanced conditions. In comparison, ACC and F1 are typically calculated at a predefined decision threshold and therefore reflect model performance at a specific operating point. In the comparison with the other two fusion strategies, the proposed method showed modest improvements in ACC and F1. For AUPRC, the observed improvement was supported by statistical testing. These results suggest that the proposed fusion strategy provides a more favorable precision–recall trade-off under the class distribution examined in this study. We have revised the corresponding descriptions in the manuscript to present these findings in a more objective and cautious manner.

We sincerely thank the reviewer again for the careful and constructive comments. These suggestions prompted us to refine the experimental evaluation and statistical analysis, enabling a more comprehensive and rigorous interpretation of the model performance differences among fusion strategies.

The following content constitutes revisions to the main text.

1) Corresponding revisions have been made in the "Model component evaluation" section (Page 12, Lines 364–379).

Building on the preceding results indicating that feature tokenization contributes to model performance, we further compared different feature integration strategies. Specifically, feature tokenization, the core fusion mechanism of GeoEPred, was compared with cross-attention fusion and feature concatenation.

As shown in Table 4, the feature tokenization method exhibited improved performance across the evaluated metrics. In five-fold cross-validation, it achieved an accuracy of 0.899 (95% CI: 0.878-0.920), outperforming direct concatenation (0.884; 95% CI: 0.849-0.919) and cross-attention fusion (0.886; 95% CI: 0.860-0.912) by 0.015 and 0.013, respectively. On the independent test set, our fusion strategy improved the AUPRC by 0.0521 and 0.0415 compared with feature concatenation and cross-attention fusion, respectively. In S8 Table, stratified bootstrap hypothesis testing confirmed that these AUPRC improvements were statistically significant (p = 0.002 vs. concatenation; p = 0.012 vs. cross-attention fusion). Although improvements in some threshold-dependent metrics were not statistically significant, this does not contradict the AUPRC findings, as these metrics capture different aspects of model performance. AUPRC evaluates the precision--recall trade-off across varying decision thresholds and is particularly informative under imbalanced class distributions. Collectively, these results suggest that token-level multimodal fusion provides an effective integration mechanism within the GeoEPred framework compared with attention-based interaction and simple feature concatenation.

2) Updated Table 4.

Table 4. Performance evaluation metrics for different fusion strategies.

Method	ACC		F1		AUPRC	
	CV	Ind	CV	Ind	CV	Ind
Concatenation	0.884	0.889	0.860	0.848	0.854	0.863
Cross-Attention Fusion	0.886	0.908	0.863	0.862	0.901	0.873
Feature Tokenization	**0.899**	**0.923**	**0.880**	**0.884**	**0.907**	**0.915**

3) Newly added S8 Table.

S8 Table. Performance gain, 95% confidence intervals, and statistical significance analysis of the proposed model compared to baseline models.

Method	Metric	Δ	95% CI	*p*-values
Concatenation	ACC	+0.0346	[0.0038, 0.0692]	0.0360
	F1	+0.0368	[-0.0006, 0.0792]	0.0559
	AUC	+0.0039	[-0.0087, 0.0146]	0.4795
	AUPRC	+0.0521	[0.0195, 0.1043]	0.0020
Cross-Attention	ACC	+0.0154	[-0.0154, 0.0462]	0.3916

Fusion	F1	+0.0224	[-0.0152, 0.0670]	0.2717
	AUC	+0.0054	[-0.0046, 0.0159]	0.2877
	AUPRC	+0.0415	[0.0070, 0.0908]	0.0120

7. The discussion states that GeoEPred accurately identifies validated effectors and discovers novel candidates, yet no functional enrichment, domain analysis, or literature evidence is provided for the newly predicted proteins.

Response: We sincerely thank the reviewer for this insightful and valuable comment. We fully agree that providing multi-perspective in silico evidence—including functional enrichment, domain characterization, and structural homology—is essential to support the biological credibility of our newly predicted effector candidates. To address this concern, we have expanded our analysis of the newly predicted candidate effectors in the revised manuscript.

Specifically, using two widely accepted bioinformatics approaches, we additionally performed Gene Ontology (GO) functional enrichment analysis using the entire set of annotated proteins in each genome as the background set, and InterPro-based protein family and domain annotation. For GO and Pfam enrichment analyses, statistical significance was assessed using the hypergeometric test, followed by Benjamini–Hochberg (BH) correction for multiple testing, with an adjusted p-value (q-value) < 0.05 considered significant. These additional analyses and their corresponding results have been fully incorporated into the revised manuscript.

The proteome of Pseudomonas aeruginosa PAO1 (NCBI accession: NC_002516.2) comprises 5,572 protein sequences, of which 377 were identified by our model as candidate proteins. Among the 5,572 proteins included in the background set, 4,345 had Gene Ontology (GO) annotations, while 201 of the 377 predicted candidates were GO-annotated. GO enrichment analysis of the newly predicted candidates identified several significantly overrepresented functional categories. Among the biological process terms, protein secretion was significantly enriched (fold enrichment = 7.2, p = 10^{-28}). Significant enrichment was also observed for the type VI protein secretion system complex within the cellular component category (fold enrichment = 15.3, p = 10^{-7}). At the molecular function level, enriched terms included hydrolase, lipase, and phospholipase activities, which have been associated with effector-mediated modulation of host cellular processes. These enrichment patterns provide functional context for the predicted candidates and identify biological processes that may be relevant to their subsequent experimental characterization. Pfam domain enrichment analysis provided complementary information at the protein-domain level. The T6SE candidate set showed significant enrichment of phage tail- and baseplate-associated domains (fold enrichment = 4.6, p

$= 6.6 \times 10^{-7}$), consistent with the established evolutionary relationship between the T6SS apparatus and bacteriophage tail structures. These domains included Sel1 repeats, ADP-ribosyltransferase exotoxin domains, and the YopE GAP domain, all of which have been reported in association with known effector proteins. These observations provide additional biological context for the predicted candidate sets while remaining subject to further experimental validation.

The proteome of Salmonella enterica serovar Typhimurium LT2 (NCBI accession: NC_003197.2) comprises 4,432 protein sequences. After excluding 38 experimentally validated T3SEs, GeoEPred identified 307 additional candidate effectors across the five effector classes examined. GO annotations were available for 3,600 background proteins and 244 candidates. GO enrichment analysis revealed significant enrichment of functional categories related to adhesion, biofilm formation, protein secretion, and extracellular enzymatic activity. The most enriched terms included cell--substrate adhesion and biofilm formation (fold enrichment = 8.5, $p = 1.9 \times 10^{-10}$), pilus-associated cellular components (fold enrichment = 8.5, $p = 1.2 \times 10^{-11}$), and glycosyl hydrolase activity (fold enrichment = 8.1, $p = 6.1 \times 10^{-8}$). These findings indicate that the predicted candidate set is enriched for annotations associated with secretion-related and extracellular biological processes, including biofilm development, extracellular enzymatic activity, and interbacterial interactions.

The Legionella pneumophila Philadelphia-1 strain (NCBI accession: NC_002942.5) contains 2,972 protein-coding genes. After excluding 307 experimentally validated type IV secretion effectors (T4SEs), GeoEPred further identified 224 candidate effectors that have not yet been experimentally validated. Because GO annotation coverage was low in both the whole-genome background (560/2,972, 18.8%) and the candidate set (3/224, 1.4%), the available annotations were insufficient for a reliable GO enrichment analysis. We therefore further characterized the candidate proteins based on Pfam domain annotations. Comprehensive annotation using 13 functional databases in InterProScan showed that 170 of the 224 candidate proteins (75.9%) received at least one functional or domain annotation. In the Pfam database used for the subsequent enrichment analysis, 76 candidate proteins (33.9%) were matched to known Pfam domains. Two domains of unknown function (DUF4917 and DUF8197) showed significant enrichment among the predicted candidate proteins (Fold Enrichment = 12.00, $p_{adjust} = 0.012$). These results indicate that the domain composition of the GeoEPred-predicted candidates is not randomly distributed, but is preferentially associated with specific protein families whose functions remain incompletely characterized, providing clues for further investigation of the potential relationships between these domains and effector

functions. In addition, Sel1-like repeats, which are associated with Legionella--host interactions, also showed an enrichment trend (Fold Enrichment = 4.32, p = 0.011). Overall, these findings indicate that the candidate set predicted by GeoEPred exhibits distinct biological structural features and further identifies several protein families and domains that may warrant subsequent experimental investigation.

We sincerely thank the reviewer for this insightful and highly professional comment. This suggestion provided valuable guidance for further refining our study design and evaluation framework, and it also prompted us to conduct additional validation under conditions more closely aligned with practical application scenarios, thereby providing empirical support for the potential research relevance of the candidate proteins.

The following content constitutes revisions to the main text.

1) Newly added Fig 9.

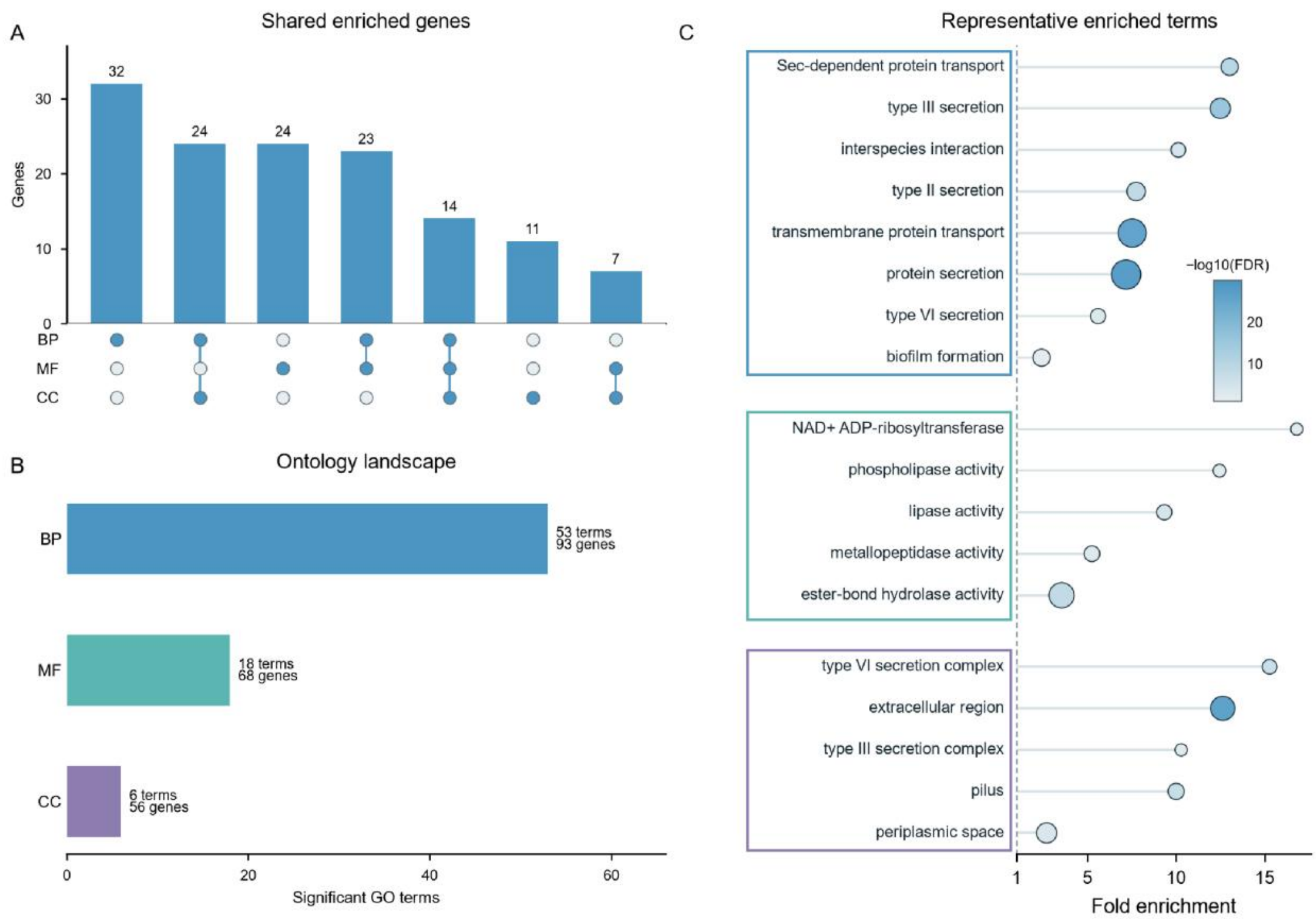

Fig 9. Gene Ontology (GO) functional enrichment analysis of predicted candidate effectors in *Pseudomonas aeruginosa PAO1*. A: The bar plot displays the number of genes shared among different combinations of Biological Process (BP), Molecular Function (MF), and Cellular Component (CC), as indicated by the connected dot matrix below. B: Ontology landscape. Overview of the significant GO terms and associated gene counts categorized by BP (blue), MF (teal), and CC (purple). C: Representative enriched terms. Lollipop charts illustrating representative enriched terms for BP, MF, and CC, respectively. The x-axis represents the fold enrichment, and the bubble size and color intensity correspond to the statistical significance scaled $-\log_{10}(FDR)$.

2) Newly added S7 Fig.

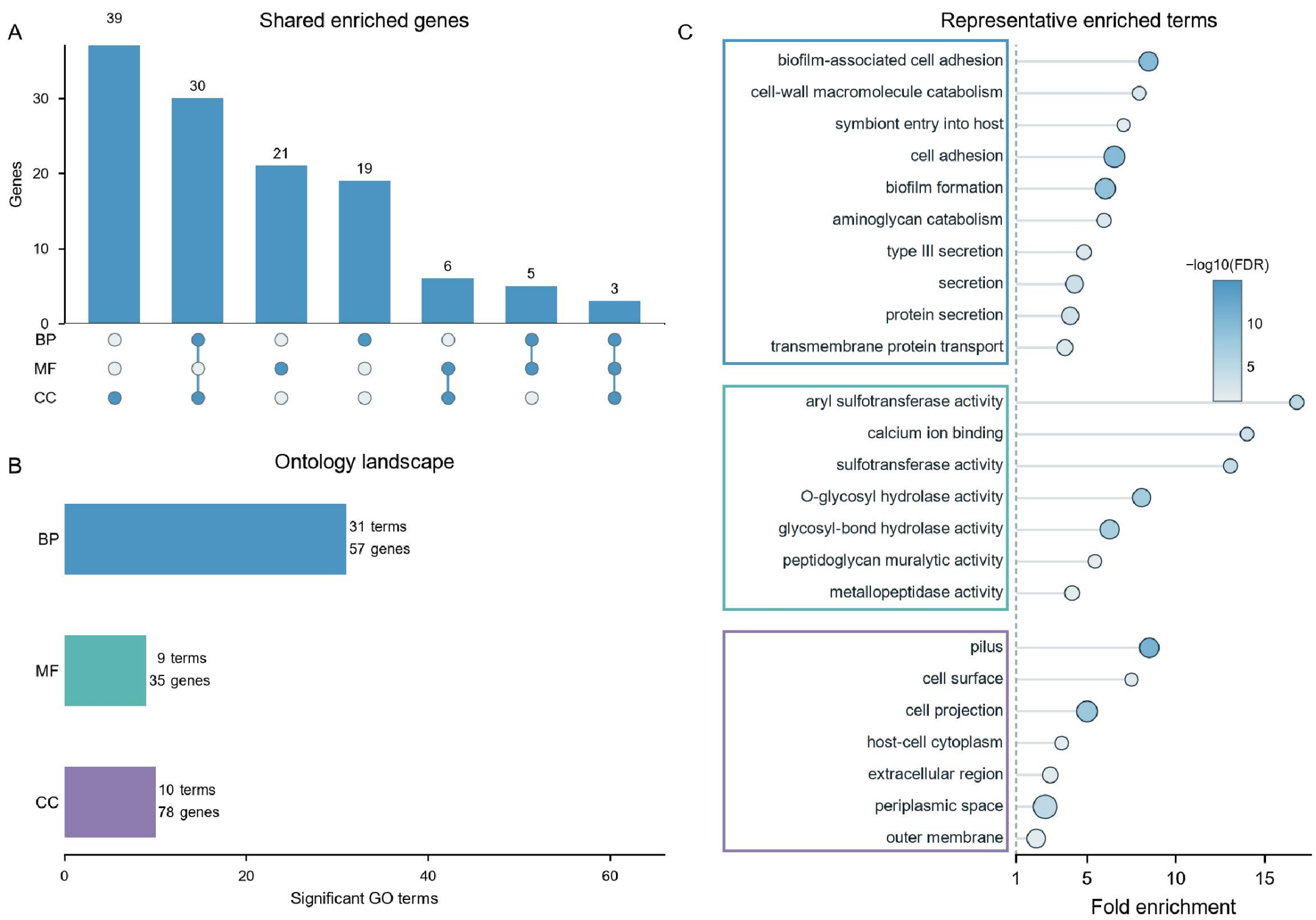

S7 Fig. Gene Ontology (GO) functional enrichment analysis of predicted candidate effectors in *Salmonella Typhimurium LT2*. A The bar plot displays the number of genes shared among different combinations of Biological Process (BP), Molecular Function (MF), and Cellular Component (CC), as indicated by the connected dot matrix below. B: Ontology landscape. Overview of the significant GO terms and associated gene counts categorized by BP, MF (teal), and CC (purple). C: Representative enriched terms. Lollipop charts illustrating representative enriched terms for BP, MF, and CC, respectively. The x-axis represents the fold enrichment, and the bubble size and color intensity correspond to the statistical significance scaled $-\log_{10}(FDR)$.

3) Newly added S17 Table.

S17 Table. Pfam domain enrichment analysis across candidate effector proteins in three bacterial genomes ($p_{adjust} < 0.05$).

Strain	Class	Pfam ID	Domain description	p_{adjust}
NC_002516.2	T6SE	PF00126	LysR family HTH domain	4.95e-06
	T6SE	PF03466	LysR substrate-binding domain	6.60e-07
	T6SE	PF05954	Phage baseplate hub (GPD)	3.43e-06
	T6SE	PF04717	T6SS/phage baseplate tail tube OB	1.76e-05
	T6SE	PF22178	Phage T4 gp5 trimerisation domain	8.82e-05
NC_002942.5	T6SE	PF16263	Domain of unknown function(DUF4917)	1.47e-02
	T3SE	PF26620	Domain of unknown function(DUF8197)	1.96e-02
NC_003197.2	T4SE	PF08238	Sel1 repeat	2.39e-02
	T6SE	PF05638	T6SS effector protein Hcp	4.21e-02
	T6SE	PF04717	T6SS/phage baseplate OB	4.21e-02
	T6SE	PF02368	Bacterial Ig-like domain	4.21e-02
	T6SE	PF05954	Phage baseplate hub	4.21e-02
	T6SE	PF16461	Lambda phage tail tube protein TTP	4.21e-02

4) Corresponding revisions have been made in the "case studies" section (Page 17, Lines 526–559).

To further assess the biological relevance of the newly predicted candidates, we performed functional characterization using Gene Ontology (GO) enrichment analysis and protein domain annotation. Enrichment significance was assessed using a hypergeometric test followed by Benjamini–Hochberg correction for multiple testing. In P. aeruginosa PAO1, GO enrichment analysis revealed significant overrepresentation of secretion-associated functions, including protein secretion and Type VI secretion system complexes. Pfam domain analysis further identified enrichment of several effector-associated domains, including Sel1-like repeats, ADP-ribosyltransferase domains, and YopE-like GAP domains. Sel1-like repeats have been implicated in Legionella host-cell interactions and virulence-associated processes [48]. ADP-ribosyltransferase domains are characteristic of several bacterial toxins and secreted effectors involved in host cellular modulation, such as the T3SS effector ExoS from Pseudomonas aeruginosa [49]. Similarly, the YopE GAP domain represents a well-characterized effector module that manipulates host Rho GTPase signaling [50]. These observations provide additional functional context supporting the potential biological relevance of the predicted candidates.

In addition, we further evaluated the model on two other Gram-negative bacterial genomes: Salmonella enterica subsp. enterica serovar Typhimurium str. LT2 (NC_003197.2) and Legionella pneumophila subsp. pneumophila str. Philadelphia 1 (NC_002942.5). The predicted results were compared against experimentally validated data. GeoEPred reliably identified a large proportion of experimentally confirmed T3SE and T4SE proteins, achieving recall rates of 94.78% (36 out of 38) and 91.2% (280 out of 307), respectively. These results demonstrate that GeoEPred performs robustly across different biological contexts and maintains reliable performance for large-scale genome-wide annotation tasks.

Functional characterization of predicted candidates from these genomes further supported their biological relevance. In Salmonella Typhimurium LT2, GO enrichment analysis revealed significant associations with secretion-related and extracellular biological processes, including adhesion, biofilm formation, and extracellular enzymatic activities (Fig.9). In Legionella pneumophila Philadelphia-1, where GO annotation coverage was insufficient for reliable enrichment analysis, InterPro and Pfam domain analyses identified several enriched protein domains, including DUF4917, DUF8197, and Sel1-like repeats. Sel1-like repeat-containing proteins have previously been implicated in Legionella virulence and host-cell interaction processes, suggesting that these domain architectures may represent potential molecular features associated with host adaptation [48]. In addition, among the predicted T6SE candidates, enrichment of phage tail- and baseplate-associated domains was observed, which is consistent with the reported evolutionary relationship between the Type VI secretion system and contractile bacteriophage tail structures [51]. These findings suggest that the GeoEPred-predicted candidates exhibit distinct functional and structural characteristics associated with bacterial secretion and host interaction. Detailed functional enrichment and domain annotation results are provided in S7 Fig and S16 Table.

8. The authors must provide separate, class-stratified SHAP swarm plots for each of the six target classes (Non-effector, T1SE, T2SE, T3SE, T4SE, T6SE) to show whether the feature utilization profiles shift for the minority classes. Additionally, they must implement a balanced metric comparison if possible.

Response: We sincerely thank the reviewer for this important and constructive suggestion. We agree that class-stratified SHAP analyses across all six classes are valuable for examining whether feature utilization patterns differ among effector classes with different sample sizes, particularly for minority classes. We also agree that performance evaluation under class imbalance should include metrics that provide complementary information beyond accuracy-based measurements. Following the reviewer's suggestion, we recalculated SHAP attribution values for each of the six model outputs: non-effector, T1SE, T2SE, T3SE, T4SE, and T6SE. In addition, we incorporated AUPRC into all comparative experiments as a class-imbalance-sensitive metric. By focusing on the precision–recall trade-off, AUPRC provides a complementary assessment of the model's ability to identify minority effector classes in imbalanced settings, where accuracy-based metrics may be dominated by the performance on majority-class samples. The corresponding revisions have been incorporated into the revised manuscript.

Specifically, we generated separate SHAP beeswarm plots for all six output

classes and ranked the top 15 features according to their mean absolute SHAP values (S4 Fig). Sequence-derived representations were predominant across all classes, while the composition of highly influential features differed among individual classes. Among the top 15 features for non-effectors, T1SE, T2SE, T3SE, T4SE, and T6SE, structural features accounted for 2, 6, 3, 4, 3, and 6 features, respectively. This distribution suggests a relatively higher representation of structural features among the most influential features for T1SE and T6SE prediction, whereas T2SE showed a higher proportion of sequence-derived features among its top-ranked features. These results indicate that the model integrates sequence-derived and structural representations while exhibiting class-dependent feature attribution patterns.

The class-stratified SHAP analysis further showed that the integration of ESM-derived sequence features and spatial structural features resulted in distinct attribution profiles across different effector classes. Minority classes, including T1SE, T2SE, and T6SE, exhibited right-tailed positive SHAP distributions for several important features in their corresponding class outputs. For example, the core T6SE-associated feature "esm_f43" showed a positive SHAP contribution of approximately 0.45 on the model output scale. T3SE and T4SE also exhibited increased contributions from several structural features, suggesting that structural representations contribute differently across secretion-system-associated effector classes.

We sincerely thank the reviewer again for this valuable suggestion. The additional class-specific SHAP analyses and class-imbalance-sensitive performance evaluation help clarify the feature utilization patterns of different effector classes and provide a more comprehensive assessment of predictive performance under imbalanced classification conditions.

The following content constitutes revisions to the main text.

1) We added S4 Fig to present the SHAP beeswarm plots for all six classes.

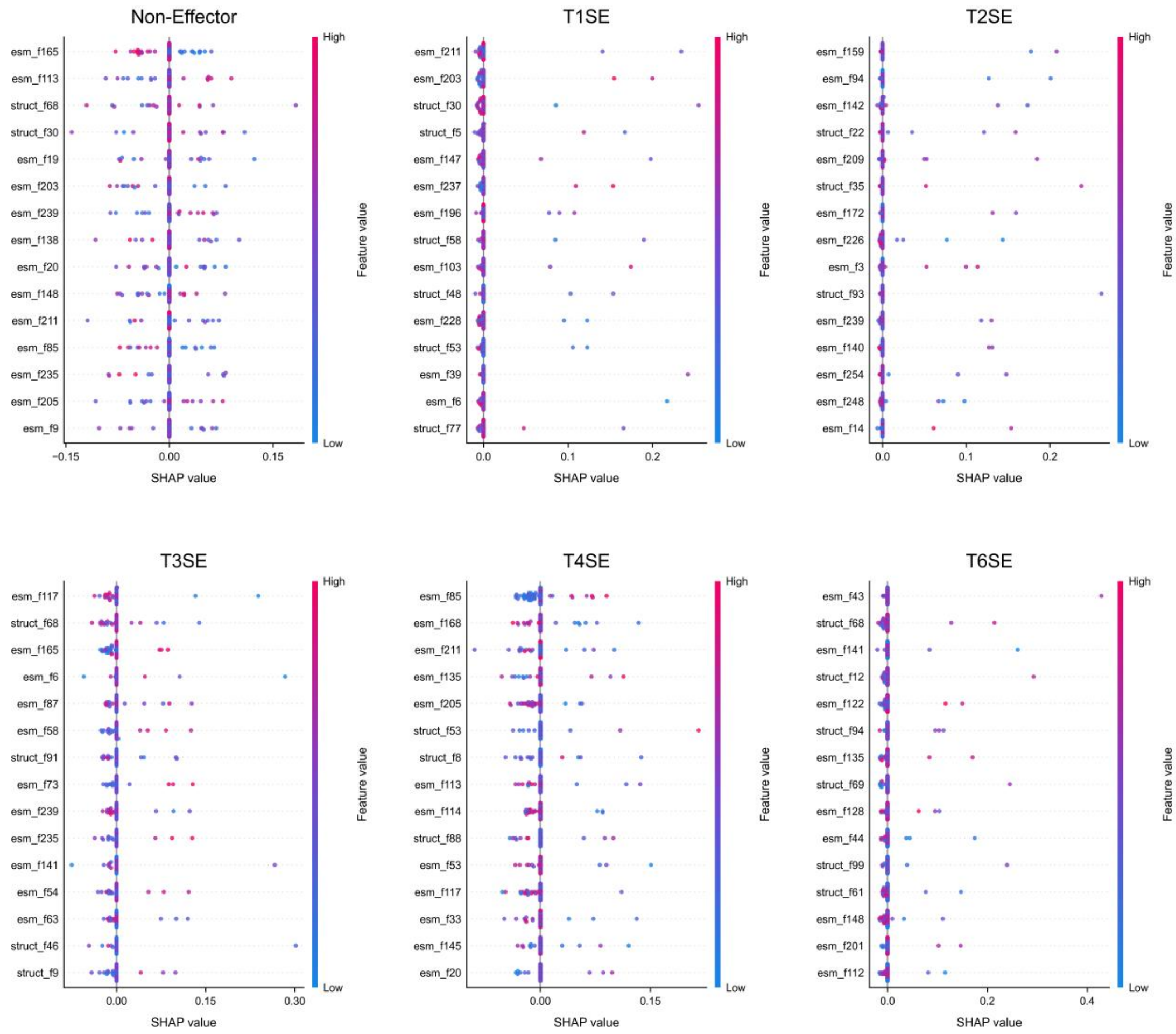

S4 Fig. Class-stratified SHAP summary beeswarm plot for six target categories. This figure displays the top 15 most influential features for each of the six categories—Non-Effector, T1SE, T2SE, T3SE, T4SE, and T6SE—ranked from top to bottom based on their total contribution (mean absolute SHAP value) to the prediction for that category. The horizontal axis represents the SHAP value, indicating the marginal impact of the feature value on the model output.

2) We added new content to the subsection "Interpretability Analysis of Multimodal Feature Contributions" to further examine class-specific differences in feature utilization and to clarify how sequence- and structure-derived information contributes differently across output classes. (Page 13, Lines 402–410).

To further examine class-specific feature utilization, we generated separate SHAP beeswarm plots for the six output classes (S4 Fig). Although ESM-derived features dominated the top-ranked features across all classes, their importance profiles varied substantially among classes. Structural features were particularly prominent for T1SE and T6SE, accounting for 6 of the top 15 features in each class, compared with 2-4 in the other classes. These results indicate that GeoEPred employs distinct multimodal feature-utilization patterns for different effector classes, with structural information

providing especially important complementary signals for T1SE and T6SE prediction.

3) We updated Table 2 to include AUPRC comparisons across different feature modalities.

Table 2. Performance evaluation of multimodal feature ablation.

Feature Type	ACC		F1		AUPRC	
	CV	Ind	CV	Ind	CV	Ind
Geometric Priors	0.615	0.527	0.541	0.459	0.561	0.454
Sequence only	0.681	0.723	0.639	0.664	0.681	0.683
Sequence Semantics	0.877	0.881	0.854	0.846	0.893	0.907
GeoEPred	**0.899**	**0.923**	**0.880**	**0.884**	**0.907**	**0.915**

4) We updated Table 3 to include a comparison of AUPRC performance.

Table 3. Performance comparison of model variants in the ablation study using ACC, F1, and AUPRC on cross-validation and independent test sets.

Model Variant	ACC		F1		AUPRC	
	CV	Ind	CV	Ind	CV	Ind
w/o Contrastive Learning	0.881	0.889	0.851	0.854	0.901	0.887
w/o Adaptive Attention Pooling	0.886	0.885	0.863	0.851	0.899	0.890
w/o Feature Tokenization	0.861	0.873	0.829	0.838	0.864	0.875
Full Model (Ours)	**0.899**	**0.923**	**0.880**	**0.884**	**0.907**	**0.915**

5) We updated Table 4 to include a comparison of AUPRC performance.

Table 4. Performance evaluation metrics for different fusion strategies.

Method	ACC		F1		AUPRC	
	CV	Ind	CV	Ind	CV	Ind
Concatenation	0.884	0.889	0.860	0.848	0.854	0.863
Cross-Attention Fusion	0.886	0.908	0.863	0.862	0.901	0.873
Feature Tokenization	**0.899**	**0.923**	**0.880**	**0.884**	**0.907**	**0.915**

6) We updated Table 5 to include comparisons of class-imbalance-sensitive performance metrics across the different conventional methods.

Table 5. Comparison of the proposed method with baseline methods.

Method	ACC		F1		AUPRC	
	CV	Ind	CV	Ind	CV	Ind
Traditional Machine Learning						
SVM	0.892	0.908	0.854	0.878	0.882	0.885
Random Forest	0.882	0.896	0.786	0.806	0.825	0.831
Gradient Boosting	0.865	0.869	0.770	0.749	0.805	0.794
KNN	0.857	0.831	0.809	0.804	0.828	0.821
Naive Bayes	0.804	0.815	0.744	0.757	0.768	0.778
Decision Tree	0.691	0.692	0.603	0.611	0.642	0.642
LDA	0.578	0.619	0.611	0.641	0.602	0.632
Deep Learning Method						

BiLSTM_Attention	0.862	0.900	0.852	0.864	0.879	0.885
MLP	0.875	0.900	0.856	0.850	0.880	0.871
GRU	0.884	0.900	0.848	0.860	0.883	0.886
Vanilla_Transformer	0.866	0.881	0.854	0.852	0.871	0.891
Our method						
GeoEPred	**0.899**	**0.923**	**0.880**	**0.884**	**0.907**	**0.915**

9. If possible a quantitative empirical p-value for the co-localization claims should be provided.

Response: We sincerely thank the reviewer for this valuable and constructive suggestion. Following the reviewer's recommendation, we performed a genome-constrained permutation analysis to quantitatively evaluate whether predicted effector proteins exhibit significant enrichment in genomic regions adjacent to their corresponding secretion-system gene clusters.

Specifically, we analyzed the 5,572 protein sequences encoded by the Pseudomonas aeruginosa PAO1 genome. Secretion systems were annotated from the ordered proteome using MacSyFinder v2.1.6 with the TXSScan v1.1.4 diderm models. The protein identifiers obtained from GeoEPred predictions were subsequently mapped to their corresponding genomic positions based on the same genome annotation.

To establish an empirical null distribution, we performed 10,000 random permutations. In the primary analysis, genomic co-localization was defined as a predicted effector gene located within a neighborhood of no more than 10 intervening CDSs from a compatible secretion-system gene cluster (k=10). For each permutation, the same number of proteins as the observed predicted effectors of a given secretion-system class was randomly sampled without replacement from the eligible PAO1 proteome while maintaining the genomic organization of secretion-system loci. The number of randomly selected proteins satisfying the same co-localization criterion was then recalculated to generate the null distribution. The one-sided empirical p-value was calculated as:

$$p_{emp}=\frac{1+\sum_{b=1}^{B} I(X_b \geq X_{obs})}{B+1}$$

where X_{obs} denotes the observed number of co-localized predicted effectors, X_b denotes the corresponding number obtained in the b-th permutation, and I(.) is the indicator function. In addition, to assess the robustness of the results to the choice of neighborhood window, we performed sensitivity analyses using k=0, 5, 20, and 50 CDS windows. The resulting *p*-values were adjusted for multiple hypothesis testing using the Benjamini–Hochberg procedure to control the false discovery rate (FDR),

yielding the corresponding q-values.

The results revealed a system-specific pattern of genomic co-localization. The predicted T3SEs (N = 343), T6SEs (N = 163), and T1SEs (N = 12) were all enriched in the vicinity of their cognate secretion-system loci. For example, within the <10-CDS window, T3SEs and T6SEs exhibited 10.24-fold ($p < 0.0001$, $q < 0.0001$) and 4.54-fold ($p < 0.0001$, $q < 0.0002$) enrichment, respectively. T1SEs showed an even more pronounced, localized enrichment within the ≤5-CDS neighborhood, reaching 42.99-fold above the random expectation ($p < 0.0001$, $q < 0.0002$). Notably, fold enrichment decreased monotonically as the flanking genomic window was widened, supporting the highly localized nature of the signal rather than an effect driven by genome-wide background noise. In contrast, T2SEs (N = 113) showed no significant co-localization at any distance threshold; for example, only 1.57-fold enrichment was observed within the <10-CDS window ($p = 0.2970$, $q = 0.3305$). We therefore revised the original conclusion to clarify that significant genomic-neighborhood enrichment was observed specifically for T1SEs, T3SEs, and T6SEs, rather than representing a universal property of all predicted effector classes.

We sincerely thank the reviewer again for these insightful comments. The reviewer's suggestions guided us to refine the statistical analysis and provide a more detailed assessment of the genomic distribution of the predicted effectors, thereby improving the rigor and completeness of the study. We have added the relevant discussion and corresponding p-value analyses to the revised manuscript.

The following content constitutes revisions to the main text.

1) We have added a discussion of the co-localization *p*-values in the "Case Study" section (Page 16, Lines 512–519).

Meanwhile, high-confidence candidate proteins identified by GeoEPred, specifically T1SE, T3SE, and T6SE (S6 Fig), show strong genomic proximity to their cognate secretion system loci detected by MacSyFinder (Fig.8), suggesting that these candidate effectors may be functionally and genomically associated with their corresponding secretion machinery. Permutation testing confirmed significant spatial co-localization near cognate gene clusters for T1SE, T3SE, and T6SE, as shown in S15 Table ($p < 0.0001$). This observation is consistent with previous reports showing that some experimentally validated effector proteins are frequently located near secretion system gene clusters or pathogenicity islands [47].

2) We added S15 Table to present the genome-wide co-localization p-values for each effector class in Pseudomonas aeruginosa PAO1.

S15 Table. Genomic co-localization analysis of predicted effectors and corresponding secretion system gene clusters in *Pseudomonas aeruginosa PAO1*. Co-localization patterns were assessed across different CDS neighborhood windows,

including observed co-localized effectors, expected values from random permutations, enrichment levels, and statistical significance.

type	N_{pred}	k_{cds}	X_{obs}	X_{exp}	Enrichment Fold	p-value	FDR q-value
T1SE	12	≤ 5	5	0.1163	42.9914x	< 0.0001	< 0.0002
		≤ 10	5	0.2024	24.6986x	< 0.0001	< 0.0002
		≤ 20	5	0.3747	13.3429x	< 0.0001	< 0.0002
T2SE	113	≤ 5	3	1.2983	2.3108x	0.1372	0.1747
		≤ 10	3	1.9063	1.5737x	0.2970	0.3305
		≤ 20	5	3.1231	1.6009x	0.2037	0.2377
T3SE	343	≤ 5	23	2.2150	10.3837x	< 0.0001	< 0.0001
		≤ 10	29	2.8306	10.2452x	< 0.0001	< 0.0001
		≤ 20	34	4.0628	8.3686x	< 0.0001	< 0.0001
T6SE	163	≤ 5	14	2.4280	5.7660x	< 0.0001	< 0.0001
		≤ 10	15	3.3056	4.5377x	< 0.0001	< 0.0001
		≤ 20	17	5.0590	3.3603x	0.0002	0.0003

Reviewer #4:

Manuscript is interesting I have some major queries.

1. Authors needs to evaluate geopred on completely independent data sets.

Response: We sincerely thank the reviewer for this important and constructive comment. We fully agree that evaluation on a completely independent test set is essential for assessing the generalizability of GeoEPred. We acknowledge that the description of the model training and testing procedures in the original manuscript was insufficiently detailed. To address this concern, we have added a new subsection entitled "Model Training" in the Methods section of the revised manuscript, providing a detailed description of the model development and validation workflow. In addition, we have supplemented the comparative experiments by reporting the mean and standard deviation from five-fold cross-validation and the performance on the independent test set.

Specifically, GeoEPred was developed using stratified five-fold cross-validation. Within each fold, the training data were further divided into training and validation subsets, and the model checkpoint achieving the highest macro-F1 score on the validation set was selected for evaluation. The independent test set consisted entirely of unseen samples and remained isolated throughout model training, feature selection, hyperparameter optimization, decision-threshold determination, and model selection. The test set was used exclusively for the final performance evaluation. This strict separation ensured an unbiased assessment of model performance on unseen data.

We sincerely thank the reviewer again for this valuable suggestion. The additional description of the training strategy and independent test evaluation has improved the transparency and methodological rigor of the revised manuscript.

The following content constitutes revisions to the main text.

1) To address this concern, we have added a new subsection entitled "Model training" to Section 2 of the revised manuscript (Page 8, Lines 228–233).

GeoEPred was trained using stratified five-fold cross-validation, with all experiments repeated using five different random seeds (42, 52, 62, 72, and 82). For each random seed, the available training data were partitioned into training and validation subsets within each fold, and the model checkpoint achieving the highest macro-F1 score on the validation set was selected and retained. The independent test set remained completely isolated throughout model training, hyperparameter determination, and model selection, and was used solely for the final performance evaluation [37].

2. Needs to clarify the homologous proteins between training and testing datasets were removed?

Response: We sincerely thank the reviewer for highlighting this important issue. We fully agree that a clear description of the sequence redundancy removal procedure, together with explicit evidence supporting the sequence-level independence of the training and independent test sets, is important for a rigorous assessment of model generalization. In response to the reviewer's suggestion, we systematically re-examined the data-processing workflow and the potential presence of homologous sequences between the training and independent test sets. A detailed description of the sequence clustering, redundancy removal, and cross-dataset homology screening procedures has now been added to the Methods section of the revised manuscript.

Following the dataset construction strategy proposed by Zhang et al. [1], we used CD-HIT (v4.8.1) to reduce sequence redundancy within the candidate training set and the independent test set separately. A global sequence identity threshold of 60% was applied, with the aligned region required to cover at least 80% of both the shorter and longer sequences. For each dataset, protein sequences from all classes were pooled for clustering irrespective of class labels, and only one representative sequence was retained from each cluster.

Cross-dataset homology was then examined using CD-HIT-2D under the same criteria, namely, sequence identity ≥60% and alignment coverage ≥80% for both sequences. The redundancy-reduced independent test set was used as the reference set, and training sequences meeting these similarity criteria with any test sequence were removed. Following this filtering procedure, the final dataset comprised 3,155 training sequences and 260 independent test sequences. In addition, to improve the transparency and reproducibility of the study, we have made the redundancy-reduced FASTA files, CD-HIT clustering results, and relevant intermediate files generated during data processing publicly available at:

https://github.com/shouzhensong/GeoEPred.

We sincerely thank the reviewer again for this constructive suggestion. These revisions provide a clearer description of the dataset construction and redundancy-removal procedures and improve the transparency and methodological rigor of the study.

Reference:

[1] Zhang Y, Guan J, Li C, Wang Z, Deng Z, Gasser RB, Song J, Ou HY. DeepSecE: A deep-learning-based framework for multiclass prediction of secreted proteins in Gram-negative bacteria. Research (Wash D C). 2023;6:0258. doi:10.34133/research.0258.

The following content provides supplementary additions to the main text.

1) Corresponding revisions have been made in the "Dataset construction" section (Page 4, Lines 59–88).

The datasets used for model training and evaluation were compiled from datasets released with established effector prediction methods and from widely used biological databases.

The dataset construction and redundancy-removal procedures were designed with reference to DeepSecE [13], a representative study in the field. Specifically, T1SE and T2SE sequences were obtained from the TSE-ARF dataset [21]. The T3SE and T6SE datasets were compiled by integrating sequences from Bastion3 [7], DeepT3 [22], SecReT6 [23], Bastion6 [8], DeepT3_4 [24], and TSE-ARF [21]. The T4SE dataset was assembled from DeepSecE [13], OPT4e [25], T4SEfinder [26], iT4SE-EP [27], Bastion4 [28], T4SE-XGB [29], TSE-ARF [21], T4SEpp [30], and DeepT3_4 [24]. Following previous representative studies [31, 32], the negative class was constructed using curated non-secreted proteins and homologous sequences from selected pathogenic bacteria reported in earlier studies. These candidate negative samples had been further screened against multiple bacterial genomes using BLASTp in the original studies, based on predefined thresholds for the *E*-value and sequence identity. To provide an objective assessment of model generalization, we additionally assembled an independent test set. The T3SE, T4SE, T6SE, and non-secreted protein samples were collected from the test datasets released with Bastion3, CNN-T4SE [33], and Bastion6, whereas the T1SE and T2SE samples were randomly selected from the original BastionHub dataset.

To minimize potential overestimation of model performance caused by sequence redundancy, CD-HIT v4.8.1 [33] was applied separately to the candidate training set and the independent test set. A global sequence identity threshold of 60% was used, and the aligned region was required to cover at least 80% of both the shorter and longer sequences. Within each dataset, sequences from all effector and non-effector classes were pooled before clustering, and only one representative sequence was retained from each cluster. Cross-dataset filtering was subsequently performed using CD-HIT-2D under the same identity and coverage criteria. The non-redundant independent test set was used as the reference dataset, and candidate training sequences showing at least 60% global sequence identity to any test sequence were removed. This procedure minimized the risk of information leakage between the training and test sets.

The data sources for each effector type and the corresponding sample numbers after CD-HIT filtering are summarized in S1 Table. The resulting training set comprised 1,575 non-effector proteins, 128 T1SEs, 65 T2SEs, 505 T3SEs, 623 T4SEs,

and 259 T6SEs. The independent test set contained 150 non-effector proteins, 20 T1SEs, 10 T2SEs, 30 T3SEs, 30 T4SEs, and 20 T6SEs (S1 Fig A). Protein lengths ranged from fewer than 100 to more than 1,000 amino acids, with most sequences falling between 100 and 700 amino acids (S1 Fig B).

3. Proteins from the same bacterial Species appears in both sets?

Response: We sincerely thank the reviewer for raising this important point. Following the reviewer's suggestion, we conducted additional database-based analyses to assess both sequence identity and sequence similarity between the training set and the independent test set. As the reviewer correctly noted, the training and independent test sets do contain proteins originating from the same bacterial species. We acknowledge that this aspect of the dataset construction was not described in sufficient detail in the original manuscript, and we sincerely apologize for any lack of clarity this may have caused.

In effector protein prediction, experimentally validated effectors remain limited in availability and unevenly distributed across bacterial species, making it challenging to achieve strict species-level separation while preserving the biological diversity required for robust model development. To minimize sequence-level information leakage while retaining representative sequence diversity, we adopted a sequence-level redundancy-control strategy consistent with those used in recent representative studies in this field, including DeepSecE [1] and MoCETSE [2]. Specifically, we applied CD-HIT and CD-HIT-2D with a sequence identity threshold of 60% and a minimum sequence coverage requirement of 80% to remove identical and homologous protein sequences shared between the training and independent test sets. Following this procedure, proteins in the independent test set were neither identical to nor homologous with those used during model development. This sequence-level redundancy-control procedure reduces potential bias arising from highly similar sequences and minimizes the possibility of information leakage between the model-development and independent test datasets.

We found the reviewer's suggestion highly insightful and fully agree that further evaluating the model on genuinely unseen genomes is of substantial importance for assessing its generalizability. Accordingly, we searched for experimentally validated effectors reported in recently sequenced bacterial genomes, focusing on genomes that were not represented in our original dataset, and performed an additional evaluation. Specifically, we collected experimentally validated effectors from *Aeromonas schubertii ATCC 43700* [3], *Aeromonas jandaei DSM 7311* [4], *Xanthomonas hortorum pv. pelargonii 305* [5], and *Aeromonas veronii C4* [6]. Among these, the genomes of *A. veronii C4* and *X. hortorum pv. pelargonii 305* were newly sequenced

and released after 2025. Although experimentally validated effectors from other secretion-system classes have also been reported in the past two years, the corresponding genomes had been sequenced earlier and were already represented in our dataset. We therefore did not include those samples in this additional evaluation. After excluding sequences sharing more than 60% sequence identity with proteins in the training set, we obtained 9 experimentally validated T3SEs and 7 experimentally validated T6SEs. On this small, genome-level unseen test set, GeoEPred showed encouraging generalization performance, correctly identifying 8 of the 9 T3SEs and all 7 T6SEs, generally with high prediction confidence, as shown in the table below. One T3SE, however, was misclassified as a non-effector.

We sincerely thank the reviewer once again for this valuable comment. This suggestion prompted us to further examine and more clearly define the independence criteria used in our dataset construction, thereby improving the transparency of the dataset preparation process and strengthening our discussion of the scope and limitations of the current evaluation framework.

Reference:

[1] Zhang Y, Guan J, Li C, Wang Z, Deng Z, Gasser RB, Song J, Ou HY. DeepSecE: A deep-learning-based framework for multiclass prediction of secreted proteins in Gram-negative bacteria. Research (Wash D C). 2023;6:0258. doi:10.34133/research.0258.

[2] Shi H, Lin Y, Liu D, Zou Q. MoCETSE: A mixture-of-convolutional experts and transformer-based model for predicting Gram-negative bacterial secreted effectors. PLoS Comput Biol. 2026;22(3):e1013397. doi:10.1371/journal.pcbi.1013397.

[3] Michova H, Pliva J, Jirsova A, Jurnecka D, Kamanova J. Cytotoxicity induced by Aeromonas schubertii is orchestrated by a unique set of type III secretion system effectors. Vet Res. 2025;56(1):113. doi:10.1186/s13567-025-01548-2.

[4] Fridman CM, Keppel K, Rudenko V, Altuna-Alvarez J, Albesa-Jové D, Bosis E, Salomon D. A new class of type VI secretion system effectors can carry two toxic domains and are recognized through the WHIX motif for export. PLoS Biol. 2025;23(3):e3003053. doi:10.1371/journal.pbio.3003053.

[5] Wagner N, Ben-Meir D, Teper D, Pupko T. Complete genome sequence of an Israeli isolate of Xanthomonas hortorum pv. pelargonii strain 305 and novel type III effectors identified in Xanthomonas. Front Plant Sci. 2023;14:1155341. doi:10.3389/fpls.2023.1155341.

[6] Jiang X, Li H, Ma J, Li H, Ma X, Tang Y, Li J, Chi X, Deng Y, Zeng S, Liu Z. Role of Type VI secretion system in pathogenic remodeling of host gut microbiota during Aeromonas veronii infection. ISME J. 2024;18(1):wrae053. doi:10.1093/ismejo/wrae053.

The following content constitutes revisions to the main text.

1) We have added a corresponding discussion in the Discussion section of the revised manuscript to clarify this limitation (Page 21, Lines 725–737).

Third, the current evaluation primarily supports the sequence-level generalization of GeoEPred rather than strict species-level generalization. Although homologous sequences between the training and independent test sets were strictly controlled, complete species-level separation was not feasible because experimentally validated effectors remain limited and unevenly distributed across species. To preliminarily address this issue, we collected experimentally validated effectors reported in recent years from genomes absent from our current dataset [63-66]. GeoEPred successfully identified 8 T3SEs and all T6SEs in this external evaluation (S18 Table), providing preliminary support for its applicability to previously unseen genomic backgrounds. As more experimentally validated effectors become available, additional external datasets without species overlap with the training data will be incorporated to further assess cross-species generalization. Moreover, because pretrained protein language models are typically trained on large-scale public databases, potential overlap or indirect association with independent test data cannot be completely excluded. Future evaluations using newly available external datasets will therefore provide a more stringent assessment of model generalization.

2) We added S18 Table to provide a more comprehensive evaluation of GeoEPred on recently experimentally validated effector proteins.

S18 Table. GeoEPred predictions for recently experimentally validated bacterial effector proteins.

Effector	Strain	Secretion system	GeoEPred prediction	Confidence	Reference
AopI	*Aeromonas schubertii ATCC 43700*	T3SE	T3SE	0.99202687	[3]
AopJ	*Aeromonas schubertii ATCC 43700*	T3SE	T3SE	0.9745239	[3]
AopL	*Aeromonas schubertii ATCC 43700*	T3SE	T3SE	0.9941506	[3]
AopT	*Aeromonas schubertii ATCC 43700*	T3SE	T3SE	0.99421096	[3]
AopU	*Aeromonas schubertii ATCC 43700*	T3SE	T3SE	0.993871	[3]
TseI-like	*Aeromonas jandaei DSM 7311*	T6SE	T6SE	0.99375856	[4]
Tle1-like	*Aeromonas jandaei DSM 7311*	T6SE	T6SE	0.9903679	[4]
Awe1	*Aeromonas jandaei DSM 7311*	T6SE	T6SE	0.990186	[4]
DUF3289	*Aeromonas jandaei DSM 7311*	T6SE	T6SE	0.98906076	[4]
XopBB	*X. hortorum pv. pelargonii 305*	T3SE	T3SE	0.99400467	[5]
XopBC	*X. hortorum pv. pelargonii 305*	T3SE	T3SE	0.99064296	[5]
XopBD	*X. hortorum pv. pelargonii 305*	T3SE	T3SE	0.99371815	[5]
XopBE	*X. hortorum pv. pelargonii 305*	T3SE	Non-Effector	0.9620185	[5]
Tse1	*A. veronii C4*	T6SE	T6SE	0.99262416	[6]
Tse2	*A. veronii C4*	T6SE	T6SE	0.9911902	[6]
Tse3	*A. veronii C4*	T6SE	T6SE	0.9931722	[6]

4. This manuscript is focused on computational performance no biological validation is seen, I suggest that validate the data biologically.

Response: We sincerely thank the reviewer for this valuable comment. We agree that experimental validation is essential for confirming computational predictions and further establishing the biological functions of candidate effector proteins. Following

the reviewer's suggestion, we have incorporated additional multi-dimensional bioinformatics analyses, including functional enrichment analysis, protein domain characterization, and genomic context analysis, to evaluate whether the predicted candidates exhibit biological features consistent with experimentally characterized effectors reported in previous studies and to further strengthen the biological interpretation of the predictions. These complementary analyses provide evidence supporting the biological relevance of the predicted candidates from functional, structural, and genomic perspectives, and facilitate the prioritization of high-confidence candidates for subsequent experimental investigation.

Specifically, using two widely accepted bioinformatics approaches, we additionally performed Gene Ontology (GO) functional enrichment analysis using the entire set of annotated proteins in each genome as the background set, and InterPro-based protein family and domain annotation. For GO and Pfam enrichment analyses, statistical significance was assessed using the hypergeometric test, followed by Benjamini–Hochberg (BH) correction for multiple testing, with an adjusted p-value (q-value) < 0.05 considered significant. These additional analyses and their corresponding results have been fully incorporated into the revised manuscript.

The proteome of *Pseudomonas aeruginosa PAO1* (NCBI accession: NC_002516.2) comprises 5,572 protein sequences, of which 377 were identified by our model as candidate proteins. Among the 5,572 proteins included in the background set, 4,345 had Gene Ontology (GO) annotations, while 201 of the 377 predicted candidates were GO-annotated. GO enrichment analysis of the newly predicted candidates identified several significantly overrepresented functional categories. Among the biological process terms, protein secretion was significantly enriched (fold enrichment = 7.2, $p = 10^{-28}$). Significant enrichment was also observed for the type VI protein secretion system complex within the cellular component category (fold enrichment = 15.3, $p = 10^{-7}$). At the molecular function level, enriched terms included hydrolase, lipase, and phospholipase activities, which have been associated with effector-mediated modulation of host cellular processes. These enrichment patterns provide functional context for the predicted candidates and identify biological processes that may be relevant to their subsequent experimental characterization. Pfam domain enrichment analysis provided complementary information at the protein-domain level. The T6SE candidate set showed significant enrichment of phage tail- and baseplate-associated domains (fold enrichment = 4.6, $p = 6.6\times10^{-7}$), consistent with the established evolutionary relationship between the T6SS apparatus and bacteriophage tail structures. These domains included Sel1 repeats, ADP-ribosyltransferase exotoxin domains, and the YopE GAP domain, all of which have been reported in association with known effector proteins. These

observations provide additional biological context for the predicted candidate sets while remaining subject to further experimental validation.

The proteome of *Salmonella enterica serovar Typhimurium LT2* (NCBI accession: NC_003197.2) comprises 4,432 protein sequences. After excluding 38 experimentally validated T3SEs, GeoEPred identified 307 additional candidate effectors across the five effector classes examined. GO annotations were available for 3,600 background proteins and 244 candidates. GO enrichment analysis revealed significant enrichment of functional categories related to adhesion, biofilm formation, protein secretion, and extracellular enzymatic activity. The most enriched terms included cell--substrate adhesion and biofilm formation (fold enrichment = 8.5, $p = 1.9\times10^{-10}$), pilus-associated cellular components (fold enrichment = 8.5, $p = 1.2\times10^{-11}$), and glycosyl hydrolase activity (fold enrichment = 8.1, $p = 6.1\times10^{-8}$). These findings indicate that the predicted candidate set is enriched for annotations associated with secretion-related and extracellular biological processes, including biofilm development, extracellular enzymatic activity, and interbacterial interactions.

The *Legionella pneumophila Philadelphia-1* strain (NCBI accession: NC_002942.5) contains 2,972 protein-coding genes. After excluding 307 experimentally validated type IV secretion effectors (T4SEs), GeoEPred further identified 224 candidate effectors that have not yet been experimentally validated. Because GO annotation coverage was low in both the whole-genome background (560/2,972, 18.8%) and the candidate set (3/224, 1.4%), the available annotations were insufficient for a reliable GO enrichment analysis. We therefore further characterized the candidate proteins based on Pfam domain annotations. Comprehensive annotation using 13 functional databases in InterProScan showed that 170 of the 224 candidate proteins (75.9%) received at least one functional or domain annotation. In the Pfam database used for the subsequent enrichment analysis, 76 candidate proteins (33.9%) were matched to known Pfam domains. Two domains of unknown function (DUF4917 and DUF8197) showed significant enrichment among the predicted candidate proteins (Fold Enrichment = 12.00, $p_{adjust} = 0.012$). These results indicate that the domain composition of the GeoEPred-predicted candidates is not randomly distributed, but is preferentially associated with specific protein families whose functions remain incompletely characterized, providing clues for further investigation of the potential relationships between these domains and effector functions. In addition, Sel1-like repeats, which are associated with *Legionella*--host interactions, also showed an enrichment trend (Fold Enrichment = 4.32, $p = 0.011$). Overall, these findings indicate that the candidate set predicted by GeoEPred exhibits distinct biological structural features and further identifies several protein families and

domains that may warrant subsequent experimental investigation.

These computational analyses are not intended to replace experimental validation; rather, they are designed to assess whether the predicted candidates exhibit biological characteristics consistent with experimentally characterized effector proteins reported in previous studies and to provide biologically informed prioritization of candidate effectors for subsequent functional investigations. We fully acknowledge the importance of experimental validation highlighted by the reviewer and have explicitly acknowledged the absence of direct experimental validation as a limitation in the revised Discussion section. Targeted experimental validation of high-priority candidates will be an important direction for our future studies. We sincerely thank the reviewer again for this insightful suggestion, which has helped us further improve the biological interpretation, transparency, and future applicability of GeoEPred.

The following content constitutes revisions to the main text.

1) We added a description of the enrichment analysis and Pfam domain analysis conducted for the candidate proteins in the "Case Studies" section (Page 17, Lines 526–559).

To further assess the biological relevance of the newly predicted candidates, we performed functional characterization using Gene Ontology (GO) enrichment analysis and protein domain annotation. Enrichment significance was assessed using a hypergeometric test followed by Benjamini–Hochberg correction for multiple testing. In *P. aeruginosa PAO1*, GO enrichment analysis revealed significant overrepresentation of secretion-associated functions, including protein secretion and Type VI secretion system complexes. Pfam domain analysis further identified enrichment of several effector-associated domains, including Sel1-like repeats, ADP-ribosyltransferase domains, and YopE-like GAP domains. Sel1-like repeats have been implicated in Legionella host-cell interactions and virulence-associated processes [48]. ADP-ribosyltransferase domains are characteristic of several bacterial toxins and secreted effectors involved in host cellular modulation, such as the T3SS effector ExoS from Pseudomonas aeruginosa [49]. Similarly, the YopE GAP domain represents a well-characterized effector module that manipulates host Rho GTPase signaling [50]. These observations provide additional functional context supporting the potential biological relevance of the predicted candidates.

In addition, we further evaluated the model on two other Gram-negative bacterial genomes: *Salmonella enterica subsp. enterica serovar Typhimurium str. LT2* (NC_003197.2) and *Legionella pneumophila subsp. pneumophila str. Philadelphia 1* (NC_002942.5). The predicted results were compared against experimentally validated data. GeoEPred reliably identified a large proportion of experimentally confirmed T3SE and T4SE proteins, achieving recall rates of 94.78% (36 out of 38)

and 91.2% (280 out of 307), respectively. These results demonstrate that GeoEPred performs robustly across different biological contexts and maintains reliable performance for large-scale genome-wide annotation tasks.

Functional characterization of predicted candidates from these genomes further supported their biological relevance. In Salmonella Typhimurium LT2, GO enrichment analysis revealed significant associations with secretion-related and extracellular biological processes, including adhesion, biofilm formation, and extracellular enzymatic activities. In Legionella pneumophila Philadelphia-1, where GO annotation coverage was insufficient for reliable enrichment analysis, InterPro and Pfam domain analyses identified several enriched protein domains, including DUF4917, DUF8197, and Sel1-like repeats. Sel1-like repeat-containing proteins have previously been implicated in Legionella virulence and host-cell interaction processes, suggesting that these domain architectures may represent potential molecular features associated with host adaptation [48]. In addition, among the predicted T6SE candidates, enrichment of phage tail- and baseplate-associated domains was observed, which is consistent with the reported evolutionary relationship between the Type VI secretion system and contractile bacteriophage tail structures [51]. These findings suggest that the GeoEPred-predicted candidates exhibit distinct functional and structural characteristics associated with bacterial secretion and host interaction. Detailed functional enrichment and domain annotation results are provided in S7 Fig and S16 Table.

2) We have explicitly acknowledged the lack of direct experimental validation as a limitation in the revised Discussion section. Targeted experimental characterization of high-priority candidate proteins will represent an important direction for future studies. (Page 21, Lines 738–743).

Finally, although this study supports the biological plausibility of the predicted candidate proteins through predictive performance, model interpretation, functional annotation, and genome-scale analyses, the newly predicted candidates have not yet been directly validated experimentally. Therefore, these proteins should currently be regarded as computationally prioritized candidate effectors. Future work may further validate high-priority candidates and systematically evaluate the applicability of GeoEPred to practical effector protein discovery.

3) Newly added Fig 9.

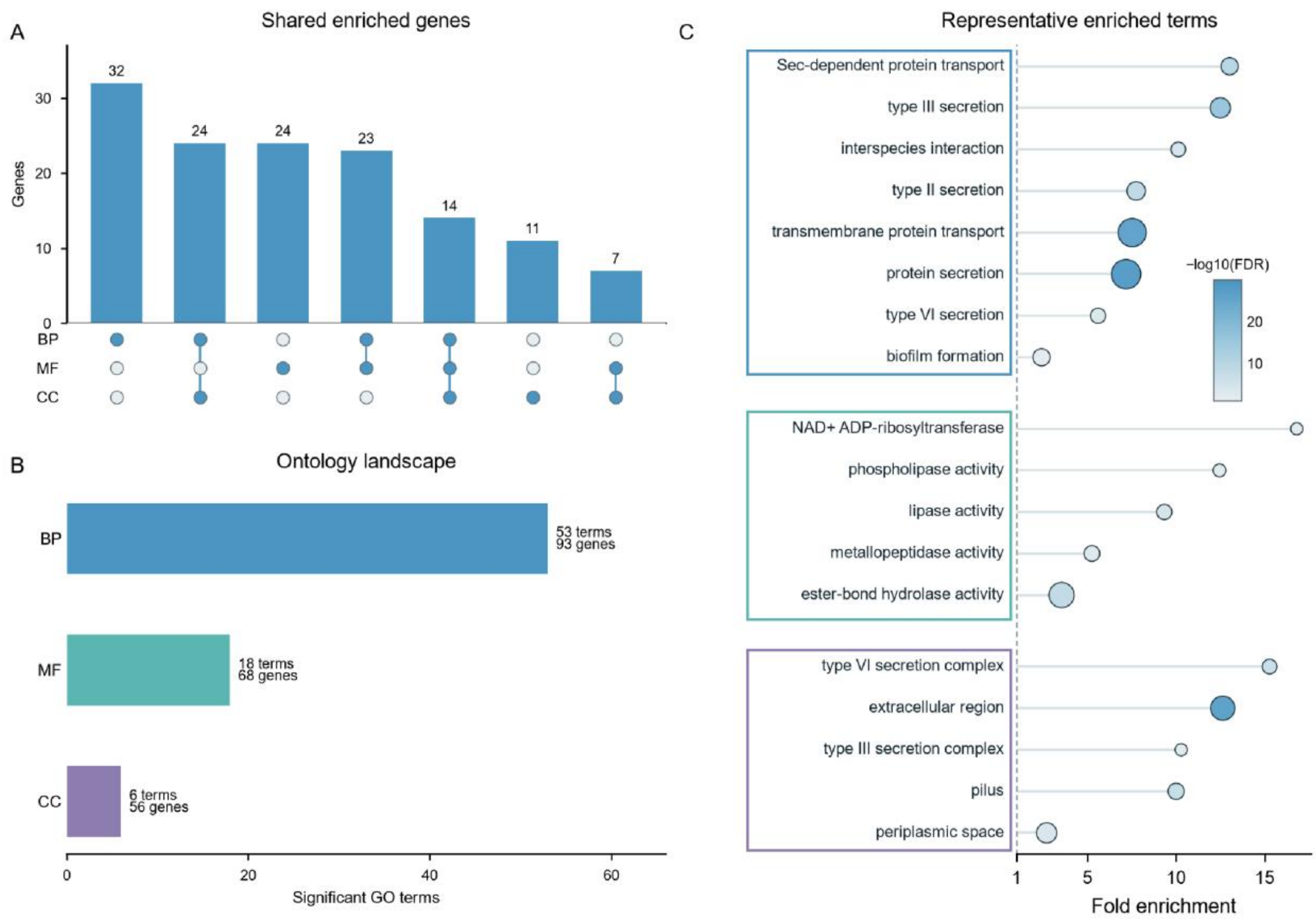

Fig 9. Gene Ontology (GO) functional enrichment analysis of predicted candidate effectors in *Pseudomonas aeruginosa PAO1*. (A) The bar plot displays the number of genes shared among different combinations of Biological Process (BP), Molecular Function (MF), and Cellular Component (CC), as indicated by the connected dot matrix below. (B) Ontology landscape. Overview of the significant GO terms and associated gene counts categorized by BP (blue), MF (teal), and CC (purple). (C) Representative enriched terms. Lollipop charts illustrating representative enriched terms for BP, MF, and CC, respectively. The x-axis represents the fold enrichment, and the bubble size and color intensity correspond to the statistical significance scaled $-\log_{10}(FDR)$.

4) Newly added S7 Fig.

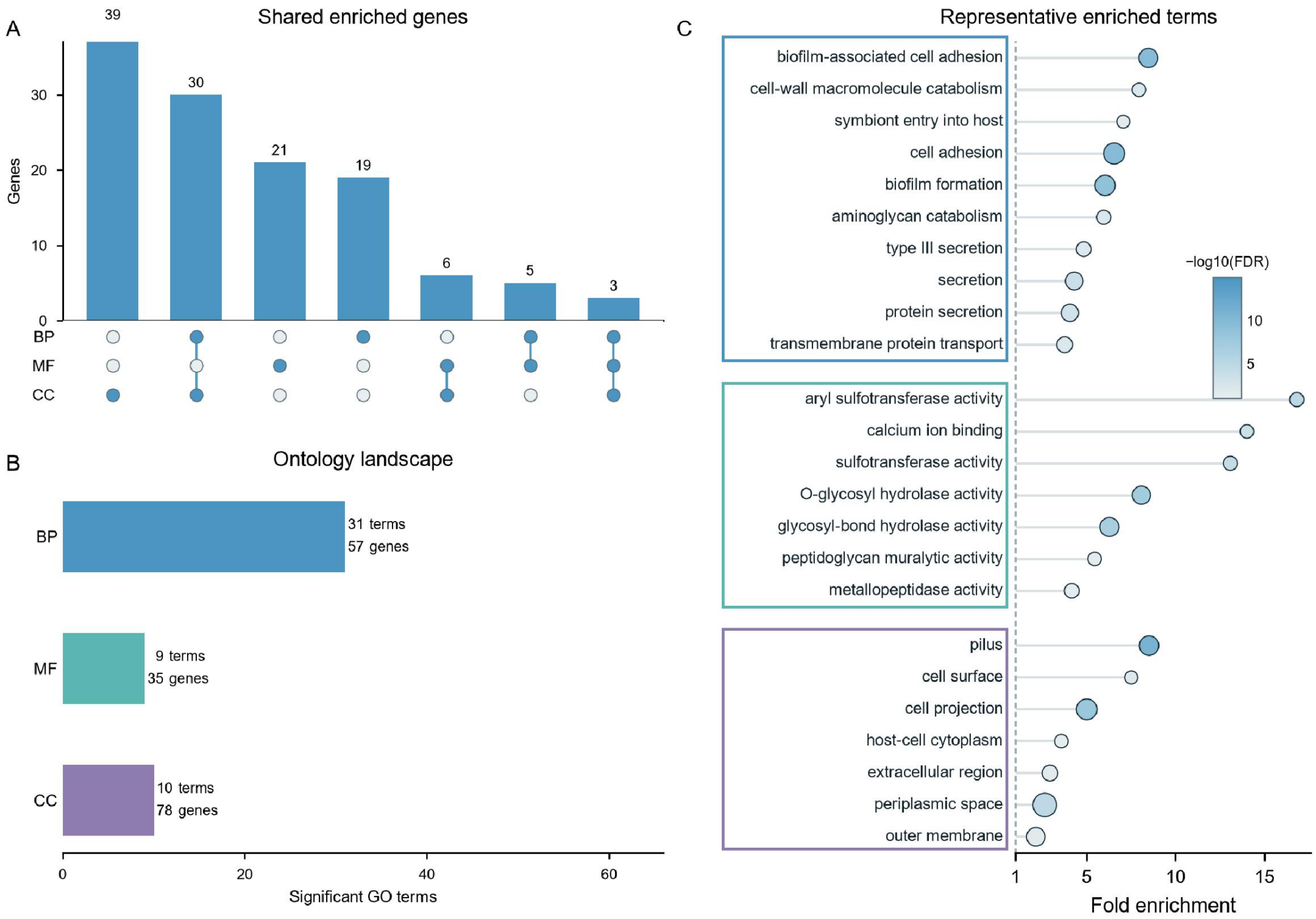

S7 Fig. Gene Ontology (GO) functional enrichment analysis of predicted candidate effectors in *Salmonella Typhimurium LT2*. A: The bar plot displays the number of genes shared among different combinations of Biological Process (BP), Molecular Function (MF), and Cellular Component (CC), as indicated by the connected dot matrix below. B: Ontology landscape. Overview of the significant GO terms and associated gene counts categorized by BP (blue), MF (teal), and CC (purple). C: Representative enriched terms. Lollipop charts illustrating representative enriched terms for BP, MF, and CC, respectively. The x-axis represents the fold enrichment, and the bubble size and color intensity correspond to the statistical significance scaled $-\log_{10}(FDR)$.

5) Newly added S16 Table.

S16 Table. Pfam domain enrichment analysis across candidate effector proteins in three bacterial genomes ($p_{adjust} < 0.05$).

Strain	Class	Pfam ID	Domain description	p_{adjust}
NC_002516.2	T6SE	PF00126	LysR family HTH domain	4.95e-06
	T6SE	PF03466	LysR substrate-binding domain	6.60e-07
	T6SE	PF05954	Phage baseplate hub (GPD)	3.43e-06
	T6SE	PF04717	T6SS/phage baseplate tail tube OB	1.76e-05
	T6SE	PF22178	Phage T4 gp5 trimerisation domain	8.82e-05
NC_002942.5	T6SE	PF16263	Domain of unknown function(DUF4917)	1.47e-02
	T3SE	PF26620	Domain of unknown function(DUF8197)	1.96e-02
NC_003197.2	T4SE	PF08238	Sel1 repeat	2.39e-02
	T6SE	PF05638	T6SS effector protein Hcp	4.21e-02
	T6SE	PF04717	T6SS/phage baseplate OB	4.21e-02
	T6SE	PF02368	Bacterial Ig-like domain	4.21e-02
	T6SE	PF05954	Phage baseplate hub	4.21e-02
	T6SE	PF16461	Lambda phage tail tube protein TTP	4.21e-02
