# Supplementary material for: GeoEPred: A multimodal structure-aware geometric deep learning framework for Gram-negative bacterial secreted effector prediction with sequence semantics
Source: PLoS Comput Biol. 2026 Sep 30;22(9):e1014344. doi: 10.1371/journal.pcbi.1014344 (PMC13641719; doi:10.1371/journal.pcbi.1014344)
Supplement: Peer review history

# Peer review: Decision Letter 0

**DOI:** 10.1371/journal.pcbi.1014344.r001

**Authors:** Can Yang, Rahul Singh

20 Jul 2026

GeoEPred: A Multimodal Structure-Aware Geometric Deep Learning Framework for Gram-Negative Bacterial Secreted Effector Prediction with Sequence Semantics

PLOS Computational Biology

Dear Dr. Shi,

Thank you for submitting your manuscript to PLOS Computational Biology. After careful consideration, we feel that it has merit but does not fully meet PLOS Computational Biology's publication criteria as it currently stands. Therefore, we invite you to submit a revised version of the manuscript that addresses the points raised during the review process.

Please submit your revised manuscript by Sep 18 2026 11:59PM. If you will need more time than this to complete your revisions, please reply to this message or contact the journal office at ploscompbiol@plos.org. When you're ready to submit your revision, log on to https://www.editorialmanager.com/pcompbiol/ and select the 'Submissions Needing Revision' folder to locate your manuscript file.

Please include the following items when submitting your revised manuscript:

* A letter that responds to each point raised by the editor and reviewer(s). You should upload this letter as a separate file labeled 'Response to Reviewers'. This file does not need to include responses to formatting updates and technical items listed in the 'Journal Requirements' section below.

* A marked-up copy of your manuscript that highlights changes made to the original version. You should upload this as a separate file labeled 'Revised Manuscript with Track Changes'.

* An unmarked version of your revised paper without tracked changes. You should upload this as a separate file labeled 'Manuscript'.

Kind regards,

Rahul Singh, Ph.D.

Academic Editor

PLOS Computational Biology

Can Yang

Section Editor

PLOS Computational Biology

**Additional Editor Comments (if provided):**

**Journal Requirements:**

If the reviewer comments include a recommendation to cite specific previously published works, please review and evaluate these publications to determine whether they are relevant and should be cited. There is no requirement to cite these works unless the editor has indicated otherwise.

1) We ask that a manuscript source file is provided at Revision. Please upload your manuscript file as a .doc, .docx, .rtf or .tex. If you are providing a .tex file, please upload it under the item type u2018LaTeX Source Fileu2019 and leave your .pdf version as the item type u2018Manuscriptu2019.

2) Please upload all main figures as separate Figure files in .tif or .eps format. For more information about how to convert and format your figure files please see our guidelines:

https://journals.plos.org/ploscompbiol/s/figures

3) Please amend your detailed Financial Disclosure statement. This is published with the article. It must therefore be completed in full sentences and contain the exact wording you wish to be published.

i) State the initials, alongside each funding source, of each author to receive each grant. For example: "This work was supported by the National Institutes of Health (####### to AM; ###### to CJ) and the National Science Foundation (###### to AM)."

ii) State what role the funders took in the study. If the funders had no role in your study, please state: "The funders had no role in study design, data collection and analysis, decision to publish, or preparation of the manuscript."

iii) If any authors received a salary from any of your funders, please state which authors and which funders..

If you did not receive any funding for this study, please simply state: u201cThe authors received no specific funding for this work.u201d.

**Reviewers' comments:**

Reviewer's Responses to Questions

**Comments to the Authors:**

**Please note here if the review is uploaded as an attachment.**

Reviewer #1: This manuscript proposes GeoEPred, a multimodal deep learning framework for Gram-negative bacterial effector prediction by combining protein language model embeddings with structure-aware geometric learning. The manuscript is generally well organized and supported by extensive experiments. However, several concerns should be addressed to strengthen the scientific rigor and reliability of the study before accepting it for publication. The major comments are listed below.

1.The manuscript does not clearly demonstrate that the training, validation, and independent benchmark datasets are completely non-overlapping. The authors should provide evidence of redundancy removal across datasets and ensure that no homologous proteins are shared between training and test sets.

2.The study relies heavily on ESMFold-predicted structures. The authors should assess how structural confidence (e.g., pLDDT scores) influences model performance and validate the findings using experimentally resolved protein structures if available.

3.The computational complexity of the proposed framework is not discussed. The authors should report training time, inference speed, GPU requirements etc. for genome-wide applications.

4.Statistical significance tests should be performed to demonstrate that the observed performance gains are meaningful.

5.The construction of the remote homology benchmark is insufficiently described. The manuscript should explain how homologs were identified, how TM-scores were calculated, which structural alignment tools were used, and how benchmark independence was ensured.

6.The biological interpretability analysis remains largely qualitative. The highlighted residues and structural regions should be quantitatively compared with experimentally validated conserved motifs, catalytic residues, or known functional regions.

7.The comparison with existing methods may not be entirely fair. Several competing methods were evaluated using predictions from web servers rather than under identical preprocessing and evaluation conditions. The authors should discuss this limitation or perform standardized comparisons whenever possible.

8.No statistical analysis accompanies the reported performance metrics. Confidence intervals, repeated experiments with different random seeds should be included for all major comparisons.

9.The manuscript does not adequately demonstrate the generalizability of GeoEPred beyond the evaluated Gram-negative secretion systems. Additional discussion or validation on other secretion systems or independent external datasets would strengthen the claims of broad applicability.

10.Several implementation details required for reproducibility are missing. The manuscript should provide complete information regarding optimizer settings, learning rate schedule, batch size, early stopping criteria, random seed initialization, hardware configuration, and hyperparameter optimization strategy.

Reviewer #2: The manuscript presents a promising multimodal framework, but the dataset construction lacks sufficient detail to ensure reproducibility. The authors should explicitly describe how positive and negative samples were selected, whether homologous proteins were removed between training and test sets, and how data leakage was prevented beyond the reported 60% CD-HIT filtering.

The manuscript reports superior performance across multiple benchmarks, yet the statistical significance of the improvements is not evaluated. The authors should perform statistical tests to demonstrate that the reported performance gains over competing methods are statistically meaningful.

The manuscript relies entirely on ESMFold-predicted structures without discussing the influence of prediction confidence on downstream performance. The authors should investigate the effect of low-confidence structural regions to validate the robustness of the structural branch.

The manuscript compares GeoEPred with state-of-the-art methods using predictions retrieved from official web servers, but it remains unclear whether all models were evaluated under identical datasets, thresholds, and preprocessing conditions. The authors should ensure a fair comparison by describing the evaluation protocol in detail or by reimplementing the competing methods under standardized conditions.

The manuscript introduces several architectural components, including contrastive learning, feature tokenization, and adaptive attention pooling, but the contribution of each individual module is not fully isolated. The authors should include additional ablation experiments that independently remove each component to quantify its unique contribution to the overall performance.

The manuscript claims strong generalization capability; however, the evaluation is limited to benchmark datasets derived from similar sources. The authors should validate the model on completely independent external datasets or newly collected bacterial genomes to better demonstrate real-world applicability and generalization. Refer to this article: PMID-39657879 to improve the methodology, result, and discussion sections which will be more beneficial for the readership of the article and journal.

The manuscript reports nearly perfect performance for some prediction tasks, particularly T6SE, which raises concerns regarding potential dataset bias or overfitting. The authors should discuss this unusually high performance, provide additional validation analyses, and examine whether class similarity, sample redundancy, or dataset composition contributed to these results.

The manuscript would benefit from a more comprehensive description of the training procedure and computational settings. The authors should report essential implementation details such as optimizer, learning rate schedule, batch size, early stopping criteria, number of epochs, random seed, hardware configuration, and computational time to facilitate reproducibility.

Reviewer #3: The manuscript presents an interesting computational framework and is generally well organized. However, several issues should be addressed to improve the study.

1. In my opinion authors should provide a clearer description of the dataset construction process, including the criteria for sequence inclusion/exclusion and the rationale for selecting the sequence identity threshold.

2. Pretrained protein language models and structure prediction methods are used, the authors should clarify whether proteins in the independent test set were represented in the pretraining datasets and discuss the potential impact on performance evaluation.

3. The T6SE prediction results show perfect performance (ACC, F1, and MCC = 1.000). Such exceptional performance is unusual for biological datasets and raises concerns regarding dataset redundancy, information leakage, or potential overfitting. Justification is required for this.

4. In Table 1, the "Structure only" model exhibits very poor accuracy (50.76%) despite the manuscript emphasizing the importance of structural information. This suggests that structural representations alone have limited discriminative power, and the authors should discuss why geometric features fail to perform independently.

5. The multimodal model achieves only a marginal improvement in AUC compared with the sequence semantic model (98.44% vs. 98.48%), despite a substantial increase in ACC and MCC. This discrepancy should be explained.

6. The authors claim that feature tokenization substantially outperforms cross-attention and concatenation strategies. However, the differences among fusion strategies are relatively modest, and no statistical tests are presented to establish significance. Explain why?

7. The discussion states that GeoEPred accurately identifies validated effectors and discovers novel candidates, yet no functional enrichment, domain analysis, or literature evidence is provided for the newly predicted proteins.

8. The authors must provide separate, class-stratified SHAP swarm plots for each of the six target classes (Non-effector, T1SE, T2SE, T3SE, T4SE, T6SE) to show whether the feature utilization profiles shift for the minority classes. Additionally, they must implement a balanced metric comparison if possible.

9. If possible a quantitative empirical p-value for the co-localization claims should be provided.

Reviewer #4: Manuscript is interesting I have some major queries

1.Authors needs to evaluate geopred on completely independent data sets

2.Needs to clarify the homologous proteins between training and testing datasets were removed??

3.Proteins from the same bacterial Species appears in both sets?

4.This manuscript is focused on computational performance no biological validation is seen, I suggest that validate the data biologically

**********

**Have the authors made all data and (if applicable) computational code underlying the findings in their manuscript fully available?**

The PLOS Data policy requires authors to make all data and code underlying the findings described in their manuscript fully available without restriction, with rare exception (please refer to the Data Availability Statement in the manuscript PDF file). The data and code should be provided as part of the manuscript or its supporting information, or deposited to a public repository. For example, in addition to summary statistics, the data points behind means, medians and variance measures should be available. If there are restrictions on publicly sharing data or code —e.g. participant privacy or use of data from a third party—those must be specified.

Reviewer #1: Yes

Reviewer #2: Yes

Reviewer #3: **No:**

Reviewer #4: Yes

**********

PLOS authors have the option to publish the peer review history of their article (what does this mean?). If published, this will include your full peer review and any attached files.

If you choose “no”, your identity will remain anonymous but your review may still be made public.

**Do you want your identity to be public for this peer review?**  For information about this choice, including consent withdrawal, please see our Privacy Policy.

Reviewer #1: No

Reviewer #2: No

Reviewer #3: No

Reviewer #4: No

[NOTE: If reviewer comments were submitted as an attachment file, they will be attached to this email and accessible via the submission site. Please log into your account, locate the manuscript record, and check for the action link "View Attachments". If this link does not appear, there are no attachment files.]

**Figure resubmission:**

**Reproducibility:**

?>

# Author comment: Author response to Decision Letter 1

**DOI:** 10.1371/journal.pcbi.1014344.r002

30 Aug 2026

Attachment Submitted filename: Response to Reviewers.pdf

# Peer review: Decision Letter 1

**DOI:** 10.1371/journal.pcbi.1014344.r003

**Authors:** Can Yang, Rahul Singh

21 Sep 2026

Dear Prof. Shi,

We are pleased to inform you that your manuscript 'GeoEPred: A Multimodal Structure-Aware Geometric Deep Learning Framework for Gram-Negative Bacterial Secreted Effector Prediction with Sequence Semantics' has been provisionally accepted for publication in PLOS Computational Biology.

Before your manuscript can be formally accepted you will need to complete some formatting changes, which you will receive in a follow up email. A member of our team will be in touch with a set of requests.

Please note that your manuscript will not be scheduled for publication until you have made the required changes, so a swift response is appreciated.

IMPORTANT: The editorial review process is now complete. PLOS will only permit corrections to spelling, formatting or significant scientific errors from this point onwards. Requests for major changes, or any which affect the scientific understanding of your work, will cause delays to the publication date of your manuscript.

Should you, your institution's press office or the journal office choose to press release your paper, you will automatically be opted out of early publication. We ask that you notify us now if you or your institution is planning to press release the article. All press must be co-ordinated with PLOS.

Thank you again for supporting Open Access publishing; we are looking forward to publishing your work in PLOS Computational Biology.

Best regards,

Rahul Singh, Ph.D.

Academic Editor

PLOS Computational Biology

Can Yang

Section Editor

PLOS Computational Biology

***********************************************************

Reviewer's Responses to Questions

**Comments to the Authors:**

**Please note here if the review is uploaded as an attachment.**

Reviewer #1: The authors have addressed the concerns raised during the revision process. This MS can be accepted for publication.

Reviewer #2: The reviewers have responded well to the queries and questions of the reviewers. The manuscript is now worthy of publication.

Reviewer #3: Authors have addressed all the comments.

**********

**Have the authors made all data and (if applicable) computational code underlying the findings in their manuscript fully available?**

The PLOS Data policy requires authors to make all data and code underlying the findings described in their manuscript fully available without restriction, with rare exception (please refer to the Data Availability Statement in the manuscript PDF file). The data and code should be provided as part of the manuscript or its supporting information, or deposited to a public repository. For example, in addition to summary statistics, the data points behind means, medians and variance measures should be available. If there are restrictions on publicly sharing data or code —e.g. participant privacy or use of data from a third party—those must be specified.

Reviewer #1: Yes

Reviewer #2: None

Reviewer #3: Yes

**********

PLOS authors have the option to publish the peer review history of their article (what does this mean?). If published, this will include your full peer review and any attached files.

If you choose “no”, your identity will remain anonymous but your review may still be made public.

**Do you want your identity to be public for this peer review?**  For information about this choice, including consent withdrawal, please see our Privacy Policy.

Reviewer #1: No

Reviewer #2: No

Reviewer #3: No

# Editor report: Acceptance letter

**DOI:** 10.1371/journal.pcbi.1014344.r004

**Authors:** Can Yang, Rahul Singh

PCOMPBIOL-D-26-01167R1

GeoEPred: A Multimodal Structure-Aware Geometric Deep Learning Framework for Gram-Negative Bacterial Secreted Effector Prediction with Sequence Semantics

Dear Dr Shi,

I am pleased to inform you that your manuscript has been formally accepted for publication in PLOS Computational Biology. Your manuscript is now with our production department and you will be notified of the publication date in due course.

The corresponding author will soon be receiving a typeset proof for review, to ensure errors have not been introduced during production. Please review the PDF proof of your manuscript carefully, as this is the last chance to correct any errors. Please note that major changes, or those which affect the scientific understanding of the work, will likely cause delays to the publication date of your manuscript.

Soon after your final files are uploaded, unless you have opted out, the early version of your manuscript will be published online. The date of the early version will be your article's publication date. The final article will be published to the same URL, and all versions of the paper will be accessible to readers.

For Research, Software, and Methods articles, you will receive an invoice from PLOS for your publication fee after your manuscript has reached the completed accept phase. If you receive an email requesting payment before acceptance or for any other service, this may be a phishing scheme. Learn how to identify phishing emails and protect your accounts at https://explore.plos.org/phishing.

Thank you again for supporting PLOS Computational Biology and open-access publishing. We are looking forward to publishing your work!

With kind regards,

Janani Seenivasan

PLOS Computational Biology | Carlyle House, Carlyle Road, Cambridge CB4 3DN | United Kingdom ploscompbiol@plos.org | Phone +44 (0) 1223-442824 | ploscompbiol.org | @PLOSCompBiol
